# Biosynthesis and antifungal activity of fungus-induced *O*-methylated flavonoids in maize

**DOI:** 10.1093/plphys/kiab496

**Published:** 2021-10-27

**Authors:** Christiane Förster, Vinzenz Handrick, Yezhang Ding, Yoko Nakamura, Christian Paetz, Bernd Schneider, Gabriel Castro-Falcón, Chambers C Hughes, Katrin Luck, Sowmya Poosapati, Grit Kunert, Alisa Huffaker, Jonathan Gershenzon, Eric A Schmelz, Tobias G Köllner

**Affiliations:** 1 Department of Biochemistry, Max Planck Institute for Chemical Ecology, Jena D-07745, Germany; 2 Section of Cell and Developmental Biology, University of California, San Diego, California 92093-0380, USA; 3 Research Group Biosynthesis/NMR, Max Planck Institute for Chemical Ecology, Jena D-07745, Germany; 4 Scripps Institution of Oceanography, University of California, San Diego, California 92093, USA

## Abstract

Fungal infection of grasses, including rice (*Oryza sativa*), sorghum (*Sorghum bicolor*), and barley (*Hordeum vulgare*), induces the formation and accumulation of flavonoid phytoalexins. In maize (*Zea mays*), however, investigators have emphasized benzoxazinoid and terpenoid phytoalexins, and comparatively little is known about flavonoid induction in response to pathogens. Here, we examined fungus-elicited flavonoid metabolism in maize and identified key biosynthetic enzymes involved in the formation of *O*-methylflavonoids. The predominant end products were identified as two tautomers of a 2-hydroxynaringenin-derived compound termed xilonenin, which significantly inhibited the growth of two maize pathogens, *Fusarium graminearum* and *Fusarium verticillioides*. Among the biosynthetic enzymes identified were two *O*-methyltransferases (OMTs), flavonoid OMT 2 (FOMT2), and FOMT4, which demonstrated distinct regiospecificity on a broad spectrum of flavonoid classes. In addition, a cytochrome P450 monooxygenase (CYP) in the CYP93G subfamily was found to serve as a flavanone 2-hydroxylase providing the substrate for FOMT2-catalyzed formation of xilonenin. In summary, maize produces a diverse blend of *O*-methylflavonoids with antifungal activity upon attack by a broad range of fungi.

## Teaser

Upon fungal infection, maize produces a complex mixture of *O*-methylated flavonoids, which are biosynthesized by regiospecific *O*-methyltransferases and that contribute to the blend of defense-related specialized metabolites.

## Introduction

Plants dynamically deploy a suite of low-molecular weight metabolites to protect against pathogen infection that is chemically diverse and often species-specific. When these compounds are produced in response to microbial challenge or other environmental stresses, they have been termed phytoalexins (VanEtten et al., 1994; Hammerschmidt, 1999). Rapid phytoalexin biosynthesis is often associated with enhanced pathogen resistance (Hain et al., 1993; He and Dixon, 2000). Phytoalexins have representatives from many known classes of specialized metabolites (Jeandet et al., 2014), including the stilbene resveratrol in grapes (*Vitis vinifera*; Langcake and Pryce, 1976) and an indole thiazole alkaloid, termed camalexin, in Arabidopsis (*Arabidopsis thaliana*; Browne et al., 1991). In maize (*Zea mays*), complex networks of sesquiterpenoid and diterpenoid phytoalexins have been described, which include zealexins, kauralexins, and dolabralexins (Huffaker et al., 2011; Schmelz et al., 2011; Mafu et al., 2018; Ding et al., 2020).

Many phytoalexins are flavonoids, a large group of phenylpropanoid and polyketide-derived metabolites present in all plants (Tohge et al., 2017; de Souza et al., 2020; Ube et al., 2021). The accumulation of flavonoids after pathogen infection has been demonstrated to play a role in disease resistance in several plants, such as for the 3-deoxyanthocyanidins of sorghum (*Sorghum bicolor*) (Nicholson et al., 1987; Snyder et al., 1991; Liu et al., 2010) and the flavan-3-ols of poplar (Ullah et al., 2017).

The core pathways of flavonoid biosynthesis are well conserved among plant species (Grotewold, 2006; Tohge et al., 2017). The first step is the condensation of a phenylpropanoid derivative, 4-coumaroyl-CoA, with three malonyl-CoA subunits catalyzed by a polyketide synthase, chalcone synthase. The naringenin chalcone produced is then cyclized by chalcone isomerase to form flavanones, which are converted successively to dihydroflavonols and flavonols by soluble Fe^2+^/2-oxoglutarate-dependent dioxygenases (2-ODDs). Flavanones can also be desaturated to form flavones via different mechanisms. While flavone synthases of type I (FNSI) belong to the 2-ODDs, FNSII are membrane-bound oxygen- and nicotinamide adenine dinucleotide phosphate(NADPH)-dependent cytochrome P450 monooxygenases (CYPs; Martens and Mithofer, 2005; Jiang et al., 2016).

Other common modifications of the flavonoid backbone include *C*- and *O*-glycosylation, acylation, and *O*-methylation (Grotewold, 2006). *O*-Methylation of flavonoids is catalyzed by *O*-methyltransferases (OMTs), which transfer the methyl group of the cosubstrate *S*-adenosyl-L-methionine (SAM) to a specific hydroxyl group of the flavonoid. Two major classes of plant phenylpropanoid OMTs exist; the caffeoyl-CoA OMTs (CCoAOMTs) of low-molecular weight (26–30 kDa) that require bivalent ions for catalytic activity, and the higher molecular weight (40–43 kDa) and bivalent ion-independent caffeic acid OMTs (COMTs). Flavonoid OMTs (FOMTs) are members of the COMT class (Kim et al., 2010). *O*-Methylation modifies the chemical properties of flavonoids and can alter biological activity, depending on the position of reaction (Kim et al., 2010). In general, the reactivity of hydroxyl groups is reduced coincident with increased lipophilicity and antimicrobial activity (Ibrahim et al., 1998).

Many *FOMT* genes have been cloned from dicot species and the corresponding enzymes biochemically characterized (Kim et al., 2010; Berim et al., 2012; Liu et al., 2020). In contrast, only a few *FOMT* genes from monocotyledons, all belonging to the grass family (*Poaceae*), have been functionally characterized so far. Four FOMTs from rice (*Oryza sativa*), wheat (*Triticum aestivum*), barley (*Hordeum vulgare*), and maize are flavonoid 3′-/5′-OMTs that prefer the flavone tricetin as substrate (Kim et al., 2006; Zhou et al., 2006a, 2006b, 2008). The other two known *Poaceae* FOMTs are flavonoid 7-OMTs from barley and rice that mainly utilize apigenin and naringenin as substrates, respectively (Christensen et al., 1998; Shimizu et al., 2012). In both cases, the gene transcripts or FOMT reaction products, namely 7-methoxyapigenin (genkwanin) and 7-methoxynaringenin (sakuranetin) accumulated in leaves following challenge with pathogenic fungi or abiotic stress (Gregersen et al., 1994; Rakwal et al., 1996). Moreover, genkwanin and sakuranetin were shown to possess antibacterial and antifungal activity in vitro (Kodama et al., 1992; Martini et al., 2004; Park et al., 2014). Sakuranetin also inhibits the growth of the rice blast fungus (*Magnaporthe oryzae*) in vivo (Hasegawa et al., 2014). Despite our knowledge of the key pathogen protection roles of *O*-methylflavonoids in rice, their biosynthesis has not been previously described in maize.

To investigate fungal-induced defenses in maize, we used untargeted and targeted liquid chromatography/mass spectrometry (LC–MS) to identify and quantify flavonoids in leaves of different inbred lines infected with a necrotrophic fungus, southern leaf blight (SLB; *Bipolaris maydis*). *O*-Methylflavonoids were especially plentiful with the most abundant compound being a tautomeric *O*-dimethyl-2-hydroxynaringenin termed xilonenin, which exhibited small but significant in vitro antifungal activity against *Fusarium graminearum* and *Fusarium* *verticillioides*. Association mapping and RNA-Seq-based transcriptome analyses enabled the selection of candidate pathway genes encoding a CYP93G and three highly regiospecific OMTs.

## Results

### Fungal elicitation of maize results in the accumulation of a complex mixture of flavonoids, especially *O*-methylflavonoids

**Figure 1:**
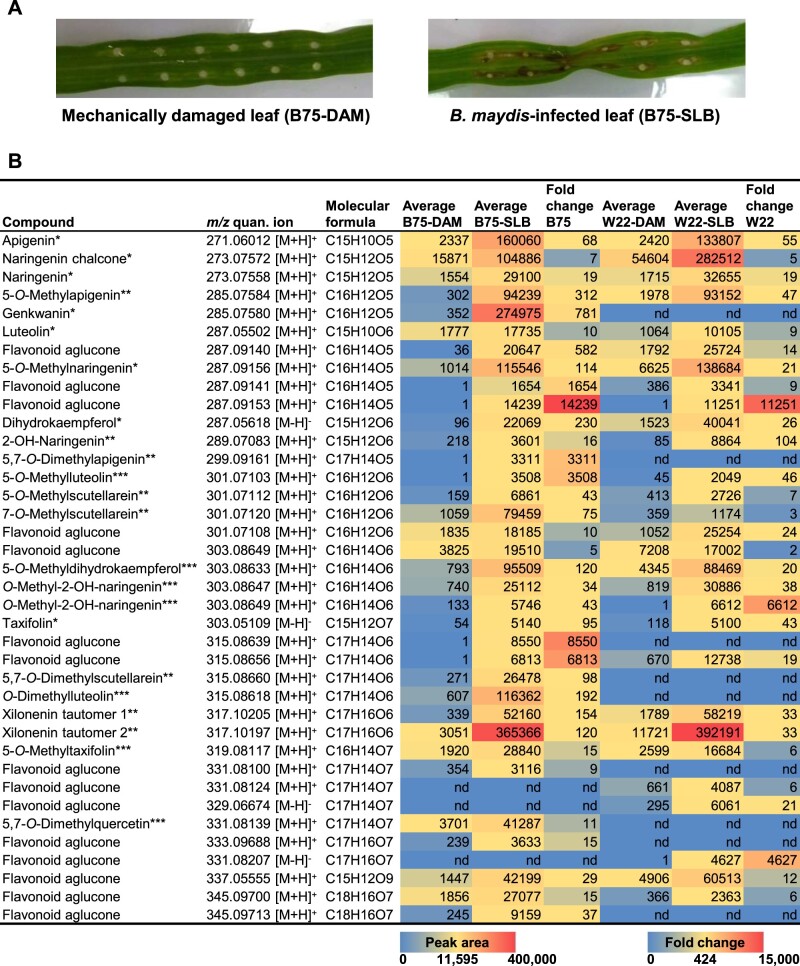
Flavonoids induced in maize leaves upon fungal infection. Damaged and water treated leaves (DAM) or damaged and *B. maydis*-infected leaves (SLB) of the maize lines B75 and W22 were harvested 4 d after inoculation. Methanol extracts made from ground leaf material were screened for putative non-*O*-methyl- and *O*-methylflavonoids by untargeted LC–MS based on the expected exact masses. A, Representative photographs of water control (DAM) and *B. maydis*-infected (SLB) B75 leaves. B, Potential flavonoids were tentatively identified using their exact masses. Only compounds with Δ *m/z* ≤ 2 ppm, a peak area more than 3,000, and a fold change of ≥ 5 after fungal infection were included in the candidate list. Mean relative abundances and fold changes are shown (*n* = 6–8). The differences between treatments are statistically significant (*P* < 0.05) for all compounds in both lines (t test implemented in MetaboScape version 4.0 software; for *P*-values see Supplemental Table S1). The identities of compounds were confirmed by commercially available standards (*), purification followed by NMR analysis (**), or inferred from specific enzymatic activities investigated in this study (***, see below).

To identify flavonoids induced by fungal infection in maize, we used untargeted LC–MS to screen for metabolites present in the leaves of two inbred lines (B75 and W22) infected with the pathogenic fungus *B. maydis*, termed SLB (Figure  1A). Based on the accurate mass (Δ *m/z* ≤ 2 ppm), we identified a collection of 38 known and putative flavonoids showing increased accumulation following fungal infection (Figure  1B;Supplemental Table S1). Flavonoid levels differed qualitatively and quantitatively between the two inbreds; however, MS/MS fragmentation patterns indicated that flavonoids with methoxy groups at positions 5 and 7 of the A-ring predominated in both lines (Figure  1B;Supplemental Figure S1). We were able to confirm the structures of 7 non-*O*-methylated flavonoids and 16 *O*-methyl- or *O*-dimethylflavonoids by authentic standards, nuclear magnetic resonance (NMR) analysis, or by deducing their *O*-methylation patterns from specific enzymatic activities as detailed below.

### Two maize *OMT* genes on chromosome 9 are genetically associated with *O*-methylflavonoid accumulation

**Figure 2:**
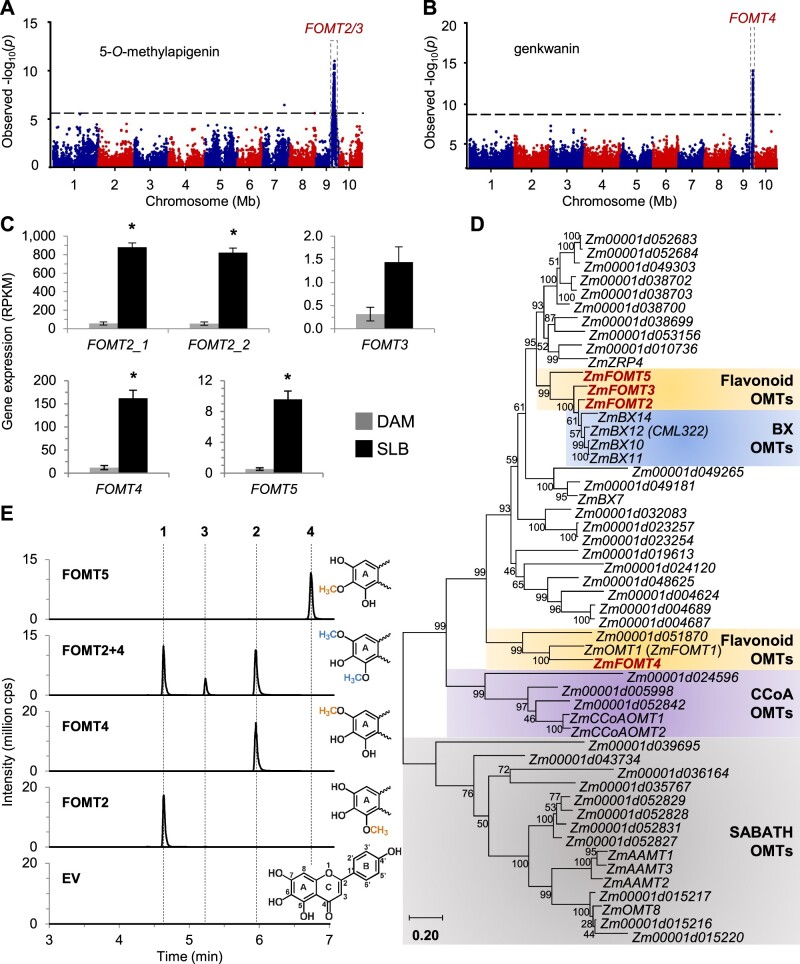
Association mapping reveals novel *O*-methyltransferases involved in maize *O*-methylflavonoid production. A, Manhattan plot of the association analysis of fungus-elicited 5-*O*-methylapigenin using the B73 × Ky21 RIL population with the GLM and 80,440 SNPs. The most statistically significant SNPs are located within the region of the maize *FOMT2/3* genes on chromosome 9 (*FOMT2*, Chr.9:119,779,040–119,780,565 bp; *FOMT3*, Chr9: 119,838,646–119,840,122 bp; B73 RefGen_v2). The black dashed line denotes the false discovery rate (< 0.05 at –log10[P]) using a Bonferroni correction. B, Manhattan plot of the association analysis (MLM) of genkwanin in the stems of maize plants from the Goodman diversity panel following 3 d of fungal elicitation. The most statistically significant SNPs are located within the region of the maize *FOMT4* gene on chromosome 9 (Chr9: 147,148,251–147,149,436 bp; B73 RefGen_v3). The black dashed line denotes the 5% Bonferroni corrected threshold for 25,457,708 SNP markers. C, Transcript abundance of identified *OMT* genes in damaged and water-treated (DAM) or damaged and *B. maydis*-infected (SLB) W22 leaves harvested after 4 d of inoculation. Gene expression is given as reads per kilobase per million reads mapped (RPKM; means ± SE; *n* = 4). Asterisks indicate statistically significant differences (*P* < 0.05) between treatments using a Bonferroni correction (for statistical values, see Supplemental Table S2). D, Phylogenetic tree showing maize *OMT* genes similar to mapped *FOMT2/3*, previously characterized *AAMT1*, and *CCoAOMT1*. The tree was inferred using the maximum likelihood method based on the General Time Reversible model, including gamma distributed rate variation among sites (+G, 4.3129). Bootstrap values (*n* = 1,000) are shown next to each node. The tree is drawn to scale, with branch lengths measured in the number of substitutions per site. All positions with < 80% site coverage were eliminated. Maize *OMTs* investigated in this study are highlighted in red. Gene accession numbers and references are provided in Supplemental Table S3. E, Enzymatic activity of purified recombinant FOMT2, FOMT4, FOMT5, and an EV control using scutellarein as substrate in the presence of the cosubstrate SAM. Reaction products were analyzed by LC–MS/MS. The structure of the substrate scutellarein (depicting flavonoid ring structure and numbering) and partial structures of the different enzymatic products highlighting the added methyl groups on the flavonoid A-ring are shown on the right side. 1, 5-*O*-methylscutellarein; 2, 7-*O*-methylscutellarein; 3, 5,7-*O*-dimethylscutellarein; 4, hispidulin; cps, counts per second.

To identify candidate OMTs involved in the formation of fungus-elicited *O*-methylflavonoids, we performed association analyses using the mapping traits 5-*O*-methylapigenin and genkwanin (7-*O*-methylapigenin), two of the compounds identified in our survey (Figure  1). Using the B73 × Ky21 recombinant inbred line (RIL) population (McMullen et al., 2009) and 5-*O*-methylapigenin levels, we performed association mapping with the general linear model (GLM) and 80,440 single-nucleotide polymorphisms (SNPs) that identified highly significant SNPs on chromosome 9 (B73 RefGen_v2) (Figure  2A;Supplemental Figure S2). The corresponding chromosomal region contained two putative *OMT* genes named *FOMT2* (*Zm00001d047192*) and *FOMT3* (*Zm00001d047194*). For clarity, unless otherwise noted, gene and protein abbreviations refer to line B73 (RefGen_v4) reference sequences. In addition, a genome-wide association study (GWAS) using the Goodman association panel (mixed linear model (MLM), 25,457,708 SNPs; Flint-Garcia et al., 2005) was performed using genkwanin or the apigenin/genkwanin ratio as traits (Figure  2B;Supplemental Figure S3), which revealed a second genomic region on chromosome 9 containing a third putative *OMT* gene named *FOMT4* (*Zm00001d048087*).

Initial sequence analyses of the identified *OMT* genes in different maize inbred lines revealed that W22 has a second copy of *FOMT2* (*Zm00004b033403* and *Zm00004b033399*, W22 RefGen_v2) on chromosome 9, differing only in a single synonymous nucleotide (Supplemental Figures S4 and S5). Furthermore, *FOMT2* and *FOMT3* are closely related and encode proteins with 79% amino acid sequence identity. RNA sequencing (RNA-seq) of W22 leaves, damaged and treated with either water (control) or *B. maydis* hyphae for 4 d, showed significantly increased accumulation of transcripts encoding both copies of *FOMT2* and *FOMT4* as predicted for their involvement in flavonoid *O*-methylation (Figure  2C;Supplemental Table S2; Supplemental Data Set S1). In contrast, *FOMT3* displayed dramatically lower expression levels that did not show statistically significant differences between the treatments.

Phylogenetic analyses demonstrated that FOMT2/3 are closely related to maize BX10/11/12/14, which catalyze various *O*-methylations of benzoxazinoid (BX) defense compounds (Meihls et al., 2013), and to an uncharacterized maize OMT named FOMT5 (Zm00001d051934) (Figure  2D;Supplemental Figure S6; Supplemental Table S3). Notably, *BX10/11/14* and *FOMT5* transcripts also increased after fungal elicitation in our experiments (Figure  2C;Supplemental Figure S7; Supplemental Table S2). In contrast to FOMT2/3, FOMT4 showed the closest relation to OsNOMT, responsible for production of the phytoalexin sakuranetin in rice, and other Poaceae FOMTs, including maize OMT1 (FOMT1), which has been described to *O*-methylate the B-ring of various flavonoids (Figure  2D;Supplemental Figure S6).

### FOMT2/3, FOMT4, and FOMT5 catalyze the regiospecific *O*-methylation of diverse flavonoids in vitro

To characterize the enzymatic activity of FOMT2, FOMT3, FOMT4, and FOMT5, we expressed the complete open reading frames in *Escherichia coli* and tested the purified recombinant proteins in enzyme assays with potential flavonoid substrates in the presence of the cofactor SAM. Using scutellarein as a substrate, LC–MS/MS analysis revealed that FOMT2, FOMT4, and FOMT5 each produced a different single product peak that was not present in the empty vector (EV) control (Figure  2E). Product purification followed by NMR structure elucidation (Supplemental Table S4; Supplemental Data Set S2) or comparison with commercially available standards confirmed regiospecific *O*-methylation on positions 5, 7, and 6 of the flavonoid A-ring catalyzed by FOMT2, FOMT4, and FOMT5, respectively. In an enzyme assay containing both FOMT2 and FOMT4, a 5,7-*O*-dimethylated product was detected (Figure  2E). Interestingly, the 5-*O*-methylated product (RT = 4.61 min), as well as the 5,7-*O*-dimethylated product (RT = 5.20 min), were retained less by the LC column than the non-*O*-methylated substrate (RT = 5.22 min; Supplemental Figure S8), suggesting that the carbonyl group on the C-ring can form a hydrogen bond to a solvent molecule, which likely makes the two products more polar compared to the substrate. The regiospecificity of FOMT2 and FOMT4 and the distinct elution patterns of their products were confirmed with enzyme assays using naringenin and apigenin as substrates (Supplemental Figure S8), followed by NMR structure verification (Supplemental Table S4; Supplemental Data Set S2), which was used as the basis for the identification of additional 5-/7-*O*-methylflavonoids given in Figure  1B. FOMT3 displayed the same enzymatic activity as FOMT2, producing the 5-*O*-methyl derivative of different flavonoid substrates, but exhibited much lower relative activity (Supplemental Table S5).

**Figure 3:**
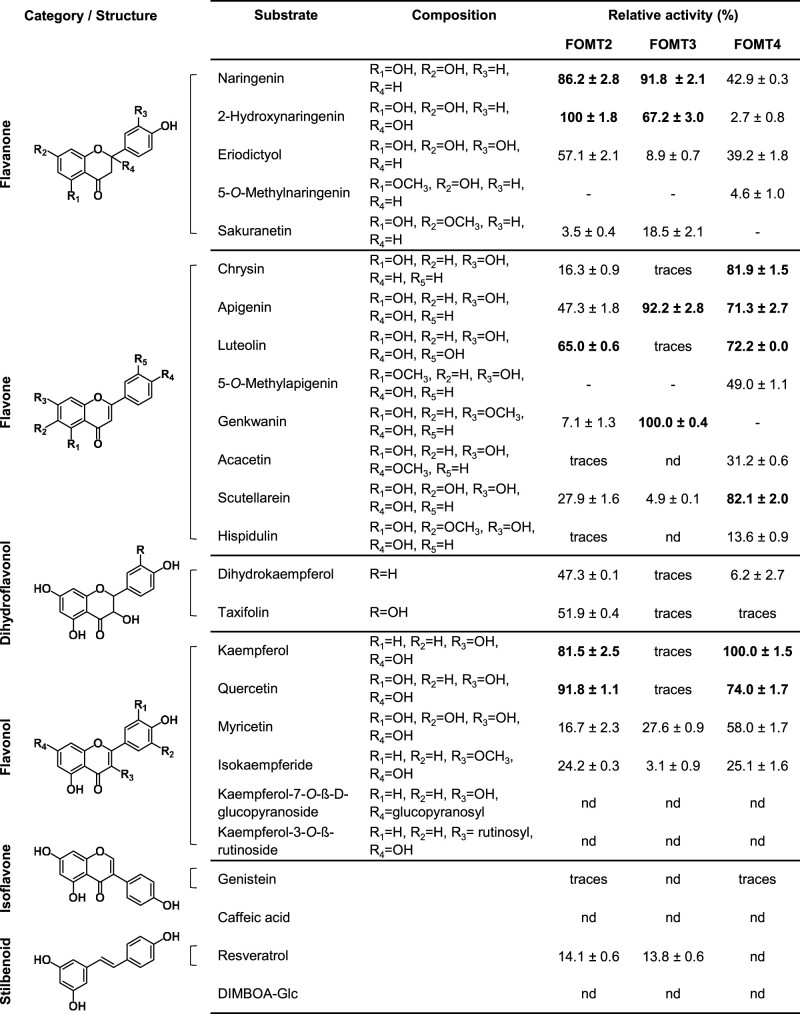
Relative activities of the flavonoid *O*-methyltransferases FOMT2, FOMT3, and FOMT4 with various substrates in vitro. The purified recombinant enzymes as well as the EV control were incubated with the respective substrates in presence of the cosubstrate SAM. Substrate turnover was analyzed by LC–MS/MS and used to estimate the relative activity of each enzyme with different substrates. Product formation in the absence of altered substrate turnover was considered as trace activity. Data are shown as means ± se (*n* = 2–3). nd, not detectable.

Despite their strict regiospecificity, FOMT2/3 and FOMT4 demonstrated an ability to functionalize a range of flavonoid skeletons (Figure  3). Preferred substrates for FOMT2 were flavanones (2-hydroxynaringenin, naringenin) and flavonols (quercetin, kaempferol), while FOMT4 showed highest activity with flavonols (kaempferol, quercetin) and flavones (scutellarein, chrysin, luteolin, apigenin). All three enzymes showed activity, albeit rather low, with *O*-methylflavonoids as substrates. The structurally similar stilbenoid resveratrol was also a substrate for FOMT2/3. Neither the tested glycosylated flavonoids nor the phenolic compounds caffeic acid and DIMBOA-Glc were accepted as substrates by any of the assayed FOMTs (Figure  3). Altogether, the in vitro characterization demonstrated that FOMT2 and FOMT4 in combination are capable of generating the majority of the *O*-methylflavonoids observed in maize.

The phylogenetically related OMTs from BX biosynthesis *BX10/11/14* are also induced upon fungal infection (Supplemental Figure S7). To investigate whether these enzymes might also play a role in *O*-methylflavonoid formation, we included BX10/11/12/14 in our OMT characterization. Besides the expected conversion of DIMBOA-Glc to HDMBOA-Glc (Supplemental Table S5), all four enzymes showed fairly low, but unspecific 5- and/or 7-*O*-methylation activity (<0.9% product formation of FOMT2 or 4) with flavonoid substrates such as naringenin, apigenin, and scutellarein (Supplemental Table S5). The only exception was the direct 5,7-*O*-dimethylation of apigenin by BX10, BX11, and BX12, which exhibited up to 60% of the activity of FOMT2 + 4 (Supplemental Table S5).

### Two predominant fungal-induced *O*-dimethylated flavonoids are 2-hydroxynaringenin derivatives associated with *FOMT2*

**Figure 4:**
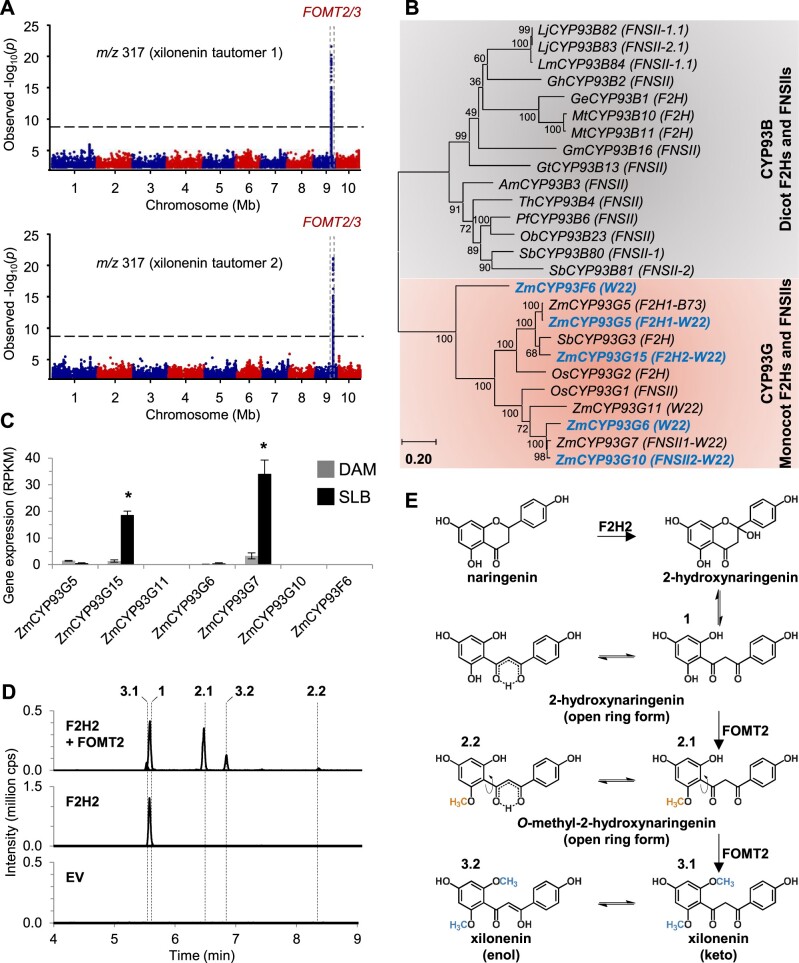
FOMT2 converts 2-hydroxynaringenin to the tautomeric *O*-dimethylated derivative xilonenin. A, Manhattan plots of the association analysis (MLM) of the two novel flavonoids with *m/z* 317 in the stems of maize plants from the Goodman diversity panel following 3 d of fungal elicitation. The most statistically significant SNPs are located within the region of the maize *FOMT2/3* genes on chromosome 9 (*FOMT2*, Chr9: 120,033,582–120,035,107 bp; *FOMT3*, Chr9: 120,093,188–120,094,664 bp; B73 RefGen_v3). The black dashed line denotes the 5% Bonferroni corrected threshold for 25,457,708 SNP markers. B, Phylogenetic analysis of maize genes (W22 NRGene_V2) similar to *F2H1* (B73 RefGen_V3) and additional *F2H* and *FNSII* genes from other monocots and dicots. The tree was inferred using the maximum likelihood method based on the General Time Reversible model, including gamma distributed rate variation among sites (+G, 1.4700) and allowing invariable sites (+I; 8.71% sites). Bootstrap values (*n* = 1,000) are shown next to each node. The tree is drawn to scale, with branch lengths measured in the number of substitutions per site. All positions with < 80% site coverage were eliminated. Maize CYP93Gs investigated in this study are highlighted in blue. Accession numbers and references are provided in Supplemental Table S6. C, Transcript accumulation of F2H candidates in damaged and water-treated leaves (DAM) or damaged and *B. maydis*-infected leaves (SLB) of W22 harvested after 4 d of inoculation. Gene expression is given as RPKM (Means ± se; *n* = 4). Asterisks indicate statistically significant differences (*P* < 0.05) between treatments using a Bonferroni correction (for statistical values, see Supplemental Table S2). D, Enzymatic activity of F2H2 (CYP93G15, Zm00004b010826) alone and in combination with purified recombinant FOMT2 using naringenin as substrate and NADPH and SAM, respectively, as cosubstrates. F2H2 was heterologously expressed in yeast and the microsomal fraction was used in the enzyme assays. Reaction products were analyzed by LC–MS/MS. 1, 2-hydroxynaringenin; 2.1 and 2.2, *O*-methyl-2-hydroxynaringenin; 3.1 and 3.2, xilonenin (keto and enol form, respectively). (E) Proposed reaction scheme for the biosynthesis of xilonenin. RPKM, reads per kilobase per million reads mapped; cps, counts per second.

Two of the most abundant *O*-methylflavonoids detected in our LC–MS profiles of fungal-infected maize leaves had identical accurate masses of *m/z* 317.102 [M + H]^+^ (Figure  1;Supplemental Figure S9), suggesting both were di-*O*-methylated derivatives of a hydroxynaringenin (proposed molecular formula: C_17_H_16_O_6_, Δ *m/z* ≤ 0.14 ppm). In addition, the fragmentation pattern (main fragments: *m/z* 181.050 [M + H]^+^ and *m/z* 121.028 [M + H]^+^), indicated that the hydroxyl group must be connected to a position on the flavonoid C-ring (Supplemental Figure S9). These two major unknowns were accompanied by two other unidentified flavonoids with *m/z* 303.086 [M + H]^+^ (proposed molecular formula: C_16_H_14_O_6_, Δ *m/z* ≤ 0.72 ppm), whose accurate mass and fragmentation pattern were consistent with being mono-*O*-methylated derivatives of a hydroxynaringenin. Moreover, there was also a peak for a non-*O*-methylated flavonoid in SLB-infected W22 leaves that was a potential precursor of these unknowns, which had *m/z* 289.071 [M + H]^+^ (proposed molecular formula: C_15_H_12_O_6_, Δ *m/z* = 0.43 ppm; Supplemental Figure S9). The fragmentation pattern of this precursor candidate was consistent with that reported for 2-hydroxynaringenin (Supplemental Figure S9), which interconverts between closed-ring and open-ring tautomers at room temperature (Zhang et al., 2007; Du et al., 2010a, 2010b). Importantly, in the GWAS as well as the association analysis using the B73 × Ky21 RIL population, *FOMT2* was associated with the occurrence of the two major unknown compounds of *m/z* 317.102 (Figure  4A;Supplemental Figures S2 and S10). We thus hypothesized that the open ring form of 2-hydroxynaringenin could serve as a substrate for two sequential *O*-methylation reactions catalyzed by FOMT2 since rotation of the A-ring creates two equivalent hydroxyl groups.

### A fungal-induced flavanone 2-hydroxylase provides 2-hydroxynaringenin for the production of two open ring tautomeric di-*O*-methylated flavonoid derivatives termed xilonenin

To test whether 2-hydroxynaringenin can act as substrate for FOMT2, we first investigated the formation of this precursor. A flavanone 2-hydroxylase (F2H) converting naringenin to its 2-hydroxy derivative was previously characterized in maize (CYP93G5, F2H1; Morohashi et al., 2012); however, *F2H1* transcript levels in W22 (*Zm00004b033614*) were low and not increased following fungal elicitation (Figures  4, B and C). We, therefore, performed a BLAST analysis of *F2H1* in the W22 (NRGene_V2) genome to identify related genes. This search revealed five additional putative flavanone hydroxylases belonging to the CYP93G subfamily that clustered with characterized monocot F2Hs or FNSIIs in a phylogenetic tree (Figure  4B;Supplemental Table S6; Supplemental Figure S11), but were only distantly related to dicot F2H/FNSII enzymes belonging to the CYP93B subfamily (Du et al., 2010a, 2010b; Morohashi et al., 2012; Lam et al., 2014). Two of these CYP93G genes, *Zm00004b010826* (*CYP93G15*) and *Zm00004b039148* (*CYP93G7*), the latter recently characterized as a FNSII (Righini et al., 2019), were found to be upregulated after fungal infection (Figure  4C;Supplemental Table S2).

To determine the enzymatic activity of Zm00004b010826 (CYP93G15), we expressed the yeast codon-optimized full-length open reading frame in *Saccharomyces cerevisiae* and performed enzyme assays with the microsomal fraction, the cosubstrate NADPH, and the potential substrates naringenin or eriodictyol. The characterized F2H1 from B73 was included as positive control. LC–MS/MS analysis showed that both F2H1 and Zm00004b010826 (CYP93G15) converted naringenin and eriodictyol to 2-hydroxynaringenin and 2-hydroxyeriodictyol, respectively, while the EV control did not show any product peak (Figure  4D;Supplemental Figure S12). Among the other putative F2Hs, Zm00004b033614 (CYP93G5) exhibited F2H activity, converting naringenin and eriodictyol to their respective 2-hydroxy derivatives (Supplemental Figure S12), while Zm00004b008124 (CYP93G10) converted naringenin and eriodictyol to the corresponding flavones apigenin and luteolin, respectively, thus exhibiting FNSII activity (Supplemental Figure S12). Notably, we also detected low amounts of 2-hydroxynaringenin in the CYP93G10 reaction (insert in Supplemental Figure S12), indicating that this compound is likely an intermediate in flavone formation. No in vitro activity with naringenin or eriodictyol was found for Zm00004b039147 (CYP93G6) and Zm00004b033036 (CYP93F6; Supplemental Figure S12). Based on their in vitro activity, Zm00004b010826 (CYP93G15) and Zm00004b008124 (CYP93G10) were designated as F2H2 and FNSII2, respectively.

To test whether the 2-hydroxynaringenin formed by F2H2 is a precursor of the two unknown compounds of *m/z* 317.102 [M + H]^+^, F2H2 and FOMT2 were incubated together in the presence of naringenin, NADPH, and SAM. In addition to

2-hydroxynaringenin, two pairs of peaks were detected consistent with the keto and enol tautomers of mono-*O*-methylated 2-hydroxynaringenin (*m*/*z* 303.086) and di-*O*-methylated 2-hydroxynaringenin (*m*/*z* 317.102), respectively (Figure  4D). In order to verify the structure of the *m/z* 317.102 [M + H]^+^ compounds, we purified them from a *FOMT2-*overexpressing *E. coli* culture incubated with chemically synthesized 2-hydroxynaringenin as substrate. NMR analyses confirmed the dominant FOMT2 products as *O*-dimethylated 2-hydroxynaringenins, which occur in both the keto and enol forms in a 1:2 ratio, at room temperature (Supplemental Figure S13; Supplemental Data Set S2). UV measurements confirmed predictions that the two tautomer peaks have different UV absorption maxima at 283 nm for the first peak (3.1; RT = 5.51 min in Figure  4D) and 352 nm for the second peak (3.2; RT = 6.81 min in Figure  4D;Supplemental Figure S14). Given that the conjugated enol system usually absorbs at longer wavelengths than the diketone system, we propose that the first peak (3.1 in Figure  4D) corresponds to the keto tautomer, while the second peak (3.2 in Figure  4D) corresponds to the enol tautomer (Figure  4E). As *O*-dimethylated 2-hydroxynaringenin appears to be an undescribed compound, we have named it xilonenin in reference to the Aztec maize goddess Xilonen. Our data thus reveal the fungus-elicited production of two di-*O*-methylated 2-hydroxynaringenin tautomers that are derived from the sequential activity of a F2H (F2H2), to produce 2-hydroxynaringenin, and FOMT2. Importantly, the free rotation of the A-ring in the chalcone-like open-ring form of 2-hydroxynaringenin allows FOMT2 to catalyze two sequential *O*-methylation reactions on the hydroxyl groups in *ortho*-position of ring A (Figure  4E).

### Flavonoids accumulate locally at the site of pathogen infection

**Figure 5:**
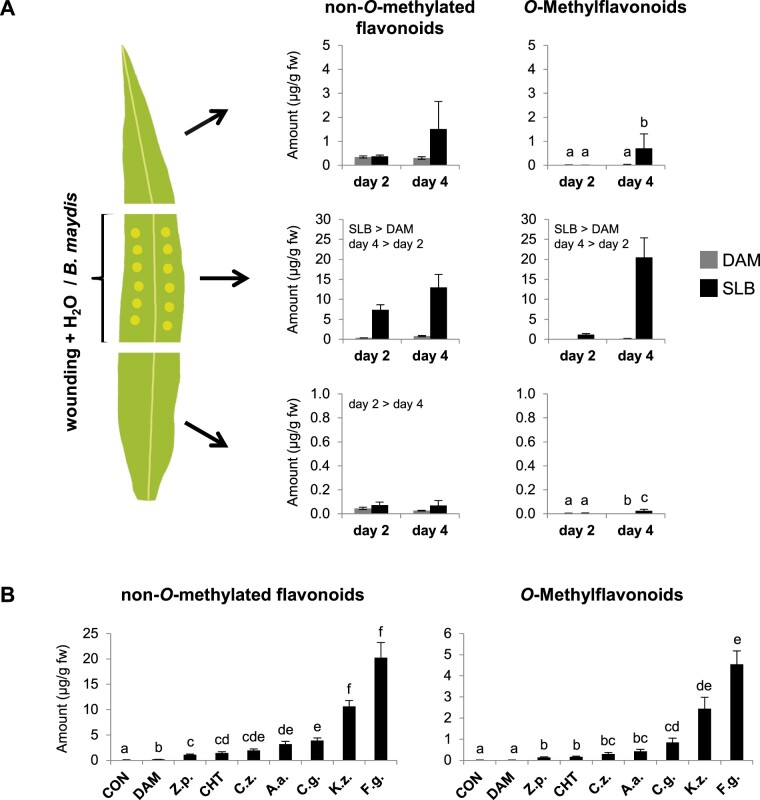
The accumulation of flavonoids is a general pathogen response in maize and occurs locally at the site of pathogen infection. A, Spatial distribution of non-*O*-methylated and *O*-methylated flavonoids in “upper,” “middle,” and “lower” (top down) segments of leaves of the inbred line B75. The middle leaf segment was mechanically damaged and either treated with water as control (DAM) or a mycelial suspension of *B. maydis* (SLB) for 2 or 4 d. Compounds were quantified in the three leaf parts using LC–MS/MS. Shown are the total amounts of all analyzed non-*O*-methylated and *O*-methylated flavonoids (left and right parts, respectively; Means ± se; *n* = 6). Significant differences for the factors treatment or day are stated. Different letters indicate significant differences between treatments and days (for statistical values, see Supplemental Table S9). Results for individual analytes are given in Supplemental Tables S7 and S8. B, Concentrations of non-*O*-methylated flavonoids (left) and *O*-methylflavonoids (right) in leaves of hybrid maize (“Sweet Nugget”) 4 d after wounding and treatment with different fungal pathogens and CHT. Controls included undamaged (CON) as well as damaged and water-treated (DAM) leaves. Shown are the total amounts of all analyzed non-*O*-methylated and *O*-methylated flavonoids (means ± se; *n* = 8). Different letters indicate significant differences (*P* < 0.05) between treatments (one-way ANOVA followed by Tukey’s honestly significant difference (HSD) *post hoc* test; non-*O*-methylated flavonoids (*F* = 198.700, *P *< 0.001); *O*-methylflavonoids (*F* = 113.500, *P* < 0.001)). Results for individual analytes are given in Supplemental Table S10). CHT, chitosan; *Z.p*., *Z. pseudotritici*; *C.z*., *C. zeae-maydis*; *A.a.*, *A. alternata*; *C.g.*, *C. graminicola*; *K.z.*, *K. zeae*; *F.g.*, *F. graminearum*.

A defining feature of phytoalexins is their rapid and local accumulation at pathogen infection sites (Nicholson and Hammerschmidt, 1992; Hammerschmidt, 1999). To investigate the spatial distribution of fungal-induced flavonoids in maize leaves, we wounded and inoculated leaves of the inbred line B75 and hybrid maize “Sweet Nugget” in a defined leaf area with *B. maydis* (SLB) hyphae and quantified non-*O*-methylated and *O*-methylated flavonoids in three different leaf segments of which only the middle segment was SLB-infected (Supplemental Tables S7 and S8). The infected middle leaf segments of B75 accumulated much larger amounts of non-*O*-methyl and *O*-methylflavonoids than the noninfected upper and lower leaf segments (Figure  5A;Supplemental Table S9). Induced accumulation was already significant 2 d post-inoculation, but was further increased at day 4. Similar results were obtained for the hybrid maize “Sweet Nugget” (Supplemental Figure S15; Supplemental Table S9).

### The induction of flavonoids is a general pathogen response

To test whether the production of maize flavonoids is elicited by diverse fungal pathogens and thus represents a common defense response, we analyzed leaves (*Z. mays* “Sweet Nugget” hybrid) treated with six different maize fungal pathogens, including necrotrophs and hemibiotrophs, and the elicitor chitosan (CHT; Supplemental Table S10). Despite remarkable quantitative differences in flavonoid content for the different fungal treatments, which are in line with the manifestation of disease symptoms (Supplemental Figure S16), all of the fungi as well as CHT significantly induced the production of both *O*-methylated and non-*O*-methylated flavonoids (Figure  5B;Supplemental Table S10). Overall non-*O*-methyl and *O*-methylflavonoid content and composition were consistent with our previous data obtained for this maize line (Supplemental Figure S15; Supplemental Tables S7 and S8). These results demonstrate that the production of flavonoids, especially *O*-methylflavonoids is part of a general maize response to fungal pathogens.

### The fungus-induced formation of *O*-methylflavonoids is accompanied by large-scale transcriptomic and metabolomic changes in the flavonoid and BX pathways

**Figure 6:**
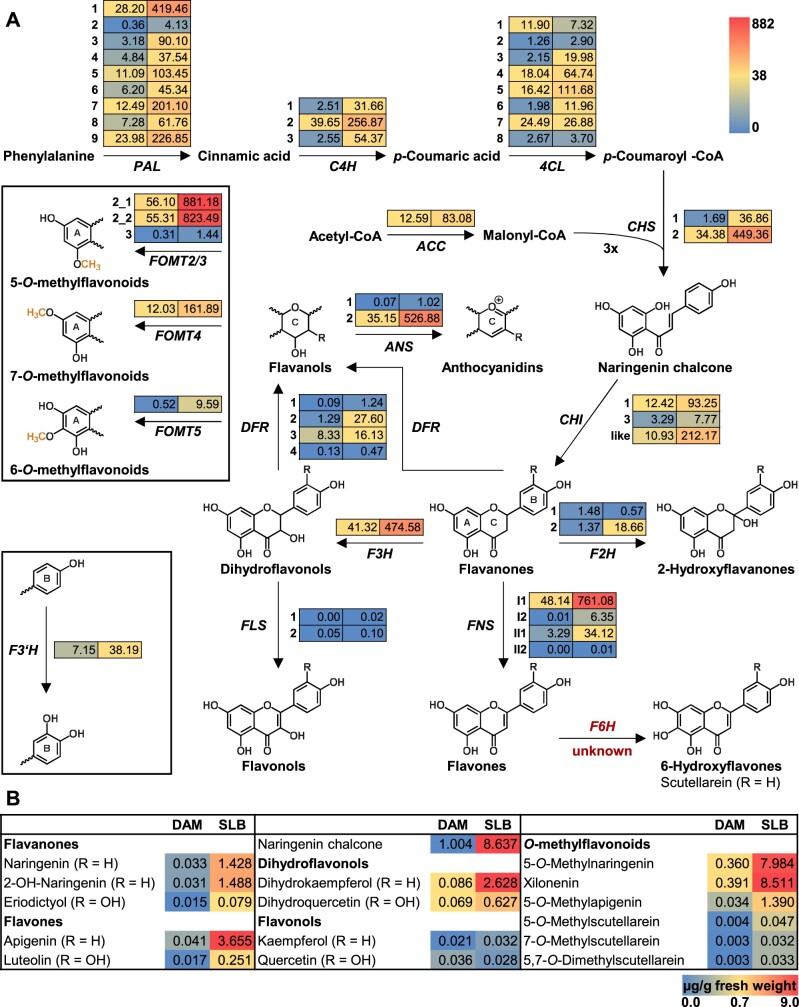
Upregulation of the flavonoid biosynthetic pathway by fungal infection. A, Expression of genes putatively involved in the flavonoid biosynthetic pathway in damaged and water-treated control leaves (DAM) or in damaged and *B. maydis*-infected leaves (SLB) of W22 after 4 d of treatment. Transcriptomes were sequenced and mapped to the *Z. mays* W22 NRGene V2 genome. RPKM values (means; *n* = 4) for each gene are shown as a heat map next to the gene abbreviation: DAM (left column) and SLB (right column). For statistics, corresponding gene abbreviations and gene IDs see Supplemental Table S2. B, Quantitative LC–MS/MS analysis of representative flavonoids in the same samples. Metabolite amounts are given in microgram per gram fresh weight for DAM (left column) and SLB (right column). RPKM, reads per kilobase per million reads mapped.

A broader investigation of transcriptomic and metabolomic data sets from SLB-infected and noninfected W22 leaves revealed many differences between the treatments beyond the *O*-methylation of flavonoids and their accumulation (Supplemental Figure S17). Apart from *FOMT2/3*, *FOMT4*, and *FOMT5*, a majority of known or predicted gene transcripts associated with flavonoid pathways increased significantly in response to the fungal elicitation (Figure  6A;Supplemental Table S2). Transcript abundance was associated with increased production of flavonoids belonging to different subclasses, mainly flavanones, flavones, and dihydroflavonols (Figure  6B;Supplemental Tables S7 and S8). In the BX pathway, transcript changes were more diverse. While genes encoding the core pathway (*BX1*-*BX8*) were downregulated after fungal infection, the terminal steps catalyzed by the OMTs BX10/11/14 were upregulated at both the transcript and metabolite levels (Supplemental Figure S18; Supplemental Tables S2 and S11). A RT-qPCR analysis of selected flavonoid and BX pathway genes confirmed the broad transcriptomic data, which was obtained from the RNA-seq experiment (Supplemental Figure S19).

### Xilonenin and other maize flavonoids have antifungal activity

**Figure 7:**
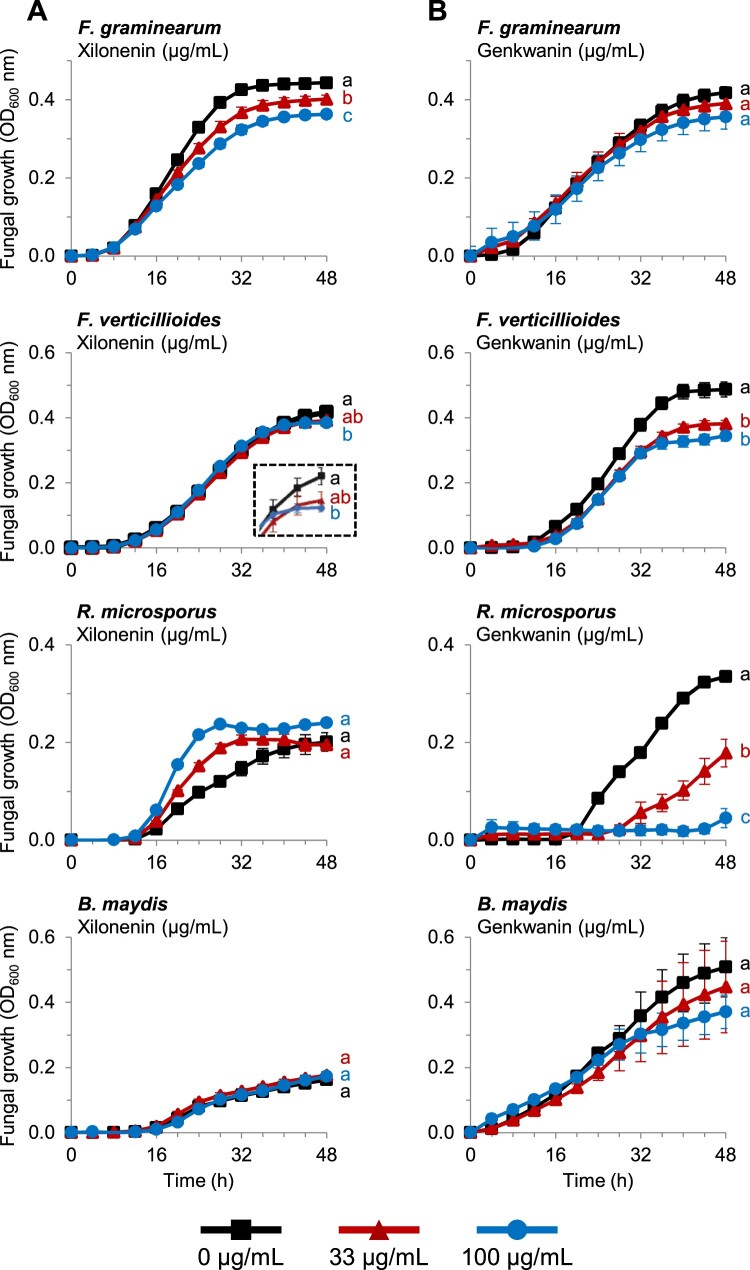
Antifungal activity of xilonenin and genkwanin. Growth (optical density (OD) at 600 nm) of *F. graminearum*, *F. verticillioides*, *R. microsporus*, and *B. maydis* in the absence and presence of purified xilonenin (A) and genkwanin (B) measured over a 48-h time course in a defined minimal broth medium using a microtiter plate assay. Data are shown as means ± se (*n* = 4–5). Different letters indicate significant differences (*P* < 0.05) between treatments at 48 h (one-way ANOVA followed by Tukey–Kramer’s *post hoc* test). *Fusarium graminearum*: xilonenin (*F* = 20.359, *P* < 0.001); genkwanin (*F* = 1.669, *P* = 0.242); *F. verticillioides*: xilonenin (*F* = 4.710, *P* = 0.031); genkwanin (*F* = 19.373, *P* < 0.001); *R. microsporus*: xilonenin (*F* = 3.386, *P *= 0.068); genkwanin (*F* = 47.766, *P* < 0.001); *B. maydis*: xilonenin (*F* = 0.485, *P* = 0.627); genkwanin (*F* = 0.460, *P* = 0.645).

Xilonenin was the most abundant FOMT product detected in our experiments (Figure  1;Supplemental Table S8). To examine its impact on the growth of specific maize fungal pathogens, we conducted in vitro bioassays using *F. graminearum*, *F. verticillioides*, *Rhizopus microsporus*, and *B. maydis*, responsible for disease in diverse tissues. After 48 h, xilonenin significantly reduced the growth of *F. graminearum* in a dose-dependent manner (Figure  7). A similar but less pronounced growth inhibition activity was observed against *F. verticillioides* at a concentration of 100 µg/mL. In contrast, xilonenin showed no antifungal activity against *R. microsporus* or *B. maydis* but rather trended toward growth promotion; however, this effect was not statistically significant at 48 h (Figure  7).

Genkwanin, another *O*-methylflavonoid highly abundant in fungus-infected maize, negatively affected the growth of *F. verticillioides* but not *F. graminearum* (Figure  7). However, this compound showed strong dose-dependent activity against *R. microsporus*, while growth of *B. maydis* was slightly, but not significantly, reduced (Figure  7).

Interestingly, the non-*O*-methylated flavonoid naringenin also reduced the growth of all tested fungi, while its 5-*O*-methyl derivative showed no statistical effects at 48 h (Supplemental Figure S20). Apigenin slightly inhibited the growth of *R. microsporus*, and 5-*O*-methylapigenin reduced the growth of both *F. verticillioides* and *R. microsporus* (Supplemental Figure S21). In contrast, apigenin and 5-*O*-methylapigenin did not result in statistically significant differences in the growth *F. graminearum* and *B. maydis* (Supplemental Figure S21).

## Discussion

Previous research has implicated *O*-methylflavonoids in grass species as anti-pathogen defenses (Kodama et al., 1992; Christensen et al., 1998; Zhou et al., 2006a; Hasegawa et al., 2014). In maize, infection studies with *Colletotrichum graminicola* first hinted that *O*-methylflavonoid pathways might play a role in maize–pathogen interactions (Balmer et al., 2013). However, the enzymes underlying the relevant biosynthetic pathways have remained unknown. In this effort, we undertook a comprehensive analysis of fungal-elicited maize *O*-methylflavonoids and pathway enzymes, resulting in the characterization of a CYP F2H and multiple OMTs with distinct product regiospecificity that produce the major inducible products. Moreover, we showed significant in vitro antifungal activity for the most abundant product, the *O*-dimethyl-2-hydroxynaringenin tautomer xilonenin, and for additional abundant *O*-methylated and non-*O*-methylated flavonoids.

### Association studies and enzyme analyses demonstrate that FOMT2 and FOMT4 are responsible for the formation of maize *O*-methylflavonoids

FOMTs have been characterized from dicot and a few monocot species (Kim et al., 2010); however, only two FOMTs active on the flavonoid A-ring have been reported in grasses (Christensen et al., 1998; Shimizu et al., 2012). Here, we identified and characterized four maize *OMT* genes, namely *FOMT2*, *FOMT3*, *FOMT4*, and *FOMT5* that were able to convert different flavonoids regiospecifically to their respective 5-, 7-, and 6-*O*-methyl derivatives (Figures  2 and 3; Supplemental Table S5). Several lines of evidence suggest that two of these OMTs, FOMT2 and FOMT4, are responsible for the formation of the bulk of the *O*-methylflavonoids detected in planta. First, metabolite-based association mapping efforts identified *FOMT2* and *FOMT4* as key biosynthetic candidates (Figure  2, A and B; Supplemental Figures S2 and S3). Second, transcripts of *FOMT2* and *FOMT4* and their corresponding enzymatic products (5- and 7-*O*-methylflavonoids, respectively) accumulated significantly after fungal elicitation, while *FOMT3* encoding another 5-OMT displayed low levels of expression (Figures  1, 2C, and 6; Supplemental Table S2). Third, biochemical characterization not only confirmed the regiospecific activity of the FOMTs, but further demonstrated that FOMT2 and FOMT4 prefer flavanones and flavones, respectively, as substrates, mirroring the qualitative and quantitative abundance of the corresponding 5- and 7-*O*-methylflavonoids in planta (Figures  2E and 3; Supplemental Figure S8; Supplemental Table S8).

To understand defense pathway specificity, we also examined the BX pathway OMTs BX10, BX11, BX12, and BX14 that are closely related to FOMT2/3 (Figure  2D). All four BX OMTs displayed only trace activities for specific subsets of the tested flavonoid substrates, with 5- and/or 7-*O*-methyl derivatives produced in unspecific amounts (Supplemental Table S5). However, BX10, BX11, and BX12 each catalyzed the 5,7-*O*-dimethylation of apigenin (Supplemental Table S5), at a rate up to 60% of that of the FOMT2 and FOMT4 combination, demonstrating that BX OMTs could contribute to the biosynthesis of specific *O*-methylflavonoids in a limited way.

### F2H2 and FOMT2 are the key enzymes in the biosynthesis of xilonenin tautomers

Previously, F2H1 (CYP93G5) was demonstrated to catalyze the conversion of flavanones (naringenin and eriodictyol) to their corresponding 2-hydroxy derivatives, which are intermediates in the production of maize *C*-glycosyl flavone anti-herbivore defenses such as maysin (Morohashi et al., 2012; Falcone-Ferreyra et al., 2013; Casas et al., 2016). Our results demonstrate that the homologous enzyme F2H2 (CYP93G15) together with FOMT2 is involved in fungus-elicited production of the tautomeric xilonenin (Figure  4). F2H2 catalyzes the same reaction as F2H1 in vitro, converting naringenin and eriodictyol to 2-hydroxynaringenin and 2-hydroxyeriodictyol, respectively (Figure  4D;Supplemental Figure S12); however, only *F2H2* expression occurs upon fungal elicitation (Figure  4C;Supplemental Figure S19). Closely related to the F2Hs are the FNSIIs (Figure  4B), which are proposed to generate the flavone double bond via a reaction where initial hydrogen abstraction from C-2 is followed by hydroxylation at this position and finally dehydration between C-2 and C-3. F2H activity is similar but with the loss of the dehydratase activity (Akashi et al., 1998; Sawada et al., 2002). The close similarity between these two enzyme groups was seen in our work with FNSII2, a close relative to the recently characterized FNSII1 (CYP93G7; Righini et al., 2019). FNSII2 produced apigenin and low yet detectable levels of 2-hydroxynaringenin (Supplemental Figure S12), supporting 2-hydroxyflavanones as intermediates in the FNSII reaction mechanism. However, whether 2-hydroxyflavanones are accepted as substrates by FNSII1/2 remains to be elucidated.

Association mapping analyses using the B73 × Ky21 RIL population and the Goodman diversity panel linked *FOMT2* with the occurrence of 2-hydroxynaringenin-derived xilonenin tautomers (Figure  4A;Supplemental Figures S2 and S10). Heterologous enzyme expression assays confirmed that recombinant FOMT2 utilizes 2-hydroxynaringenin as a substrate to catalyze the production of xilonenin in vitro (Figure  4, D and E). Furthermore, the extensive list of substrates used for comprehensive biochemical characterization demonstrates that 2-hydroxynaringenin is the preferred substrate of FOMT2 (Figure  3). Our results parallel the identification of xilonenin as most abundant FOMT2 product in both W22 and B75 inbred lines (Supplemental Table S8). Besides xilonenin tautomers, we detected two other products of FOMT2 in the enzyme assays and in the plant that are likely keto-enol tautomers of *O*-methyl-2-hydroxynaringenin (Figure  4D;Supplemental Figure S9). However, the rather low abundance of the precursor 2-hydroxynaringenin and its mono-*O*-methyl derivatives compared to xilonenin tautomers indicate a rapid and efficient turnover by FOMT2 in planta (Figure  1;Supplemental Tables S7 and S8). The presence of the first methoxyl group in *O*-methyl-2-hydroxynaringenin does not seem to influence the occurrence of the second *O*-methylation reaction considerably. This is in contrast to other mono-*O*-methylflavonoids such as sakuranetin, genkwanin, acacetin, or hispidulin that are only marginally accepted by FOMT2 as substrates for a second *O*-methylation (Figure  3).

Although xilonenin has apparently not been previously described, chalcone-like *O*-methylflavonoids are known and proposed as intermediates in the biosynthesis of echinatin, a retrochalcone with antibacterial activity from licorice (*Glycyrrhiza spp.*; Ayabe et al., 1980; Haraguchi et al., 1998).

### Maize *O*-methylflavonoids are induced by fungal infection and contribute to plant defense

Flavonoids, including *O*-methylflavonoids have been previously shown to increase pathogen resistance in several plant species (Kodama et al., 1992; Skadhauge et al., 1997; Hasegawa et al., 2014). The complex flavonoid blends we measured in maize upon fungal attack (Figure  1;Supplemental Tables S7 and S8) accumulated largely at the sites of pathogen infection (Figure  5A;Supplemental Figure S15), consistent with the response of other phytoalexins (Nicholson and Hammerschmidt, 1992; Hammerschmidt, 1999). Moreover, several of the *O*-methylflavonoids detected in fungus-elicited maize, such as genkwanin or 7-*O*-methylscutellarein (Figure  1;Supplemental Table S8), have previously been shown to possess antimicrobial activity (Martini et al., 2004; Balmer et al., 2013; Zhanzhaxina et al., 2020), suggesting that the maize flavonoid blend contributes to plant defense against pathogens. Interestingly, xilonenin, the most prominent FOMT product in the investigated maize lines (Figure  1;Supplemental Table S8), and other abundant *O*-methylated and non-*O*-methylated flavonoids exhibited contrasting effects on the growth of different maize pathogenic fungi in our experiments. While xilonenin had significant antifungal activity against two *Fusarium* species but did not inhibit the growth of *B. maydis* and *R. microsporus*, genkwanin affected the growth of *R. microsporus* and *F. verticillioides* but not *F. graminearum* and *B. maydis* (Figure  7). This suggests that the complex flavonoid blend comprising more than 35 different compounds may provide a defense barrier against a multitude of diverse maize pathogens. Moreover, additive and synergistic effects might mediate or even enhance the activity of single blend components. However, the mixed antifungal properties observed in our bioassays may also indicate that the maize pathogen defense response relies on several biochemical layers. For example, flavonoids may not be the predominant antifungal compounds, but may induce signaling pathways that trigger the formation of other antifungal defenses by, for example, acting as scavengers for reactive oxygen species (Zhang et al., 2015). On the other hand, some maize pathogens may have adapted to the toxic arsenal of their host plant by detoxifying their phytoalexins as is the case for other plant pathogens (Pedras and Ahiahonu, 2005), and this might explain the mixed antifungal effects seen in our bioassays. Recently, two rice pathogenic fungi have been reported to detoxify and tolerate 7-methoxynaringenin (sakuranetin) by hydroxylation, *O*-demethylation or glycosylation (Katsumata et al., 2017, 2018). Maize may still respond to fungal attack with the accumulation of flavonoid phytoalexins even if these are not effective since we demonstrated that flavonoid induction occurs in response to a broad range of necrotrophic and hemibiotrophic pathogens (Figure  5B).

Maize has been previously reported to biosynthesize complex mixtures of other pathogen-induced defense compounds including BXs, sesquiterpenoids, and diterpenoids (Oikawa et al., 2004; Rostas, 2007; Ahmad et al., 2011; Huffaker et al., 2011; Mafu et al., 2018; Ding et al., 2019, 2020). These substances have been demonstrated to reduce fungal diseases in experiments with defined biosynthetic mutants of the BX, kauralexin, and zealexin pathways (Ahmad et al., 2011; Ding et al., 2019, 2020). Here we highlight the role of another class of fungal-induced metabolites, the *O*-methylflavonoids, in innate immune responses that likely contribute to pathogen resistance in maize. Further investigation is required to understand if these different groups of phytoalexins have separate or joint roles in maize defense.

## Materials and methods

### Plants and growth conditions

Seeds of maize (*Z.* *mays*) inbred line W22 (NSL 30053), B73 (PI 550473), B75 (PI 608774), and Nested association mapping (NAM; McMullen et al., 2009) parental line seeds were provided by the US Department of Agriculture, Agricultural Research Service (USDA-ARS). Maize seeds for the Goodman diversity panel (Flint-Garcia et al., 2005) and the NAM RILs B73 × Ky21 subpopulation (McMullen et al., 2009) were provided by G. Jander (Boyce Thompson Institute) and P. Balint-Kurti (USDA-ARS), respectively. Seeds of the maize hybrid “Sweet Nugget” were purchased from N.L. Chrestensen Samen- und Pflanzenzucht GmbH (Erfurt, Germany). Plants were potted in soil (mix of 70 L Tonsubstrat with 200 L Kultursubstrat TS 1, Klasmann-Deilmann, Geeste, Germany) and grown in a climate-controlled chamber (Snijders Labs, Tilburg, Netherlands) under a 16-h light/8-h dark photoperiod, 1 mmol m^−2^s^−1^ photosynthetically active radiation, a temperature cycle of 24°C/20°C (day/night), and 70% relative humidity.

### Fungi and growth conditions

Fungal cultures of *B.* *maydis* (Belgian Co-ordinated Collections of Micro-Organisms, Institute of Hygiene, Epidemiology and Mycology, strain no. 5881), *C.* *graminicola* (Leibniz-Institut, Deutsche Sammlung von Mikroorganismen und Zellkulturen GmbH (DSMZ), strain (DSM) no. 63127), *F.* *graminearum* (DSM 4528), and *Kabatiella zeae* (DSM 62737) were grown on potato dextrose agar (Sigma-Aldrich, St Louis, MO, USA) for 7 d at 25°C or 28°C (*B. maydis*) in the dark prior to use and subcultured if necessary (see below) to induce sporulation. *Alternaria alternata* (DSM 62006) and *Cercospora zeae-maydis* (Westerdijk Fungal Biodiversity Institute, strain no. 117755) were grown on modified V8 agar (V8 replaced by tomato juice, pH 6.5) for 7 and 14 d, respectively, at 25°C in the dark. To obtain mycelial inoculum, sterile water was added to an agar plate; the mycelium gently scraped off, and homogenized using a tissue homogenizer (Potter-Elvehjem, Carl Roth, Karlsruhe, Germany). Sporulation of *C. graminicola* was induced by subculturing on oatmeal agar (Sigma-Aldrich) at 25°C in the dark for 5–7 d. *Kabatiella* *zeae* sporulation was enhanced using liquid *K. zeae* medium (KZM; Reifschneider and Arny, 1979). Briefly, 50 mL KZM were inoculated with a colony plug and incubated at 25°C and 150 rpm for 4 d. Afterwards, 400 µL of the liquid culture were plated on corn meal agar (Sigma-Aldrich) and grown for another 4 d. To promote sporulation of *C. zeae-maydis*, the mycelium (∼2 cm^2^) was cut in little pieces, suspended in 10 mL sterile water, mixed vigorously and pipetted on V8 agar (2 mL/plate). After 15 min, remaining liquid was decanted and the plate was incubated at room temperature and 12-h d light for 5 d. Spores of *C. zeae-maydis* could not be separated from the mycelial fragments and hence a mixed spore and mycelial inoculum was used for experiments. All other spores were harvested in sterile water, filtered through a 40-µm cell strainer and quantified for use. *Zymoseptoria pseudotritici* (STIR04 2.2.1) was kindly provided by Eva Stukenbrock (Stukenbrock et al., 2011, 2012) and grown on yeast-malt agar (4 g/L yeast extract, 4 g/L malt extract, 4 g/L sucrose, 15 g/L agar) at 18°C in the dark for 7 d. Then, colonies were picked, used to inoculate liquid yeast-malt sucrose (4 g/L yeast extract, 4 g/L malt extract, 4 g/L sucrose), and incubated at 18°C and 150 rpm for 4–5 d. Spores were harvested by centrifugation and resuspended in sterile water for quantification.

### Plant inoculations with live fungi and CHT

All experiments were performed on the third fully developed leaf of 14-d-old maize plants. To analyze the content and spatial distribution of flavonoids in different maize lines after *B. maydis* infection, the middle segments of leaves were wounded on both sides of the midrib using modified pliers (punch-inoculation method; Matsuyama and Rich, 1974), producing a crushed spot, but without punching out a hole. Usually, 12 crushed spots per middle segment of about 10-cm length were made. Afterwards, a mycelial suspension of *B. maydis* containing 0.02% (v/v) Tween-20 was applied with a sterile cotton swab to each wounded spot (*n* = 6–8). Control plants were wounded and treated with water containing 0.02% (v/v) Tween-20 (*n* = 6–8). Whole plants were wrapped in plastic oven bags (“Bratschlauch,” Toppits, Minden, Germany) left open at the top to allow moderate air circulation, but prevent direct contact between plants of different treatments, and incubated for two or 4 d. For the general pathogen response experiment, hybrid maize (var. “Sweet Nugget”) plants were treated as described above, except that *C. graminicola*, *K. zeae*, and *Z. pseudotritici* were used as spore suspensions (1 × 10^6^/mL), while all other fungi were applied as a mycelial suspension. In addition, control treatments included undamaged plants. CHT was used as an artificial elicitor. Therefore low viscous CHT (50–190 kDa; Sigma-Aldrich) was dissolved to 1% (w/v) in 1% (v/v) acetic acid in water and further diluted with sterile water to 0.1% (w/v). Control plants were treated with 0.1% (v/v) acetic acid in water, respectively. In all experiments, different leaf segments were collected separately by cutting the leaf on both sides of the wounded and inoculated area (1.5 cm distant from the outer spots), flash-freezing in liquid nitrogen (N_2_), and storing at −80°C until further processing.

### Maize stem treatments with heat-killed fungal elicitors

Treatment of NAM inbred line parents and plants of the Goodman association panel follow from previous efforts (Ding et al., 2017, 2019). Plants of the Goodman diversity panel (260 analyzed inbred lines) were grown in greenhouses while the NAM RIL B73 × Ky21 subpopulation (156 analyzed lines) was grown in the field (2016, UCSD). Using a scalpel, 35-d-old plants were slit in the center, spanning both sides of the stem, to create an 8-cm long parallel longitudinal incision spanning the upper nodes, internodes, and basal portion of unexpanded leaves. To activate antifungal defenses, 500 μL of the heat-killed fungal hyphae (commercial *Fusarium venenatum*, strain PTA-2684, Monde Nissin Corporation, Santa Rosa, Phillipines) was placed into each slit stem and sealed with clear plastic packing tape to minimize tissue desiccation. Three or 5 d after elicitation (for plants of the Goodman panel and B73 × Ky21 RILs, respectively), reacted stem tissues were harvested in liquid N_2_, ground to fine power, weighed out in 50 mg aliquots and stored at −80°C for analyses.

### Methanol extraction of plant material

Maize leaf tissue was ground to a fine powder under liquid N_2_ using a Geno/Grinder tissue homogenizer (SPEX SamplePrep). The frozen powder (50–70 mg) was weighed in a 2 mL microcentrifuge tube, and five volumes of 100% methanol (LC–MS grade, Merck) were added. The plant samples were immediately vortexed, and then further extracted using a ThermoMixer C (Eppendorf, Hamburg, Germany) for 5 min at 2,000 rpm and 20°C. Cell debris was sedimented by centrifugation at 16,000 *g* and 20°C for 25 min and the supernatant was transferred to a new 1.5-mL microcentrifuge tube. The sediment was extracted a second time with five volumes of 100% methanol and finally the combined supernatant was centrifuged again to remove all remaining particles. All samples were stored at −20°C before analysis.

### LC–MS analysis of flavonoids and BXs

#### Untargeted LC–MS analysis with accurate mass determination

Chromatography was performed on a Dionex UltiMate 3000 RS pump system (Thermo Fisher Scientific, Waltham, MA, USA) equipped with a ZORBAX RRHD Eclipse XDB-C18 column (2.1 × 100 mm, 1.8 µm; Agilent Technologies, Santa Clara, CA, USA). Aqueous formic acid (0.1% (v/v)) and acetonitrile were used as mobile phases A and B, respectively, with a flow rate of 0.3 mL/min. The column temperature was maintained at 25°C. The following elution profile was used: 0–0.5 min, 5% B; 0.5–11 min, 5–60% B; 11.1–12 min, 100% B; 12.1–15 min, 5% B. The injection volume was 2 µL. The LC system was coupled to a timsTOF mass spectrometer (Bruker Daltonics, Billerica, MA, USA) equipped with an ESI ion source. Both positive and negative ionization were used for the analysis in full scan and auto MS/MS modes, scanning masses from *m/z* 50–1,500 (detailed parameters are provided in Supplemental Table S12). Sodium formate adducts were used for internal calibration. The software programs Bruker otof control version 5.1.107 and HyStar 4.1.31.1 (Bruker Daltonics) were used for data acquisition, and DataAnalysis version 5.1.201 (Bruker Daltonics) and MetaboScape version 4.0 (Bruker Daltonics) were used for data processing.

#### Targeted LC–MS/MS analysis for quantification of compounds in plant extracts and analysis of enzyme assays

Chromatographic separation was achieved on an Agilent 1260 Infinity II LC system (Agilent Technologies) equipped with a ZORBAX Eclipse XDB-C18 column (50 × 4.6 mm, 1.8 μm; Agilent Technologies), using aqueous formic acid (0.05% (v/v)) and acetonitrile as mobile phases A and B, respectively. The flow rate was 1.1 mL/min and the column temperature was maintained at 20°C. The injection volume was 2 µL for maize leave extracts and 1–4 µL for enzyme assays. The following gradient was used for the separation of flavonoids and flavonoid glycosides: 0–0.5 min, 10% B; 0.5–8.0 min, 10–55% B; 8.5–9.0 min, 100% B; 9.02–11 min, 10% B. The LC system was coupled to a QTRAP 6500+ tandem mass spectrometer (Sciex, Framingham, MA, USA) equipped with a turbospray ESI ion source, operated in positive or negative ionization mode, for the analysis of flavonoids or flavonoid glycosides, respectively (detailed parameters are provided in Supplemental Table S13). For the analysis of BXs, the chromatography was performed as described above, except that the following elution profile was used: 0–0.5 min, 5% B; 0.5–6.0 min, 5–32.5% B; 6.02–7.0 min, 100% B; 7.10–9.5 min, 5% B. The mass spectrometer was operated in negative ionization mode (detailed settings are provided in Supplemental Table S13). Multiple reaction monitoring was used to monitor analyte precursor ion → product ion transitions of flavonoids, flavonoid glycosides and BXs (Supplemental Tables S4, S15, and S16, respectively). Flavonoids were quantified using external calibration curves (0.5, 1, 2, 5, 10, 25, 50, 100, 200, 400, 1,000, 2,000, and 4,000 ng/mL) composed of commercially available standards as well as self-purified and NMR-quantified *O*-methylflavonoids (for all standards used, see Supplemental Table S17). Analyst version 1.6.3 software (Sciex) was used for data acquisition and processing. In addition, MultiQuant version 3.0.3 software (Sciex) was used for quantitative analysis.

#### Untargeted LC–UV–MS analysis for purification

The *O*-methylflavonoid content of *E. coli* culture extracts was analyzed using an Agilent 1100 Series LC system (Agilent Technologies) coupled to an ultraviolet diode array detector (UV-DAD, Agilent Technologies) and an Esquire 6000 ESI-Ion trap mass spectrometer (Bruker Daltonics). Chromatographic separation was performed on an EC 250/4.6 Nucleodur Sphinx column (RP 5 μm, Macherey-Nagel, Düren, Germany), with 0.2% (v/v) formic acid in water (A) and acetonitrile (B) as mobile phases. The flow rate was 1 mL/min and the column temperature was set to 25°C. The following elution profile was used: 0–15 min, 30–60% B; 15.1–16 min, 100% B; 16.1–20 min, 30% B. The mass spectrometer was run in alternating ion polarity (positive/negative) mode with a skimmer voltage of +40 V/−40 V, a capillary voltage of −3,500 V/+3,000 V and a capillary exit voltage of 113.5 V/−113.5 V, to scan masses from *m/z* 50–3,000. N_2_ was used as drying gas (11 L/min, 330°C) and nebulizer gas (35 psi). The software programs esquireControl version 6.1 (Bruker Daltonics) and HyStar version 3.2 (Bruker Daltonics) were used for data acquisition, while DataAnalysis version 3.4 was used for data processing. The UV absorption of individual *O*-methylflavonoids was analyzed using the post-processing software included in the HyStar version 3.2 package (Bruker Daltonics).

#### Semi-preparative high performance liquid chromatography with ultraviolet detector(HPLC-UV) for purification

For the purification of *O*-methylflavonoids, an Agilent 1100 series LC system (Agilent Technologies) coupled to an UV/VIS-detector and connected to an SF-2120 Super Fraction Collector (Advantec MSF, Inc., Dublin, CA, USA), was used. Chromatography was performed as described above in the section “Untargeted LC–MS analysis coupled with UV for purification,” except that 0.05% (v/v) formic acid in water was used as mobile phase A. Depending on the *O*-methylflavonoid to be purified, UV absorption was monitored at a single wavelength between 280 and 335 nm and used to determine the respective peak(s) for collection. HP ChemStation for LC (Rev. A.06.03, Hewlett Packard) was used for data acquisition.

### Analyses of fungus-elicited flavonoids in stems

Analysis of fungus-elicited tissue from the NAM RIL B73 × Ky21 subpopulation and Goodman diversity panel employed LC/MS parameters and settings previously described (Ding et al., 2017). Stem tissue samples were sequentially bead homogenized in a series of solvents resulting in final volume of 450 μL and mixture of 1-propanol:acetonitrile:ethyl acetate:water (11:39:28:22). Approximately 150 μL of the particulate-free supernatant was used for LC/MS analyses using 5-μL injections. The LC consisted of an Agilent 1260 Infinitely Series HP Degasser (G4225A), 1260 binary pump (G1312B), and 1260 autosampler (G1329B). The binary gradient mobile phase consisted of 0.1% (v/v) formic acid in water (solvent A) and 0.1% (v/v) formic acid in methanol (solvent B). Chromatographic separation was performed on a Zorbax Eclipse Plus C18 Rapid Resolution HD column (Agilent; 1.8 μm, 50 × 2.1 mm) using a 0.35 mL/min flow rate. The mobile phase gradient was: 0–2 min, 5% B constant ratio; 3 min, 24% B; 18 min, 98% B; 25 min, 98% B; and 26 min, 5% B for column re-equilibration before the next injection. Electrospray ionization was accomplished with an Agilent Jet Stream Source with the following parameters: nozzle voltage (500 V), N_2_ nebulizing gas (flow, 12 L/min, 379 kPa, 225°C) and sheath gas (350°C, 12 L/min). The transfer inlet capillary was 3,500 V and both MS1 and MS2 heaters were at 100°C. Negative ionization [M-H]^−^ mode scans (0.1-atomic mass unit steps, 2.25 cycles/s) from *m/z* 100–1,000 were acquired. The compounds identified in order of relative retention times and [M-H]^−^ parent ions are: xilonenin keto tautomer (9.00 min, *m*/*z* 315), apigenin-5-methyl ether (10.37 min, *m/z* 283), xilonenin enol tautomer (10.71 min, *m*/*z* 315), apigenin (11.78 min, *m*/*z* 269), and genkwanin (13.77 min, *m*/*z* 283).

### Genetic mapping of *O*-methylflavonoid biosynthetic genes

A list of Goodman diversity panel inbred lines and NAM B73 × Ky21 subpopulation RILs used for mapping in this study is given in Supplemental Table S18. Flavonoid levels were used as traits for the association analyses. Genotypic data for the NAM B73 × Ky21 RIL subpopulation (NAM imputed AllZea GBS Build July 2012 FINAL, AGPv2) and Goodman Diversity panel (Maize HapMapV3.2.1 genotypes with imputation, AGPv3) were downloaded (www.panzea.org). SNPs with ˂20% missing genotype data and minor allele frequencies >5% were employed in the association analysis resulting in the final use of 80,440 SNPs and 25,457,708 SNPs for the RIL and diversity panel, respectively. Analyses were initially conducted in TASSEL version 5.0 using the GLM for the NAM RIL B73 × Ky21 subpopulation and the unified mixed linear model (MLM) for the Goodman association panel (Yu et al., 2006; Bradbury et al., 2007; Zhang et al., 2010). This was done to minimize false positives arising from differential population structures and familial relatedness (Yu et al., 2006). Differential population structure and familial relatedness are less common features in biparental RIL populations and enable GLM analyses for the B73 × Ky21 RILs (Ding et al., 2017, 2020). To improve GWAS analysis, the kinship matrix (K) was used jointly with population structure (Q). Final analyses were conducted with the R package GAPIT (Lipka et al., 2012). Manhattan plots were constructed in the R package qqman (version 0.1.4) (http://cran.rproject.org/web/packages/qqman; Turner, 2014).

### RNA and cDNA preparation

Total RNA was extracted from approximately 50-mg frozen plant powder using the InviTrap Spin Plant RNA Kit (Stratec) according to the manufacturer’s instructions. The RNA concentration and purity was assessed with a spectrophotometer (NanoDrop 2000c; Thermo Fisher Scientific). RNA (1 µg) was treated with DNaseI (Thermo Fisher Scientific), followed by cDNA synthesis using SuperScript III reverse transcriptase and oligo (dT)_20_ primers (Invitrogen) according to the manufacturer’s instructions.

### RNA-seq

To investigate gene expressional changes after fungal infection in W22, total RNA was extracted from leaf tissue (*n* = 4) as described above and sent to Novogene (Cambridge, UK) for RNA-seq library construction (polyA enrichment) and sequencing (NovaSeq PE150, paired reads, 6 G of raw data per sample). Trimming of the obtained sequencing reads and mapping to the maize W22 NRGene_V2 genome were performed with the program CLC Genomics Workbench (Qiagen Bioinformatics, Hilden, Germany; mapping parameter: length fraction, 0.8; similarity fraction, 0.9; max number of hits, 25). Empirical analysis of digital gene expression implemented in the program CLC Genomics Workbench was used for gene expression analysis. Raw reads were deposited in the NCBI Sequence Read Archive (SRA) under the BioProject accession PRJNA742147.

### RT-qPCR analysis

To verify gene expression data of flavonoid and BX pathway genes from RNA-Seq, RT-qPCR was performed. For the amplification of gene fragments with a length of 100–250 bp, specific primers were designed having a Tm ≥ 60°C, a GC content between 40% and 60%, and a primer length of 20–23 nucleotides (Supplemental Table S19). The primer specificity was confirmed by agarose gel electrophoresis, melting curve analysis, and by sequence verification of cloned PCR amplicons. Primer pair efficiency (90%–112%) was determined using standard curve analysis with two-fold serial dilutions of cDNA. UBCP and MEP (Manoli et al., 2012) were used as reference genes. The measurements were performed using 1 µL 1:10 diluted cDNA in 20-µL reaction mixture containing Brilliant III Ultra-Fast SYBR^®^ Green QPCR Master Mix (Agilent Technologies). All samples were run on a CFX Connect Real-Time PCR Detection System (Bio-Rad Laboratories, Hercules, CA, USA) in an optical 96-well plate. Four biological replicates per treatment were analyzed as triplicates. The following PCR conditions were applied for all reactions: Initial incubation at 95°C for 3 min followed by 40 cycles of amplification (95°C for 10 s, 60°C for 10 s). For all measurements, reads were taken during the extension step of each cycle and melting curve data were recorded at the end of cycling at 55–95°C. Cq values for the calculation of relative quantities were determined using CFX Manager version 3.1 software (Bio-Rad Laboratories).

### Cloning and heterologous expression of *OMT* genes in *E. coli*

The complete open reading frames of *FOMT2* (W22) and *FOMT4* (W22) were amplified from cDNA obtained from *B. maydis*-infected W22 leaves with the primer pairs listed in Supplemental Table S20. *FOMT3* and *FOMT5* were amplified from plasmids containing the synthesized codon-optimized open reading frames (see paragraph below). The PCR products were cloned into the expression vector pET100/D-TOPO (Invitrogen, Waltham, MA, USA) or pASK-IBA37plus (IBA Lifesciences, Göttingen, Germany) and fully sequenced. *BX10* (B73), *BX11* (B73), *BX12* (CML322), and *BX14* (B73) were provided as pASK-IBA37plus constructs by Vinzenz Handrick (Meihls et al., 2013; Handrick et al., 2016). For heterologous expression, the expression constructs were transferred in *E. coli* strain BL21 (DE3; Invitrogen). Liquid cultures were grown in lysogeny broth at 37°C and 220 rpm until an optical density at 600 nm (OD_600_) of 0.8–1, induced with a final concentration of 1 mM IPTG or 200 µg/L anhydrotetracycline, and subsequently incubated at 18°C and 220 rpm for 15 h. The cells were harvested by centrifugation at 5,000 *g* and 4°C for 10 min, resuspended in refrigerated extraction buffer (50 mM Tris–HCl pH 8, 500 mM NaCl, 20 mM imidazole, 10% (v/v) glycerol, 1% (v/v) Tween20, and 25 U/mL Benzonase Nuclease (Merck, Kenilworth, NJ, USA; freshly added)) and disrupted by sonication (4 × 20 s, with cooling on ice in between; Bandelin UW 2070). Cell debris was removed by centrifugation (16,000 *g* at 4°C for 20 min) and the N-terminal His-tagged proteins were purified from the supernatant using HisPur Cobalt Spin Columns (Thermo Fisher Scientific) according to the manufacturer’s instructions. Tris–HCl buffer (pH 8, without Tween-20; see above) containing either 20 mM or 250 mM imidazole was used for equilibration/washing and elution steps, respectively. The buffer of the eluted protein samples was exchanged for assay buffer (50 mM Tris–HCl pH 7, 10% (v/v) glycerol) by gel filtration using illustra NAP Columns (GE Healthcare, Chicago, IL, USA). Protein concentrations were determined by the Bradford method using Quick Start Bradford 1× Dye Reagent (Bio-Rad Laboratories) and prediluted BSA protein standards (Thermo Scientific).

### Gene synthesis

The complete open reading frames of *FOMT3* (B73_V3) and *FOMT5* (B73_V3) were synthesized after codon optimization for heterologous expression in *E. coli* by Eurofins MWG Operon (for sequences, see Supplemental Figure S22). CYP93G candidate genes (W22_V2) were codon optimized for *S. cerevisiae* and synthesized using the GeneArt gene synthesis service (Thermo Fisher Scientific) (for sequences, see Supplemental Figure S23). The synthetic genes were subcloned into the vector pUC57 (*FOMT3* and *FOMT5*) or pMA-T (*CYP93G* candidates), and the final constructs were verified by sequencing.

### Cloning and heterologous expression of CYP93G genes in yeast

The complete open reading frames of *F2H1* (W22), *F2H2* (W22), *FNSII2* (W22), *Zm00004b039147* (*CYP93G6-W22*), and *Zm00004b033036* (*CYP93F6-W22*) were synthesized as codon-optimized sequence (see above) and cloned as sticky-end fragments into the pESC-Leu 2d vector (Ro et al., 2008). *F2H1* (B73) was amplified from cDNA (for primers used, see Supplemental Table S19). For heterologous expression in yeast, the resulting constructs were transformed into the engineered *S. cerevisiae* strain WAT11 (Pompon et al., 1996) using the *S.c.* EasyComp Transformation Kit (Invitrogen) according to the manufacturer’s instructions. Subsequently, 30- mL Sc-Leu minimal medium (6.7 g/L yeast N_2_ base without amino acids, but with ammonium sulfate; 100 mg/L of each l-adenine, l-arginine, l-cysteine, l-lysine, l-threonine, l-tryptophan, and uracil; 50 mg/L of each l-aspartic acid, l-histidine, l-isoleucine, l-methionine, l-phenylalanine, l-proline, l-serine, l-tyrosine, and l-valine; 20 g/L d-glucose) was inoculated with single yeast colonies and grown overnight at 28°C and 180 rpm. For main cultures, 100 mL YPGA (Glc) full medium (10 g/L yeast extract, 20 g/L bactopeptone, 74 mg/L adenine hemisulfate, 20 g/L d-glucose) was inoculated with one unit OD_600_ of the overnight cultures and incubated under the same conditions for 30–35  h. After centrifugation (5,000 *g*, 16°C, 5 min), the expression was induced by resuspension of the cells in 100 mL YPGA (Gal) medium (see above, but including 20 g/L galactose instead of d-glucose) and grown for another 15–18 h at 25°C and 160 rpm. The cells were harvested by centrifugation (7,500 *g*, 10 min, 4°C), resuspended in 30 mL TEK buffer (50 mM Tris–HCl pH 7.5, 1 mM EDTA, 100 mM KCl) and centrifuged again. Then, the cells were carefully resuspended in 2 mL TES buffer (50 mM Tris–HCl pH 7.5, 1 mM EDTA, 600 mM sorbitol; freshly added: 10 g/L bovine serum fraction V protein and 1.5 mM β-mercaptoethanol) and glass beads (0.45–0.50 mm diameter; Sigma-Aldrich) were added until they reached the upper level of the cell suspension. For cell disruption, the suspensions were shaken by hand 5 times for 1 min, with cooling on ice for 1 min in between. The crude extracts were recovered by washing the glass beads 4 times with 5 mL TES. The combined washes were centrifuged (7,500 *g*, 10 min, 4°C), and the supernatant containing the microsomes was transferred into an ultracentrifuge tube. After ultracentrifugation (100,000 *g*, 90 min, 4°C), the supernatant was carefully removed and the microsomal pellet was gently washed with 2.5 mL TES buffer, then with 2.5 mL TEG buffer (50 mM Tris–HCl pH 7.5, 1 mM EDTA, 30% glycerol). The microsomal fractions were homogenized in 2 mL TEG buffer using a glass homogenizer (Potter-Elvehjem, Carl Roth). Aliquots were stored at −20°C until further use.

### In vitro enzyme assays

To test OMT activities, assays were set up containing 500 µM dithiothreitol (DTT) and 100 µM of the cosubstrate SAM. Substrates (flavonoids, caffeic acid, resveratrol, and DIMBOA-Glc) were added at 20 µM from 400 µM stock solutions made with 75% (v/v) dimethylsulfoxide (DMSO) in water. The assay buffer contained 50 mM Tris–HCl, pH 7, 10% (v/v) glycerol, and 0.8 µg purified recombinant protein (equivalent to ∼200 nM) was added in a total volume of 100 µL. Incubations were carried out for 1 h at 25°C and 300 rpm on a ThermoMixer C (Eppendorf). All assays were conducted in technical triplicates. For combined OMT assays, the first OMT was incubated with the substrate in half of the assay volume for 1 h and then the second OMT was added and incubated for another hour (final reaction volume: 100 µL). To obtain comparable activity estimates of the individual OMTs with different substrates, the decrease in substrate content compared to the EV control (substrate turnover) was used to calculate relative percent activity values. The highest percent substrate turnover per enzyme was set to 100% and the values for all other substrates were calculated accordingly.

CYP assays were performed with 1 mM cosubstrate NADPH, 20 µM substrate (naringenin or eriodictyol), assay buffer, and 60 µL microsomal fractions in a total volume of 300 µL and incubated for 2 h at 25°C and 300 rpm. All assays were repeated at least twice. For combined CYP and OMT activity assays, CYPs were pre-incubated with the substrate (30 µM) and NADPH (1 mM) in a 200 µL reaction volume for 1 h, before addition of recombinant OMT protein, DTT, and SAM (final reaction volume: 300 µL). All reactions were stopped by adding one volume of 100% methanol and centrifuged at 4,000 *g* for 5–10 min to remove denatured proteins. Product formation was monitored by the analytical methods described above.

### Purification of *O*-methylflavonoids from *E. coli* cultures

*E* *coli* BL21 (DE3) harboring a *FOMT2* or *FOMT4* expression construct (described above in “Cloning and heterologous expression of OMT genes in *E. coli*”) were grown in terrific broth at 37°C and 220 rpm, induced at an OD_600_ of 0.5 with a final concentration of 1 mM IPTG or 200 µg/L anhydrotetracycline, and incubated for another 2–3 h at 37°C and 220 rpm. Subsequently, flavonoid substrate (naringenin, apigenin, and scutellarein; solved in 75% (v/v) DMSO in water) was added to yield a final concentration of 25 µg/mL and the culture was incubated at 25°C and 220 rpm for 15 h. The culture was centrifuged at 5,000 *g* and 4°C for 20 min and the supernatant and the cell pellet were stored separately until further processing at 4°C and −20°C, respectively. For the production of 5,7-*O*-dimethylflavonoids, a *FOMT4* overexpressing culture was supplemented with the *FOMT2* culture supernatant in a ratio of 1:5 (e.g. 25 mL *FOMT2* culture supernatant/100 mL *FOMT4* overexpressing culture) and treated as described above. The culture supernatant was pre-purified by solid phase extraction (SPE) using a Chromabond HR-X column (15 mL, 500 mg, 83 µm, Macherey-Nagel). The column was washed with 40% (v/v) methanol in water and, after drying for 30–60 min under vacuum, hydrophobic components were eluted thrice with 50:50 (v/v) methanol:acetonitrile followed by two elution steps with 100% acetonitrile. The *E. coli* pellet was extracted with 100% methanol (2.5–4 mL per pellet resulting from 100 mL culture) for 2 times 5 min in an ultrasonic bath, with vortexing in between. Cell fragments were removed by centrifugation at 5,000 *g* and 4°C for 20 min. The *O*-methylflavonoid content of SPE fractions and pellet extract was analyzed using LC–UV–MS as described in the section “Untargeted LC–UV–MS analysis for purification*”* and fractions containing the desired compound were combined and dried using a Rotavapor R-114 rotary evaporation system (Büchi, Flawil, Switzerland). After redissolving in 100% methanol, partially occurring precipitate was removed using a Minisart SRP syringe filter (Sartorius) and water was added to yield a solution of 50:50 (v/v) methanol:water. The resulting *E. coli* extract was separated by HPLC-UV as described above in chapter “Semi-preparative HPLC-UV for purification.” Collected fractions were dried using a rotary evaporator and subsequently a desiccator, and subjected to NMR and LC–MS/MS analysis.

### Synthesis of 2-hydroxynaringenin

For synthesis of 2-hydroxynaringenin, we used a previously published method (Hao et al., 2016) and subsequently verified the structure by NMR.

### NMR spectroscopy for structure elucidation and quantitative analysis

^1^H NMR, ^13^C NMR, ^1^H-^1^H COSY, ^1^H-^1^H ROESY, ^1^H-^13^C HSQC, and ^1^H-^13^C HMBC spectra were measured at 300 K (except for 2-hydroxynaringenin, which was measured at 268 K) on either a Bruker Avance III HD 500 NMR spectrometer, equipped with a cryogenically cooled 5 mm TCI ^1^H{^13^C} probe or an Avance III HD 700 NMR spectrometer, equipped with cryogenically cooled 1.7 mm TCI ^1^H{^13^C} probe. Samples were measured in acetone-*d_6_* and MeOH-*d_3_*, respectively. Chemical shifts were referenced using the residual solvent signals at δ_H_ 2.05/δ_C_ 29.92 for acetone-*d_6_* and δ_H_ 3.31/δ_C_ 49.15 for MeOH-*d_3_*. Spectrometer control, data acquisition, and processing were accomplished using Bruker TopSpin version 3.6.1. Standard pulse programs as implemented in Bruker TopSpin were used. For quantitative NMR measurements the Bruker ERETIC-2 protocol was used. (-)-Catechin was used as an external standard.

### Sequence analysis and phylogenetic tree reconstruction

Maize *OMT* genes similar to *FOMT2* (B73), anthranilic acid methyltransferase 1 (*AAMT1*; B73), and *CCoAOMT1* (B73) or maize CYP93G candidates similar to *F2H1* (B73) were identified by BLASTP analysis available on MaizeGDB (https://www.maizegdb.org/), with B73 RefGen_V4 and W22 NRGene_V2 as search datasets, respectively. Multiple sequence alignments of maize *OMT* genes and characterized *FOMT* genes from several other species were generated using the MUSCLE codon algorithm implemented in the software MEGA version 7 (Kumar et al., 2016). Based on these alignments, phylogenetic trees were reconstructed with MEGA7 using a maximum likelihood method. Codon positions included were first + second + third+noncoding. All positions with < 80% site coverage (maize OMT phylogeny; Figure  2D) or < 90% site coverage (*FOMT* phylogeny; Supplemental Figure S6) were eliminated. Ambiguous bases were allowed at any position. A bootstrap resampling analysis with 1,000 replicates was performed to evaluate the topology of the generated trees. A substitution model test was performed with MEGA7 to identify the best-fitting substitution model for each dataset (for substitution model used, see respective figure legends). Phylogenetic analysis of maize genes similar to *F2H1* and characterized *F2H* and *FNSII* genes from other species was performed as described above, using all positions with ≥ 80% site coverage. All corresponding accession numbers and references are provided in Supplemental Tables S3 and S6. Amino acid sequence alignments were visualized with the software BioEdit.

### In vitro bioassays with *O*-methyl and non-*O*-methylflavonoids

Maize antifungal assays using self-purified or commercially available flavonoids (xilonenin, genkwanin, 5-*O*-methylapigenin, 5-*O*-methylnaringenin, apigenin, and naringenin; see Supplemental Table S17) were performed using the Clinical and Laboratory Standards Institute M38-A2 guidelines (Schmelz et al., 2011). Fungal cultures of *R.* *microsporus* (Northern Regional Research Laboratory [NRRL] stock no. 54029), *F. verticillioides* (NRRL stock no. 7415), *F. graminearum* (NRRL stock no. 31084), and *B. maydis* were grown on V8 agar for 12 d before the quantification and final use as 2.5 × 10^4^ conidia/mL (Huffaker et al., 2011). Using a 96-well microtiter plate, each well contained 200 µL of broth medium, fungal inoculum, and 0.5 µL of either pure ethanol or ethanol containing dilutions of flavonoids. All assays were conducted in four to five technical replicates. The flavonoid concentrations used in the bioassays (33 and 100 µg/mL) were chosen based on their abundance in fungal-infected tissue with the knowledge that (1) phytoalexin accumulation is highly localized to necrotic tissues and (2) that leaves used for metabolite quantification contained only 10–20% necrotic tissue (Figure  1A;Supplemental Figure S16). The actual flavonoid concentrations at the site of fungal attack are likely to be significantly higher than those measured at the whole leaf level. A Synergy4 (BioTek Instruments) reader was used to monitor fungal growth at 30°C through periodic measurements of changes in OD_600_.

### Statistical analysis

Statistical analyses were performed using SigmaPlot version 11.0 for Windows (Systat Software). The statistical test applied is indicated in the respective figure and table legends. Whenever necessary, the data were log-transformed to meet statistical assumptions such as normality and homogeneity of variances. Statistical significance of metabolomic data obtained by untargeted LC–MS was tested using the *t* test implemented in MetaboScape version 4.0 software (Bruker Daltonics).

To investigate whether the amount of flavonoids and *O*-methylflavonoids changed due to infection with *B. maydis* 2 or 4 d after infection, two-way analyses of variance (ANOVAs) were applied. In case of significant differences, Tukey’s honestly significant difference (HSD) tests were performed. To account for the variance heterogeneity of the residuals, data were either log-transformed prior to the ANOVA or generalized least squares models (gls from the nlme library; Pinheiro et al., 2020) were applied. The varIdent variance structure was used. Whether the different variance of fungal treatment, time, or the combination of both factors should be incorporated into the model, was determined by comparing models with different variance structures with a likelihood ratio test and choosing the model with the smallest akaike information criterion (AIC). The influence (*P*-values) of the explanatory variables was determined by sequential removal of explanatory variables starting from the full model, and comparison of the simpler with the more complex model with a likelihood ratio test (Zuur et al., 2009). Differences between factor levels were determined by factor level reduction (Crawley, 2013). Data were analyzed with R version 4.0.3 (R Core Team, 2020).

### Accession numbers

Sequence data for FOMT2-W22 (MZ484743) and FOMT4-W22 (MZ484744) can be found in the NCBI GenBank (https://www.ncbi.nlm.nih.gov/genbank/) under the corresponding identifiers. Raw reads of the RNA-seq experiment were deposited in the NCBI SRA under the BioProject accession PRJNA742147.

## Supplemental data  

The following materials are available in the online version of this article.

**Supplemental Figure S1.** MS/MS spectra of putative 5- and 7-*O*-methylflavonoids.

**Supplemental Figure S2.** Association mapping using B73 × Ky21 RIL with the GLM and 80,440 SNPs.

**Supplemental Figure S3.** GWAS mapping reveals association between the occurrence of genkwanin and *FOMT4*.

**Supplemental Figure S4.** Schematic chromosomal array of *FOMT2* and *FOMT3* in B73 and W22.

**Supplemental Figure S5.** Amino acid sequence alignment of FOMT2/3.

**Supplemental Figure S6.** Phylogenetic tree of maize *FOMT* genes characterized in this study, closely related maize *OMT* genes, and characterized *FOMT* genes from other monocots and dicots.

**Supplemental Figure S7.** Expression of *BX OMT* genes in W22 upon fungal infection.

**Supplemental Figure S8.** Regiospecific *O*-methylation and elution patterns of FOMT2 and FOMT4 products.

**Supplemental Figure S9.** Fragmentation patterns of 2-hydroxynaringenin and its *O*-methyl derivatives.

**Supplemental Figure S10.** GWAS mapping reveals association between the occurrence of xilonenin tautomers and *FOMT2/3*.

**Supplemental Figure S11.** Amino acid sequence alignment of Poaceae F2Hs belonging to the CYP93G subfamily.

**Supplemental Figure S12.** Enzymatic activity of CYP93G family members similar to F2H1 (CYP93G5) with naringenin or eriodictyol.

**Supplemental Figure S13.** NMR chemical shift data of xilonenin tautomers (in MeOH-*d_3_*).

**Supplemental Figure S14.** The two xilonenin tautomers exhibit different UV absorption.

**Supplemental Figure S15.** De novo production of flavonoids in different maize lines after fungal infection.

**Supplemental Figure S16.** Visible signs of infection on hybrid maize after inoculation with different pathogenic fungi.

**Supplemental Figure S17.** Large-scale transcriptomic and metabolomic changes upon SLB infection.

**Supplemental Figure S18.** Expression of the BX biosynthetic pathway during fungal infection.

**Supplemental Figure S19.** RT-qPCR validation of flavonoid and BX pathway gene expression results in noninfected and fungus-infected W22 leaves.

**Supplemental Figure S20.** Antifungal activity of naringenin and 5-*O*-methylnaringenin.

**Supplemental Figure S21.** Antifungal activity of apigenin and 5-*O*-methylapigenin.

**Supplemental Figure S22.** Codon*-*optimized gene sequences of *FOMT3-B73* and *FOMT5-B73* synthesized for expression in *E. coli*.

**Supplemental Figure S23.** Codon*-*optimized gene sequences of CYP93G candidates synthesized for expression in *S. cerevisiae.*

**Supplemental Table S1.** *P*-values of *t* test analysis to determine statistical significant differences of flavonoid content between treatments obtained by the LC–MS measurements shown in Supplemental Figure 1B.

**Supplemental Table S2.** Expression of maize genes putatively involved in the phenylpropanoid pathway, flavonoid pathway, BX pathway or terpenoid biosynthesis.

**Supplemental Table S3.** MaizeGDB/GenBank accessions and references corresponding to Figure  2D and Supplemental Figure S6.

**Supplemental Table S4.** NMR structure elucidation of 5-/7-*O*-methyl and 5,7-*O*-dimethylflavonoids.

**Supplemental Table S5.** Product formation of maize OMTs with different substrates.

**Supplemental Table S6.** GenBank accessions and references corresponding to Figure  4B.

**Supplemental Table S7.** Quantification of flavonoids in leaf tissue of different maize inbred lines after infection with *B. maydis*.

**Supplemental Table S8.** Quantification of *O*-methylflavonoids in leaf tissue of different maize inbred lines after infection with *B. maydis*.

**Supplemental Table S9.** Statistical values for the analysis of the amount of non-*O*-methylated- and *O*-methylated flavonoids in different maize lines according to treatment, duration of treatment (day), and the interaction between treatment and its duration corresponding to the experiments shown in Figure  5A and Supplemental Figure S15.

**Supplemental Table S10.** Quantification of flavonoids and *O*-methylflavonoids in leaf tissue of hybrid maize (“Sweet Nugget”) after treatment with different pathogenic fungi and CHT.

**Supplemental Table S11.** Relative quantification of BXs in leaf tissue of different maize inbred lines after infection with *B. maydis*.

**Supplemental Table S12.** MS settings used for the analysis on the timsTOF mass spectrometer.

**Supplemental Table S13.** MS settings used for the analysis on the QTRAP 6500+.

**Supplemental Table S14.** Mass analyzer settings used for the analysis of flavonoids and additional phenylpropanoids on the QTRAP 6500+.

**Supplemental Table S15.** Mass analyzer settings used for the analysis of flavonoid glycosides on the QTRAP 6500+.

**Supplemental Table S16.** Mass analyzer settings used for the analysis of BXs on the QTRAP 6500+.

**Supplemental Table S17.** Authentic standards used for identification and quantification.

**Supplemental Table S18.** Maize mapping lines used for GWASs in the Goodman diversity panel and Quantitative Trait Loci mapping in NAM subpopulation B73 × Ky21.

**Supplemental Table S19.** RT-qPCR primers.

**Supplemental Table S20.** PCR primers for the amplification of full-length open reading frames of investigated FOMTs and CYP93Gs.

**Supplemental Data Set S1.** Complete RNA-seq data set derived from damaged and water-treated control leaves (DAM) and damaged and *B. maydis*-infected leaves (SLB) of W22 after 4 d of treatment (*n* = 4).

**Supplemental Data Set S2.** NMR spectra.

## Supplementary Material

kiab496_Supplementary_Data

## Notes

T.G.K., E.A.S., C.F., V.H., and J.G. designed the research. C.F., V.H., Y.D., Y.N., K.L., and S.P. performed the research. C.P., Y.N., B.S., C.C.H., and G.C.F. performed NMR analysis and structure elucidation. C.F., V.H., A.H., G.K., E.A.S., Y.D., and T.G.K. analyzed the data. C.F., T.G.K., E.A.S., and J.G. wrote the article with input from all other authors.

The author responsible for distribution of materials integral to the findings presented in this article in accordance with the policy described in the Instructions for Authors (https://academic.oup.com/plphys/pages/general-instructions) is Tobias G. Köllner (koellner@ice.mpg.de).
