# Supplementary material for: Biosynthesis and antifungal activity of fungus-induced O-methylated flavonoids in maize
Source: Plant Physiol. 2021 Oct 27;188(1):167–90. doi: 10.1093/plphys/kiab496 (PMC8774720; doi:10.1093/plphys/kiab496)
Supplement: kiab496_Supplementary_Data [file kiab496_supplementary_data.zip › kiab496-suppl_data/2021-10-29_supplemental_figures.pdf]

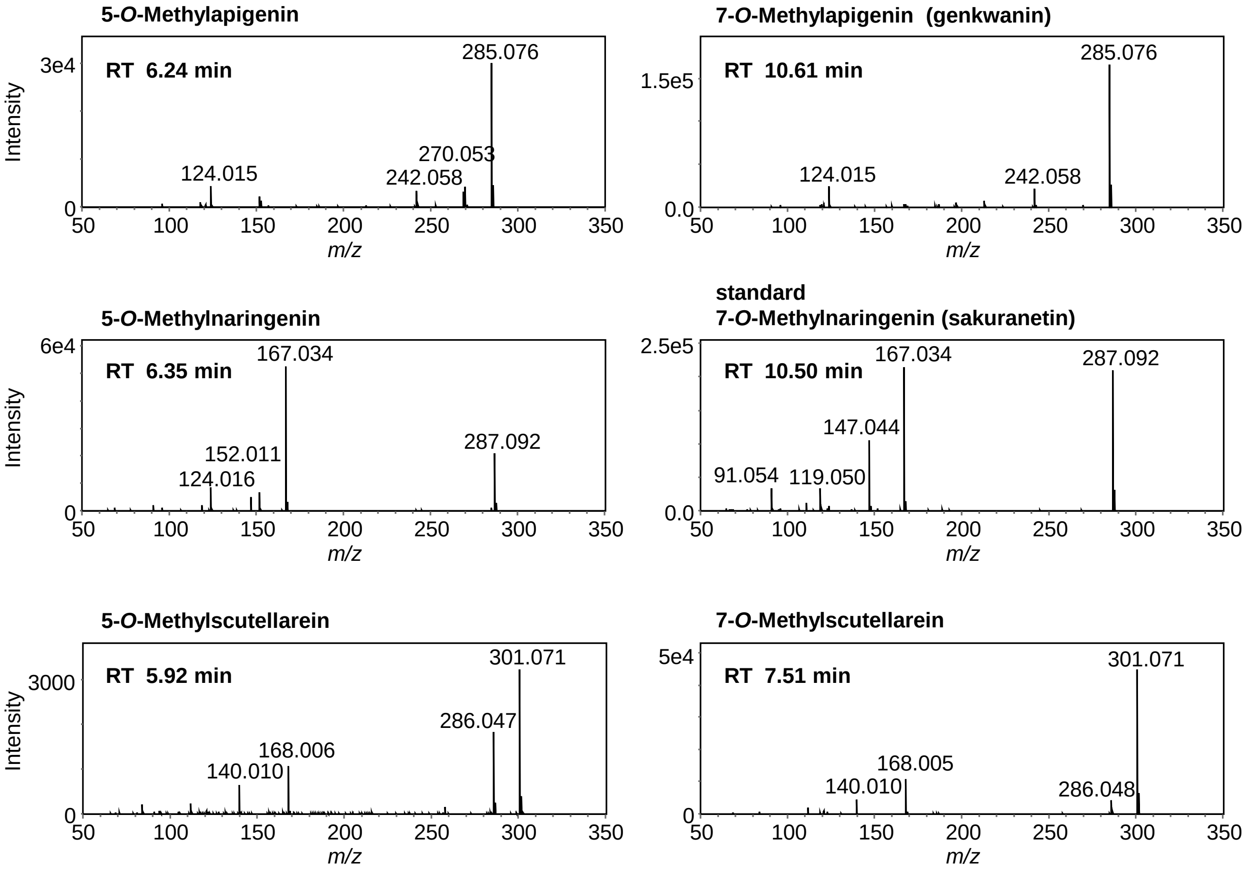

Supplemental Figure S1. MS/MS spectra of putative 5- and 7-*O*-methylflavonoids. The compounds were observed in *B. maydis*-infected W22 and/or B75 leaf tissue using untargeted LC-MS (full scan and auto MS/MS mode) as described in the methods section. The MS/MS spectrum of sakuranetin was obtained using a commercially available standard. 5- and 7-*O*-methylderivatives have similar MS/MS spectra, but differ greatly in retention time (RT).

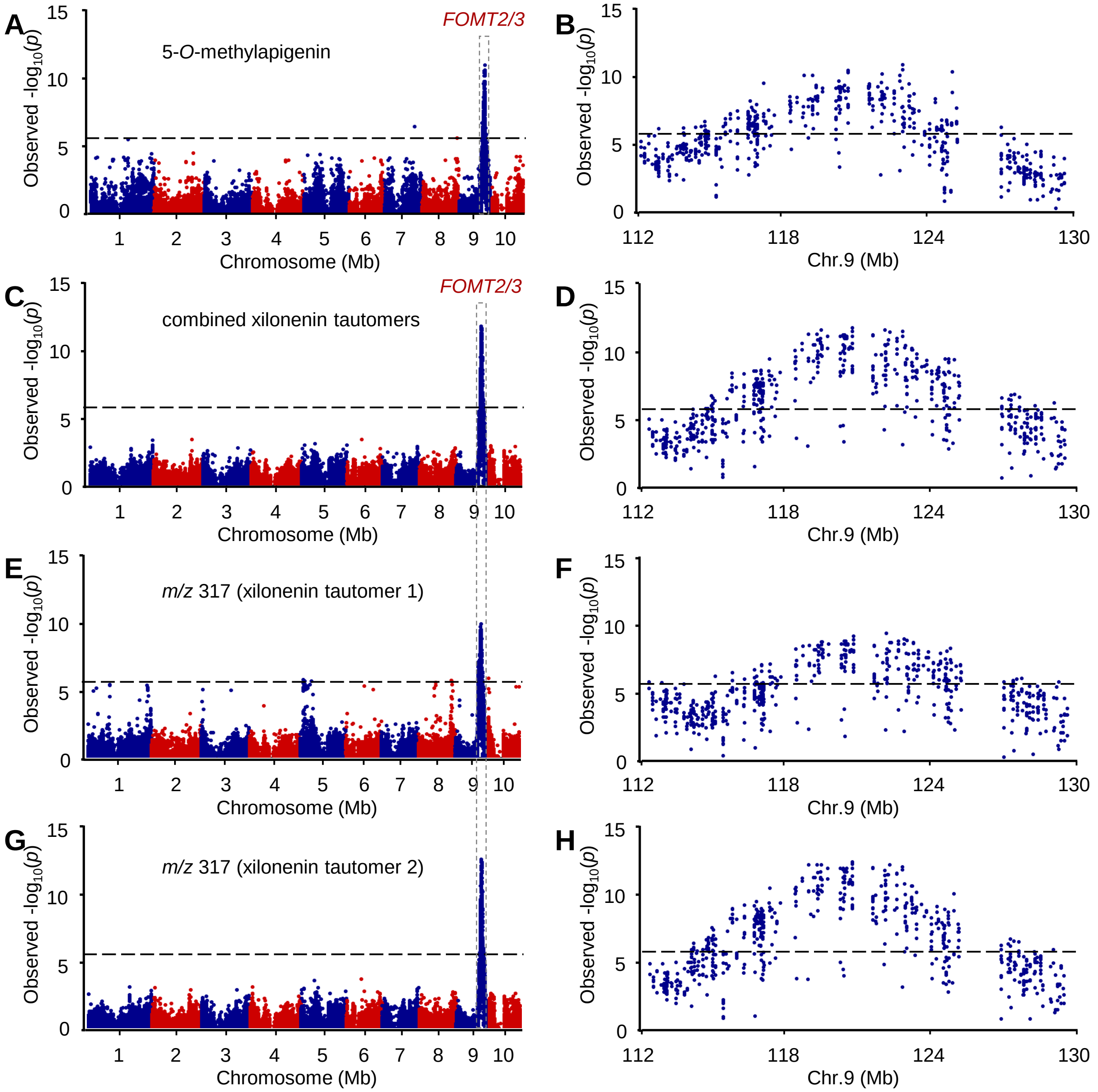

Supplemental Figure S2. Association mapping using B73 × Ky21 RIL with the GLM and 80,440 SNPs. Left panel (A,C,E,G): Manhattan plots of the association analysis of the indicated metabolites. The most statistically significant SNPs are located within the region of the maize FOMT2/3 on chromosome 9 (FOMT2, Chr.9:119,779,040-119,780,565 bp; FOMT3, Chr9: 119,838,646-119,840,122 bp; B73 RefGen_v2). The black dashed line denotes the false discovery rate (< 0.05 at –log10[P]) using a Bonferroni correction. **Right panel (B, D, F, H):** Regional Manhattan plots representing a 'zoomed-in' view of the signal between 112.0 Mb and 130.0 Mb on chromosome 9, and each dot representing a single SNP.

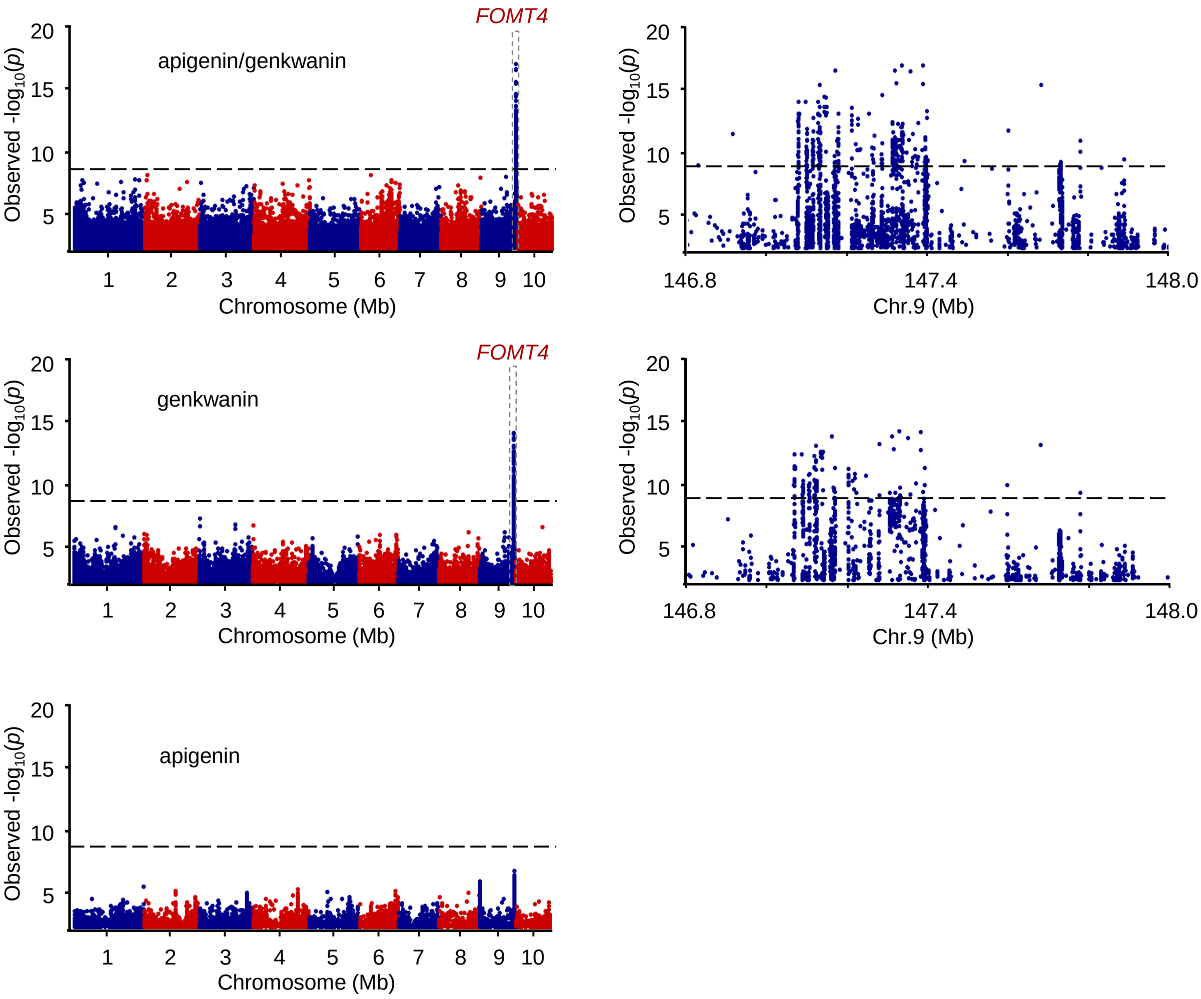

Supplemental Figure S3. GWAS mapping reveals association between the occurrence of genkwanin and *FOMT4*. Left panel: Manhattan plots of the association analysis (mixed linear model) of the indicated metabolites in the stems of maize plants from the Goodman diversity panel following 3 days of fungal elicitation. The most statistically significant SNPs associated with genkwanin and the ratio of apigenin to genkwanin are located within the region of the maize FOMT4 on chromosome 9 (Chr9: 147,148,251-147,149,436 bp; B73 RefGen_v3), while no significant SNPs are associated with apigenin. The black dashed line denotes the false discovery rate (< 0.05 at –log10[P]) using a Bonferroni correction. **Right panel:** Regional Manhattan plots representing a 'zoomed-in' view of the signal between 146.8 Mb and 148.0 Mb on chromosome 9, and each dot representing a single SNP.

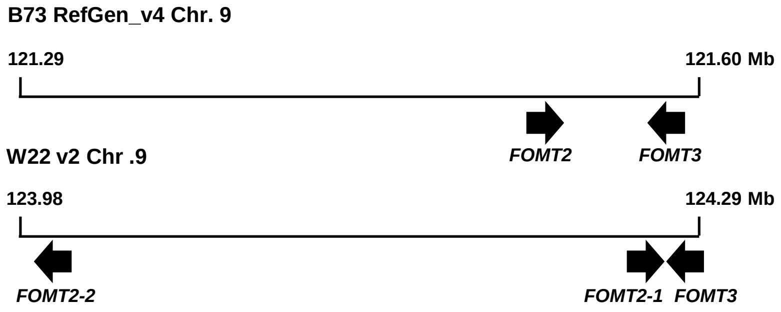

Supplemental Figure S4. Schematic chromosomal array of *FOMT2* and *FOMT3* in B73 and W22. *FOMT2-B73,* Zm00001d047192; *FOMT3-B73,* Zm00001d047194; *FOMT2-W22-1,* Zm00004b033403; *FOMT2-W22-2,* Zm00004b033399; *FOMT3-W22*, Zm00004b033404.

```
                     10        20        30        40        50        60
            ....|....|....|....|....|....|....|....|....|....|....|....|
FOMT2-B73   ---MALSTQDLLEAHVELWHQSLCYAKSLALAVALDLRIPDAIHHHGGSATLPQILAETA
FOMT2-W22-1 MHTMALSTQDLLEAHVELWHQSLCYAKSLALAVALDLRIPDAIHHHGGSATLPQILAETA
FOMT2-W22-2 MHTMALSTQDLLEAHVELWHQSLCYAKSLALAVALDLRIPDAIHHHGGSATLPQILAETA
FOMT3-B73   MAFTEESSQDLLQAHDELWHQSVSYLKSLALTVALDLRIPDAIHHHGGGATLLQILDKTA

                     70        80        90       100       110       120
            ....|....|....|....|....|....|....|....|....|....|....|....|
FOMT2-B73   AHPSKLRALRRLMRVLTVSGTFSVEQQPPAGGDDSTVDAPDEAVYRLTAASRFLVSDEVS
FOMT2-W22-1 AHPSKLRALRRLMRVLTVSGTFSVEQQPPAGGDDSTVDAPDEAVYRLTAASRFLVSDEVS
FOMT2-W22-2 AHPSKLRALRRLMRVLTVSGTFSVEQQPPAGGDDSTVDAPDEAVYRLTAASRFLVSDEVS
FOMT3-B73   LHQSKLRALRRLMRVLTVSGTFSVVQQPPCGDDDST-------VYRLTAASRFLVSFEVS

                    130       140       150       160       170       180
            ....|....|....|....|....|....|....|....|....|....|....|....|
FOMT2-B73   SSTLAPFVSLALHPIAVSPHTMGICAWFRQEQREPSPYGLAFQQTFPTIWEHADDVNALL
FOMT2-W22-1 SATLAPFVSLALHPIAVSPHTMGICAWFRQEQREPSPYGLAFQQTFPTIWEHADDVNALL
FOMT2-W22-2 SATLAPFVSLALHPIAVSPHTMGICAWFRQEQREPSPYGLAFQQTFPTIWEHADDVNALL
FOMT3-B73   SATLAPFMSVVLHPISQSSHARGICAWFRQEHHDPSAFGLAFGQA-PTIWEHADDINAIL

                    190       200       210       220       230       240
            ....|....|....|....|....|....|....|....|....|....|....|....|
FOMT2-B73   NKGMVADSRFLMPIMLRECGE-VFRGIESLVDVGGGHGGATAAIAAAFPHLKCSVLDLPH
FOMT2-W22-1 NKGMVADSRFLMPIMLRECGE-VFRGIESLVDVGGGHGGATAAIAAAFPHLKCSVLDLPH
FOMT2-W22-2 NKGMVADSRFLMPIMLRECGE-VFRGIESLVDVGGGHGGATAAIAAAFPHLKCSVLDLPH
FOMT3-B73   NKGLAAQSRFLVPVMLRECGEAVFRGIDSLVDVGGGHGGAATAIAAAFPHLKCSVLDLPH

                    250       260       270       280       290       300
            ....|....|....|....|....|....|....|....|....|....|....|....|
FOMT2-B73   VVAGAPSDVNVQFVAGNMFQSIPPATAVFLKTTLHDWGDDECVKILKNCRQAISPRDAGG
FOMT2-W22-1 VVAGAPSDVNVQFVAGNMFQSIPPATAVFLKTTLHDWGDDECVKILKNCRQAISPRDAGG
FOMT2-W22-2 VVAGAPSDVNVQFVAGNMFQSIPPATAVFLKTTLHDWGDDECVKILKNCRQAISPRDAGG
FOMT3-B73   VVAGAPSDGNVQFVAGDMFQSIPPATAVFLKTALHDWGDDECVKILKNCRQAISPCDEGG

                    310       320       330       340       350       360
            ....|....|....|....|....|....|....|....|....|....|....|....|
FOMT2-B73   KVIILDMVVGYGQPNITHLETQVMFDLYIMTVNGAERDEQEWKKIFIEAGFKDYKILPIL
FOMT2-W22-1 KVIILDMVVGYGQPNITHLETQVMFDLYIMTVNGAERDEQEWKKIFIEAGFKDYKILPIL
FOMT2-W22-2 KVIILDMVVGYGQPNITHLETQVMFDLYIMTVNGAERDEQEWKKIFIEAGFKDYKILPIL
FOMT3-B73   KVIIMDMVVGYDESNTKRLEVQILFDLFIMMVNGAERDEQEWKKIFIQAGFKDYKILPVV

                    370
            ....|....|.
FOMT2-B73   GALSVIEVYP*
FOMT2-W22-1 GALSVIEVYP*
FOMT2-W22-2 GALSVIEVYP*
FOMT3-B73   GSLSVIEVYP*
```

Supplemental Figure S5. Amino acid sequence alignment of FOMT2/3. The amino acid sequences encoded by *Zm00001d047192* (*FOMT2-B73*), *Zm00004b033403* (*FOMT2-W22-1*), *Zm00004b033399* (*FOMT2-W22-2*), and *Zm00001d047194* (*FOMT3-B73*) were aligned using the MUSCLE codon algorithm implemented in the software MEGA7 and visualized with the program BioEdit. Identical amino acids are shaded in black and similar amino acids in grey. FOMT2-B73 shares 99% and 79% amino acid identity with FOMT2-W22-1/2 and FOMT3-B73, respectively. **Note:** Unlike the W22 database sequences displayed here, the cloned FOMT2 sequence contains a D instead of an E on alignment position 17.

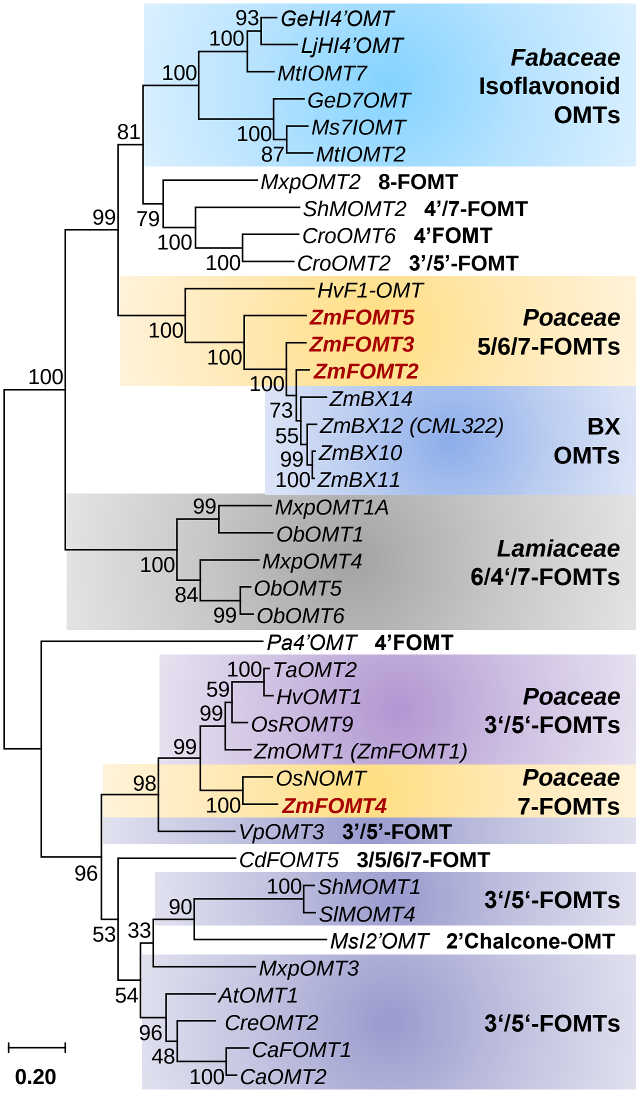

Supplemental Figure S6. Phylogenetic tree of maize *FOMT* genes characterized in this study, closely related maize *OMT* genes and characterized *FOMT* genes from other monocots and dicots. The tree was inferred using the maximum likelihood method based on the General Time Reversible model, including gamma distributed rate variation among sites (+G, 2.2239). Bootstrap values (n = 1000) are shown next to each node. The tree is drawn to scale, with branch lengths measured in the number of substitutions per site. All positions with < 90% site coverage were eliminated. Maize *FOMTs* investigated in this study are highlighted in red. The gene accession numbers and references are provided in Supplemental Table S3.

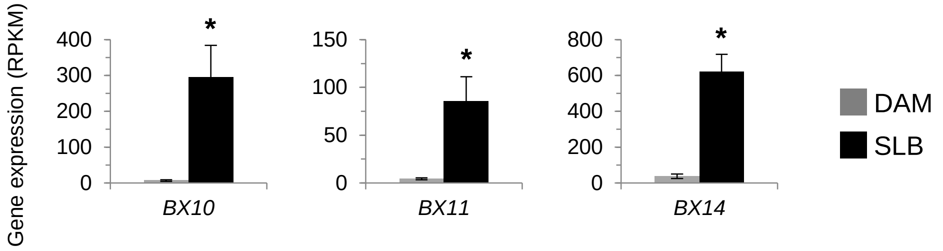

Supplemental Figure S7. Expression of *BX OMT* genes in W22 upon fungal infection.
Transcript abundance of *BX10*, *BX11* and *BX14* derived from RNA-seq of damaged and either water treated (DAM) or *B. maydis*-infected (SLB) W22 leaves harvested after 4 days. Gene expression is given as reads per kilobase million (RPKM; Means ± SE; $n = 4$). Stars indicate significant differences ($P < 0.05$) between treatments using a Bonferroni correction (for statistical values, see Supplemental Table S2).

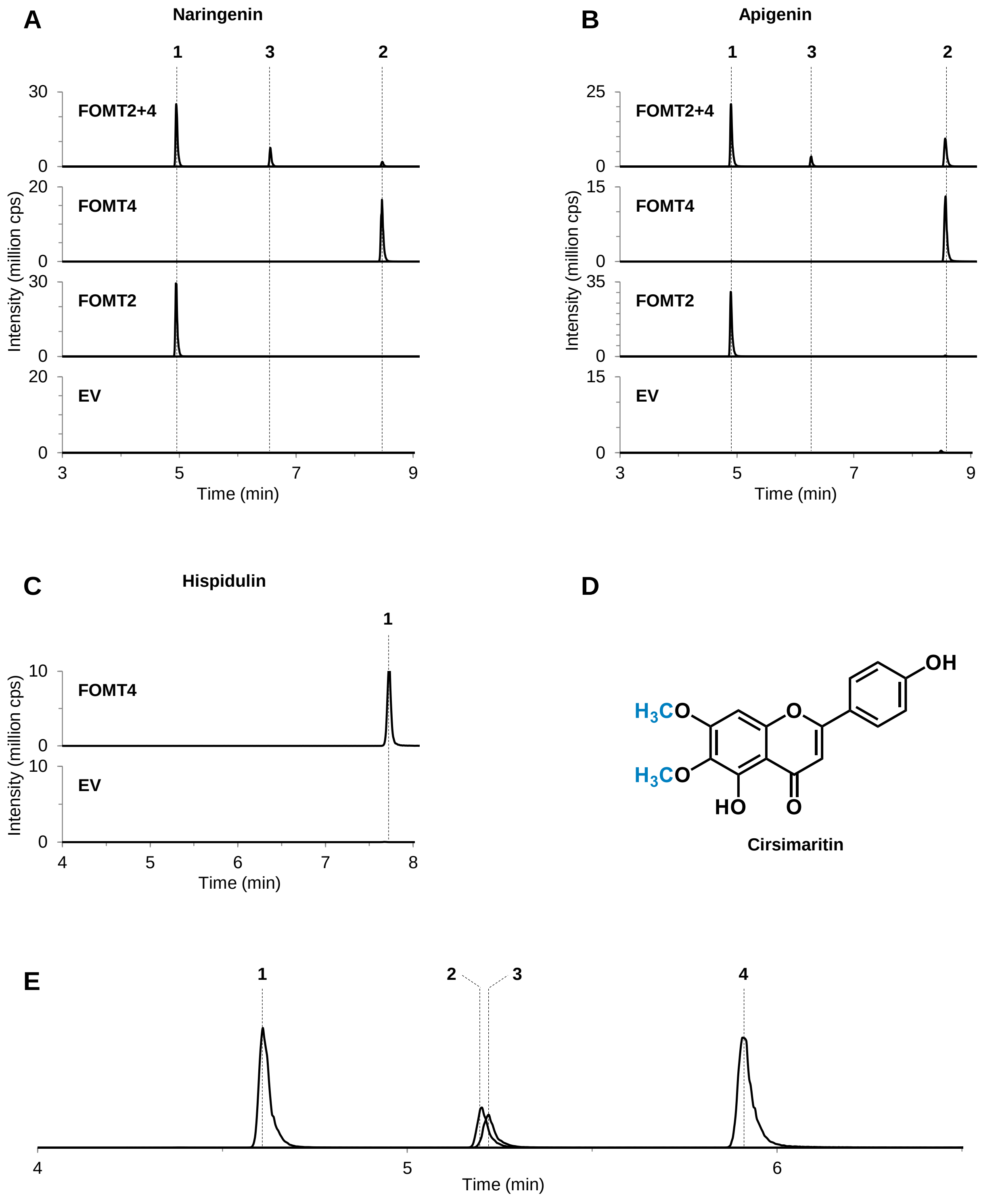

Supplemental Figure S8. Regiospecific *O*-methylation and elution patterns of FOMT2 and FOMT4 products. The purified recombinant enzymes as well as the empty vector control (EV) were incubated with the substrate naringenin **(A)**, apigenin **(B)** or hispidulin **(C)** in presence of the cosubstrate *S*-adenosyl-L-methionine. Reaction products were analysed by LC-MS/MS. Peak numbers in panel A: 1, 5-*O*-methylnaringenin; 2, 7-*O*-methylnaringenin (sakuranetin); 3, 5,7-*O*-dimethylnaringenin. Peak numbers in panel B: 1, 5-*O*-methylapigenin; 2, 7-*O*-methylapigenin (genkwanin); 3, 5,7-*O*-dimethylapigenin. Peak numbers in panel C: 1, 6,7-*O*-dimethylscutellarein (cirsimaritin). cps, counts per second. **(D)** Structure of cirsimaritin, the FOMT4 product in panel C. **(E)** The elution pattern of flavonoid substrates and their 5- and 7-*O*-methylated products, illustrated using the example of scutellarein. Peak numbers: 1, 5-*O*-methyl(scutellarein); 2, 5,7-*O*-dimethyl(scutellarein); 3, substrate (scutellarein); 4, 7-*O*-methyl(scutellarein). **Note:** For illustration, the peaks of different enzyme assays are combined in the chromatogram.

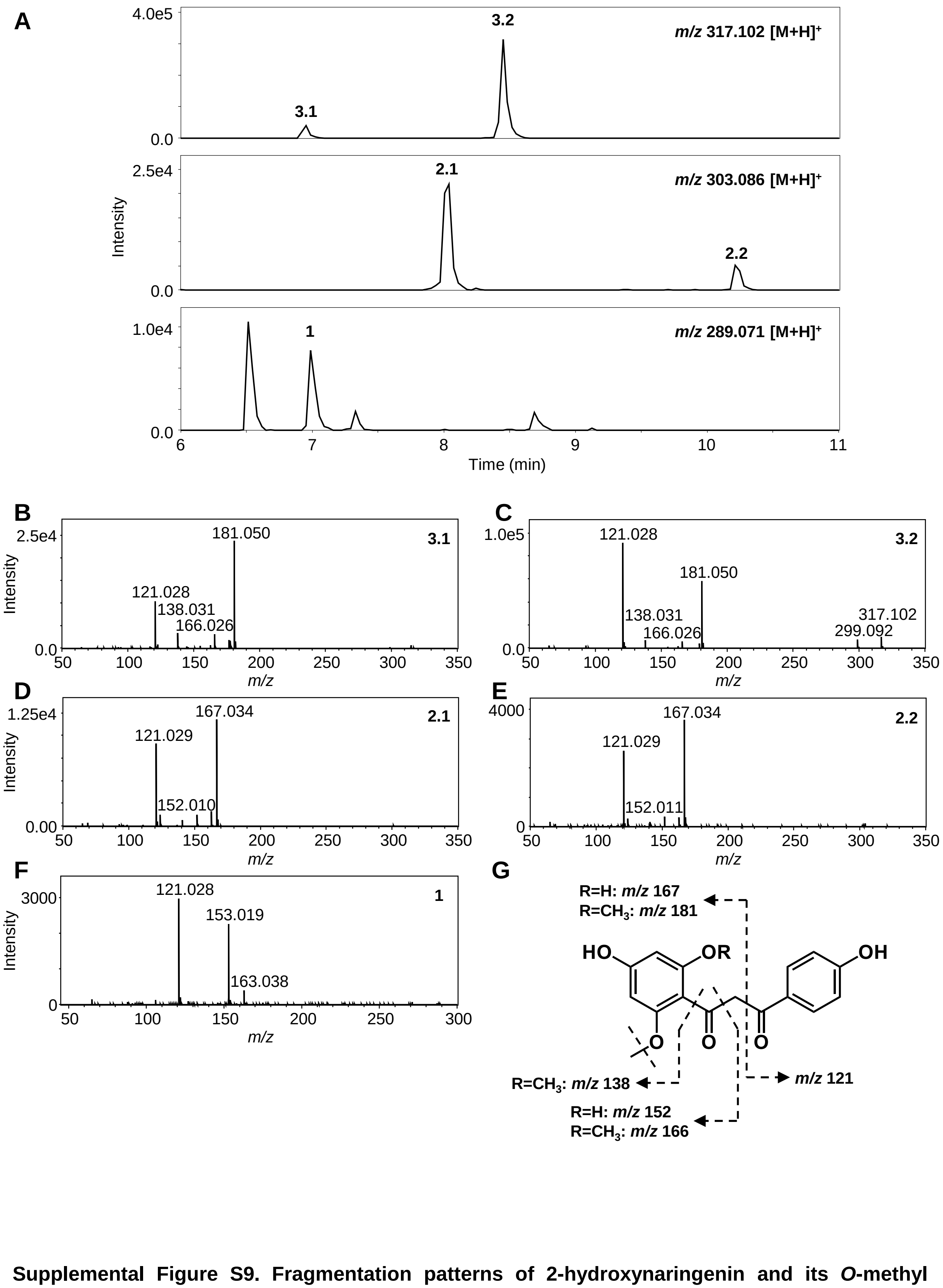

Supplemental Figure S9. Fragmentation patterns of 2-hydroxynaringenin and its *O*-methyl derivatives. Extracted ion chromatograms **(A)** and MS/MS spectra of 2-hydroxynaringenin **(F)** and different *O*-methyl derivatives **(B-E)**, which were consistent with the fragmentation patterns of a mono- or di-*O*-methylated 2-hydroxynaringenin **(G)**. The compounds were observed in *B. maydis*-infected W22 and/or B75 leaf tissue using untargeted LC-MS (full scan and auto MS/MS mode) as described in the methods section. 1, 2-hydroxynaringenin; 2.1 and 2.2, *O*-methyl-2-hydroxynaringenin; 3.1 and 3.2, *O*-dimethyl-2-hydroxynaringenin (xilonenin).

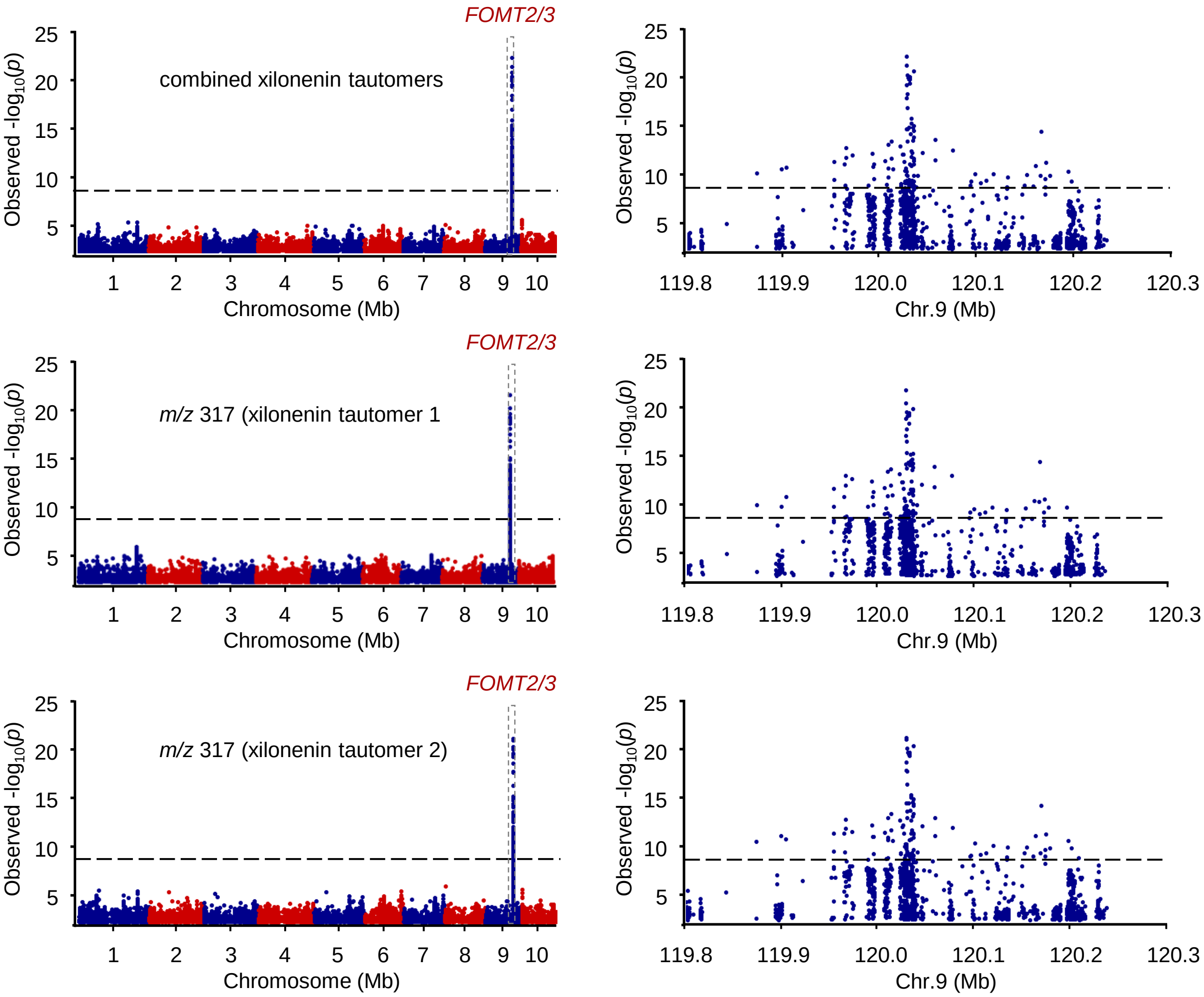

Supplemental Figure S10. GWAS mapping reveals association between the occurrence of xilonenin tautomers and *FOMT2/3*. Left panel: Manhattan plots of the association analysis (mixed linear model) of the indicated metabolites in the stems of maize plants from the Goodman diversity panel following 3 days of fungal elicitation. The black dashed line denotes the 5% Bonferroni corrected threshold for 25,457,708 SNP markers. The most statistically significant SNPs are located within the region of the maize FOMT2/3 on chromosome 9 (FOMT2, Chr9: 120,033,582-120,035,107 bp; FOMT3, Chr9: 120,093,188-120,094,664 bp; B73 RefGen_v3). **Right panel:** Regional Manhattan plots representing a ‘zoomed-in’ view of the signals between 119.8 Mb and 120.3 Mb on chromosome 9, and each dot representing a single SNP.

```
                          10        20        30        40        50        60
                  ....|....|....|....|....|....|....|....|....|....|....|....|
ZmCYP93G5(B73)    MEAD--AAAASGG------------LALLPAVLLLVALSTLVFS-TWSNRNSRLPPSPMA
ZmCYP93G5(W22)    MEADAAAAAASGG------------LALLPGVLLLVALSTLVFS-TWSNRNSRLPPSPMA
ZmCYP93G15(W22)   ME----AAAAVTP------------LALL---LLLLLVTRWRWS---SSRNSKLPPSPMA
SbCYP93G3         MEAAEAVTVGVGGSIGAPSAPGGVLLLFLLALSTLIIIIRWRWN---NNRNSRLPPSPMA
OsCYP93G2         MEE---GVVGGGG------------AAVLVALLVTVVLAVMRSAGSRSSKRGRLPPSPMA

                          70        80        90       100       110       120
                  ....|....|....|....|....|....|....|....|....|....|....|....|
ZmCYP93G5(B73)    LPLIGHLHLIRPPPHRAFDRILARYGPLVYLRLGPSTHCVVAGTADAARDLLKHEASIPE
ZmCYP93G5(W22)    LPLIGHLHLIRPPPHRAFDRILARYGPLVYLRLGPSTHCVVAGTADAARDLLKHEASIPE
ZmCYP93G15(W22)   LPLIGHLHLIRRLPHRSLDRILARYGPLVYLRLGPSTHCIVAGTADAARDLLKHEASIPQ
SbCYP93G3         LPLVGHLHLIRSPPHRSLDRIVKRYGPLVYLRLGPSTHCVVAGTADAARDLLKHEASIPE
OsCYP93G2         LPIIGHLHLIRPPPHRAFDRILARHGPLVYLRLGPSTHCVVIGSADVARDFLKFEASIPE

                         130       140       150       160       170       180
                  ....|....|....|....|....|....|....|....|....|....|....|....|
ZmCYP93G5(B73)    RPLTAVTRHLAYDDAGFAFAPYGPHWRFMKRLCMSELLGPRTVDQLRPVREAELAAVLEA
ZmCYP93G5(W22)    RPLTAVTRHLAYDDAGFAFAPYGAHWRFMKRLCMSELLGPRTVDQLRPVREAELAAVLEA
ZmCYP93G15(W22)   RPLTVVARHLAYDDAGFAFAPYGAHWRFMKRLCMSELLGPRTVDQLRPVREAELAAVLGA
SbCYP93G3         RPITVVAHHLAYGDAGFAFAPYGAHWRFMKRLCMSELLGPRTVDQLRPVREAELAAVLGA
OsCYP93G2         RPPTAVTRQLAYGKAGFAFAPYGAYWRFVKRLCMSELLGPRTVELLRPVRAAELADVLRA

                         190       200       210       220       230       240
                  ....|....|....|....|....|....|....|....|....|....|....|....|
ZmCYP93G5(B73)    ARQAAAA--REPIDVSRHLISMSNNAIMRMVASALPGHMTEAARDCAKHVAELVGAFNVE
ZmCYP93G5(W22)    ARQAAAA--REPIDVSRHLISMSNNAIMRMVASALPGHMTEAARDCAKHVAELVGAFNVE
ZmCYP93G15(W22)   AASASASGEGEPIDVSRHLISMSNNAIMRMVASALPGHMTEAARDCAKHVAELVGAFNIE
SbCYP93G3         ARQASAS--GERIDVSRYLISMSNNAIMRMVASALPGHMTEAARDCAKHVAELVGAFNIE
OsCYP93G2         AQSAAER--GEGVDMSHELVRMANNSIMRMVASALPGEMAEAARDCAKQVAELVGAFNAE

                         250       260       270       280       290       300
                  ....|....|....|....|....|....|....|....|....|....|....|....|
ZmCYP93G5(B73)    DYVGLCRGWDLQGLTRRTREVRDKFDALLEIMITGKEESRRRRHATTDTGGGTKDLLDIL
ZmCYP93G5(W22)    DYVGLCRGWDLQGLTRRTREVRDKFDALLEIMITGKEESRRRRHATTDTGGGTKDLLDIL
ZmCYP93G15(W22)   DYVGLCRGWDLQGLTRRTRQVRDKFDALLEVMITAKEEKRRRRQ---QGQGDHHDLLDIL
SbCYP93G3         DYVGICRGWDLQGITRRTREVRDKFDALMEIMITAKEE-KRRSQGDDGAETPTKDLLDIL
OsCYP93G2         DFVAVCRGWDLQGIGRRTNEVHARFDALLETIIEAKEEARRRSLRLGRRESSSKDLLDML

                         310       320       330       340       350       360
                  ....|....|....|....|....|....|....|....|....|....|....|....|
ZmCYP93G5(B73)    MDAAEDANAEVRLTRENIKAFVLDIFTAGSDTTATSVEWMLALLINHPACMDKLRAELDA
ZmCYP93G5(W22)    MDAAEDANAEVRLTRENIKAFVLDIFTAGSDTTATSVEWMLALLINHPACMDKLRAELDA
ZmCYP93G15(W22)   MDAAADENAEVRLTRENIKAFVLDIFTAGSDTTATSVEWMLAYLINHPACMDKLRAELDG
SbCYP93G3         MDAAADENAEVRLTRENIKAFVIDIFTAGSDTTATSVEWMLAYLINHPACMDKLRAELDA
OsCYP93G2         MDAAEDDTAEVKLTRDNIKAFVLDIFTAGSDTTATIVEWMLAELVNHPECMAKLRGELDA

                         370       380       390       400       410       420
                  ....|....|....|....|....|....|....|....|....|....|....|....|
ZmCYP93G5(B73)    VVGASRLVGEQDVPRLPYLQAVFKETLRLQPPAVFAQRETIEPVHVRGYVIPPKTSVFFN
ZmCYP93G5(W22)    VVGASRLVGEQDVPRLPYLQAVFKETLRLQPPAVFAQRETIEPVHVRGYVIPSKTSVFFN
ZmCYP93G15(W22)   VVGASRLVGEQDVPHLPYLQAVFKETLRLQPPAVFAQRETVDTVRVRGYVIPPKTSVIFN
SbCYP93G3         VVGTSRLVGENDVPRLPYLQAVFKETLRLQPPAVFSQRETIEPVHVRGYVIPRKTTVFFN
OsCYP93G2         VVGRSRLVGEQDVARLPYLQAVLKETLRLRPPAVFAQRVTVEPVQVRGYTIPTDTQVFFN
```

```
                              430       440       450       460       470       480
                 ....|....|....|....|....|....|....|....|....|....|....|....|
ZmCYP93G5(B73)   IFSIGRDPGCWEDPLQFRPERFMPGGAGAGVDPKGQHMQLMPFGSGRRACPGMGLAMQAV
ZmCYP93G5(W22)   IFSIGRDPGCWEDPLQFRPERFMPGGVGAGVDPKGQHMQLMPFGSGRRACPGMGLAMQAV
ZmCYP93G15(W22)  VFSIGRDPGWWEDPLQFRPERFMPGGAGAGIDPKGQHMQLIPFGSGRRACPGMGLAMQAV
SbCYP93G3        IFSIGRDPAWWEDPLEFRPERFMPGGAGAAVDPKGQHMHFLPFGSGRRACPGMGLAMQAV
OsCYP93G2        IFSIGRDATYWDQPLHFRPDRFLPDGAGATVDPKGQHPQLMPFGSGRRACPGMGLAMQAV

                              490       500       510       520       530       540
                 ....|....|....|....|....|....|....|....|....|....|....|....|
ZmCYP93G5(B73)   PAFLAALVQCFHWEVPIPPGQSTAPPLDMEEAAGLVTARKNHLLLIPTPRLNPLPARAAT
ZmCYP93G5(W22)   PAFLAALVQCFHWEVPIPPGQSTAPPLDMEEAAGLVTARKNHLLLIPTPRLNPLPARAAT
ZmCYP93G15(W22)  PAFLAALVQCFHWAVPIQQGQSKAPPLDMEEAPGLVTARKHPLLLIPTPRLNPLPLQATA
SbCYP93G3        PAFLAALVQCFHWAVPIPQGQSTAPPLDMEEEAGLVTARKHHLVLIPTPRLNPLPVPVPV
OsCYP93G2        PAFLAALVQCFDWAPP--PSQP--LPLDMEEAAGLVSARKHPLLLLPTPRIQPLPSFYS*

                 ....|...
ZmCYP93G5(B73)   *-------
```

Supplemental Figure S11. Amino acid sequence alignment of Poaceae F2Hs belonging to the CYP93G subfamily. The amino acid sequences encoded by *GRMZM2G167336* (*ZmCYP93G5*; *F2H1-B73*), *Zm00004b033614* (*ZmCYP93G5*; F*2H1-W22*), *Zm00004b010826* (*ZmCYP93G15*; *F2H2-W22*), *SbCYP93G3 (Sorghum bicolor;* XM_002461241), and *OsCYP93G2 (Oryza sativa;* AK099468) were aligned using the MUSCLE codon algorithm implemented in the software MEGA7 and visualized with the program BioEdit. Identical amino acids are shaded in black and similar amino acids in grey. F2H2-W22 characterized in this study shares 85%, 84%, 80%, and 69% amino acid identity with F2H1-B73, F2H1-W22, SbCYP93G3, and OsCYP93G2, respectively.

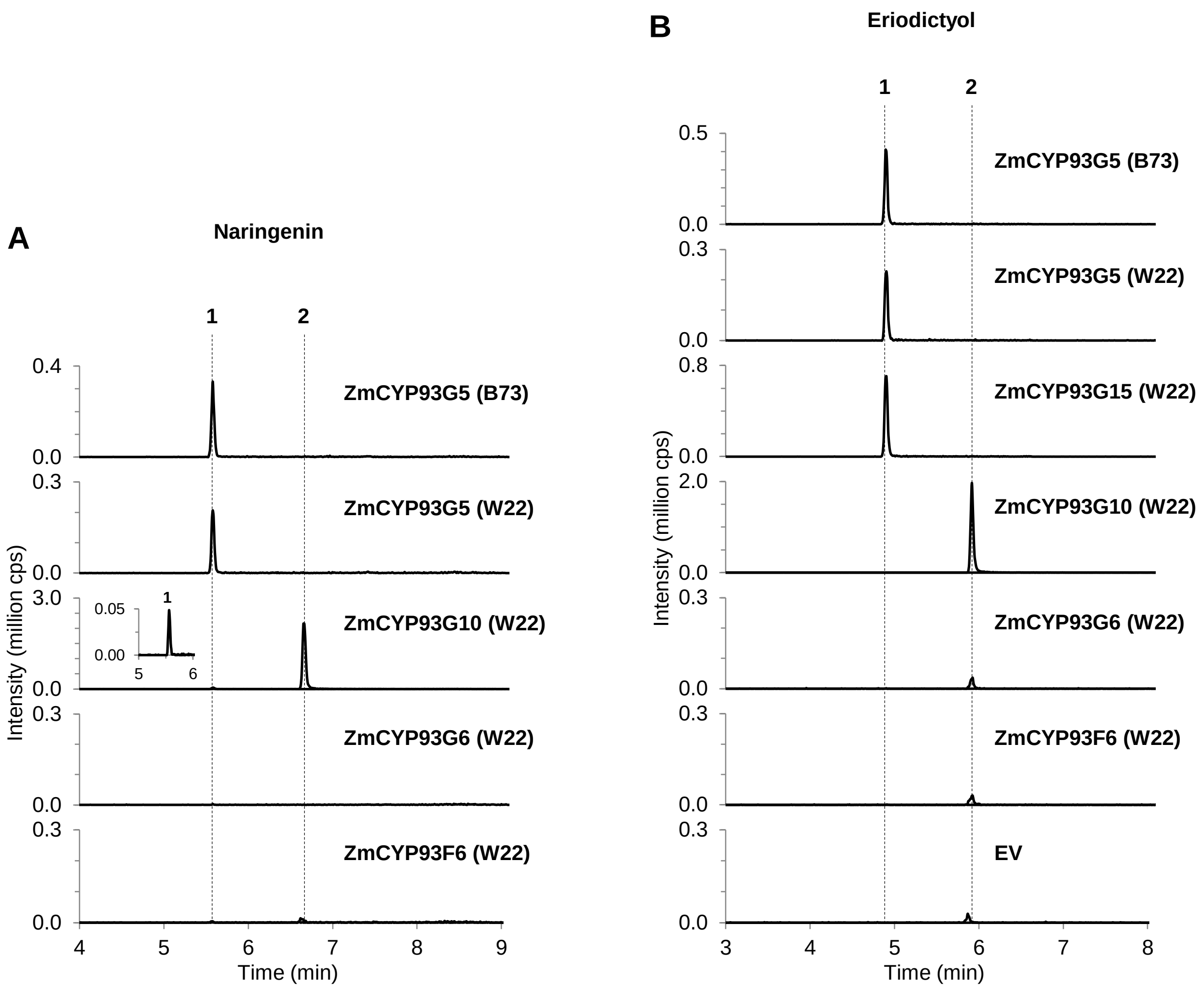

Supplemental Figure S12. Enzymatic activity of CYP93G family members similar to F2H1 (CYP93G5) with naringenin or eriodictyol. The CYP93Gs were heterologously expressed in yeast and the microsomal fraction was incubated with the substrate naringenin **(A)** or eriodictyol **(B)** in presence of the cosubstrate NADPH. Enzyme products were analysed by LC-MS/MS. Numbers in panel A: 1, 2-hydroxynaringenin; 2, apigenin. Numbers in panel B: 1, 2-hydroxyeriodictyol; 2, luteolin. cps, counts per second; EV, empty vector control.

A **B**

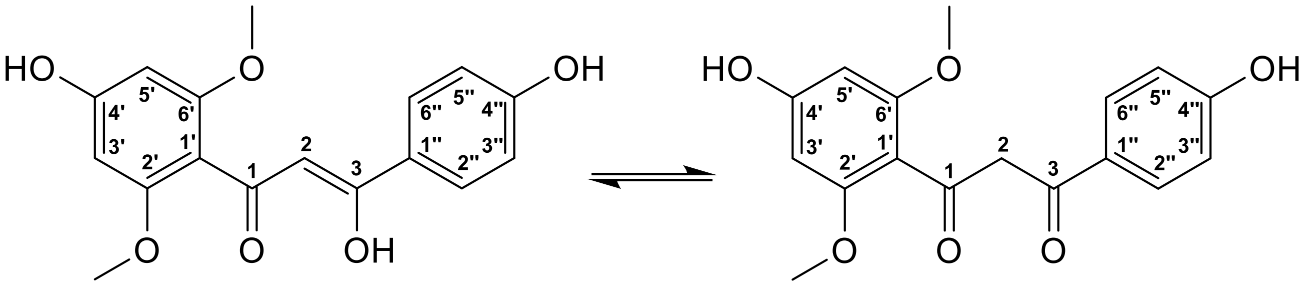

(2Z)-3-hydroxy-1-(4-hydroxy-2,6-dimethoxyphenyl)-3-(4-hydroxyphenyl)prop-2-en-1-one

1-(4-hydroxy-2,6-dimethoxyphenyl)-3-(4-hydroxyphenyl)propane-1,3-dione

pos.	δ_H	*mult.*, J_{HH} [Hz]	δ_C
**1**	-	-	189.5
**2**	6.33	*s*	100.5
**3**	-	-	183.2
**1'**	-	-	109.6
**2'**	-	-	160.3
**3'**	6.13	*s*	92.8
**4'**	-	-	162.2
**5'**	6.13	*s*	92.8
**6'**	-	-	160.3
**1''**	-	-	126.9
**2''**	7.79	*d*, 7.8	130.0
**3''**	6.84	*m*	116.3
**4''**	-	-	162.9
**5''**	6.84	*m*	116.3
**6''**	7.79	*d*, 7.8	130.0
**-OCH$_3$**	3.75	*s*	56.0

pos.	δ_H	*mult.*, J_{HH} [Hz]	δ_C
**1**	-	-	198.4
**2**	4.34	*s*	56.0
**3**	-	-	194.8
**1'**	-	-	111.5
**2'**	-	-	160.3
**3'**	6.07	*s*	92.8
**4'**	-	-	162.9
**5'**	6.07	*s*	92.8
**6'**	-	-	160.3
**1''**	-	-	130.0
**2''**	7.84	*d*, 7.8	132.2
**3''**	6.84	*m*	116.3
**4''**	-	-	163.9
**5''**	6.84	*m*	116.3
**6''**	7.84	*d*, 7.8	132.2
**-OCH$_3$**	3.68	*s*	56.0

Supplemental Figure S13. NMR chemical shift data of xilonenin tautomers (in MeOH-*d*$_3$). The enol form **(A)** and keto form **(B)** occured in a ratio of 2:1 at room temperature. In both tautomers the aromatic rings did not change into a quinoid conformation as is reflected in the chemical shifts; the tautomerism occured only within the positions 2 and 3 of the propanoid moiety connecting the two aromatic rings. Another tautomeric structure could not be identified from the spectral data. The structures have been solved as follows: The methylene group in position 2 of tautomer B showed two long-range C-H correlations with keto functions at position 1 (d_C 198.4) and position 2 (d_C 194.8). The latter showed a long-range C-H correlation with positions 2''/ 6'' (*d*, d_H 7.84/ d_C 132.2) of a 4-hydroxylated aromatic ring. The chemical shifts of positions 3''/ 5'' (d_H 6.84/ d_C 116.3) were determined by COSY/HSQC correlations. Tautomer A showed a similar long-range C-H correlation 2''/ 6'' (*d*, d_H 7.79/ d_C 130.0) → 3 (d_C 183.2). Position 2 in tautomer A was an unsaturated methine (*s*, d_H 6.33/ d_C 100.5) showing long-range C-H correlations with a keto function at position 1 (d_C 189.5) and the unsaturated hydroxyl function at position 3. For both tautomers A and B the connection of the second aromatic ring with the tautomeric 1,3-diketo propane moiety could be proven by a four-bond long-range C-H correlation (3'/5' → 1). This second aromatic ring was symmetrically *O*-substituted as the signal for position 3'/5' appeared as singlet (B: d_H 6.07/ A: d_H 6.13). The position 2'/6' was shown to be methoxylated in both tautomers; a four-bond long-range C-H correlation (-OCH3 →3'/5') determined the structure of the ring.

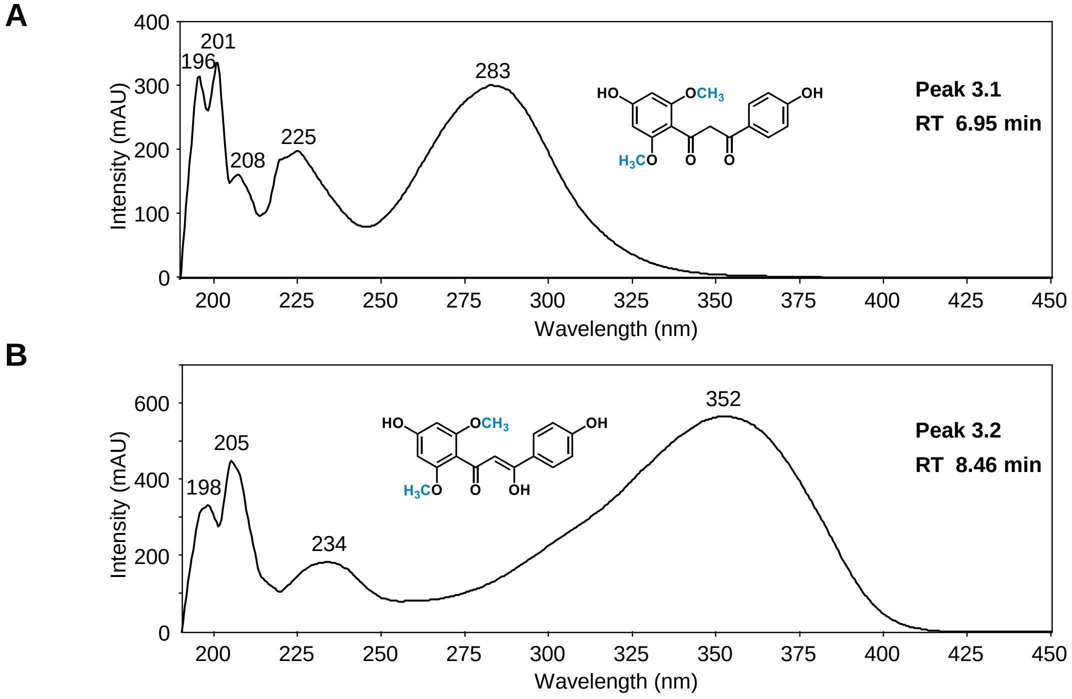

Supplemental Figure S14. The two xilonenin tautomers exhibit different UV absorption. UV spectra of the first **(A)** and second **(B)** peak of xilonenin (compare to Supplemental Figure S9A) were recorded using the purified compound and a LC-MS instrument additonally equipped with an UV detector as described in the methods section. The shift of the UV absorption maximum from 283 nm to 352 nm is indicative for the enol tautomer having a longer conjugated system than the keto tautomer.

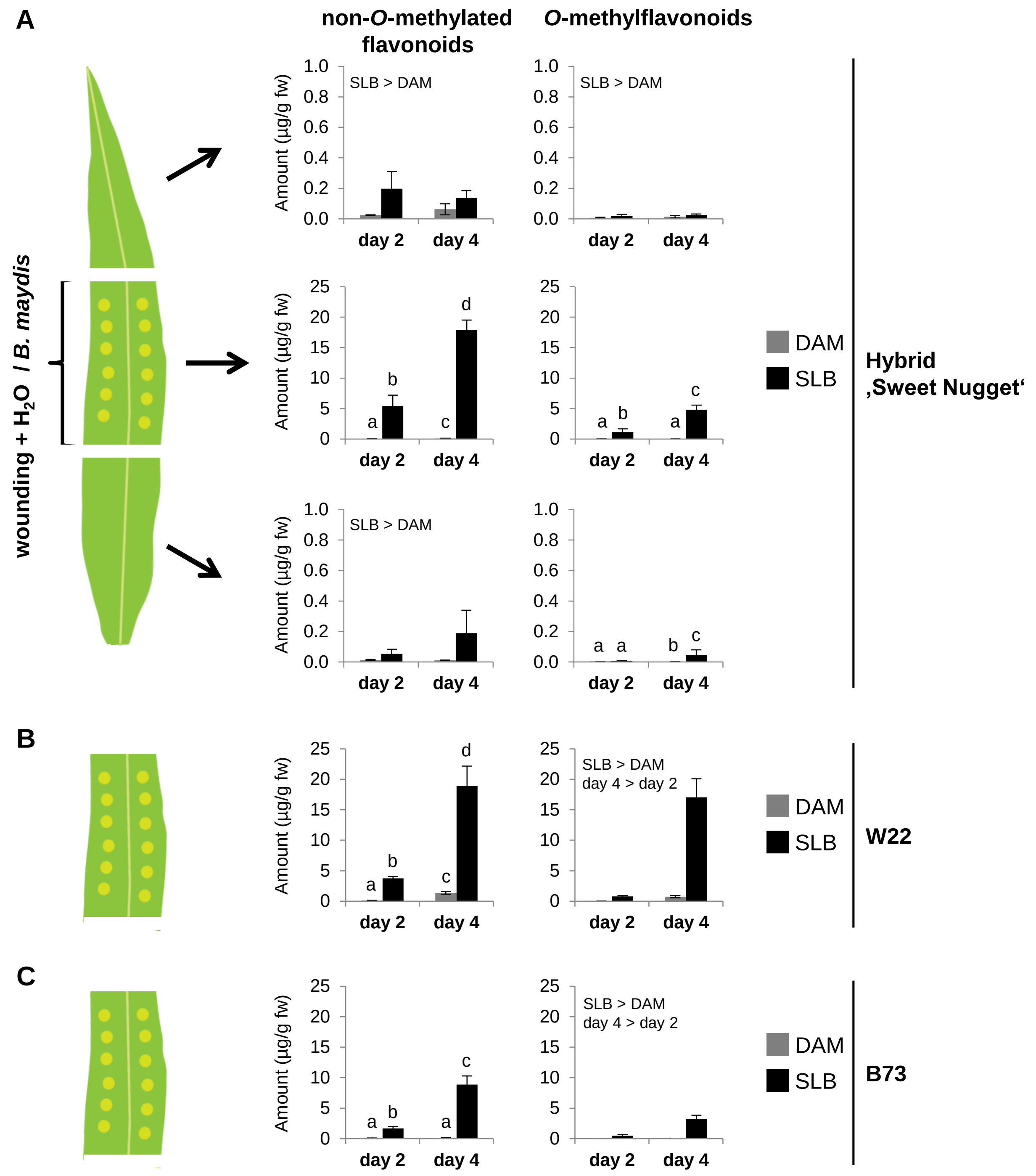

Supplemental Figure S15. De novo production of flavonoids in different maize lines after fungal infection. (A) Spatial distribution of non-*O*-methylated and *O*-methylated flavonoids in ‘upper’, ‘middle’ and ‘lower’ (top down) leaf segments of hybrid maize (‘Sweet Nugget’). The middle leaf segment was damaged and either treated with water as control (DAM) or a mycelial suspension of *B. maydis* (SLB) for 2 or 4 days, and several different non-*O*-methylated and *O*-methylated flavonoids were quantified in the three leaf parts using LC-MS/MS. **(B)** and **(C)** The experiment was repeated with W22 and B73, respectively, with only the middle leaf segment harvested and analyzed. Shown are the total amounts of all analyzed non-*O*-methylated and *O*-methylated flavonoids (left and right panel, respectively; Means ± SE; n = 8). Significant differences for the factors treatment or day are stated. Different letters indicate significant differences between treatments and days (for statistical values, see Supplemental Table S9). Results for individual analytes can be taken from Supplemental Tables S7 and S8.

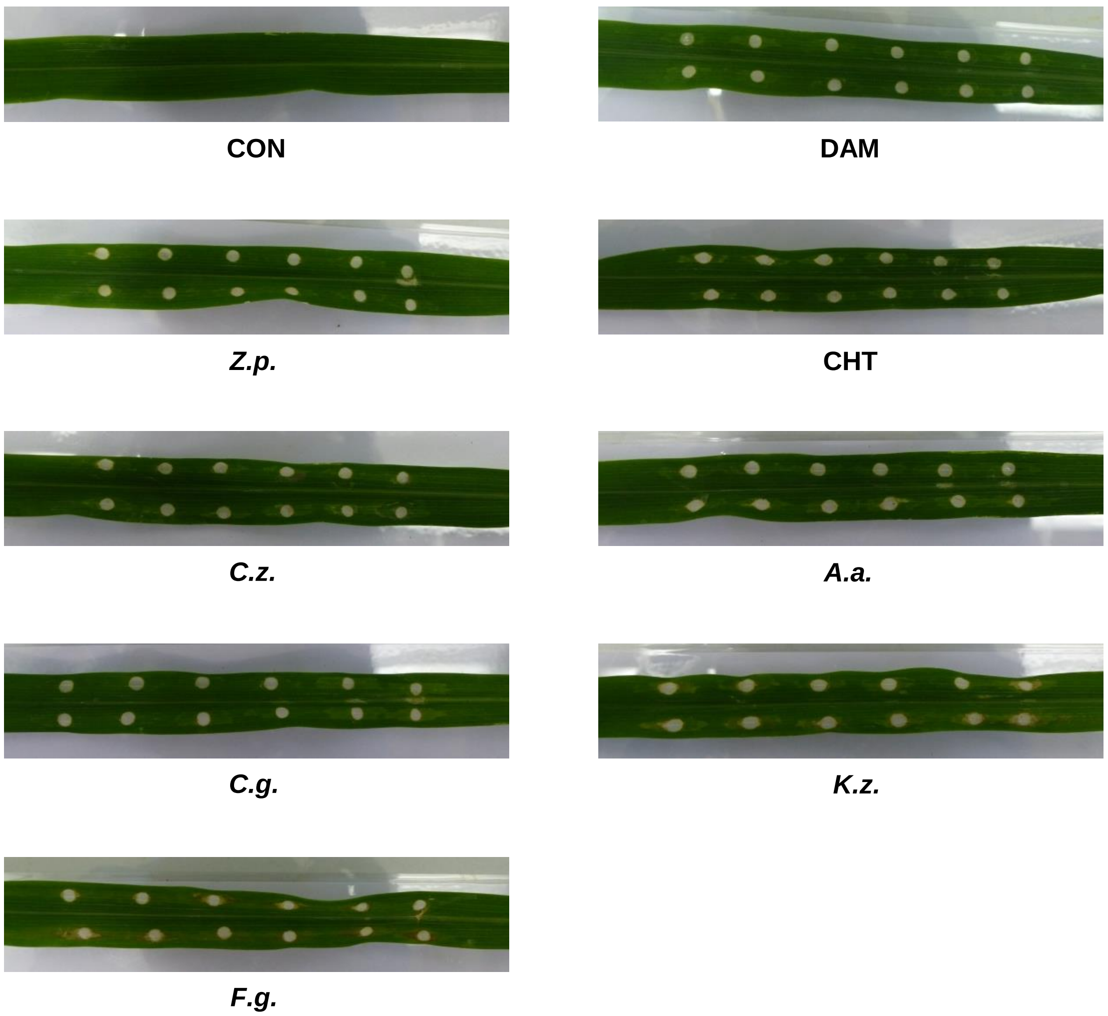

Supplemental Figure S16. Visible signs of infection on hybrid maize after inoculation with different pathogenic fungi. Shown are representative pictures of the following treatments: CON, undamaged; DAM, damaged and treated with water; *Z.p.*, *Zymoseptoria pseudotritici;* CHT, chitosan; *C.z.*, *Cercospora zeae-maydis*; *A.a.*, *Alternaria alternata*; *C.g.*, *Colletotrichum graminicola*; *K.z.*, *Kabatiella zeae; F.g.*, *Fusarium graminearum.*

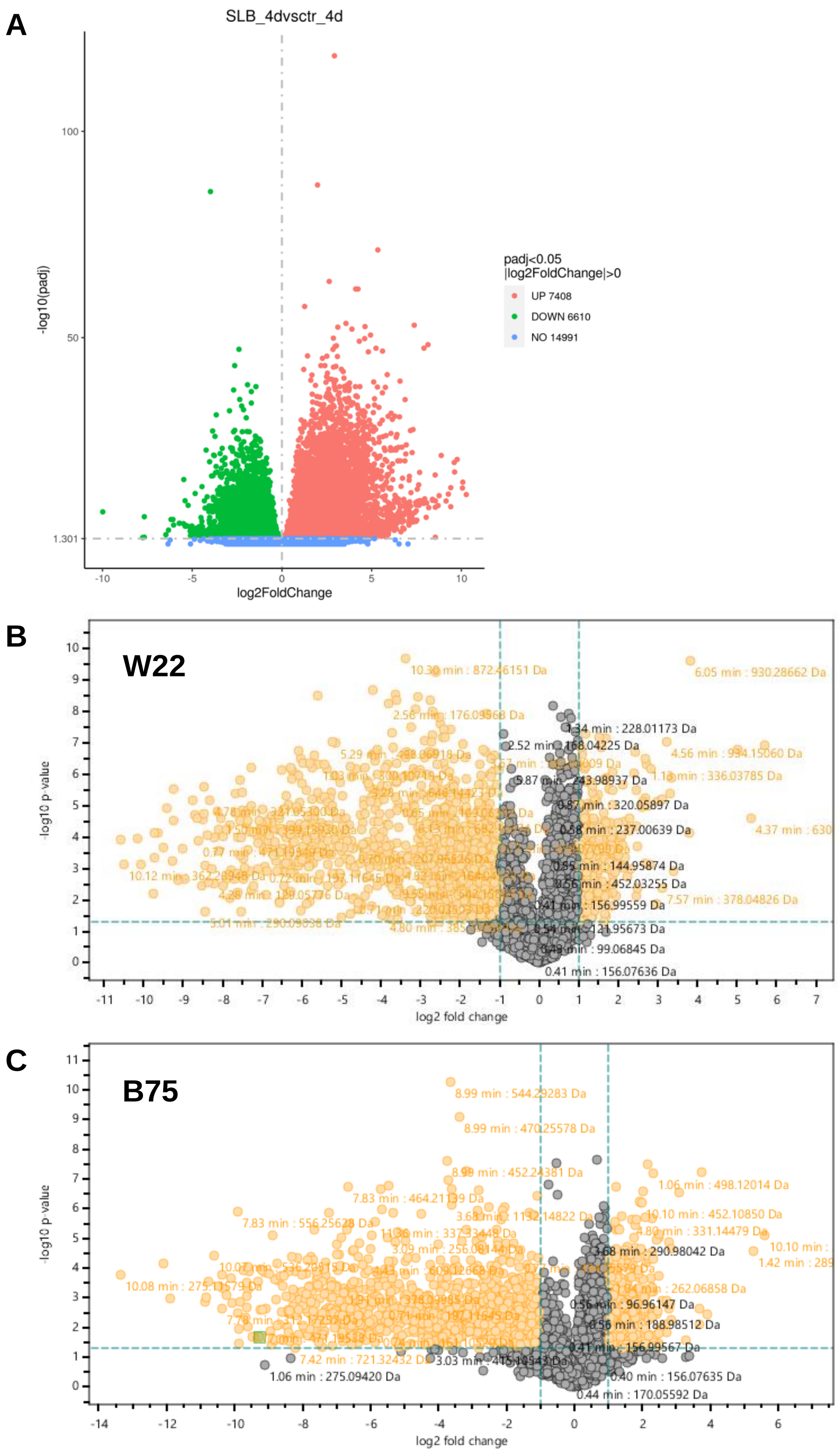

Supplemental Figure S17. Large-scale transcriptomic and metabolomic changes upon SLB-infection. The middle leaf segment of W22 or B75 maize plants was mechanically damaged and either treated with water as control (DAM) or a mycelial suspension of *B. maydis* (SLB) for 4 days. **(A)** Valcano plot based on RNA-seq data (Novogene) using *n* = 4 biological replicates of W22. Upregulated genes (red), downregulated genes (green) and genes that are not differentially expressed (blue) upon SLB-infection are shown. **(B)** and **(C)** Volcano plots based on metabolomic data derived from untargeted LC-MS measurements of *n* = 8 (W22) or *n* = 6 (B75) biological replicates and following analysis using the software MetaboScape 4.0.

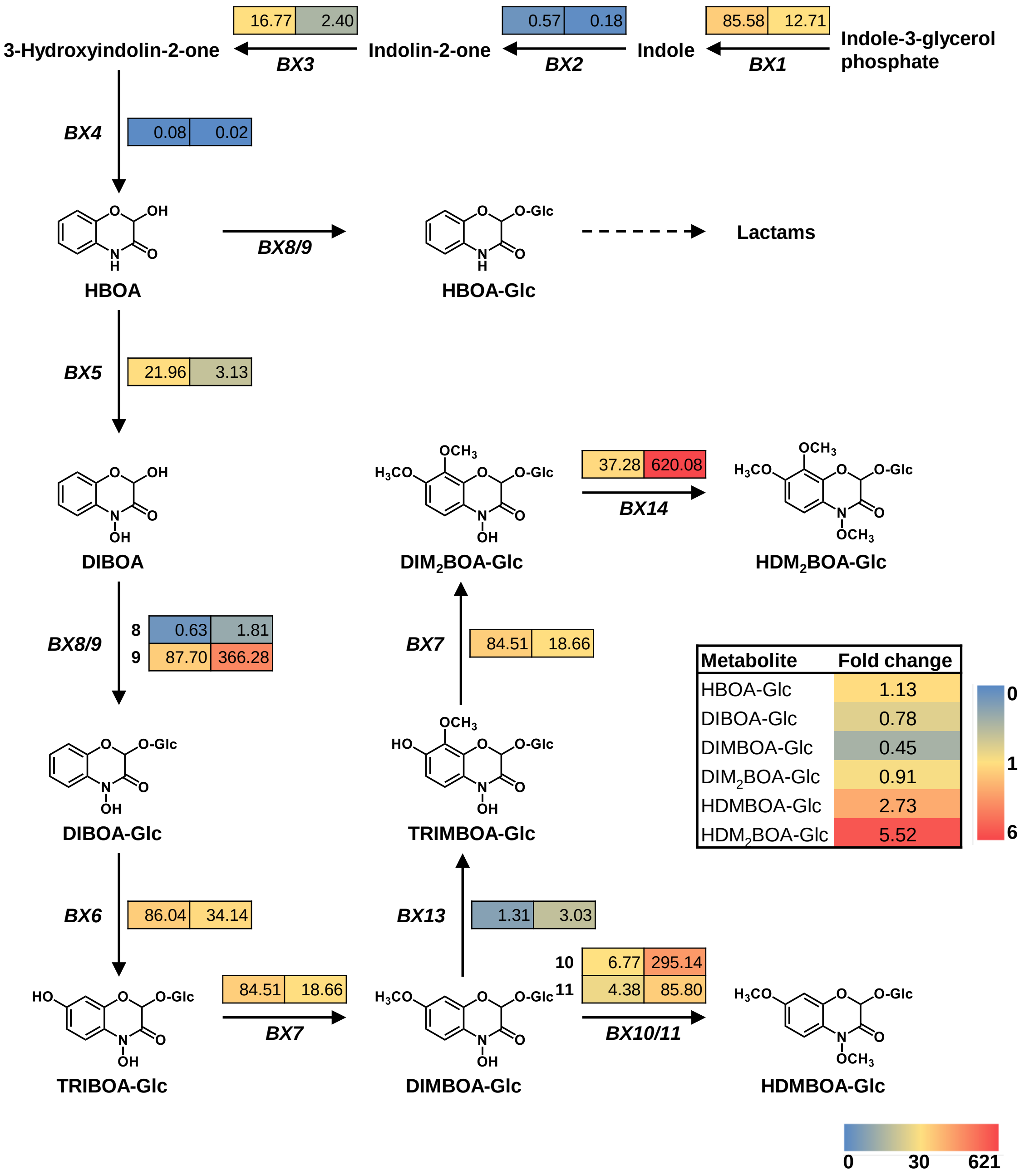

Supplemental Figure S18. Expression of the BX biosynthetic pathway during fungal infection. Expression of genes involved in the BX biosynthetic pathway in damaged and either water treated control (DAM) or *B. maydis*-infected (SLB) W22 leaves after 4 days of treatment. Transcriptomes were sequenced and mapped to the *Zea mays* W22 NRGene V2 genome. RPKM values for each gene are shown as a heat map next to the gene abbreviation (Means; n = 4): DAM (left column) and SLB (right column). For statistics see Supplemental Table S2. The inserted table gives fold changes of corresponding BX metabolites analysed by LC-MS/MS. Relative values can be taken from Supplemental Table S11). HBOA, 2-Hydroxy-3,4-dihydro-2*H*-1,4-benzoxazin-3-one; HBOA-Glc, 2-Hydroxy-3,4-dihydro-2*H*-1,4-benzoxazin-3-one glucoside;

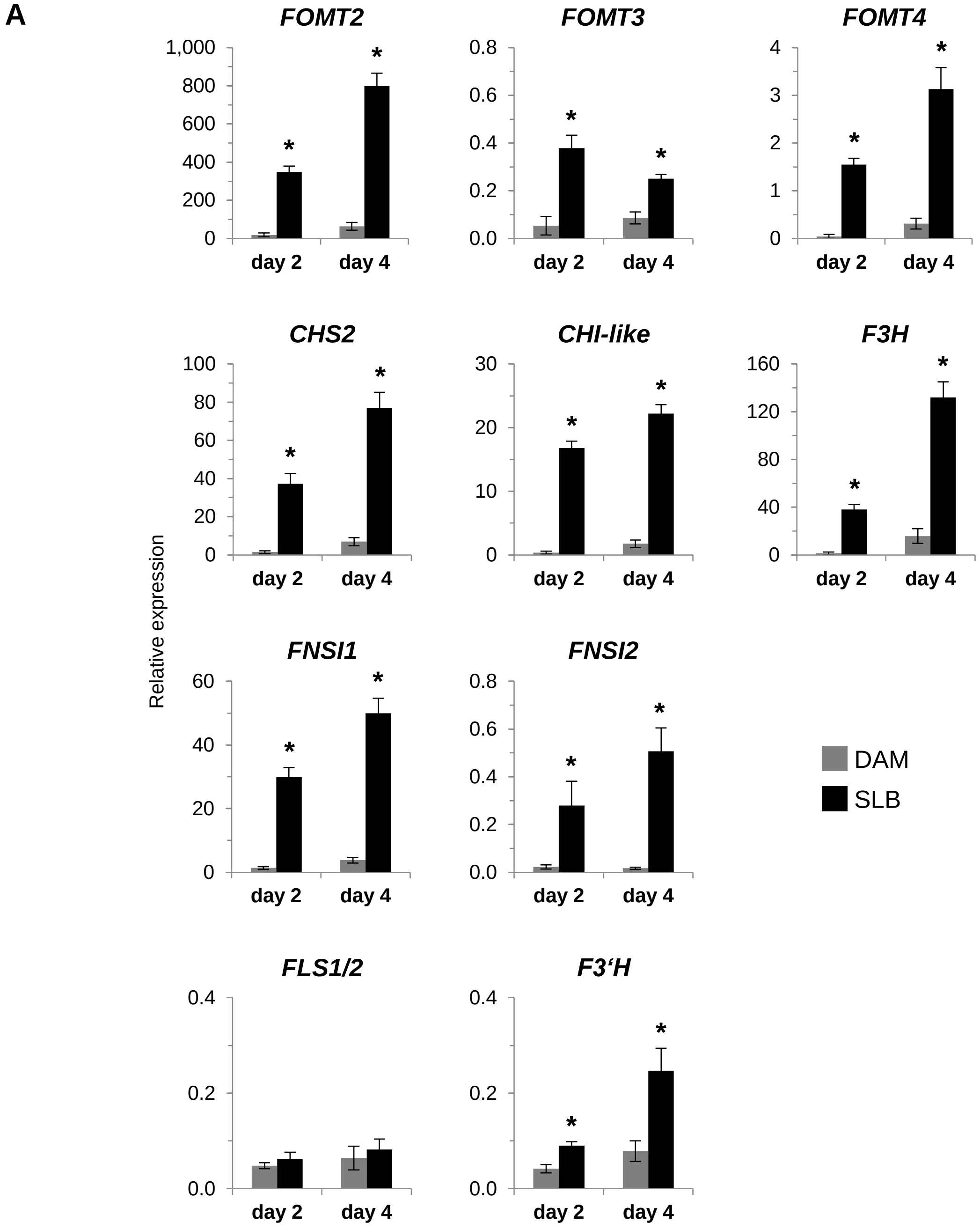

Supplemental Figure S19. RT-qPCR validation of flavonoid and BX pathway gene expression results in noninfected and fungus-infected W22 leaves. Expression of the indicated genes in damaged and either water treated (DAM) or *B. maydis*-infected (SLB) W22 leaves was measured by RT-qPCR relative to UBCP und MEP (Means ± SE; *n* = 4). The relative expression is normalized in a way that values < 1 reflect cq values > 30 and hence rather low expression. Stars indicate statistically significant differences (P < 0.05) between treatments (Student's t-test or Mann-Whitney Rank Sum Test). All gene abbreviations and corresponding IDs are provided in Supplemental Table S2. **(A)** Relative expression of *FOMTs* investigated in this study and flavonoid core pathway genes. *FOMT2*, day 2 (P = 0.029, T = 10); *FOMT2*, day 4 (P = 0.029, T = 10); *FOMT3*, day 2 (P = 0.003, t = -4.937); *FOMT3*, day 4 (P = 0.002, t = -5.267); *FOMT4*, day 2 (P = < 0.001, t = -10.804); *FOMT4*, day 4 (P = < 0.001, t = -6.086); *CHS2*, day 2 (P = 0.029, T = 10); *CHS2*, day 4 (P = < 0.001, t = -8.247); *CHI-like*, day 2 (P = 0.029, T = 10); *CHI-like*, day 4 (P = 0.029, T = 10); *F3H*, day 2 (P = 0.029, T = 10); *F3H*, day 4 (P = <0.001, t = -7.997); *FNSI1*, day 2 (P = <0.001, t = -9.460); *FNSI1*, day 4 (P = < 0.001, t = -9.520); *FNSI2*, day 2 (P = 0.044, t = -2.536); *FNSI2*, day 4 (P = 0.029, T = 10); *FLS1/2*, day 2 (P = 0.407, t = -0,892); *FLS1/2*, day 4 (P = 0.617, t = -0,528); *F3'H*, day 2 (P = 0.008, t = -3.951); *F3'H*, day 4 (P = 0.018, t = -3.246).

B

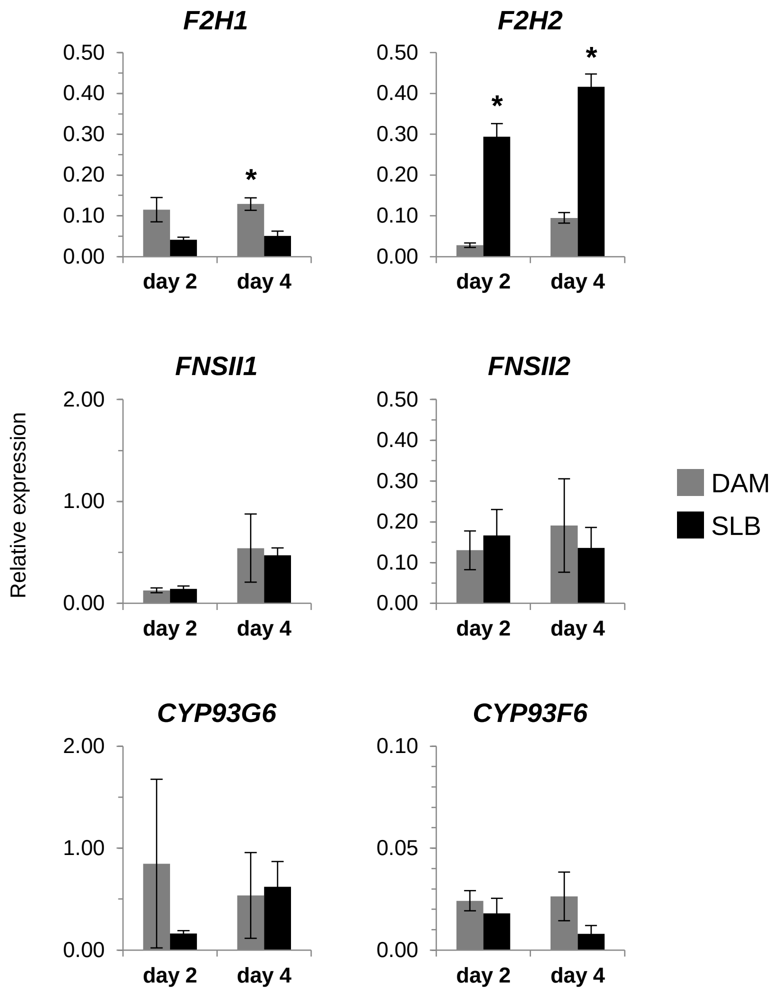

C

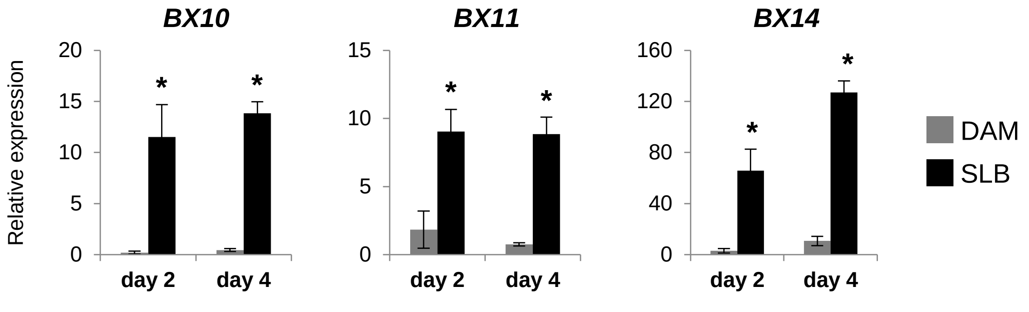

Supplemental Figure S19 continued. (B) Relative expression of *CYP93s* investigated in this study. *F2H1*, day 2 (P = 0.029, T = 10); *F2H1*, day 4 (P = 0.029, T = 10); *F2H2*, day 2 (P = 0.051, t = 2.430); *F2H2* (P = 0.007, t = 4.000); *FNSII1*, day 2 (P = 0.735, t = -0.354); *FNSII1*, day 4 (P = 0.486, T = 15); *FNSII2*, day 2 (P = 0.486, T = 15); *FNSII2*, day 4 (P = 0.686, T = 20); *CYP93G6*, day 2 (P = 0.343, T = 14); *CYP93G6*, day 4 (P = 0.343, T = 14); *CYP93F6*, day 2 (P = 0.517, t = 0,688); *CYP93F6*, day 4 (P = 0.400, T = 9). **(C)** Relative expression of *BX OMTs*. *BX10*, day 2 (P = 0.029, T = 10); *BX10*, day 4 (P = < 0.001, t = -11.636); *BX11*, day 2 (P = 0.014, t = -3.435); *BX11*, day 4 (P = <0,001, t = -6.572); *BX14*, day 2 (P = 0.010, t = -3.718); *BX14*, day 4 (P = <0.001, t = -12.104).

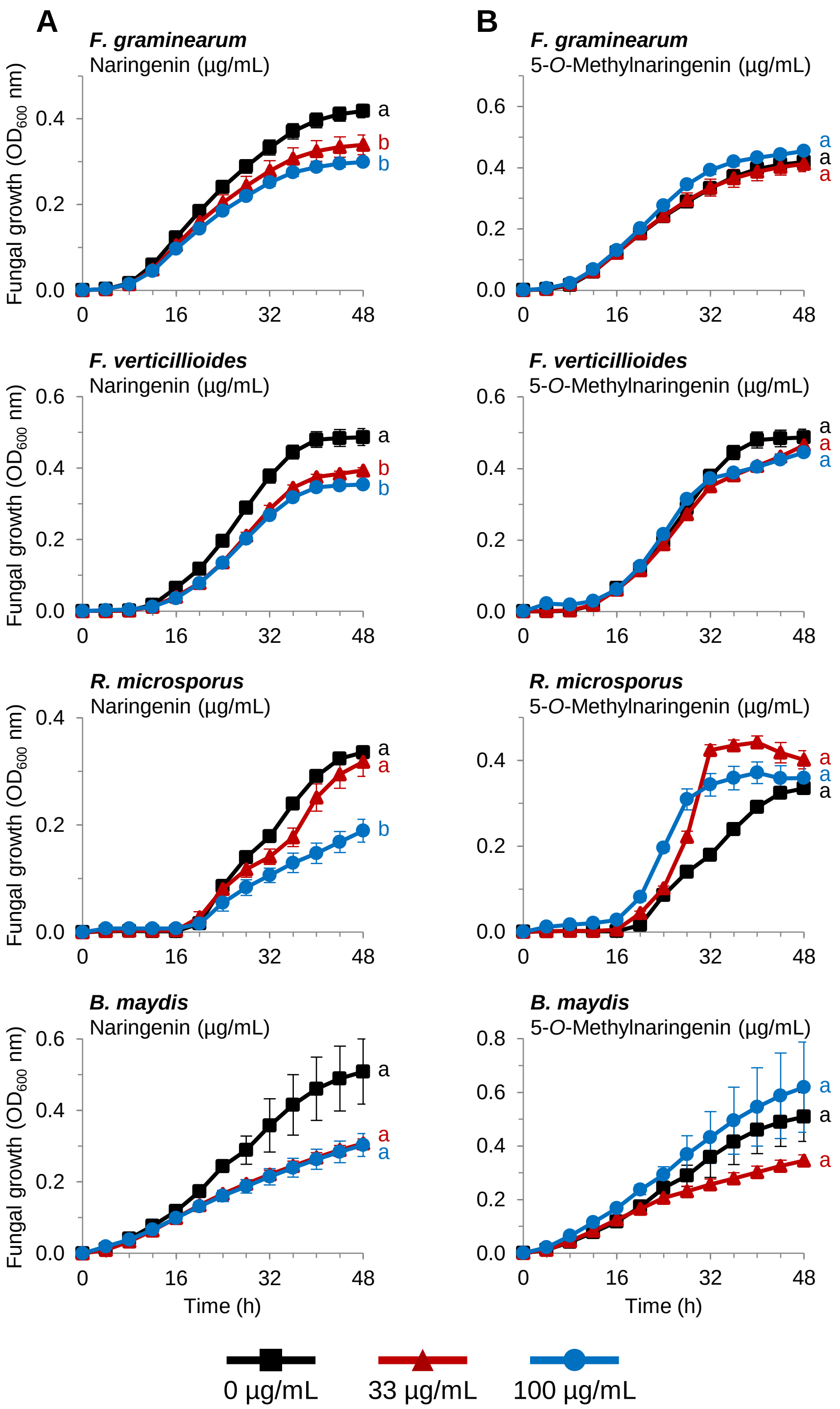

Supplemental Figure S20. Antifungal activity of naringenin and 5-*O*-methylnaringenin. Growth (optical density at 600 nm) of *F. graminearum*, *F. verticillioides*, *R. microsporus* and *B. maydis* in the absence and presence of naringenin **(A)** and purified 5-*O*-methylnaringenin **(B)** measured over a 48-h time course in a defined minimal broth medium using a microtiter plate assay. Data are shown as means ± SE (n = 4). Different letters indicate significant differences (P < 0.05) between treatments at 48 h (one-way ANOVA followed by Tukey-Kramer's post-hoc test or Student's t-test). *F. graminearum*: naringenin (F = 12.948, P = 0.002); 5-*O*-methylnaringenin (F = 1.552, P = 0.264); *F. verticillioides*: naringenin (F = 20.144, P < 0.001); 5-*O*-methylnaringenin (F = 1.923, P = 0.202); *R. microsporus*: naringenin (F = 14.022, P = 0.002); 5-*O*-methylnaringenin (F = 1.901, P = 0.205); *B. maydis*: naringenin (F = 4.350, P = 0.048); 5-*O*-methylnaringenin (F = 1.531, P = 0.268).

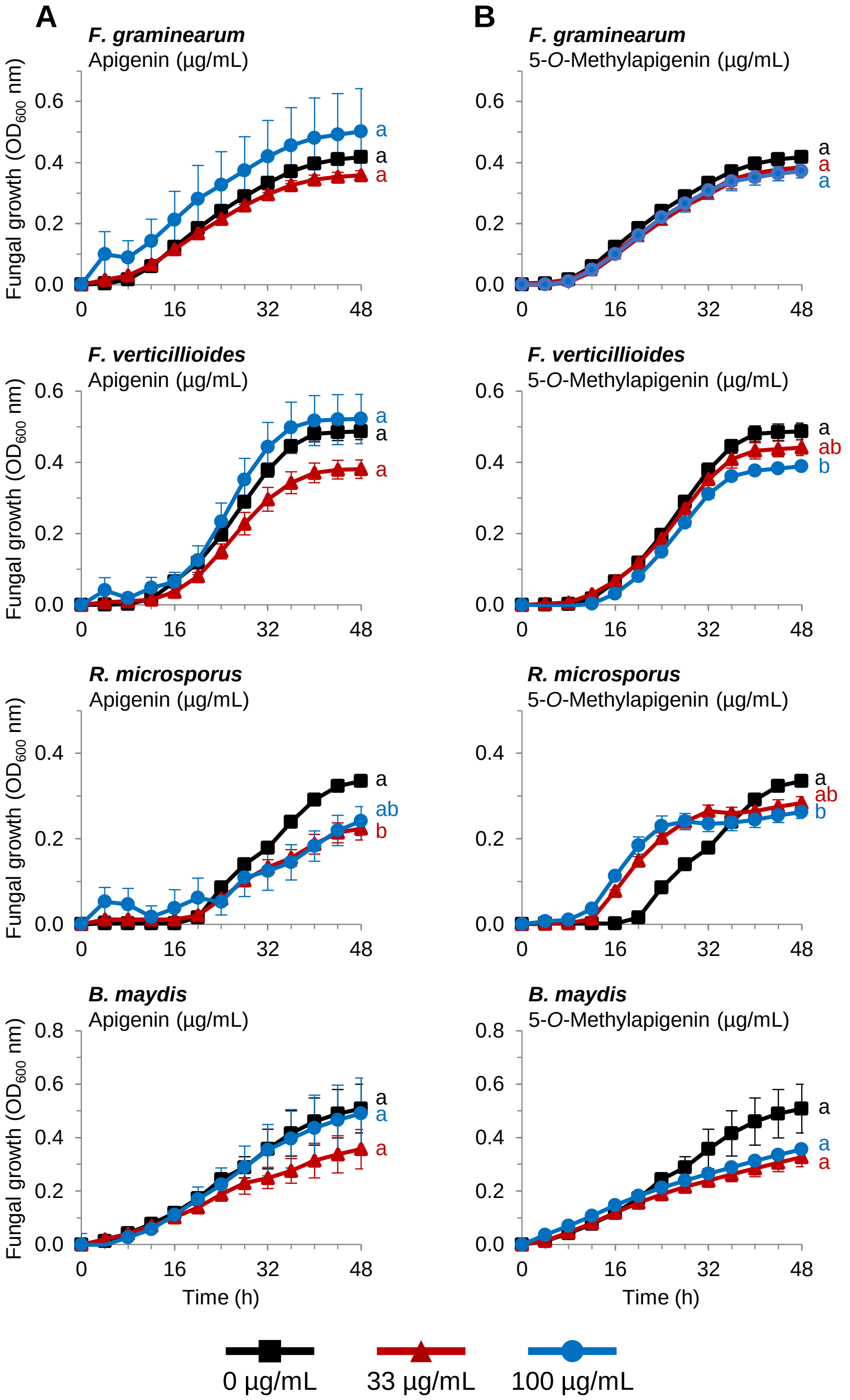

Supplemental Figure S21. Antifungal activity of apigenin and 5-*O*-methylapigenin. Growth (optical density at 600 nm) of *F. graminearum*, *F. verticillioides*, *R. microsporus* and *B. maydis* in the absence and presence of apigenin **(A)** and purified 5-*O*-methylapigenin **(B)** measured over a 48-h time course in a defined minimal broth medium using a microtiter plate assay. Data are shown as means ± SE (n = 4). Different letters indicate significant differences ($P < 0.05$) between treatments at 48 h (one-way ANOVA followed by Tukey-Kramer's post-hoc test). *F. graminearum*: apigenin (F = 0.775, P = 0.489); 5-*O*-methylapigenin (F = 1.634, P = 0.248); *F. verticillioides*: apigenin (F = 2.707, P = 0.120); 5-*O*-methylapigenin (F = 6.781, P = 0.016); *R. microsporus*: apigenin (F = 5.397, P = 0.029); 5-*O*-methylapigenin (F = 7.061, P = 0.014); *B. maydis*: apigenin (F = 0.660, P = 0.540); 5-*O*-methylapigenin (F = 2.914, P = 0.106).

FOMT3-B73 codon optimized

ATGGCGTTTACGGAAGAGAGTTCCCAAGACCTCCTGCAAGCGCACGATGAATTATGGCACCAGAGTGTCAGCTACCTGAAATCGCTCGCCTTAAC
GGTGGCGTTGGATCTGCGCATCCCAGATGCCATTCACCACCATGGTGGCGGTGCCACACTCCTGCAGATTCTTGACAAAACCGCACTGCATCAGA
GCAAATTGCGTGCGTTACGTCGCCTCATGCGTGTTCTTACGGTTAGCGGGACCTTCAGTGTGGTACAACAACCTCCGTGTGGCGACGATGACTCA
ACCGTATATCGCTTAACCGCGGCGAGCCGGTTCTTAGTGAGCGAAGAAGTGTCGTCTGCGACACTGGCTCCTTTCATGTCCGTGGTACTTCACCC
GATTAGCCAGAGCTCCCATGCGCGTGGTATCTGCGCATGGTTTCGCCAAGAGCACCATGATCCGAGTGCATTTGGTCTGGCGTTTGGACAGGCAC
CTACCATTTGGGAACATGCCGATGATACCAATGCCATTCTGAACAAAGGCCTGGCGGCTCAGTCTCGCTTTCTGGTTCCGGTTATGCTGCGCGAA
TGTGGTGAAGCCGTGTTTCGTGGAATTGACTCTCTTGTCGACGTTGGTGGTGGCCATGGCGGAGCCGCAACTGCGATCGCGGCAGCCTTTCCGCA
TCTGAAATGCTCAGTGCTGGATCTGCCACATGTGGTTGCAGGGGCTCCCTCAGATGGGAATGTGCAGTTTGTCGCTGGCGACATGTTTCAGTCGA
TTCCACCGGCAACTGCGGTGTTTCTGAAAACGGCTCTGCATGATTGGGGTGACGATGAATGCGTGAAAATCCTGAAGAACTGCCGCCAAGCCATT
TCGCCGTGTGATGAAGGCGGCAAAGTCATCATCATGGATATGGTTGTTGGCTATGATGAGTCCAACACCAAGCGCTTGGAGGTCCAGATTCTGTT
CGATCTGTTCATCATGATGGTCAATGGTGCCGAACGGGATGAACAGGAATGGAAGAAAATCTTCATTCAGGCTGGCTTCAAAGACTACAAAATTC
TGCCGGTAGTAGGGAGCTTGTCTGTGATTGAGGTCTATCCCTAA

FOMT5-B73 codon optimized

ATGGCCTTACTCGGGGAATACTCCAGCCAAGAGCTGCTGCAAGGGCAACTGCAGCTGTGGCATCAGTCGCTGGGCTTCTTTAAGTCACTGGCCCT
GGCCGTCGCGATGGATCTGCGCATTGCAGATGCGATTCACCGCTTGGGTGGCGCAGCGACGCTTCCGCAGATTCTGGCGGAAGCGGGCATCAAAA
TTGACCCTTGTAACAACAAACTGCGGGACTTACGCCGTGTAATGCGCGCCCTGACCGTGAGCGGGATCTTTACGGTCGTACAACGTCCAGCGAGT
TCGTCCGATGGCGGTGGCCGTCGTGGTGCTGAAGCGGAAGGTCCGGTTTACAAACTGACAGCGGCCTCTCGCTTACTGGTAGTTGGCGAGAAAGA
GTCGTCTACTACCGCCACTACCCGTCAATTGCCGCCGCCGCCTTCTTTAACGGTGTATGTGCAGCTGTTGCTGGAAACCTGTCGCGGCAGTGCGT
TTAGTCGTGCTGTTCGCGAGTGGTTCCAATTCCAACAGCCGCCACAGGACGATGGTCAGCATCAGCAGCCAGCTGGCCGTCTTAGCAGTCCCTTT
GCCTTGGCATGTGGTGGACAGACCATTTGGGAACGTGCAGAACGCGATGCGCACGCATTTCCGTTTGACGATGCCATGGCGTCGGATACCGCGTT
TCTGATGCCGATTGTATTACGGGAATGCGGCGATGAAGTTTTCGGTCGTGGCCTTACCAGCTTGGTGGATGTTGCTGGTGGACTGGGAGGAGCGG
CCGCGACGATTGCAGCTGCTTTTCCCGATCTTAGCTGCACAGTCCTCGATCTGCCCCATGTGGTTGCGAAAGCAGCGGCCGGCACTACGGATAGC
AATGTCCAGTATGTGGCAGGCGACATGTTCCAGTGCATTCCGCCTGCAGATGCCGTCTTACTCAAATGGATTCTCCACGATTGGTCCGACGACGA
GTGTGTGCGTATTCTGAAGAACTGCAAACAGGCTATTCCGCCACGCGCTGCCGGTGGGAAAGTGATTATCATCGACATGGTGGTTGCGGGTCCTG
GCTCAGGGTCCGCAGATGATGAACCGAGCGAAAGCGATCTGCGCCATGTCGAAACCCAGATCTTGTTTGATCTGCTGATGATGTGCGTGAATGGT
GTGGAACGCGACGAACAAGAGTGGAAGAAAATCTTCTCAGAGGCCGGTTTCCAGGACTATCGCATCATGCCGCTCCTGGGCGTTCGCTCTATCAT
CGAACTGTACCCGAATTAA

Supplemental Figure S22. Codon-optimized gene sequences of *FOMT3-B73* and *FOMT5-B73* synthesized for expression in *E. coli*. Synthetic genes were inserted in the pUC57 vector.

F2H1-W22 codon optimized

ATGGAAGCTGATGCTGCTGCTGCAGCTGCTTCTGGTGGTTTGGCTTTGTTGCCAGGTGTTTTGTTGTTGGTTGCTTTGTCTACTTTGGTTTTCTC
TACCTGGTCTAACAGAAACTCTAGATTGCCACCATCTCCAATGGCTTTGCCATTGATTGGTCACTTGCATTTGATTAGACCACCACCACATAGAG
CCTTCGATAGAATTTTGGCTAGATATGGTCCATTGGTCTACTTGAGATTGGGTCCATCTACTCATTGTGTTGTTGCTGGTACTGCAGATGCTGCT
AGAGATTTGTTGAAACATGAAGCCTCTATTCCAGAAAGACCATTGACTGCTGTTACTAGACATTTGGCTTATGATGATGCTGGTTTTGCTTTTGC
TCCATATGGTGCTCATTGGAGGTTTATGAAGAGATTGTGCATGTCCGAATTATTGGGTCCTAGAACTGTTGATCAATTGAGGCCAGTTAGAGAAG
CTGAATTGGCTGCTGTTTTGGAAGCTGCTAGACAAGCTGCTGCCGCTAGAGAACCTATTGATGTTTCAAGACATCTGATCTCCATGTCCAACAAC
GCTATTATGAGAATGGTTGCTTCTGCTTTGCCAGGTCACATGACTGAAGCTGCAAGAGATTGCGCTAAACATGTTGCTGAATTAGTTGGTGCCTT
TAACGTTGAAGATTACGTTGGTTTGTGTAGAGGTTGGGACTTGCAAGGTTTGACTAGAAGAACTAGAGAAGTCAGAGATAAGTTCGATGCCTTGT
TGGAAATTATGATCACCGGTAAAGAAGAGTCTAGAAGAAGAAGGCACGCTACTACTGATACAGGTGGTGGTACAAAAGACTTGTTGGATATATTG
ATGGATGCTGCCGAAGATGCTAATGCTGAAGTTAGATTGACACGTGAAAACATCAAGGCTTTCGTTTTGGATATTTTCACTGCTGGTTCTGATAC
AACTGCTACTTCTGTTGAATGGATGTTGGCCTTGTTGATTAACCATCCAGCTTGTATGGATAAGTTGAGAGCAGAATTAGATGCAGTTGTTGGTG
CATCTAGATTGGTTGGTGAACAAGATGTTCCAAGATTGCCATACTTGCAAGCCGTTTTCAAAGAAACTTTGAGATTACAACCACCAGCTGTTTTC
GCTCAAAGAGAAACAATTGAACCAGTTCACGTTAGAGGTTACGTTATTCCATCTAAGACCTCCGTGTTCTTCAACATTTTCTCCATTGGTAGAGA
TCCAGGTTGTTGGGAAGATCCATTGCAATTCAGACCAGAAAGATTCATGCCAGGTGGTGTTGGTGCCGGTGTTGATCCAAAAGGTCAACATATGC
AATTGATGCCATTTGGTTCTGGTAGAAGGGCTTGTCCAGGTATGGGTTTAGCTATGCAAGCTGTTCCAGCTTTTTTGGCTGCTTTAGTTCAATGT
TTCCATTGGGAAGTTCCAATTCCACCAGGTCAATCTACTGCTCCACCATTGGATATGGAAGAAGCTGCTGGTTTGGTTACTGCTAGAAAGAACCA
CTTGTTGTTGATTCCAACACCAAGATTGAATCCATTGCCAGCTAGAGCTGCTACTTAA

F2H2-W22 codon optimized

ATGGAAGCTGCTGCTGCAGTTACTCCATTGGCTTTGTTGTTGTTGCTTTTGTTGGTTACTAGATGGCGTTGGTCCTCTTCTAGAAATTCTAAATT
GCCACCATCACCAATGGCCTTGCCATTGATTGGTCACTTGCATTTGATTAGAAGATTGCCACACAGATCCTTGGATAGAATTTTGGCTAGATATG
GTCCATTGGTCTACTTGAGATTGGGTCCATCTACTCATTGCATAGTTGCTGGTACTGCTGATGCTGCTAGAGATTTGTTGAAACATGAAGCCTCT
ATTCCACAAAGACCATTGACAGTTGTTGCTAGACATTTGGCTTATGATGATGCTGGTTTTGCTTTTGCTCCATATGGTGCTCATTGGAGATTCAT
GAAGAGATTGTGCATGTCCGAATTATTGGGTCCTAGAACTGTTGATCAATTGAGGCCAGTTAGAGAAGCTGAATTGGCTGCTGTTTTGGGTGCTG
CTGCTTCTGCTTCAGCATCTGGTGAAGGTGAACCTATTGATGTTTCCAGACATCTGATCTCCATGTCCAACAATGCTATTATGAGAATGGTTGCT
TCAGCTTTGCCAGGTCACATGACTGAAGCAGCTAGAGACTGTGCTAAACATGTTGCTGAATTAGTTGGTGCCTTCAACATCGAAGATTACGTTGG
TTTGTGTAGAGGTTGGGACTTGCAAGGTTTGACTAGAAGAACTAGACAAGTCAGAGATAAGTTCGATGCCTTGTTGGAAATGATGATTACCGCCA
AAGAAGAAAAGCGTAGAAGAAGGCAACAAGGTCAAGGTGATCATCATGATTTGCTGGATATTTTGATGGATGCTGCAGCTGACGAAAATGCTGAA
GTTAGATTGACTAGGGAAAACATCAAGGCTTTCGTTTTGGATATCTTCACTGCTGGTTCTGATACAACTGCTACTTCTGTTGAATGGATGTTGGC
CTACTTGATTAACCATCCAGCTTGTATGGATAAGTTGAGAGCAGAATTGGATGGTGTTGTTGGTGCATCTAGATTGGTTGGTGAACAAGATGTTC
CACATTTGCCATACTTGCAAGCCGTTTTCAAAGAAACTTTGAGATTGCAACCACCAGCTGTTTTTGCTCAAAGAGAAACAGTTGATACCGTTAGA
GTTAGAGGTTACGTTATTCCACCAAAAACCTCCGTCATTTTCAACGTTTTCTCCATTGGTAGAGATCCAGGTTGGTGGGAAGATCCATTGCAATT
CAGACCAGAAAGATTCATGCCAGGTGGTGCTGGTGCAGGTATTGATCCAAAAGGTCAACATATGCAATTGATCCCATTTGGTTCTGGTAGAAGGG
CTTGTCCAGGTATGGGTTTAGCTATGCAAGCTGTTCCAGCTTTTTTGGCTGCTTTGGTTCAATGTTTTCATTGGGCTGTTCCAATTCAACAAGGC
CAATCTAAAGCTCCACCATTGGATATGGAAGAAGCTCCAGGTTTGGTTACTGCTAGAAAACATCCTTTGCTGTTGATTCCAACTCCAAGATTGAA
TCCATTGCCATTGCAAGCTACAGCTACTTGA

FNSII2-W22 codon optimized

ATGAAGGAACAACAACCTAGACCAAGACCATCCATTATGTTCGTTTTATCCTCTTTGGCTAAGAACAACCCAGAAGCTGTTTTGGCTTTGATTGC
TGTTGTTACTGTTGTTGCCTTGAGACACTTGATTTCATCTTGGAGACAACAAGCTCCATTGCCACCATCTCCAACATCTTTGCCAGTTATTGGTC
ACTTGCATTTGTTAAGACCACCAGTTCATAGAACCTTCCAAGAATTGGCTTCTAGAATTGGTCCATTGATGCATATCAGATTGGGTTCTACTCAT
TGCGTTGTTGCATCTTCTCCAGAAGTTGCTTCTGAATTGATTAGAGGTCATGAGGGTTCCATTTCTGAAAGACCATTGACTGCTGTTGCTAGACA
ATTTGCTTATGATTCTGCTGGTTTTGCTTTCGCTCCATACAATACTCATTGGAGATTCATGAAGAGGTTGTGCATGTCTGAATTATTGGGTCCAA
GAACCGTTGAACAACTAAGACCAATTAGACGTGCTGGTACTGTTTCTTTGTTGGGTGATTTGTTGGCTTCTTCTGCTAGAGGTGAAACTGTTGAT
TTGACCAGACATTTGATCAGGTTGTCCAACACCTCCATTATTAGAATGGTTGCTTCTACTGTTCCAGGTTCTGTTACTGATGAAGCTCAAAAGGT
TGTTAAGGATGTTGCTGAATTGGTTGGTGCCTTTAACGTTGATGATTACATTGCAGTTGTTAGAGGTTGGGACTTGCAAGGTTTAAGACGTAGAG
CTGCTGATGTCCATAGAAGATTTGATGCTTTGTTGGAGGACATCTTGAGGCACAAAGAAGAAGCTAGAGCAGCTAGAAGATTGGATCAAGATGAT
GGTCAAGGTATCTCTTCCAAGCAAGATAAGAAACAAGCTACCCACTCTAAGGACTTGTTGGATATTTTGATGGATAAGGCTGAAGATCAAGCTGC
CGAAGTTAAGTTGACTAGAGAAAACATTAAGGCCTTCATCATCGATGTTGTTACAGCTGGTTCTGATACTTCTGCTGCTATGGTTGAATGGATGT
TGGCAGAATTGATGAACCATCAAGAAACCTTGAGAAAGGTCGTTGAAGAAATTGATGCCGTTGTTGGTGGTGATAGAATTGCTAGTGAAGCTGAT
TTGCCAAGATTGCCATATTTGATGGCTGCTTACAAAGAGACTTTGAGATTGCATCCAGCTGCTCCAATTGCTCATAGACAATCTTCAGAAGAAAT
GGTCGTCAGAGGTTTTACAGTTCCACCACAAACTGCTGTTTTCATTAACGTTTGGGCTATTGGTAGAGATCCAGCTTATTGGGAAGAACCATTGG
CTTTTAGACCAGAAAGATTCATGCCAGGTGGTGCTGCTGAATCTTTGGAACCTAGAGGTCAACATTTTCAGTACATGCCATTTGGTTCTGGTAGA
AGAGGATGTCCAGGTATGGGTTTAGCTTTACAATCTGTTCCAGCTGTTTTAGCCGCTTTGGTTCAATGTTTCCATTGGGCTACTGTTGATGGTGA
TGGTGGTGTAAACAAGATCGATATGTCAGAATCTGATGGTTTGGTTTGCGCTAGAAAAAAGCCTTTGCTATTAAGACCAACTCCAAGGTTGACTC
CATTTCCAGCAGTTGTTTAA

Zm00004b039147 codon optimized

ATGGAAGAACAACAATTGAGAGCCAGACCAAATATGATGGTCTTATCTTCTTTGGCCAAGAACAATCCAGAAGCTGTTTTGGCTTTGATTGCTTT
CGTTACTGTTGTTGCCTTGAGACACTTGATTTCATCTTGGAGACAACATGGTAGATTGCCACCAGGTCCAACATCTTTGCCAGTTATTGGTCACT
TGCATTTGTTAAGACCACCAGTTCATAGAACCTTGCAAGAATTGGCTTCTAGAATTGGTCCATTGATGCATATCAGATTGGGTTCTACCAATTGC
GTTGTTGCATCTTCTCCAGAAGTTGCCTCTGAATTGATTAGAGGTCATGAAGGTTCAATTTCCGCTAGACCTTTTACTGCTGTTGCTAGAAAGTT
CTCTTATGATTCTGCTGGTTTCGTGTTCGAACCATACAATACTCATTGGAGATTCATGAAGAGGTTGTGCATGTCTGAATTATTGGGTCCAAGAA
CCGTTGAACAACTAAGACCAGTTAGAAGGGCTGTTACTGTTTCTTTGGTTTCTGATTTGTTGGCTTCTTCTGCTAGAGGTGAAACTGTTGATATT
ACCAGACATTTGATCAGGTTGACCAACACCTCCATTATTAGAATGGTTGCTTCTACCGTTTCTGGTTCTGTTACTGATGAAGCTCATGAATTGGC
TAAGGCCGTTATTGAAGTTGTTGGTGCTTTTAACGTTGACGATTACATTGCTGTTGTTAGAGGTTGGGATTTTCAAGGTTTGGGTAGAAAAGCTG
CTGATGTCCATAGAAGATTTGATGCCTTGTTGGAGGATATCTTGAGGCACAAAGAAGAAGCTAGAGCTGCTAGAAGATTGGACGATGGTCATGGT
AAACAAGCTACTCATTCTAAGGACTTGTTGGACATCTTGATGGATAAGGCTGAAGATCCAGCTGCTGAAGTTAAGTTGACTAGAGAAAACATTAA
GGCCTTCGTTATCGATGTTGTTACCTCTGGTTCAGATACTTCTGCTGCTATGGCTGAATGGATGTTGGCAGAATTGATGAATCATCCAGAAACCT
TGAGAAAGGTCGTTGAAGAAATTGATGCTGTTGTAGGTGGTGGTAGAATTGCTTCTGAAGCTGATTTGCCACAATTGCCATATTTGATGGCCGTT
TACAAAGAGACTTTGAGATTGCATCCAGCTGGTCCAATTGCTCATAGACAATCTACTGAAGAAATGGTTGTTCATGGTTTCACTGTTCCACCACA
ATCCACTGTTTTGATTCATGTTTGGGCTATTGGTAGAGATCCAGCATATTGGGAAGAACCTTTGTTGTTTAGACCAGAAAGATTCATGCCAGGTG
GTGCTGCTGAATCTTTGGAGCCAAGAGGTAAACATTTTCAGTACATTCCATTCGGTTCTGGTAGAAGAGGATGTCCAGGTATGGGTTTAGCTATG
CAATCTGTTCCAGCAGTTGTTGCTGCATTGGTTCAATGTTTTCATTGGTCTACTGTTGATGGTGGTATGGATAAGATCGACATGTCAGAATCTGA
TGGTTTGGTTTGCGCTAGAAAAAAGCCTTTGCTATTAAGACCAACCTCCAGATTGACTCCATTTCCACCTGTTGTTTGA

Zm00004b033036 codon optimized

ATGGAAGTTGTTACCGCTAGGGAATTGATTAACCCAACTGGTTTGCCAATCTTGTTGTTGGTTGCTGGTTTGACTGTTTTCTACGTTTTGCGTAG
AAGATCATCTGGTGGTTTGAGATTGCCACCATCTCCATTTGCTTTGCCAGTTTTGGGTCACTTGCATTTGTTGGCTCCATTGCCACATCAAGCCT
TGCATAGATTGGCTGCTAGACATGGTCCTTTGTTGTATTTGAGATTAGGTTCCATGCCAGCTATTGCTGCTTGTTCTCCAGATGCTGCTAGAGAA
GTTTTGAAAACTCATGAAGCTGCTTTCTTGGATAGACCAAAACCTACTGCTGTTCATAGATTGACTTATGGTGGTCAAGACTTCTCTTTTTCTCC
ATATGGTCCTTATTGGAGGTTTATGAAGAGGGCTTGTGTTCATGAATTATTGGCTGGTAGAACCTTGGAAAGATTGAGACATGTTAGAAGGGAAG
AAGTCTCTAGATTGGTTGGTTCTTTGTCTAGATCTGCTGGTGATGGTGCTGCTGTTGATGTTGATGCAGTTTTGATGGGTGTTACCGGTGATATT
ATCTCCAGAATGGTTATGTCTAGAAGATGGACTGGTGATGATTCTACTACCGAAGAAATGAGATCTTTGGTTGCAGAAACTGCTGAATTGACTGG
TACTTTCAACTTGCAAGATTACATCGGTATGTTCAAGCACTGGGATGTTCAAGGTTTGGGTAAAAGAATTGATGCCGTTCACAGAAAGTTCGATG
CTATGATGGAAAGAATTTTGACCGCTAGAGATGCTGAAAGAAGGTGTAGAAGAAAAGGTGCTGCAGATGGTGCCGGTGAAGGTGATAAGAAAGAT
TTGTTGGATATGCTGTTCGATATGCACGAAGATGAAGGTGCTGAAATGAGACTAACTAGAGATAACATTAAGGCCTTCATGCTGGATATTTTTGC
TGCTGGTACTGATACCACTACCATTACTTTGGAATGGGCTTTGTCTGAGTTGATTAACAATCCAGCTGTTTTGAGAAGGGCTCAAGCTGAATTGG
ATGCAGCTGTTGGTGCTTCTAGATTAGCTGATGAATCTGATATTCCACGTTTGCCATACTTGCAAGCTATTGCCAAAGAAACTTTAAGATTGCAT
CCAACCGGTCCATTGGTTGTTAGAAGATCTATGGCTCCATGTAACGTTTCTGGTTATGATGTTCCAGCTGGTGCTACTGTTTTTGTTAATGTTTG
GGCTATTGGTAGAGATCCAGCTTGTTGGGCTCCAGATCCATTGGCTTTTAGACCTGAAAGATTCTTGGAAGAGGAAGGTGGCGGAGAATCAGCTG
GTTTGGATGTTAGAGGTCAACATTTTCATTTGTTGCCATTCGGTTCCGGTAGAAGAATTTGTCCAGGTGCTTCTTTGGCTATGTTGGTTGTTCAA
GCTGCATTAGCTGCTATGTTGCAATGTTTTGAATGGACTCCAGTTGGTGGTGCTCCAGTTGATATGGAAGAAGGTCCAGGTTTGACTTTGCCAAG
AAAAAGACCATTGGTCTGTACTGTTAAGGCTAGATTGCACCCATTGCCAGTTCCAGCCGCTGCTGCTGATAATGGTGTTGAAGAAACCGCTGGTG
TTTGA

Supplemental Figure S23. Codon-optimized gene sequences of CYP93G candidates synthesized for expression in *S. cerevisiae.* Synthetic genes were inserted in the pMA-T vector.
