# Supplementary material for: Biosynthesis and antifungal activity of fungus-induced O-methylated flavonoids in maize
Source: Plant Physiol. 2021 Oct 27;188(1):167–90. doi: 10.1093/plphys/kiab496 (PMC8774720; doi:10.1093/plphys/kiab496)
Supplement: kiab496_Supplementary_Data [file kiab496_supplementary_data.zip › kiab496-suppl_data/2021-10-29_supplemental_tables_part1.pdf]

Supplemental Table S1. *P*-values of *t*-test analysis to determine statistical significant differences of flavonoid content between treatments obtained by the LC-MS measurements shown in Supplemental Figure 1B. T-test was performed as implemented in the MetaboScape 4.0 software (Bruker Daltonics). na, not available.

**Compound**	**RT (min)**	***m/z* quan. ion**	**P-value (Rank) B75**	**P-value (Rank) W22**
Apigenin*	8.25	271.06012 $[M+H]^+$	0.003948	0.000778
Naringenin chalcone*	8.12	273.07572 $[M+H]^+$	0.010406	0.000778
Naringenin*	8.31	273.07558 $[M+H]^+$	0.003948	0.000778
5-*O*-Methylapigenin**	6.24	285.07584 $[M+H]^+$	0.003948	0.000778
Genkwanin*	10.61	285.07580 $[M+H]^+$	0.003948	na
Luteolin*	7.35	287.05502 $[M+H]^+$	0.003948	0.000778
Flavonoid aglucone	4.83	287.09140 $[M+H]^+$	0.003948	0.000778
5-*O*-Methylnaringenin*	6.35	287.09156 $[M+H]^+$	0.003948	0.000778
Flavonoid aglucone	7.24	287.09141 $[M+H]^+$	0.003948	0.000778
Flavonoid aglucone	9.25	287.09153 $[M+H]^+$	0.003948	0.000778
Dihydrokaempferol*	6.53	287.05618 $[M-H]^-$	0.003948	0.000778
2-OH-Naringenin**	7.00	289.07083 $[M+H]^+$	0.003948	0.000778
5,7-*O*-Dimethylapigenin**	7.88	299.09161 $[M+H]^+$	0.003948	na
5-*O*-Methylluteolin***	5.65	301.07103 $[M+H]^+$	0.003948	0.000778
5-*O*-Methylscutellarein**	5.92	301.07112 $[M+H]^+$	0.003948	0.000778
7-*O*-Methylscutellarein**	7.51	301.07120 $[M+H]^+$	0.003948	0.001131
Flavonoid aglucone	8.42	301.07108 $[M+H]^+$	0.003948	0.000778
Flavonoid aglucone	4.18	303.08649 $[M+H]^+$	0.003948	0.002322
5-*O*-Methyldihydrokaempferol***	5.67	303.08633 $[M+H]^+$	0.003948	0.000778
O-Methyl-2-OH-naringenin***	8.03	303.08647 $[M+H]^+$	0.003948	0.000778
O-Methyl-2-OH-naringenin***	10.24	303.08649 $[M+H]^+$	0.003948	0.000778
Taxifolin*	5.69	303.05109 $[M-H]^-$	0.003948	0.000778
Flavonoid aglucone	6.15	315.08639 $[M+H]^+$	0.003948	na
Flavonoid aglucone	6.39	315.08656 $[M+H]^+$	0.003948	0.000778
5,7-*O*-Dimethylscutellarein**	6.64	315.08660 $[M+H]^+$	0.003948	na
O-Dimethylluteolin***	10.84	315.08618 $[M+H]^+$	0.003948	na
Xilonenin tautomer 1**	6.95	317.10205 $[M+H]^+$	0.003948	0.000778
Xilonenin tautomer 2**	8.46	317.10197 $[M+H]^+$	0.003948	0.000778
5-*O*-Methyltaxifolin***	4.96	319.08117 $[M+H]^+$	0.003948	0.001131
Flavonoid aglucone	6.12	331.08100 $[M+H]^+$	0.003948	na
Flavonoid aglucone	6.20	331.08124 $[M+H]^+$	na	0.001131
Flavonoid aglucone	7.58	329.06674 $[M-H]^-$	na	0.000778
5,7-*O*-Dimethylquercetin***	7.70	331.08139 $[M+H]^+$	0.003948	na
Flavonoid aglucone	7.46	333.09688 $[M+H]^+$	0.003948	na
Flavonoid aglucone	7.59	331.08207 $[M-H]^-$	na	0.000778
Flavonoid aglucone	4.39	337.05555 $[M+H]^+$	0.003948	0.000778
Flavonoid aglucone	6.80	345.09700 $[M+H]^+$	0.003948	0.000778
Flavonoid aglucone	10.67	345.09713 $[M+H]^+$	0.003948	na

Supplemental Table S2: see Supplemental Tables (Excel file)

Supplemental Table S3. MaizeGDB/GenBank accessions and references corresponding to Figure 2D and Supplemental Figure S6.

Name	Plant species	MaizeGDB/GenBank accession	Reference
ZmBX10	*Zea mays*	Zm00001d029359	Meihls et al., 2013
ZmBX11	*Zea mays*	Zm00001d029356	
ZmBX12	*Zea mays*	Zm00025ab019610	
ZmBX14	*Zea mays*	Zm00001d004921	
ZmBX7	*Zea mays*	Zm00001d049179	Jonczyk et al., 2008
ZmOMT1 (ZmFOMT1, ZmCOMT1)	*Zea mays*	Zm00001d049541; EF586877 / M73235	Zhou et al., 2008; Collazo et al., 1992
ZmFOMT2	*Zea mays*	Zm00001d047192	this work
ZmFOMT3	*Zea mays*	Zm00001d047194	this work
ZmFOMT4	*Zea mays*	Zm00001d048087	this work
ZmFOMT5	*Zea mays*	Zm00001d051934	this work
ZmAAMT1	*Zea mays*	HM242244	Köllner et al., 2010
ZmAAMT2	*Zea mays*	HM242246	Köllner et al., 2010
ZmAAMT3	*Zea mays*	HM242247	Köllner et al., 2010
ZmOMT8	*Zea mays*	HM242248	Köllner et al., 2010
ZmZRP4	*Zea mays*	Zm00001d049020	/
ZmCCoAOMT1	*Zea mays*	Zm00001d036293	Li et al., 2013
ZmCCoAOMT2	*Zea mays*	Zm00001d045206	Wang et al., 2016
HvF1-OMT	*Hordeum vulgare*	X77467	Christensen et al., 1998
HvOMT1	*Hordeum vulgare*	EF586876	Zhou et al., 2008
TaOMT2	*Triticum aestivum*	DQ223971	Zhou et al., 2006 (2)
OsNOMT	*Oryza sativa*	AB692949	Shimizu et al., 2012
OsROMT9	*Oryza sativa*	DQ288259	Kim et al., 2006
Ms7IOMT	*Medicago sativa*	AF000976	He et al., 1998
MsI2'OMT	*Medicago sativa*	L10211	Maxwell et al., 1993
MtIOMT2	*Medicago truncatula*	DQ419910	Deavours et al., 2006
MtIOMT7	*Medicago truncatula*	DQ419914	Deavours et al., 2006
GeD7OMT	*Glycyrrhiza echinata*	AB091685	Akashi et al., 2003
GeHI4'OMT	*Glycyrrhiza echinata*	AB091684	Akashi et al., 2003
LjHI4'OMT	*Lotus japonicus*	AB091686	Akashi et al., 2003

Supplemental Table S3 continued.

Name	Plant species	MaizeGDB/GenBank accession	Reference
MxpOMT1A	*Mentha x piperita*	AY337457	Willits et al., 2004
MxpOMT2	*Mentha x piperita*	AY337459	Willits et al., 2004
MxpOMT3	*Mentha x piperita*	AY337460	Willits et al., 2004
MxpOMT4	*Mentha x piperita*	AY337461	Willits et al., 2004
ObOMT1	*Ocimum basilicum*	JQ653275	Berim et al., 2012
ObOMT5	*Ocimum basilicum*	JQ653279	Berim et al., 2012
ObOMT6	*Ocimum basilicum*	JQ653280	Berim et al., 2012
Pa4'OMT	*Plagiochasma appendiculatum*	KY977687	Liu et al., 2017
CroOMT6	*Catharanthus roseus*	AY343490	Schröder et al., 2004
CroOMT2	*Catharanthus roseus*	AY127568	Cacace et al., 2003
CaFOMT1	*Chrysosplenium americanum*	U16794	Gauthier et al., 1996
CaOMT2	*Chrysosplenium americanum*	U16793	Gauthier et al., 1998
AtOMT1	*Arabidopsis thaliana*	U70424	Zhang et al., 1997
CdFOMT5	*Citrus depressa*	LC126059	Itoh et al., 2016
CreOMT2	*Citrus reticulata*	/	Liu et al., 2020
VpOMT3	*Vanilla planifolia*	DQ400400	Li et al., 2006
ShMOMT1	*Solanum habrochaites*	JF499656	Schmidt et al., 2011
ShMOMT2	*Solanum habrochaites*	JF499657	Schmidt et al., 2011
SlMOMT4	*Solanum lycopersicum*	KF740343	Kim et al., 2014

Akashi, T., Sawada, Y., Shimada, N., Sakurai, N., Aoki, T., and Ayabe, S. (2003). cDNA cloning and biochemical characterization of *S*-adenosyl-L-methionine: 2,7,4 '-trihydroxyisoflavanone 4 '-*O*-methyltransferase, a critical enzyme of the legume isoflavonoid phytoalexin pathway. Plant Cell Physiol **44,** 103-112.

Berim, A., Hyatt, D.C., and Gang, D.R. (2012). A Set of Regioselective *O*-Methyltransferases Gives Rise to the Complex Pattern of Methoxylated Flavones in Sweet Basil. Plant Physiol **160,** 1052-1069.

Cacace, S., Schroder, G., Wehinger, E., Strack, D., Schmidt, J., and Schroder, J. (2003). A flavonol *O*-methyltransferase from *Catharanthus roseus* performing two sequential methylations. Phytochemistry **62,** 127-137.

Christensen, A.B., Gregersen, P.L., Olsen, C.E., and Collinge, D.B. (1998). A flavonoid 7-*O*-methyltransferase is expressed in barley leaves in response to pathogen attack. Plant Mol Biol **36,** 219-227.

Collazo, P., Montoliu, L., Puigdomenech, P., and Rigau, J. (1992). Structure and Expression of the Lignin *O*-Methyltransferase Gene from *Zea Mays* L. Plant Mol Biol **20,** 857-867.

Deavours, B.E., Liu, C.J., Naoumkina, M.A., Tang, Y.H., Farag, M.A., Sumner, L.W., Noel, J.P., and Dixon, R.A. (2006). Functional analysis of members of the isoflavone and isoflavanone *O*-methyltransferase enzyme families from the model legume *Medicago truncatula*. Plant Mol Biol **62,** 715-733.

Gauthier, A., Gulick, P.J., and Ibrahim, R.K. (1996). CDNA cloning and characterization of a 3'/5'-*O*-methyltransferase for partially methylated flavonols from *Chrysosplenium americanum*. Plant Mol Biol **32,** 1163-1169.

Gauthier, A., Gulick, P.J., and Ibrahim, R.K. (1998). Characterization of two cDNA clones which encode *O*-methyltransferases for the methylation of both flavonoid and phenylpropanoid compounds. Arch Biochem Biophys **351,** 243-249.

He, X.Z., Reddy, J.T., and Dixon, R.A. (1998). Stress responses in alfalfa (*Medicago sativa* L). XXII. cDNA cloning and characterization of an elicitor-inducible isoflavone 7-*O*-methyltransferase. Plant Mol Biol **36,** 43-54.

Itoh, N., Iwata, C., and Toda, H. (2016). Molecular cloning and characterization of a flavonoid-*O*-methyltransferase with broad substrate specificity and regioselectivity from *Citrus depressa*. Bmc Plant Biol **16**.

Jonczyk, R., Schmidt, H., Osterrieder, A., Fiesselmann, A., Schullehner, K., Haslbeck, M., Sicker, D., Hofmann, D., Yalpani, N., Simmons, C., Frey, M., and Gierl, A. (2008). Elucidation of the final reactions of DIMBOA-glucoside biosynthesis in maize: Characterization of Bx6 and Bx7. Plant Physiol **146,** 1053-1063.

Kim, B.G., Lee, Y., Hur, H.G., Lim, Y., and Ahn, J.H. (2006). Flavonoid 3 '-*O*-methyltransferase from rice: cDNA cloning, characterization and functional expression. Phytochemistry **67,** 387-394.

Kim, J., Matsuba, Y., Ning, J., Schilmiller, A.L., Hammar, D., Jones, A.D., Pichersky, E., and Last, R.L. (2014). Analysis of natural and induced variation in tomato glandular trichome flavonoids identifies a gene not present in the reference genome. Plant Cell **26,** 3272-3285.

Kollner, T.G., Lenk, C., Zhao, N., Seidl-Adams, I., Gershenzon, J., Chen, F., and Degenhardt, J. (2010). Herbivore-Induced SABATH Methyltransferases of Maize That Methylate Anthranilic Acid Using *S*-Adenosyl-L-Methionine. Plant Physiol **153,** 1795-1807.

Li, H.M., Rotter, D., Hartman, T.G., Pak, F.E., Havkin-Frenkel, D., and Belanger, F.C. (2006). Evolution of novel *O*-methyltransferases from the *Vanilla planifolia* caffeic acid *O*-methyltransferase. Plant Mol Biol **61,** 537-552.

Li, X.Y., Chen, W.J., Zhao, Y., Xiang, Y., Jiang, H.Y., Zhu, S.W., and Cheng, B.J. (2013). Downregulation of caffeoyl-CoA *O*-methyltransferase (CCoAOMT) by RNA interference leads to reduced lignin production in maize straw. Genet Mol Biol **36,** 540-U230.

Liu, H., Xu, R.X., Gao, S., and Cheng, A.X. (2017). The Functional Characterization of a Site-Specific Apigenin 4'-*O*-methyltransferase Synthesized by the Liverwort Species *Plagiochasma appendiculatum*. Molecules **22**.

Liu, X., Wang, Y., Chen, Y., Xu, S., Gong, Q., Zhao, C., Cao, J., and Sun, C. (2020). Characterization of a Flavonoid 3'/5'/7-*O*-Methyltransferase from *Citrus reticulata* and Evaluation of the In Vitro Cytotoxicity of Its Methylated Products. Molecules **25**.

Maxwell, C.A., Harrison, M.J., and Dixon, R.A. (1993). Molecular Characterization and Expression of Alfalfa Isoliquiritigenin 2'-*O*-Methyltransferase, an Enzyme Specifically Involved in the Biosynthesis of an Inducer of *Rhizobium Meliloti* Nodulation Genes. Plant J **4,** 971-981.

Meihls, L.N., Handrick, V., Glauser, G., Barbier, H., Kaur, H., Haribal, M.M., Lipka, A.E., Gershenzon, J., Buckler, E.S., Erb, M., Kollner, T.G., and Jander, G. (2013). Natural Variation in Maize Aphid Resistance Is Associated with 2,4-Dihydroxy-7-Methoxy-1,4-Benzoxazin-3-One Glucoside Methyltransferase Activity. Plant Cell **25,** 2341-2355.

Schmidt, A., Li, C., Shi, F., Jones, A.D., and Pichersky, E. (2011). Polymethylated Myricetin in Trichomes of the Wild Tomato Species *Solanum habrochaites* and Characterization of Trichome-Specific 3 '/5 '- and 7/4 '-Myricetin *O*-Methyltransferases. Plant Physiol **155,** 1999-2009.

Schroder, G., Wehinger, E., Lukacin, R., Wellmann, F., Seefelder, W., Schwab, W., and Schroder, J. (2004). Flavonoid methylation: a novel 4 '-*O*-methyltransferase from *Catharanthus roseus*, and evidence that partially methylated flavanones are substrates of four different flavonoid dioxygenases. Phytochemistry **65,** 1085-1094.

Shimizu, T., Lin, F.Q., Hasegawa, M., Okada, K., Nojiri, H., and Yamane, H. (2012). Purification and Identification of Naringenin 7-*O*-Methyltransferase, a Key Enzyme in Biosynthesis of Flavonoid Phytoalexin Sakuranetin in Rice. J Biol Chem **287,** 19315-19325.

Wang, G.F., and Balint-Kurti, P.J. (2016). Maize Homologs of CCoAOMT and HCT, Two Key Enzymes in Lignin Biosynthesis, Form Complexes with the NLR Rp1 Protein to Modulate the Defense Response. Plant Physiol **171,** 2166-2177.

Willits, M.G., Giovanni, M., Prata, R.T.N., Kramer, C.M., De Luca, V., Steffens, J.C., and Graser, G. (2004). Bio-fermentation of modified flavonoids: an example of in vivo diversification of secondary metabolites. Phytochemistry **65,** 31-41.

Zhang, H., Wang, J., and Goodman, H.M. (1997). An Arabidopsis gene encoding a putative 14-3-3-interacting protein, caffeic acid/5-hydroxyferulic acid *O*-methyltransferase. Bba-Gene Struct Expr **1353,** 199-202.

Zhou, J.M., Fukushi, Y., Wollenweber, E., and Ibrahim, R.K. (2008). Characterization of two *O*-methyltransferase-like genes in barley and maize. Pharm Biol **46,** 26-34.

Zhou, J.M., Gold, N.D., Martin, V.J.J., Wollenweber, E., and Ibrahim, R.K. (2006). Sequential *O*-methylation of tricetin by a single gene product in wheat. Bba-Gen Subjects **1760,** 1115-1124.

Supplemental Table S4. NMR structure elucidation of 5-/7-*O*-methyl- and 5,7-*O*-dimethyl-flavonoids.

pos.	δ_H	*mult.*, J_{HH} [Hz]	δ_C
1	-	-	-
2	5.35	*dd*, 2.7/12.9	79.5
3a	2.95	*dd*, 12.9/16.3	46.1
3b	2.57	*dd*, 2.7/16.3	46.1
4	-	-	188.0
5	-	-	163.5
6	6.13	*d*, 2.0	93.8
7	-	-	165.7
8	6.05	*d*, 2.0	96.4
8a	-	-	164.4
4a	-	-	105.9
1'	-	-	131.0
2'	7.36	*d*, 8.5	128.6
3'	6.88	*d*, 8.5	115.9
4'	-	-	158.5
5'	6.88	*d*, 8.5	115.9
6'	7.36	*d*, 8.5	128.6
5-OCH_3	3.79	*s*	55.8

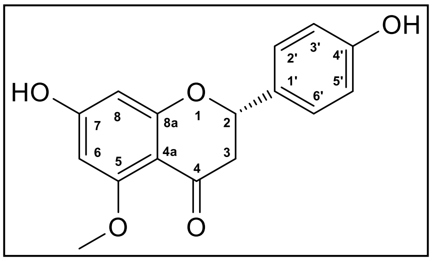

5-*O*-methyl naringenin in acetone-d_6

**pos.**	δ_H	*mult.*, J_{HH} [Hz]	δ_C
**1**	-	-	-
**2**	5.48	*dd*, 3.0/12.9	79.8
**3a**	3.21	*dd*, 12.9/17.2	43.3
**3b**	2.75	*dd*, 3.0/17.2	43.3
**4**	-	-	197.3
**5**	-	-	164.1
**6**	6.04	*d*, 2.2	94.3
**7**	-	-	168.5
**8**	6.03	*d*, 2.2	95.1
**8a**	-	-	n.d.
**4a**	-	-	103.8
**1'**	-	-	130.4
**2'**	7.39	*d*, 8.5	128.8
**3'**	6.89	*d*, 8.5	115.9
**4'**	-	-	158.5
**5'**	6.89	*d*, 8.5	115.9
**6'**	7.39	*d*, 8.5	128.8
**7-OCH_3**	3.84	*s*	56.0
**5-OH**	12.13	*s*	-
**4'-OH**	8.55	*s*	-

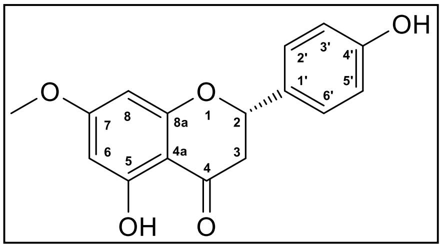

7-*O*-methyl naringenin in acetone-d_6

pos.	δ_H	*mult.*, J_{HH} [Hz]	δ_C
**1**	-	-	-
**2**	5.37	*dd*, 2.8/12.9	79.6
**3a**	3.21	*dd*, 12.9/16.3	45.8
**3b**	2.75	*dd*, 2.8/16.3	45.8
**4**	-	-	187.8
**5**	-	-	162.9
**6**	6.17	*d*, 2.3	94.1
**7**	-	-	166.2
**8**	6.14	*d*, 2.3	94.1
**8a**	-	-	n.d.
**4a**	-	-	106.2
**1'**	-	-	130.9
**2'**	7.37	*d*, 8.5	128.8
**3'**	6.88	*d*, 8.5	115.9
**4'**	-	-	158.3
**5'**	6.88	*d*, 8.5	115.9
**6'**	7.37	*d*, 8.5	128.8
**5-OCH_3**	3.81	*s*	55.8
**7-OCH_3**	3.84	*s*	55.8
**4'-OH**	8.50	*s*	-

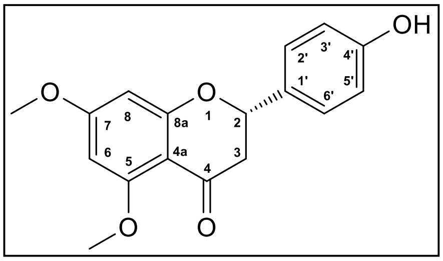

5,7-*O*-dimethyl naringenin in acetone-d_6

pos.	δ_H	*mult.*, J_{HH} [Hz]	δ_C
**1**	-	-	-
**2**	-	-	161.2
**3**	6.46	*s*	106.5
**4**	-	-	177.5
**5**	-	-	161.9
**6**	6.42	*d*, 2.2	94.1
**7**	-	-	163.5
**8**	6.58	*d*, 2.2	95.9
**8a**	-	-	160.2
**4a**	-	-	107.8
**1'**	-	-	123.0
**2'**	7.83	*d*, 8.8	128.6
**3'**	6.97	*d*, 8.8	116.6
**4'**	-	-	161.9
**5'**	6.97	*d*, 8.8	116.6
**6'**	7.83	*d*, 8.8	128.6
**5-OCH_3**	3.84	*s*	56.0

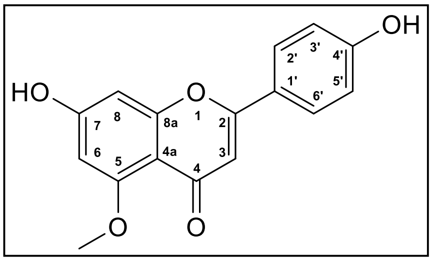

5-*O*-methyl apigenin in acetone-d_6

pos.	δ_H	*mult.*, J_{HH} [Hz]
**1**	-	-
**2**	-	-
**3**	6.67	*s*
**4**	-	-
**5**	-	-
**6**	6.32	*d*, 2.2
**7**	-	-
**8**	6.69	*d*, 2.2
**8a**	-	-
**4a**	-	-
**1'**	-	-
**2'**	7.96	*d*, 8.9
**3'**	7.03	*d*, 8.9
**4'**	-	-
**5'**	7.03	*d*, 8.9
**6'**	7.96	*d*, 8.9
**7-OCH$_3$**	3.92	*s*

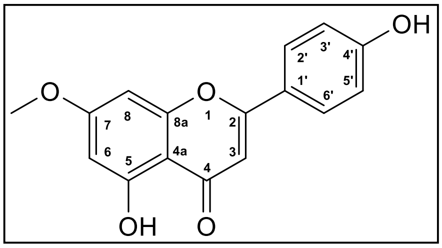

7-*O*-methyl apigenin in acetone-*d*$_6$

The identification was accomplished by comparison of the ^{1}H NMR data with that of an authentic reference standard.

pos.	δ_H	*mult.*, J_{HH} [Hz]	δ_C
**1**	-	-	-
**2**	-	-	161.2
**3**	6.45	*s*	107.3
**4**	-	-	176.5
**5**	-	-	161.7
**6**	6.47	*d*, 2.2	96.6
**7**	-	-	164.8
**8**	6.75	*d*, 2.2	93.8
**8a**	-	-	160.3
**4a**	-	-	110.0
**1'**	-	-	123.3
**2'**	7.87	*d*, 8.9	128.3
**3'**	7.00	*d*, 8.9	116.5
**4'**	-	-	161.3
**5'**	7.00	*d*, 8.9	116.5
**6'**	7.87	*d*, 8.9	128.3
**5-OCH$_3$**	3.86	*s*	56.2
**7-OCH$_3$**	3.94	*s*	55.1

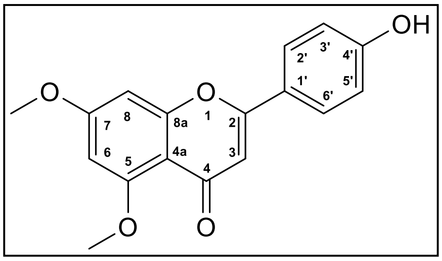

5,7-*O*-dimethyl apigenin in acetone-*d*$_6$

pos.	δ_H	***mult.*****, J$_{HH}$ [Hz]**	δ_C
**1**	-	-	-
**2**	-	-	163.6
**3**	6.56	*s*	105.5
**4**	-	-	179.6
**5**	-	-	145.6
**6**	-	-	138.3
**7**	-	-	154.1
**8**	6.84	*s*	100.2
**8a**	-	-	153.2
**4a**	-	-	111.5
**1'**	-	-	123.2
**2'**	7.82	*d*, 8.8	128.7
**3'**	6.92	*d*, 8.8	116.6
**4'**	-	-	162.1
**5'**	6.92	*d*, 8.8	116.6
**6'**	7.82	*d*, 8.8	128.7
**5-OCH$_3$**	3.85	*s*	62.6

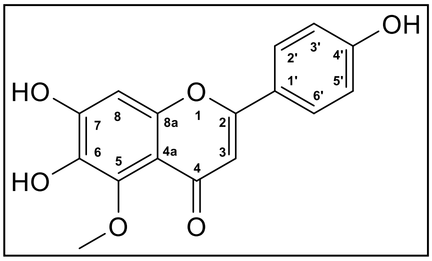

5-*O*-methyl scutellarein in methanol-d_3

pos.	δ_H	***mult.*****, J$_{HH}$ [Hz]**	δ_C
**1**	-	-	-
**2**	-	-	163.8
**3**	6.66	*s*	102.8
**4**	-	-	181.6
**5**	-	-	154.6
**6**	-	-	130.8
**7**	-	-	154.0
**8**	6.87	*s*	90.8
**8a**	-	-	150.6
**4a**	-	-	105.4
**1'**	-	-	122.7
**2'**	7.96	*d*, 8.8	128.3
**3'**	7.05	*d*, 8.8	116.2
**4'**	-	-	162.1
**5'**	7.05	*d*, 8.8	116.2
**6'**	7.96	*d*, 8.8	128.3
**7-OCH$_3$**	3.99	*s*	55.9

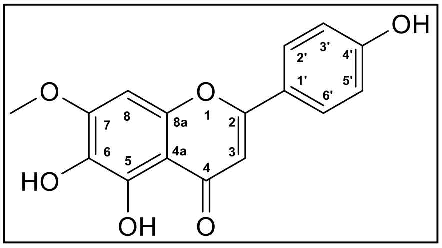

7-*O*-methyl scutellarein in acetone-d_6

pos.	δ_H	*mult.*, J_{HH} [Hz]	δ_C
**1**	-	-	-
**2**	-	-	161.2
**3**	6.50	*s*	105.9
**4**	-	-	176.1
**5**	-	-	144.5
**6**	-	-	137.5
**7**	-	-	153.0
**8**	7.09	*s*	96.6
**8a**	-	-	151.7
**4a**	-	-	112.5
**1'**	-	-	123.0
**2'**	7.90	*d*, 8.8	127.8
**3'**	7.01	*d*, 8.8	115.8
**4'**	-	-	160.0
**5'**	7.01	*d*, 8.8	115.8
**6'**	7.90	*d*, 8.8	127.8
**5-OCH$_3$**	3.87	*s*	61.3
**7-OCH$_3$**	4.00	*s*	55.9

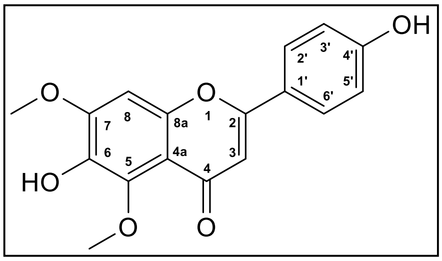

5,7-*O*-dimethyl scutellarein in acetone-d_6

pos.	δ_H	*mult.*, J_{HH} [Hz]	δ_C
**1**	-	-	-
**2**	-	-	155.6
**3**	-	-	138.3
**4**	-	-	n.d.
**5**	-	-	163.1
**6**	6.24	*d*, 2.1	98.9
**7**	-	-	165.3
**8**	6.47	*d*, 2.1	93.8
**8a**	-	-	157.3
**4a**	-	-	104.5
**1'**	-	-	121.7
**2'**	8.01	*d*, 8.9	127.8
**3'**	7.01	*d*, 8.9	115.9
**4'**	-	-	160.2
**5'**	7.01	*d*, 8.9	115.9
**6'**	8.01	*d*, 8.9	127.8
**3-OCH$_3$**	3.86	*s*	59.5

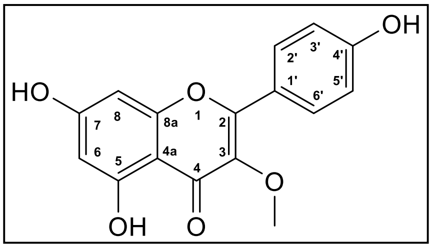

3-*O*-methyl apigenin
(isokaempferide)
in acetone-d_6

pos.	δ_H	*mult.*, J_{HH} [Hz]	δ_C
**1**	-	-	-
**2**	-	-	102.9
**3a**	2.83	*d*, 16.8	49.7
**3b**	3.20	*d*, 16.8	49.7
**4**	-	-	196.4
**5**	-	-	164.7
**6**	5.96	*d*, 2.0	96.6
**7**	-	-	167.2
**8**	5.99	*d*, 2.0	96.8
**8a**	-	-	161.8
**4a**	-	-	102.9
**1'**	-	-	134.6
**2'**	7.54	*d*, 8.7	127.7
**3'**	6.88	*d*, 8.7	115.6
**4'**	-	-	158.6
**5'**	6.88	*d*, 8.7	115.6
**6'**	7.54	*d*, 8.7	127.7
**5-OH**	12.10	*s*	-

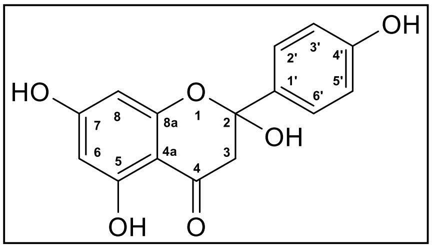

2-hydroxy naringenin in methanol-d_3

Supplemental Table S5. Product formation of maize OMTs with different substrates. Data belong to the experiment shown in Figure 3. Enzyme assays were performed with substrate (A) naringenin.

Substrate	Analyte	Enzyme	Mean area	SE	Rate of product formation (%) relative to most active enzyme
naringenin	5-*O*-Me-naringenin	EV	nd	-	-
		**FOMT2**	**45401370.0**	**1520553.3**	**100.000**
		FOMT3	4308670.7	366608.5	9.490
		FOMT4	nd	-	-
		FOMT5	nd	-	-
		BX10	35527.7	2533.6	0.078
		BX11	19946.3	751.0	0.044
		BX12	38427.3	4261.4	0.085
		BX14	nd	-	-
	sakuranetin	EV	nd	-	-
		FOMT2	nd	-	-
		FOMT3	nd	-	-
		**FOMT4**	**20238949.0**	**120674.6**	**100.000**
		FOMT5	59594.0	2828.9	0.294
		BX10	68111.7	9895.7	0.337
		BX11	33719.7	5051.9	0.167
		BX12	73842.3	3920.3	0.365
		BX14	nd	-	-
	5,7-*O*-DiMe-naringenin	EV	nd	-	-
		FOMT2	nd	-	-
		FOMT3	nd	-	-
		FOMT4	nd	-	-
		FOMT5	nd	-	-
		**FOMT2+4**	**1268022.0**	**37258.6**	**100.000**
		FOMT4+2	631538.3	8818.4	49.805
		BX10	nd	-	-
		BX11	nd	-	-
		BX12	nd	-	-
		BX14	nd	-	-

Supplemental Table S5 continued. (B) apigenin.

Substrate	Analyte	Enzyme	Mean area	SE	Rate of product formation (%) relative to most active enzyme
apigenin	5-O-Me-apigenin	EV	nd	-	-
		**FOMT2**	**33210895.0**	**1280700.4**	**100.000**
		FOMT3	1225440.0	68099.8	3.690
		FOMT4	nd	-	-
		FOMT5	nd	-	-
		BX10	95205.0	2070.0	0.287
		BX11	96935.3	5771.2	0.292
		BX12	116276.0	11595.1	0.350
		BX14	70209.3	3049.2	0.211
	genkwanin	EV	nd	-	-
		FOMT2	nd	-	-
		FOMT3	nd	-	-
		**FOMT4**	**31123877.7**	**652404.0**	**100.000**
		FOMT5	nd	-	-
		BX10	nd	-	-
		BX11	nd	-	-
		BX12	nd	-	-
		BX14	nd	-	-
	5,7-O-DiMe-apigenin	EV	nd	-	-
		FOMT2	nd	-	-
		FOMT3	15968.3	1665.7	22.983
		FOMT4	nd	-	-
		FOMT5	nd	-	-
		**FOMT2+4**	**69479.3**	**3485.8**	**100.000**
		FOMT4+2	34356.0	1741.6	49.448
		BX10	33042.3	3200.0	47.557
		BX11	11115.7	1115.4	15.999
		BX12	41505.7	762.4	59.738
		BX14	nd	-	-

Supplemental Table S5 continued. (C) scutellarein

Substrate	Analyte	Enzyme	Mean area	SE	Rate of product formation (%) relative to most active enzyme
scutellarein	5-O-Me-scutellarein	EV	nd	-	-
		**FOMT2**	**16750983.3**	**304101.2**	**100.000**
		FOMT3	108764.7	8348.8	0.649
		FOMT4	nd	-	-
		FOMT5	nd	-	-
		BX10	15653.0	2434.7	0.093
		BX11	-	-	-
		BX12	-	-	-
		BX14	nd	-	-
	7-O-Me-scutellarein	EV	nd	-	-
		FOMT2	216538.7	12953.1	0.934
		FOMT3	176534.3	13467.1	0.761
		**FOMT4**	**23189739.3**	**461690.8**	**100.000**
		FOMT5	nd	-	-
		BX10	191362.3	5027.8	0.825
		BX11	-	-	-
		BX12	-	-	-
		BX14	6156.3	1172.1	0.027
	hispidulin	EV	nd	-	-
		FOMT2	26008.3	1882.4	0.113
		FOMT3	nd	-	-
		FOMT4	nd	-	-
		**FOMT5**	**23059117.0**	**650611.1**	**100.000**
		BX10	8545.0	970.9	0.037
		BX11	-	-	-
		BX12	-	-	-
		BX14	nd	-	-

Supplemental Table S5 continued. (D) DIMBOA-Glc

Substrate	Analyte	Enzyme	Mean area	SE	Rate of product formation (%) relative to most active enzyme
DIMBOA-Glc	HDMBOA-Glc	EV	nd	-	-
		FOMT2	nd	-	-
		FOMT3	nd	-	-
		FOMT4	nd	-	-
		FOMT5	nd	-	-
		BX10	37476.3	4382.2	66.949
		BX11	24555.7	2428.6	43.867
		**BX12**	**55977.3**	**3493.1**	**100.000**
		BX14	5553.3	810.8	9.921

Supplemental Table S6. GenBank accessions and references corresponding to Figure 4B.

Name	Plant species	MaizeGDB/GenBank accession	Reference
CYP93G5 (B73)	*Zea mays*	GRMZM2G167336	Morohashi et al., 2012
CYP93G5 (W22)	*Zea mays*	Zm00004b033614	this work
CYP93G15 (W22)	*Zea mays*	Zm00004b010826	this work
CYP93G7	*Zea mays*	GRMZM2G148441	Righini et al., 2019
CYP93G10 (W22)	*Zea mays*	Zm00004b008124	this work
CYP93G3	*Sorghum bicolor*	XM_002461241	Du et al., 2010b
CYP93G1	*Oryza sativa*	AK100972	Lam et al., 2014
CYP93G2	*Oryza sativa*	AK099468	Du et al., 2010a
CYP93B16	*Glycine max*	FJ767774	Fliegmann et al., 2010
CYP93B10	*Medicago truncatula*	AC146789	Zhang et al., 2007
CYP93B11	*Medicago truncatula*	DQ354373	Zhang et al., 2007
CYP93B1	*Glycyrrhiza echinata*	AB001380	Akashi et al., 1998
CYP93B2	*Gerbera hybrida*	AF156976	Martens and Forkmann, 1999
CYP93B13	*Gentiana triflora*	AB193314	Nakatsuka et al., 2005
CYP93B6	*Perilla frutescens*	AB045592	Kitada et al., 2001
CYP93B4	*Torenia hybrida*	AB028152	Akashi et al., 1999
CYP93B3	*Antirrhinum majus*	AB028151	Akashi et al., 1999
CYP93B23	*Ocimum basilicum*	JX162213	Berim et al., 2013
CYP93B80	*Scutellaria baicalensis*	KT963453	Zhao et al., 2016
CYP93B81	*Scutellaria baicalensis*	KT963454	Zhao et al., 2016
CYP93B82	*Lonicera japonica*	KU127576	Wu et al., 2016
CYP93B83	*Lonicera japonica*	KU127578	Wu et al., 2016
CYP93B84	*Lonicera macranthoides*	KU127580	Wu et al., 2016

Akashi, T., Aoki, T., and Ayabe, S. (1998). Identification of a cytochrome P450 cDNA encoding (2*S*)-flavanone 2-hydroxylase of licorice (*Glycyrrhiza echinata* L.; Fabaceae) which represents licodione synthase and flavone synthase II. FEBS letters **431,** 287-290.

Akashi, T., Fukuchi-Mizutani, M., Aoki, T., Ueyama, Y., Yonekura-Sakakibara, K., Tanaka, Y., Kusumi, T., and Ayabe, S. (1999). Molecular cloning and biochemical characterization of a novel cytochrome P450, flavone synthase II, that catalyzes direct conversion of flavanones to flavones. Plant Cell Physiol **40,** 1182-1186.

Berim, A., and Gang, D.R. (2013). The Roles of a Flavone-6-Hydroxylase and 7-*O*-Demethylation in the Flavone Biosynthetic Network of Sweet Basil. J Biol Chem **288,** 1795-1805.

Du, Y.G., Chu, H., Chu, I.K., and Lo, C. (2010). CYP93G2 Is a Flavanone 2-Hydroxylase Required for *C*-Glycosylflavone Biosynthesis in Rice. Plant Physiol **154,** 324-333.

Du, Y.G., Chu, H., Wang, M.F., Chu, I.K., and Lo, C. (2010). Identification of flavone phytoalexins and a pathogen-inducible flavone synthase II gene (SbFNSII) in sorghum. J Exp Bot **61,** 983-994.

Fliegmann, J., Furtwangler, K., Malterer, G., Cantarello, C., Schuler, G., Ebel, J., and Mithofer, A. (2010). Flavone synthase II (CYP93B16) from soybean (*Glycine max* L.). Phytochemistry **71,** 508-514.

Kitada, C., Gong, Z.Z., Tanaka, Y., Yamazaki, M., and Saito, K. (2001). Differential expression of two cytochrome P450s involved in the biosynthesis of flavones and anthocyanins in chemo-varietal forms of *Perilla frutescens*. Plant Cell Physiol **42,** 1338-1344.

Lam, P.Y., Zhu, F.Y., Chan, W.L., Liu, H.J., and Lo, C. (2014). Cytochrome P450 93G1 Is a Flavone Synthase II That Channels Flavanones to the Biosynthesis of Tricin *O*-Linked Conjugates in Rice. Plant Physiol **165,** 1315-1327.

Martens, S., and Forkmann, G. (1999). Cloning and expression of flavone synthase II from Gerbera hybrids. Plant J **20,** 611-618.

Morohashi, K., Casas, M.I., Ferreyra, M.L.F., Mejia-Guerra, M.K., Pourcel, L., Yilmaz, A., Feller, A., Carvalho, B., Emiliani, J., Rodriguez, E., Pellegrinet, S., McMullen, M., Casati, P., and Grotewold, E. (2016). A genome-wide regulatory framework identifies maize Pericarp color1 controlled genes (vol 24, pg 2745, 2012). Plant Cell **28,** 3058-3060.

Nakatsuka, T., Nishihara, M., Mishiba, K., and Yamamura, S. (2005). Temporal expression of flavonoid biosynthesis-related genes regulates flower pigmentation in gentian plants. Plant Sci **168,** 1309-1318.

Righini, S., Rodriguez, E.J., Berosich, C., Grotewold, E., Casati, P., and Falcone Ferreyra, M.L. (2019). Apigenin produced by maize flavone synthase I and II protects plants against UV-B-induced damage. Plant Cell Environ **42,** 495-508.

Wu, J., Wang, X.C., Liu, Y., Du, H., Shu, Q.Y., Su, S., Wang, L.J., Li, S.S., and Wang, L.S. (2016). Flavone synthases from *Lonicera japonica* and *L. macranthoides* reveal differential flavone accumulation. Sci Rep-Uk **6**.

Zhang, J.A., Subramanian, S., Zhang, Y.S., and Yu, O. (2007). Flavone synthases from *Medicago truncatula* are flavanone-2-hydroxylases and are important for nodulation. Plant Physiol **144,** 741-751.

Zhao, Q., Zhang, Y., Wang, G., Hill, L., Weng, J.K., Chen, X.Y., Xue, H.W., and Martin, C. (2016). A specialized flavone biosynthetic pathway has evolved in the medicinal plant, *Scutellaria baicalensis*. Sci Adv **2**.

Supplemental Table S7: see Supplemental Tables (Excel file)

Supplemental Table S8: see Supplemental Tables (Excel file)

Supplemental Table S9. Statistical values for the analysis of the amount of non-*O*-methylated- and *O*-methylated flavonoids in different maize lines according to treatment, duration of treatment (day), and the interaction between treatment and its duration corresponding to the experiments shown in Figure 5A and Supplemental Figure S15. Values in bold indicate significant differences (P < 0.05). Depending which statistical test was used F-values or Likelihood ratios are given. F-values are given in italics.

**Maize line**	**Leaf segment**	**Flavonoid type**	**Statistical test used**	**Variance structure**	**Trans-formation**	**Factor**	**Likelihood ratio/ *F-value***	**P-value**
B75	upper	non-*O*-methylated	ANOVA		none	treatment	*1.119*	0.303
						day	*0.889*	0.357
						treatment:day	*1.036*	0.321
		O-methylated	gls*	day	log	treatment	0.183	0.669
						day	5.537	**0.019**
						treatment:day	6.380	**0.012**
B75	middle	non-*O*-methylated	ANOVA		log	treatment	*213.755*	**< 0.001**
						day	*9.782*	**0.005**
						treatment:day	*0.273*	0.607
		O-methylated	ANOVA		log	treatment	*299.102*	**< 0.001**
						day	*103.501*	**< 0.001**
						treatment:day	*0.013*	0.911
B75	lower	non-*O*-methylated	gls*	day	log	treatment	2.581	0.108
						day	5.014	**0.025**
						treatment:day	0.066	0.797
		O-methylated	gls*	day-treatment	log	treatment	0.234	0.629
						day	12.835	**< 0.001**
						treatment:day	13.147	**< 0.001**
Hybrid ‚Sweet Nugget‘	upper	non-*O*-methylated	ANOVA		log	treatment	*18.120*	**< 0.001**
						day	*0.261*	0.613
						treatment:day	*0.635*	0.432
		O-methylated	ANOVA		log	treatment	*8.361*	**0.007**
						day	*0.461*	0.502
						treatment:day	*0.604*	0.443
Hybrid ‚Sweet Nugget‘	middle	non-*O*-methylated	gls*	day-treatment	none	treatment	14.052	**< 0.001**
						day	4.855	**0.028**
						treatment:day	15.459	**< 0.001**
		O-methylated	gls*	day-treatment	log	treatment	56.102	**< 0.001**
						day	0.011	0.916
						treatment:day	9.091	**0.003**
Hybrid ‚Sweet Nugget‘	lower	non-*O*-methylated	gls*	treatment	log	treatment	6.836	**0.009**
						day	0.310	0.578
						treatment:day	1.277	0.259
		O-methylated	gls*	day-treatment	log	treatment	10.955	**< 0.001**
						day	3.805	0.051
						treatment:day	6.398	**0.011**
W22	middle	non-*O*-methylated	ANOVA		log	treatment:	*309.744*	**< 0.001**
						day	*118.761*	**< 0.001**
						treatment:day	*4.206*	**0.050**
		O-methylated	ANOVA		log	treatment	*251.700*	**< 0.001**
						day	*231.600*	**< 0.001**
						treatment:day	*2.237*	0.146
B73	middle	non-*O*-methylated	ANOVA		log	treatment	*299.930*	**< 0.001**
						day	*24.126*	**< 0.001**
						treatment:day	*8.544*	**0.007**
		O-methylated	gls*	day-treatment	log	treatment	50.882	**< 0.001**
						day	12.482	**< 0.001**
						treatment:day	3.204	0.074

*gls, generalized least squares; The varIdent error structure for each day-treatment combination (day-treatment), or just for each treatment (treatment) was used.

Supplemental Table S10: see Supplemental Tables (Excel file)

Supplemental Table S11: see Supplemental Tables (Excel file)

Supplemental Table S12. MS settings used for the analysis on the timsTOF mass spectrometer.

**ESI ion source settings**				
Ionization mode:	positive	positive	negative	negative
Capillary voltage:	4500 V	4500 V	3500 V	3500 V
Drying gas (nitrogen):	8 L/min, 280°C	8 L/min, 280°C	8 L/min, 280°C	10 L/min, 230°C
Nebulizer gas (nitrogen):	2.8 bar	2.8 bar	2.8 bar	1.8 bar
**Aquisition parameters**				
Scan mode:	full	auto MS/MS	full	auto MS/MS
Collison energy:	-	alternating 20 eV/50 eV	-	alternating 20 eV/50 eV

Supplemental Table S13. MS settings used for the analysis on the QTRAP 6500+.

**Turbospray ESI ion source settings**			
Analytes	**flavonoids**	**flavonoid glycosides**	**benzoxazinoids**
Ionization mode:	positive	negative	negative
Ion spray voltage:	+5500 V	-4500 V	-4500 V
Turbo gas temperature:	650°C	650°C	650°C
Curtain gas:	40 psi	40 psi	40 psi
Collision gas:	medium level	medium level	medium level
Nebulizer gas:	60 psi	60 psi	70 psi
Heating gas:	60 psi	60 psi	70 psi

Supplemental Table S14. Mass analyzer settings used for the analysis of flavonoids and additional phenylpropanoids on the QTRAP 6500+. Retention time (RT) was used to distinguish between compounds with the same MRM transition. Compounds marked with (*) were verified by an authentic standard. Abbreviations: Me, methyl; DP, declustering potential; EP, entrance potential; CE, collision energy; CXP, collision cell exit potential; V, volts.

**Flavonoid / phenylpropanoid**	**RT (min)**	**MRM transition *m/z***	**DP (V)**	**EP (V)**	**CE (V)**	**CXP (V)**
Caffeic acid*	3.01	181 $[M+H]^+$ → 163	6	5	13	24
O-Me-Caffeic acid-1	-	195 $[M+H]^+$ → 180	50	5	10	6
O-Me-Caffeic acid-2	-	195 $[M+H]^+$ → 162	50	5	20	6
Resveratrol*	5.45	229 $[M+H]^+$ → 107	51	5	27	14
O-Me-Resveratrol	7.23	243 $[M+H]^+$ → 107	50	5	30	6
Chrysin*	8.36	255 $[M+H]^+$ → 153	20	5	51	12
5-*O*-Me-Chrysin	6.40	269 $[M+H]^+$ → 254	50	5	35	6
Apigenin*	6.63	271 $[M+H]^+$ → 153	50	3.5	41	4
Genistein*	6.66	271 $[M+H]^+$ → 153	50	3.5	41	4
Naringenin chalcone*	6.51	273 $[M+H]^+$ → 153	46	3	29	4
Naringenin*	6.64	273 $[M+H]^+$ → 153	41	4.5	31	4
5-*O*-Me-Apigenin*	5.04	285 $[M+H]^+$ → 270	50	6	31	8
7-*O*-Me-Apigenin (Genkwanin)*	8.53	285 $[M+H]^+$ → 242	50	6	43	4
5-*O*-Me-Genistein	5.22	285 $[M+H]^+$ → 270	50	6	31	8
4'-*O*-Me-Apigenin (Acacetin)*	8.44	285 $[M+H]^+$ → 242	50	7	43	4
5-*O*-Me-Naringenin*	5.04	287 $[M+H]^+$ → 167	36	5	31	6
7-*O*-Me-Naringenin (Sakuranetin)*	8.43	287 $[M+H]^+$ → 167	36	5	31	6
Scutellarein*	5.32	287 $[M+H]^+$ → 123	50	3.5	47	4
Luteolin*	5.89	287 $[M+H]^+$ → 153	50	3	43	4
Kaempferol*	6.80	287 $[M+H]^+$ → 153	50	2	39	4
Dihydrokaempferol*	5.12	289 $[M+H]^+$ → 243	50	4	15	6
2-Hydroxynaringenin*	5.53	289 $[M+H]^+$ → 121	21	5	21	14
Eriodictyol*	5.83	289 $[M+H]^+$ → 153	46	7	33	4
5,7-*O*-DiMe-Apigenin*	6.38	299 $[M+H]^+$ → 284	50	4	37	6
5-*O*-Me-Luteolin	4.53	301 $[M+H]^+$ → 286	50	3	35	6
7-*O*-Me-Luteolin	7.57	301 $[M+H]^+$ → 286	50	3	35	6
5-*O*-Me-Scutellarein*	4.75	301 $[M+H]^+$ → 286	50	3	33	6
7-*O*-Me-Scutellarein*	6.01	301 $[M+H]^+$ → 286	50	3	33	6
6-*O*-Me-Scutellarein (Hispidulin)*	6.67	301 $[M+H]^+$ → 286	21	5	33	28
5-*O*-Me-Kaempferol	5.54	301 $[M+H]^+$ → 286	50	2	35	6
3-*O*-Me-Kaempferol (Isokaempferide)*	7.03	301 $[M+H]^+$ → 286	50	4	27	8
7-*O*-Me-Kaempferol	8.66	301 $[M+H]^+$ → 286	50	2	35	6
5,7-*O*-DiMe-Naringenin*	6.60	301 $[M+H]^+$ → 181	46	11	31	6
5-*O*-Me-Eriodictyol	4.42	303 $[M+H]^+$ → 124	50	7	55	4
7-*O*-Me-Eriodictyol	7.50	303 $[M+H]^+$ → 124	50	7	55	4
5-*O*-Me-Dihydrokaempferol	4.44	303 $[M+H]^+$ → 107	50	4	55	2
7-*O*-Me-Dihydrokaempferol	6.99	303 $[M+H]^+$ → 107	50	4	55	2
Quercetin*	5.96	303 $[M+H]^+$ → 153	50	7	45	4
O-Me-2-hydroxynaringenin (peak 1)	6.44	303 $[M+H]^+$ → 167	21	5	21	20
7-*O*-Me-2-hydroxynaringenin	7.07	303 $[M+H]^+$ → 167	21	5	21	20
O-Me-2-hydroxynaringenin (peak 2)	8.32	303 $[M+H]^+$ → 167	21	5	21	20
Dihydroquercetin (Taxifolin)*	4.41	305 $[M+H]^+$ → 153	50	7.5	25	6
2-Hydroxyeriodictyol	4.88	305 $[M+H]^+$ → 137	21	5	21	14
5,7-*O*-DiMe-Luteolin	5.15	315 $[M+H]^+$ → 300	50	3	35	6
O-DiMe-Luteolin (*O*-Methyl group likely on A + B ring)	8.70	315 $[M+H]^+$ → 300	50	3	35	6
5-*O*-Me-Isokaempferide	5.25	315 $[M+H]^+$ → 254	11	5	39	30
7-*O*-Me-Isokaempferide	8.96	315 $[M+H]^+$ → 254	11	5	39	30
5,7-*O*-DiMe-Scutellarein*	5.34	315 $[M+H]^+$ → 254	50	3	41	8
5,6-*O*-DiMe-Scutellarein	5.50	315 $[M+H]^+$ → 282	11	5	33	34
6,7-*O*-DiMe-Scutellarein (Cirsimaritin)*	7.68	315 $[M+H]^+$ → 282	11	5	33	34
5-*O*-Me-Quercetin	4.85	317 $[M+H]^+$ → 302	50	7	35	6
7-*O*-Me-Quercetin	7.63	317 $[M+H]^+$ → 302	50	7	35	6
O-DiMe-2-Hydroxynaringenin (Xilonenin; peak 1)*	5.51	317 $[M+H]^+$ → 181	1	5	21	20
O-DiMe-2-Hydroxynaringenin (Xilonenin; peak 2)*	6.81	317 $[M+H]^+$ → 181	1	5	21	20
5-*O*-Me-Dihydroquercetin	3.81	319 $[M+H]^+$ → 123	50	7.5	55	6
7-*O*-Me-Dihydroquercetin	6.18	319 $[M+H]^+$ → 123	50	7.5	55	6
Myricetin*	5.07	319 $[M+H]^+$ → 153	50	7	40	4
5-*O*-Me-Myricetin	4.12	333 $[M+H]^+$ → 318	50	7	35	6
7-*O*-Me-Myricetin	6.57	333 $[M+H]^+$ → 318	50	7	35	6

Supplemental Table S15. Mass analyzer settings used for the analysis of flavonoid glycosides on the QTRAP 6500+. Compounds marked with (*) were verified by an authentic standard. Abbreviations: Me, methyl; DP, declustering potential; EP, entrance potential; CE, collision energy; CXP, collision cell exit potential; V, volts.

**flavonoid glycoside**	**MRM transition *m/z***	**DP (V)**	**EP (V)**	**CE (V)**	**CXP (V)**
Kaempferol-7-*O*-beta-D-glucopyranoside*	447 $[M-H]^-$ → 285	-50	-11	-38	-8
O-Me-Kaempferol-7-*O*-beta-D-glucopyranoside-1	461 $[M-H]^-$ → 299	-50	-6	-40	-4
O-Me-Kaempferol-7-O-beta-D-glucopyranoside-2	461 $[M-H]^-$ → 284	-50	-6	-50	-4
Kaempferol-3-*O*-beta-rutinoside*	593 $[M-H]^-$ → 285	-50	-6	-46	-8
O-Me-Kaempferol-3-*O*-beta-rutinoside-1	607 $[M-H]^-$ → 299	-50	-6	-40	-4
O-Me-Kaempferol-3-*O*-beta-rutinoside-2	607 $[M-H]^-$ → 284	-50	-6	-50	-4

Supplemental Table S16. Mass analyzer settings used for the analysis of BXs on the QTRAP 6500+. Compounds marked with (*) were verified by an authentic standard. Abbreviations: DP, declustering potential; EP, entrance potential; CE, collision energy; CXP, collision cell exit potential; V, volts.

**benzoxazinoid**	**MRM transition** ***m/z***	**DP (V)**	**EP (V)**	**CE (V)**	**CXP (V)**
MBOA	164 $[M-H]^-$ → 149	-40	-4	-20	-2
HBOA-Glc	326 $[M-H]^-$ → 164	-40	-4	-20	-5
DIBOA-Glc	342 $[M-H]^-$ → 134	-40	-4	-24	-4
HMBOA-Glc	356 $[M-H]^-$ → 194	-40	-4	-15	-4
DIMBOA-Glc*	372 $[M-H]^-$ → 210	-40	-4	-15	-4
HM_2BOA-Glc	386 $[M-H]^-$ → 224	-40	-4	-15	-4
HDMBOA-Glc*	432 $[M+FA-H]^-$ → 356	-40	-4	-20	-3
DIM_2BOA-Glc	402 $[M-H]^-$ → 194	-40	-4	-18	-5
HDM_2BOA-Glc	462 $[M+FA-H]^-$ → 194	-40	-4	-25	-3

Supplemental Table S17. Authentic standards used for identification and quantification. Abbreviations: Me, methyl.

**Compound**	**Supplier**	**self purified**
**Phenylpropanoids**		
Caffeic acid	Merck; S23175, > 98 % (HPLC)	-
Resveratrol	Sigma, R-5010, approx. 99 %	-
**Flavonoids**		
Chrysin	Sigma, 95082, analyt. Standard	-
Apigenin	Sigma, 42251, analyt. standard	-
Genistein	Carl Roth, 0716.2, >= 98 %, für Biochemie	-
Naringenin	Sigma, W530098, 98 %	-
Naringeninchalcone	PhytoLab, 83877, phyproof	-
Scutellarein	Sigma, S0327, >= 98 % (HPLC)	-
Luteolin	Enzo Life Sciences, ALX-385-007	-
Kaempferol	EMD Chemicals San Diego, 420345	-
Dihydrokaempferol	Sigma, 91216, >= 95 % (HPLC)	-
2-Hydroxynaringenin		✓
Eriodictyol	Sigma, 94258, >= 95 % (HPLC)	-
Quercetin hydrate	Acros organics, 95 %	-
Dihydroquercetin (Taxifolin) hydrate	Sigma, T4512, >= 90 % (HPLC)	-
Myricetin	Carl Roth, 6461.1, >= 95 %	-
**Flavonoid glycosides**		
Kaempferol-7-O-ß-D-glucopyranoside	Sigma, 18854, >= 90 % (HPLC)	-
Kaempferol-3-O-ß-rutinoside	Sigma, 90242, >= 98 % (HPLC)	-
***O*-methylflavonoids**		
5-*O*-Me-Apigenin	-	✓
7-*O*-Me-Apigenin (Genkwanin)	TRC, G360000	✓
4'-*O*-Me-Apigenin (Acacetin)	Carl Roth, 5010.1	-
5-*O*-Me-Naringenin	Sigma, SMB00201, >= 95 %	✓
7-*O*-Me-Naringenin (Sakuranetin)	Sigma, 73422, analyt. Standard	✓
5,7-*O*-DiMe-Apigenin	-	✓
5-*O*-Me-Scutellarein	-	✓
7-*O*-Me-Scutellarein	-	✓
6-*O*-Me-Scutellarein (Hispidulin)	Sigma, SML0582, >= 98 % (HPLC)	-
3-*O*-Me-Kaempferol (Isokaempferide)	-	✓
5,7-*O*-DiMe-Naringenin	-	✓
5,7-*O*-DiMe-Scutellarein	-	✓
6,7-*O*-DiMe-Scutellarein (Cirsimaritin)	Sigma, SMB00174, ≥90% (LC/MS-ELSD)	-
O-DiMe-2-Hydroxynaringenin (Xilonenin)	-	✓

Supplemental Table S18: see Supplemental Tables (Excel file)

Supplemental Table S19. RT-qPCR primers.

Primer name	Target gene	Primer Sequence (5' → 3')
ZmFOMT2_qPCR_Fwd<br>ZmFOMT2_qPCR_Rev	*FOMT2*	GTCCCTGTGCTACGCCAAAT<br>GTCGACGGTCGAATCATCAC
ZmFOMT3_qPCR_Fwd_neu1<br>ZmFOMT3_qPCR_Rev_neu1	*FOMT3*	TGATGAGTCAAATACAAAACGTC<br>GGGAGCCAACAACGGGTAGA
GRMZM2G423331_Fwd3<br>GRMZM2G423331_Rev3	*FOMT4*	CCCAGTACAAGCACCTGAGAG<br>ACCACCTACATGTTCCACACC
GRMZM2G422750-Fwd2<br>GRMZM2G422750-Rev2	*CHS2* (UTR)	CGTCCGCAAATAATGTGCTCTC<br>TAGCTCTACCCTGGTCTTGC
GRMZM2G175076-Fwd1<br>GRMZM2G175076-Rev1	*CHIL*(UTR)	TTCGTGAATGTCCGTCCTGT<br>CCACAACGACAATCTGCACAA
GRMZM2G062396-Fwd1<br>GRMZM2G062396-Rev1	*F3H* (UTR)	TATAGCTACGTGCGACCGTG<br>CTGGAACCGCACGTTGAAAA
ZmFNSI_Fwd<br>ZmFNSI_Rev	*FNSI1*	AGGAGAAGGCCAAGCTCTACT<br>CCCATGGTCTCCTTGAAATC
FNSI2_qPCR_Fwd2<br>FNSI2_qPCR_Rev2	*FNSI2*	CCGGACAACCCACCATCCTT<br>CCCAGCGCCTCCTTGATGTA
ZmFLS1/2_qPCR_Fwd2<br>ZmFLS1/2_qPCR_Rev2	*FLS1/FLS2*	TACGAGGCCAAGTACGTGCC<br>TCCACGAACATCGGCCATGA
ZmF3-H_qPCR_Fwd1<br>ZmF3-H_qPCR_Rev1	*F3,H*	ATCCGGACGTGCTCAGGAAG<br>TCTCCTTGATCACCGCCGTG
Zm33614_qPCR_Fwd1<br>Zm33614_qPCR_Rev1	*F2H1*	CAATCCACTGCGCCGCCCT<br>TACGTAGCCGCTCTTGCCG
Zm10826_qPCR_Fwd3<br>Zm10826_qPCR_Fwd3	*F2H2*	GGCAGCAGGGACAGGGAG<br>GGCAGCAGGGACAGGGAG
FNSII_qPCR_Fwd1<br>FNSII_qPCR_Rev1	*FNSII1*	GCGTACAAGGAGACGTTGCG<br>CGATGGCCCACACGTTGATG
Zm08124_qPCR_Fwd3<br>Zm08124_qPCR_Rev3	*FNSII2*	AAGACGATGGACAAGGAATAAG<br>CCGCCGCCTGGTCCTCC
Zm39147_qPCR_Fwd2<br>Zm39147_qPCR_Rev2	*ZmCYP93G6*	AGTCGACGGTGCTTATCCAC<br>ATGTATTGGAAGTGCTTCCCG
Zm33036_qPCR_Fwd3<br>Zm33036_qPCR_Rev3	*ZmCYP93F6*	ACCTGCAGGACTACATCGGC<br>GCCGTCAGTATCCGCTCCAT
ZmBx10/11_UTR_qFwd2<br>ZmBx10_UTR_qRev2	*BX10* (UTR)	GAAGGTGTTGATAGTATATTATG<br>ACTGGTACGCTTGTAACTTGA
ZmBx11_UTR_qFwd3<br>ZmBx11_UTR_qRev3	*BX11* (UTR)	TGTGTGTTGGTCAAGTAGTCGA<br>TCCTTGTTGCCATGACACAAC
BxD-2.1<br>BxD-2.2	*BX14*	GAAAGCCGCTTCTTGATGCC<br>GGAACATATTGCCCGCAACG
ZmUBCP_qPCR_Fwd1<br>ZmUBCP_qPCR_Rev1	*UBCP* (GRMZM2G102471)	TTGTCCCTGAGATTGCTCACA<br>CACCAGTTTGGCCAGCTTTTA
ZmMEP_qPCR_Fwd<br>ZmMEP_qPCR_Rev	*MEP* (GRMZM2G018103)	TGTACTCGGCAATGCTCTTG<br>TTTGATGCTCCAGGCTTACC

Supplemental Table S20. PCR primers for the amplification of full-length open reading frames of investigated FOMTs and CYP93Gs.

Primer name	Target gene	Primer Sequence (5' → 3')	Restriction recognition site	Application
FOMT2-W22-fwd	*FOMT2*	CACCATGGCACTCAGCACTCAGGA		TOPO cloning
FOMT2-W22-rev		TCATGGATAGACCTCGATGAC		
FOMT4-W22-IBA-F	*FOMT4*	ATGGTACGTCTCAGCGCATGGCCTGCACGACGGCAGC	BsmBI	pASK-IBA37plus cloning
FOMT4-W22-IBA-R		ATGGTACGTCTCATATCACTTGGTGAACTCGAGCGCCCA		
Bx10g-synt_pASK-37.fwd	*FOMT3*	ATGGTAGGTCTCAGCGCATGGCGTTTACGGAAGAGAGTTC	BsaI	
Bx10g-synt_pASK-37.rev		ATGGTAGGTCTCATATCAGGGATAGACCTCAATCACAGACA		
Bx10e-synt_pASK-37.fwd	*FOMT5*	ATGGTAGGTCTCAGCGCATGGCCTTACTCGGGGAATACTC	BsaI	
Bx10e-synt_pASK-37.rev		ATGGTAGGTCTCATATCAATTCGGGTACAGTTCGATGATAGA		
SpeI-ZmG167336_Fwd	*F2H1* (B73)	TCGTACTAGTATGGAAGCTGATGCTGCT	SpeI	subcloning into pESC-Leu2d
ZmG167336-PacI_Rev		ACACTTAATTAACTACGTAGCCGCTCTTGC	PacI	
Zm33614_NotI_Fwd	*F2H1* (W22)	CGTGCGGCCGCAATGGAAGCTGATGCTGC	NotI	
Zm33614_SacI_Rev		AGGGAGCTCTTAAGTAGCAGCTCTAGCTG	SacI	
Zm10826_NotI_Fwd	*F2H2*	CGTGCGGCCGCAATGGAAGCTGCTGCTG	NotI	
Zm10826_SacI_Rev		AGGGAGCTCTCAAGTAGCTGTAGCTTGC	SacI	
Zm08124_NotI_Fwd	*FNSII2*	CGTGCGGCCGCAATGAAGGAACAACAACCTAGA	NotI	
Zm08124_SacI_Rev		AGGGAGCTCTTAAACAACTGCTGGAAATGGA	SacI	
Zm39147_NotI_Fwd	*ZmCYP93G6*	CGTGCGGCCGCAATGGAAGAACAACAATTGAGAG	NotI	
Zm39147_SacI_Rev		AGGGAGCTCTCAAACAACAGGTGGAAATG	SacI	
Zm33036_NotI_Fwd	*ZmCYP93F6*	CGTGCGGCCGCAATGGAAGTTGTTACCGCTA	NotI	
Zm33036_SacI_Rev		AGGGAGCTCTCAAACACCAGCGGTTT	SacI	
