# Supplementary material for: Biosynthesis and antifungal activity of fungus-induced O-methylated flavonoids in maize
Source: Plant Physiol. 2021 Oct 27;188(1):167–90. doi: 10.1093/plphys/kiab496 (PMC8774720; doi:10.1093/plphys/kiab496)

**Supplemental Data Set S2:** NMR spectra

# 5-*O*-methyl naringenin

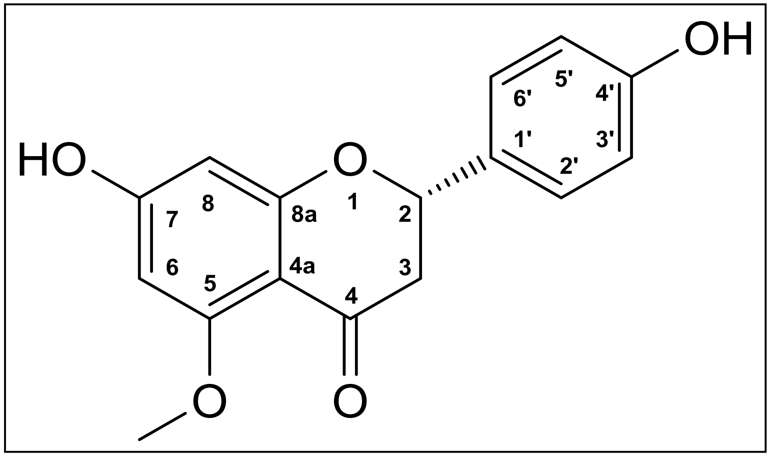


*Molecules* **2004**, *9*(7), 602-608; https://doi.org/10.3390/90700602
*J. Nat. Prod.* **1998**, *61*(1), 142-144; https://doi.org/10.1021/np970293i

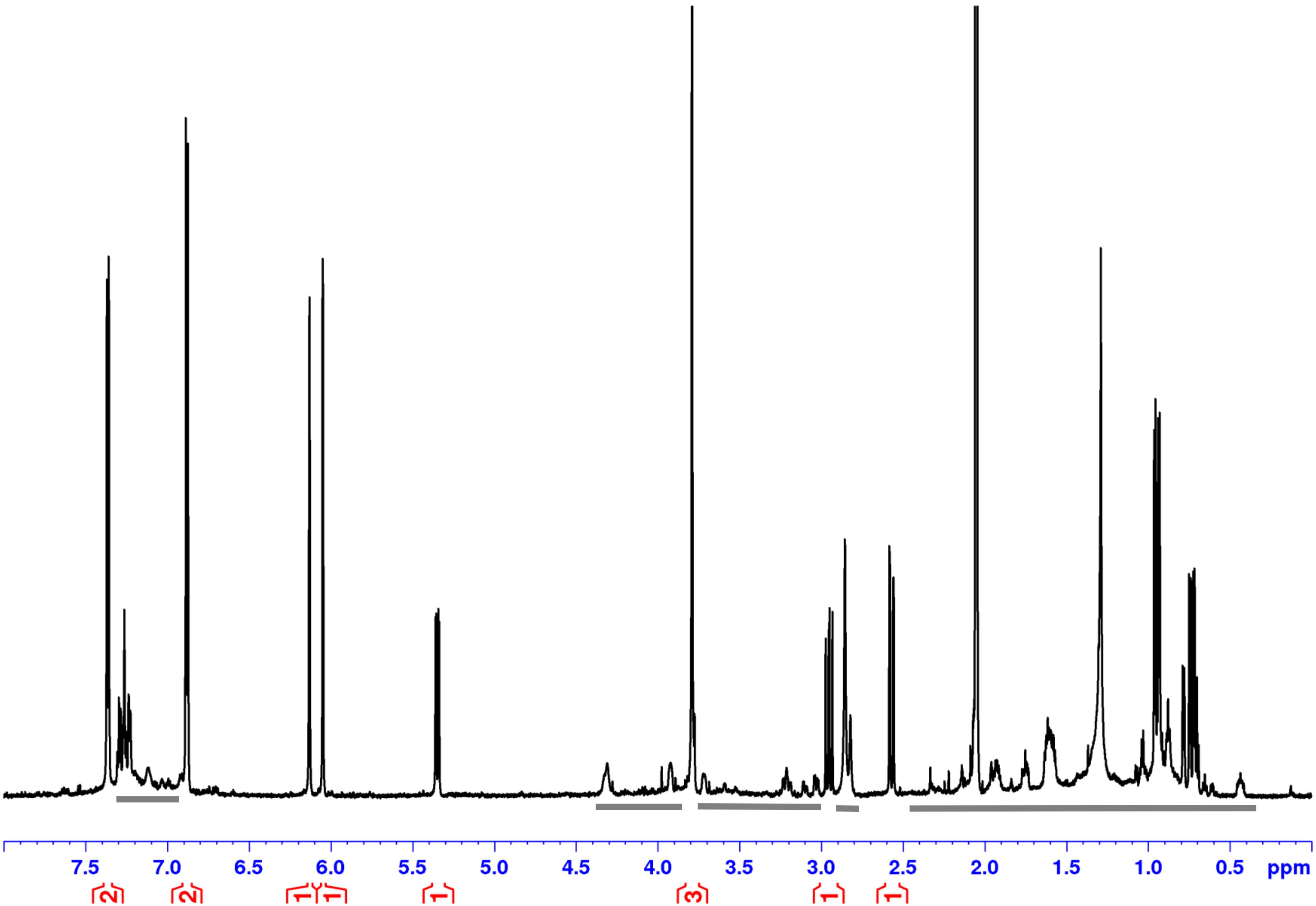


$^{1}H$ NMR spectrum of 5-*O*-methyl naringenin in acetone-$d_6$ (grey bars indicate impurities)

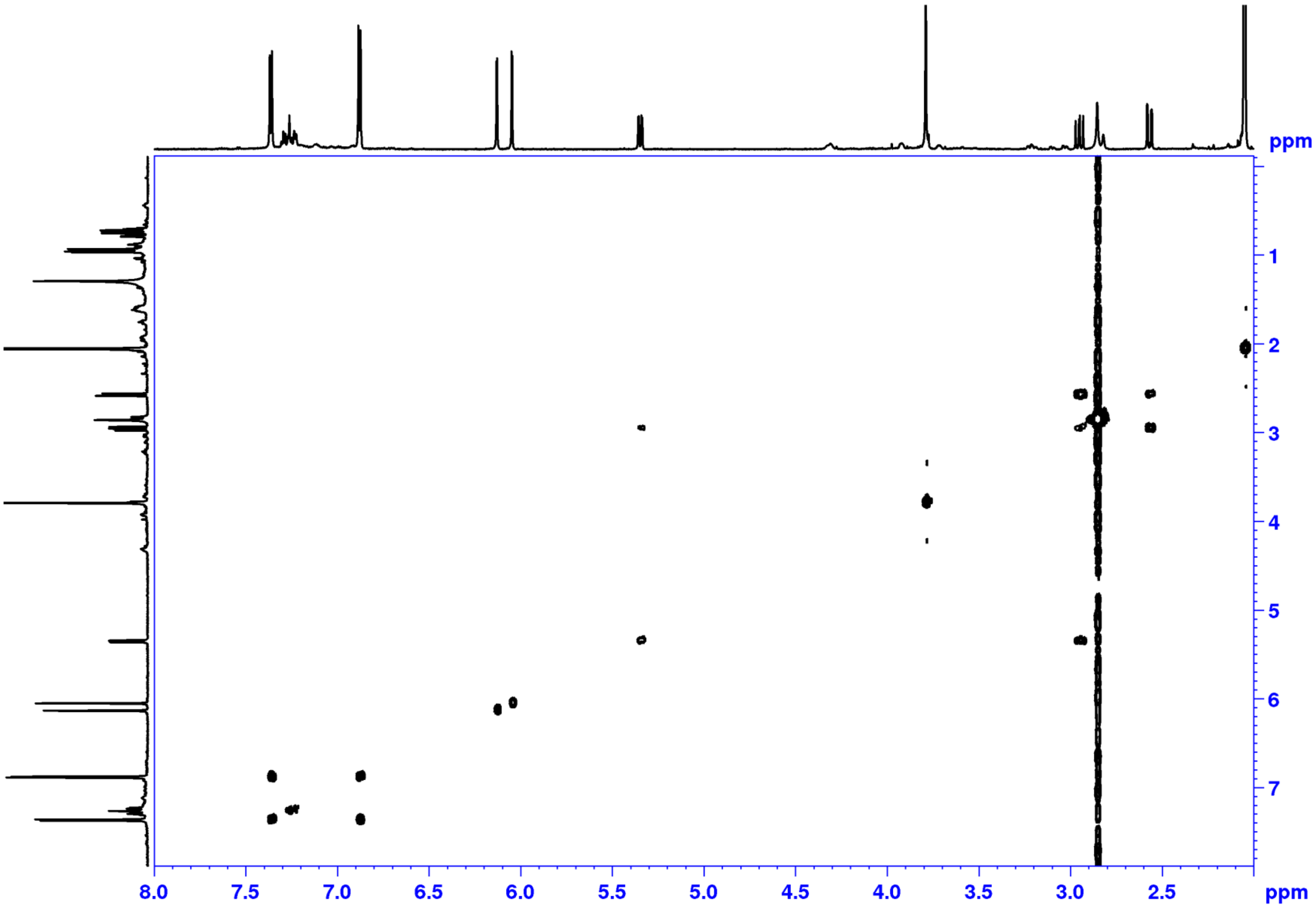


$^1$H-$^1$H COSY spectrum of 5-*O*-methyl naringenin in acetone-$d_6$

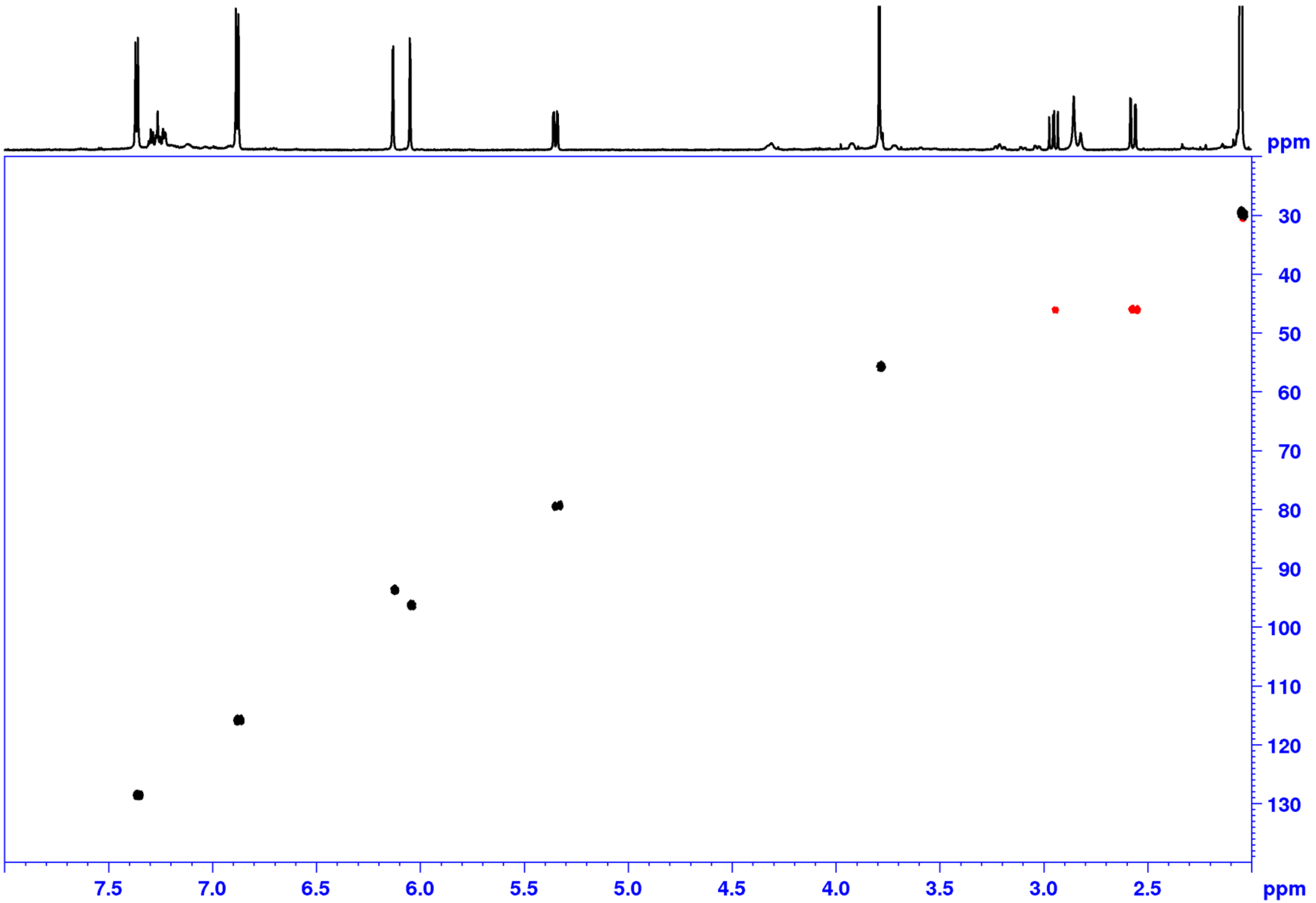


$^{1}$H-$^{13}$C HSQC spectrum of 5-*O*-methyl naringenin in acetone-$d_6$

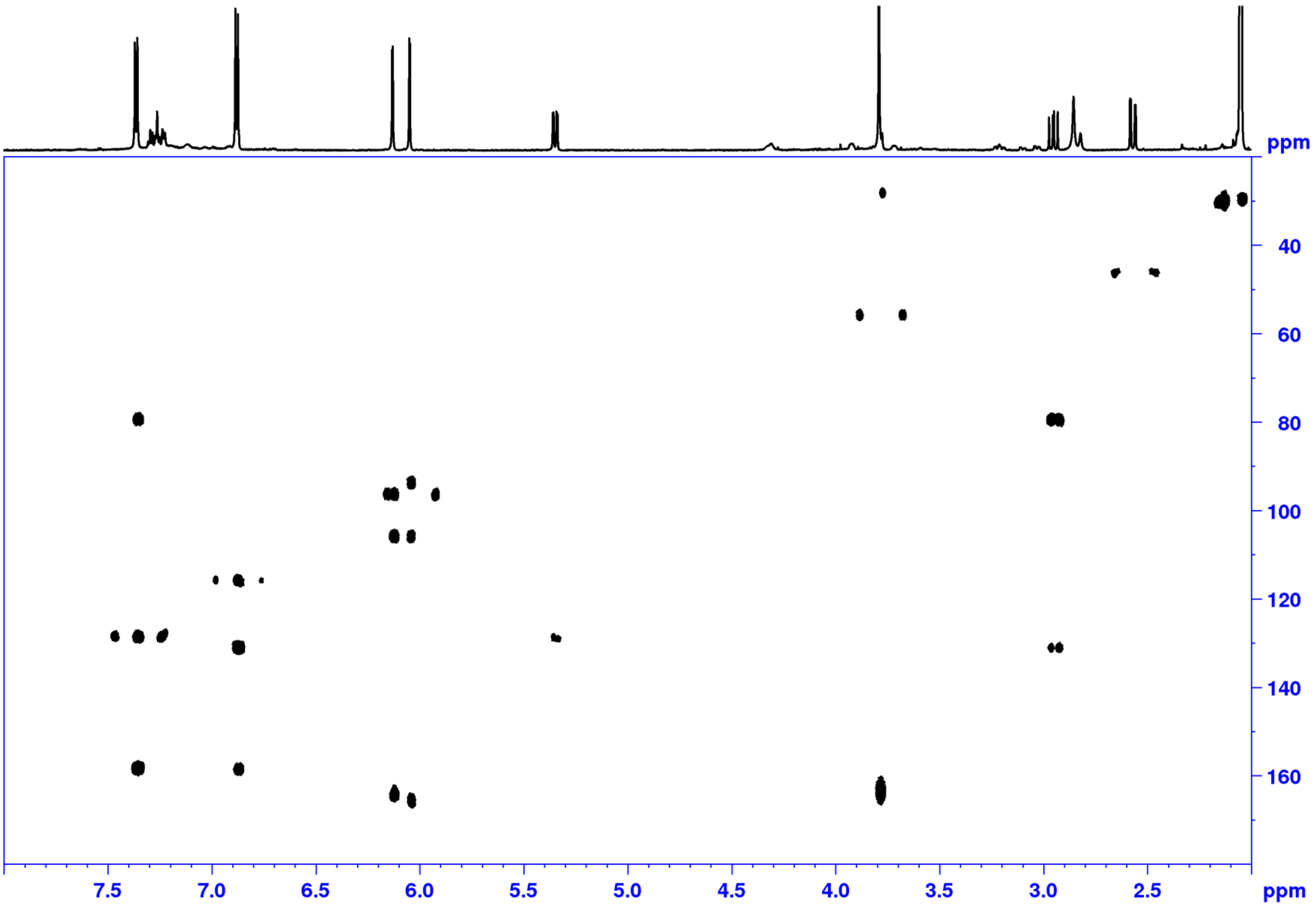


$^{1}H$-$^{13}C$ HMBC spectrum of 5-*O*-methyl naringenin in acetone-$d_6$

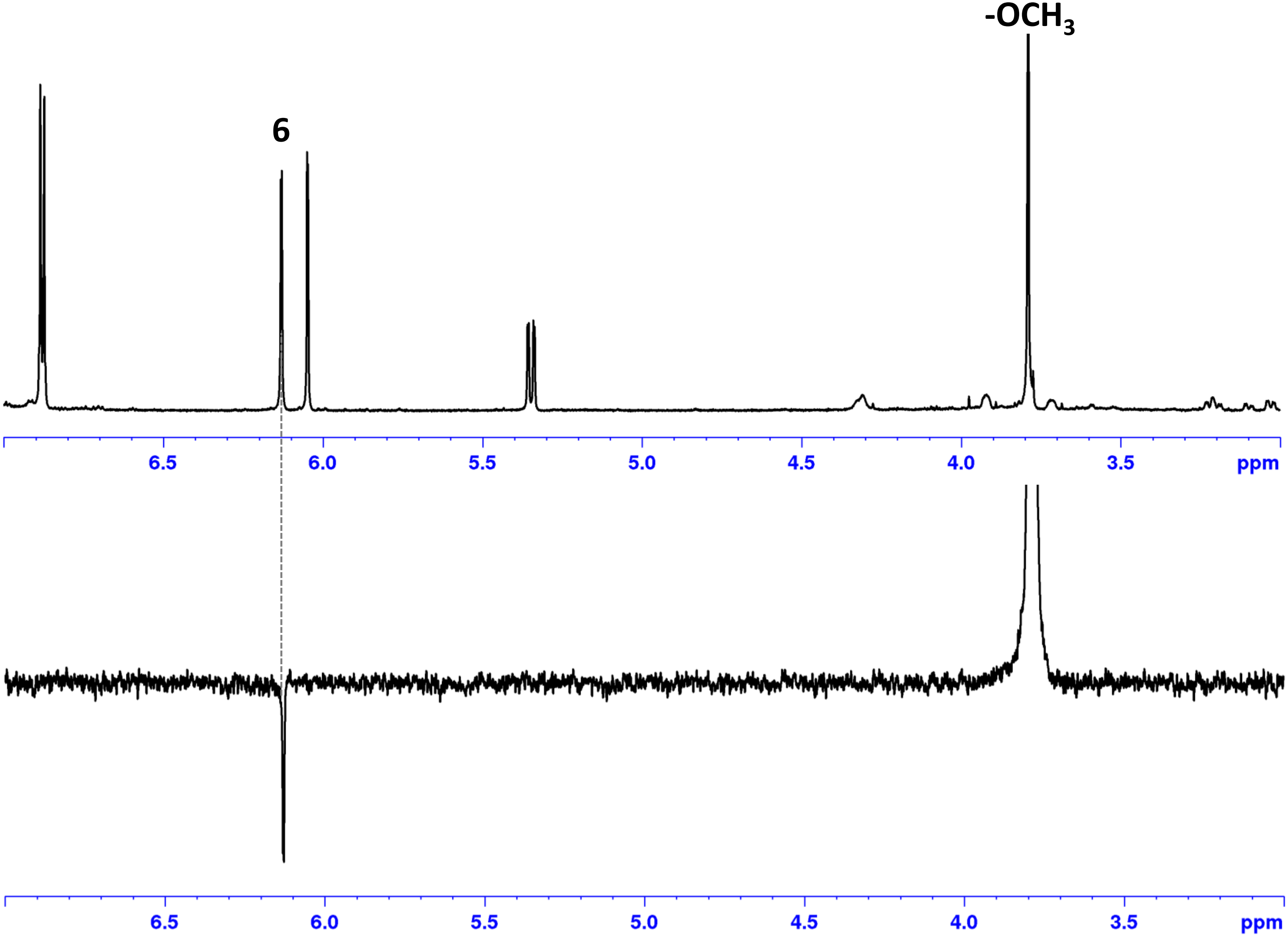


Detail of the $^{1}H$ NMR spectrum of 5-*O*-methyl naringenin (above) and a selective NOESY spectrum (below) with the transmitter set on the -OMe frequency (both in acetone-$d_6$)

# 7-*O*-methyl naringenin (sakuranetin)

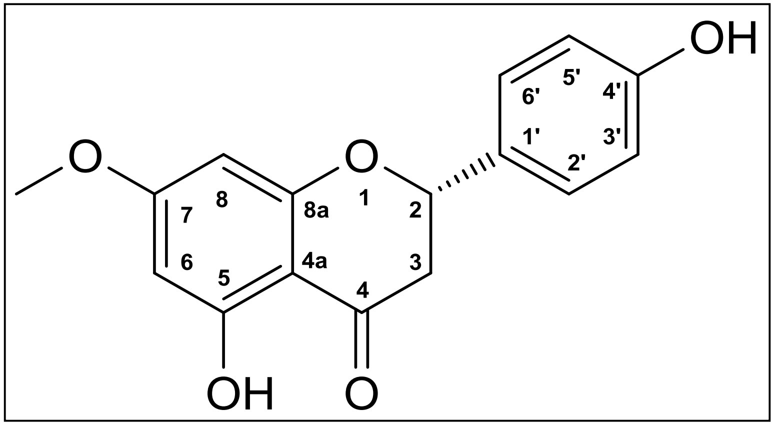


*Phytochemistry* **2005,** *66*(14), 1698-1706;
https://doi.org/10.1016/j.phytochem.2005.04.031

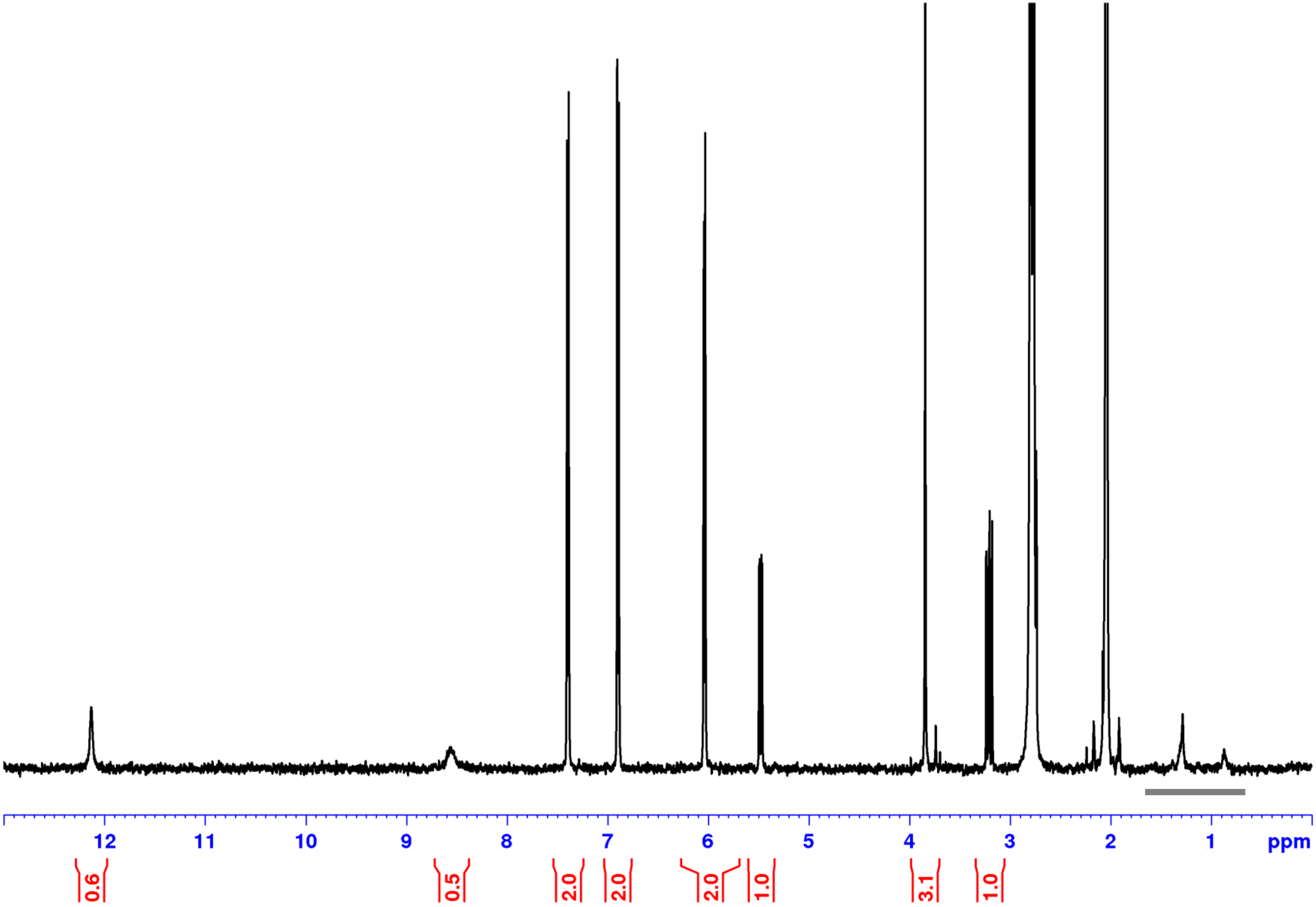

$^{1}H$ NMR spectrum of 7-*O*-methyl naringenin in acetone-$d_6$ (grey bar indicates impurities)

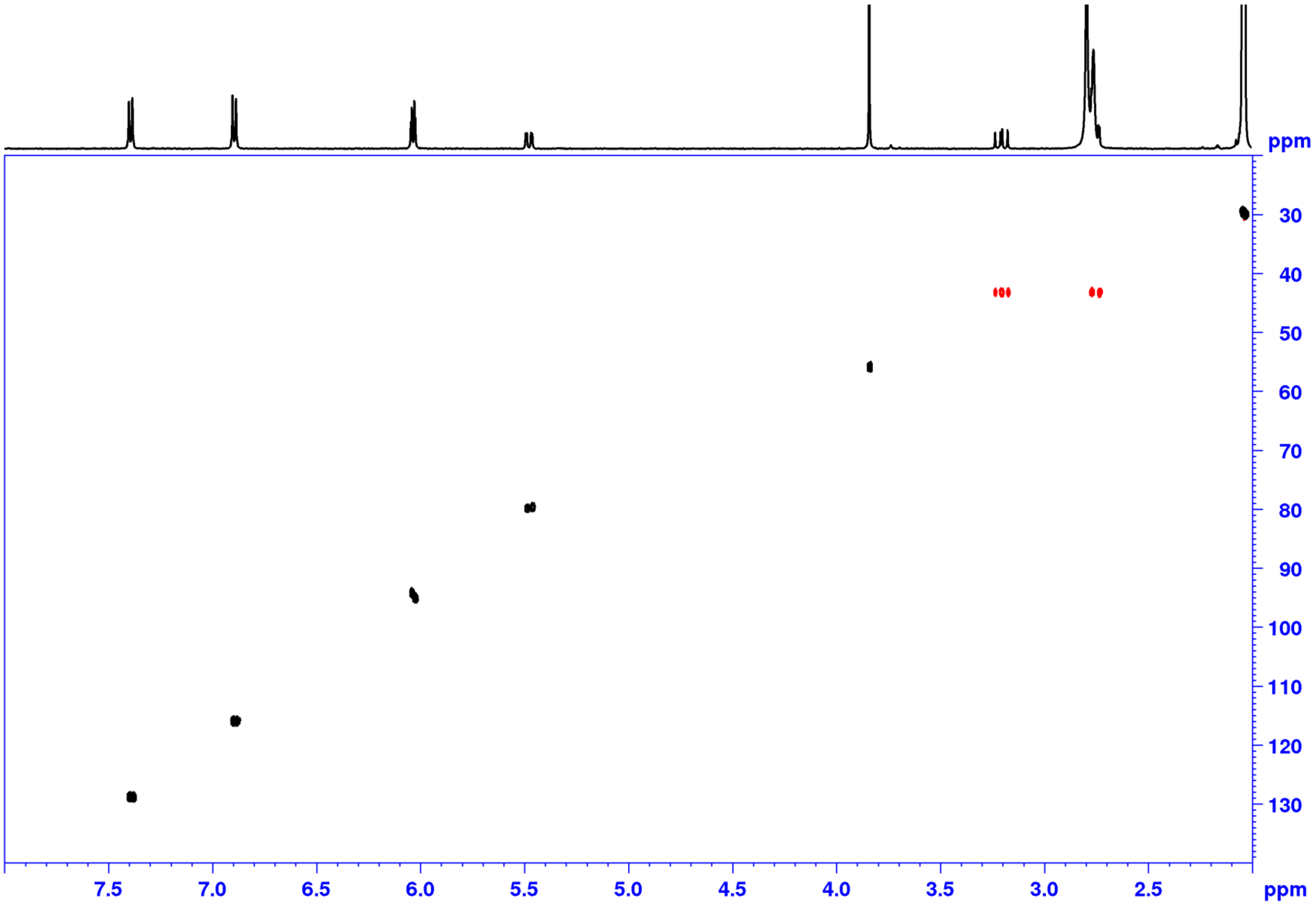


$^{1}H$-$^{13}C$ HSQC spectrum of 7-*O*-methyl naringenin in acetone-$d_6$

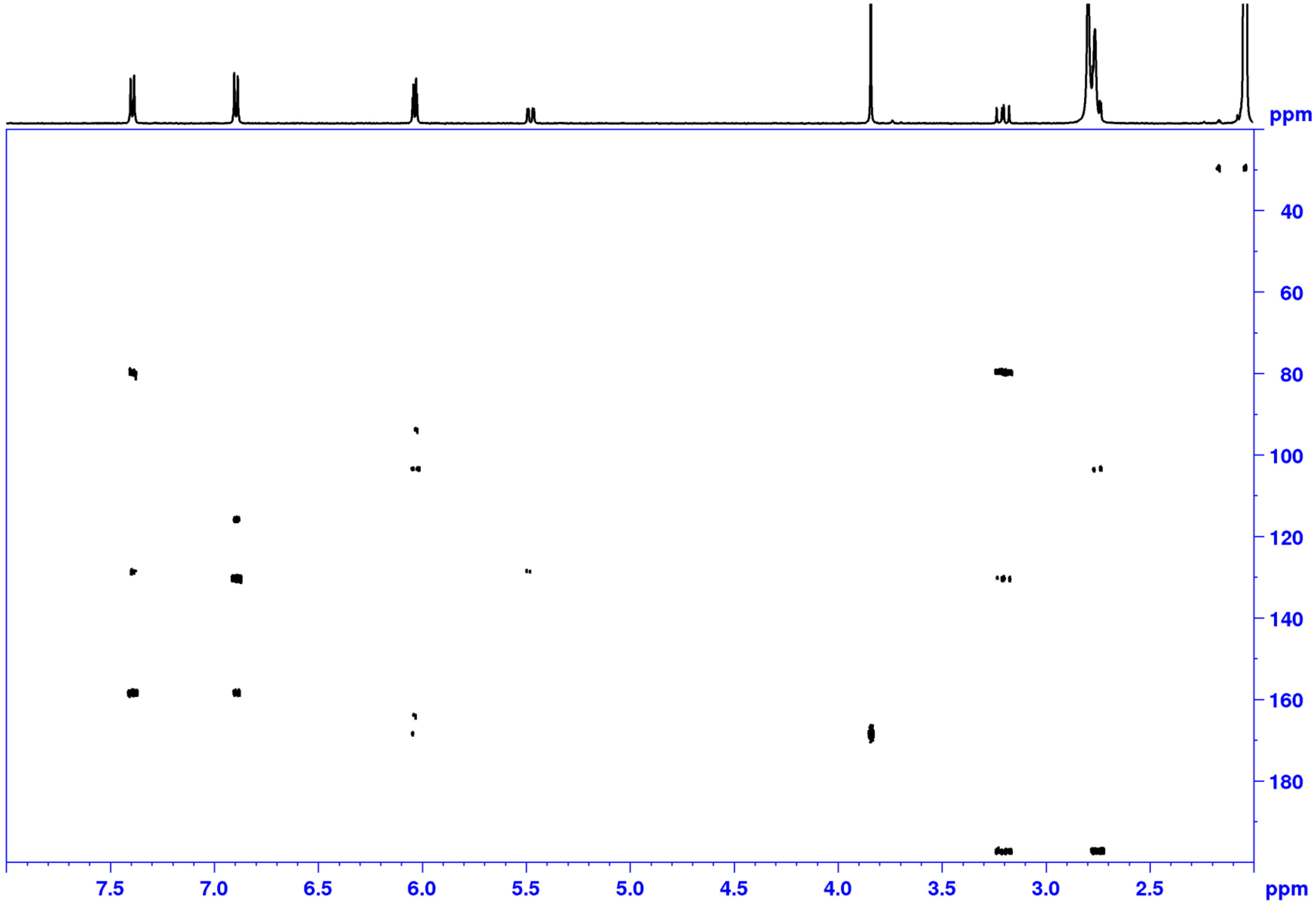


$^{1}H$-$^{13}C$ HMBC spectrum of 7-*O*-methyl naringenin in acetone-$d_6$

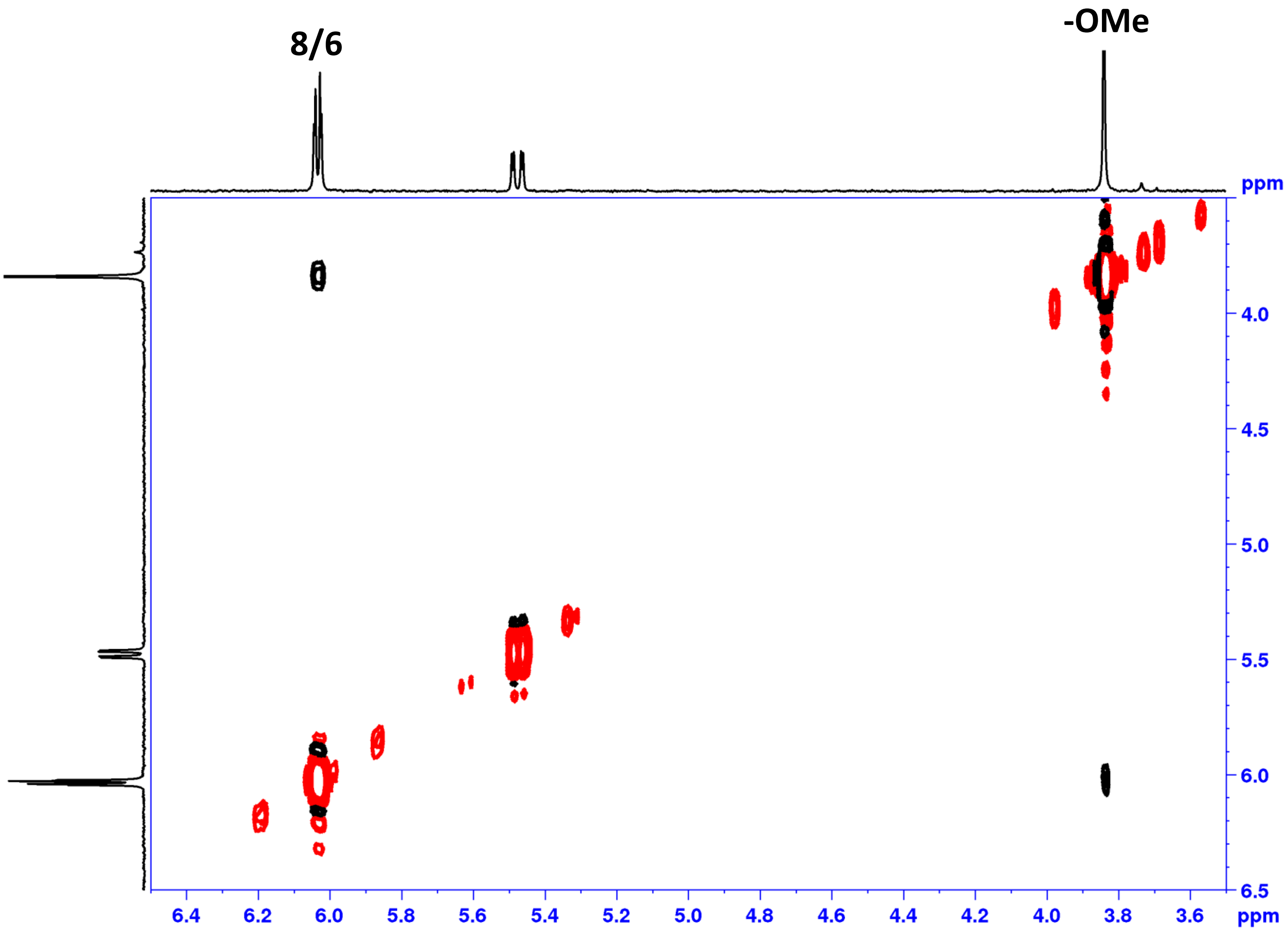


Detail of the $^{1}H$-$^{1}H$ ROESY spectrum of 7-*O*-methyl naringenin in acetone-$d_6$

# 5,7-*O*-dimethyl naringenin

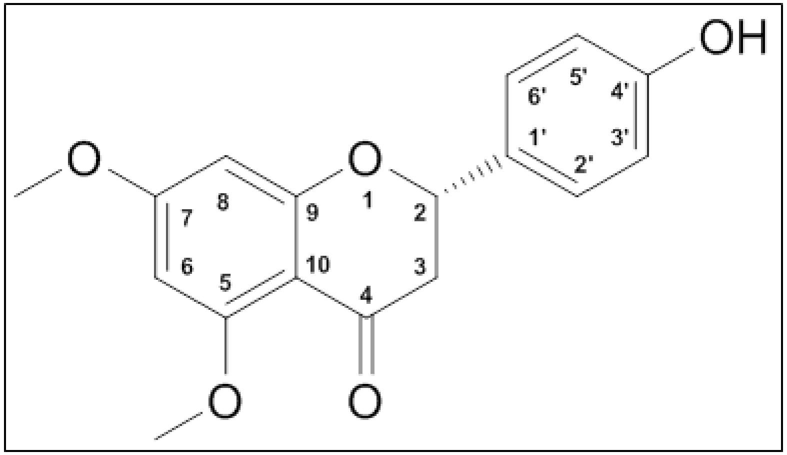


*Bulletin of the Korean Chemical Society* **2007**, *28*(12), 2527-2530; http://dx.doi.org/10.5012/bkcs.2007.28.12.2527

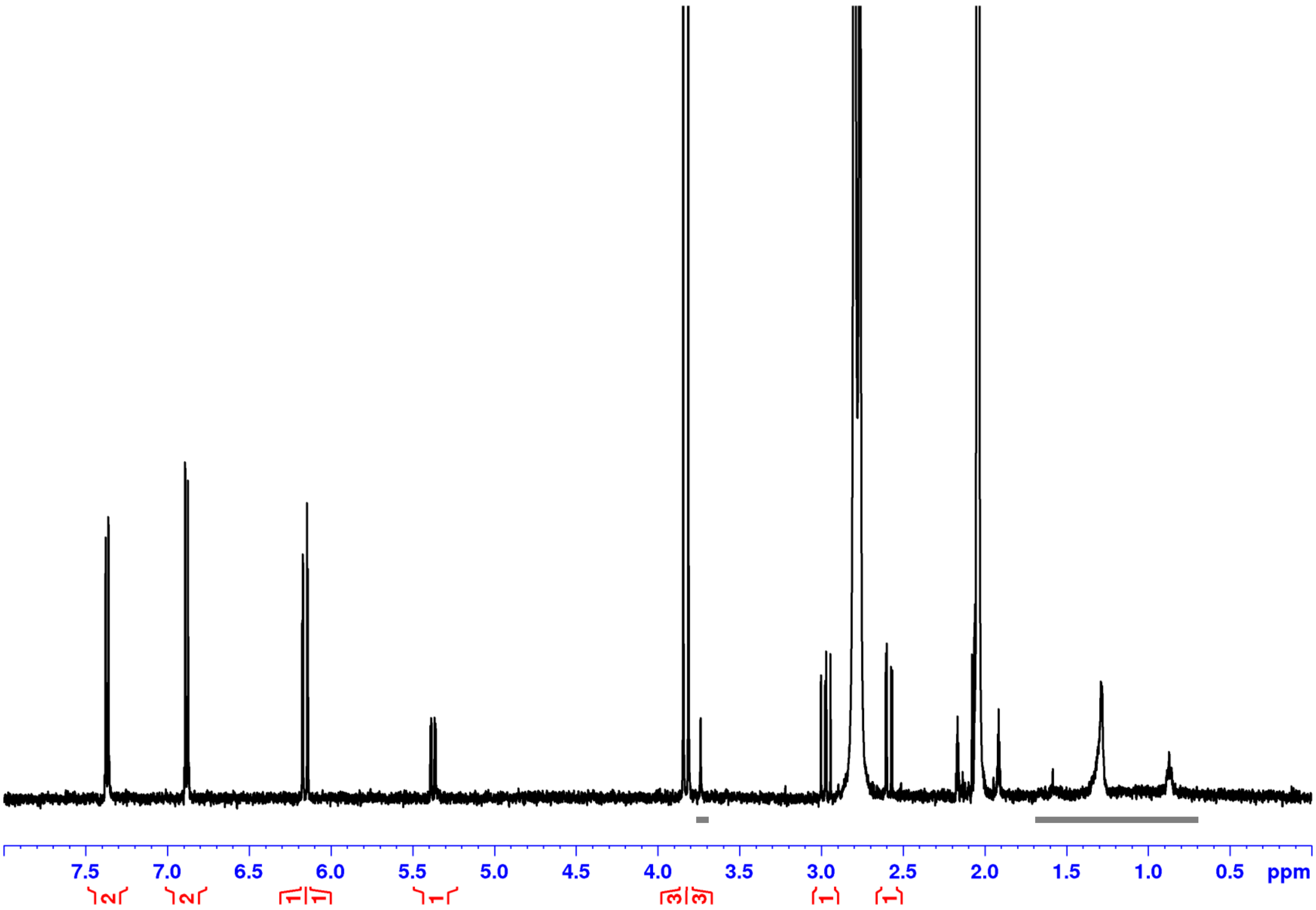


$^1H$ NMR spectrum of 5,7-*O*-dimethyl naringenin in acetone-$d_6$ (grey bar indicates impurities)

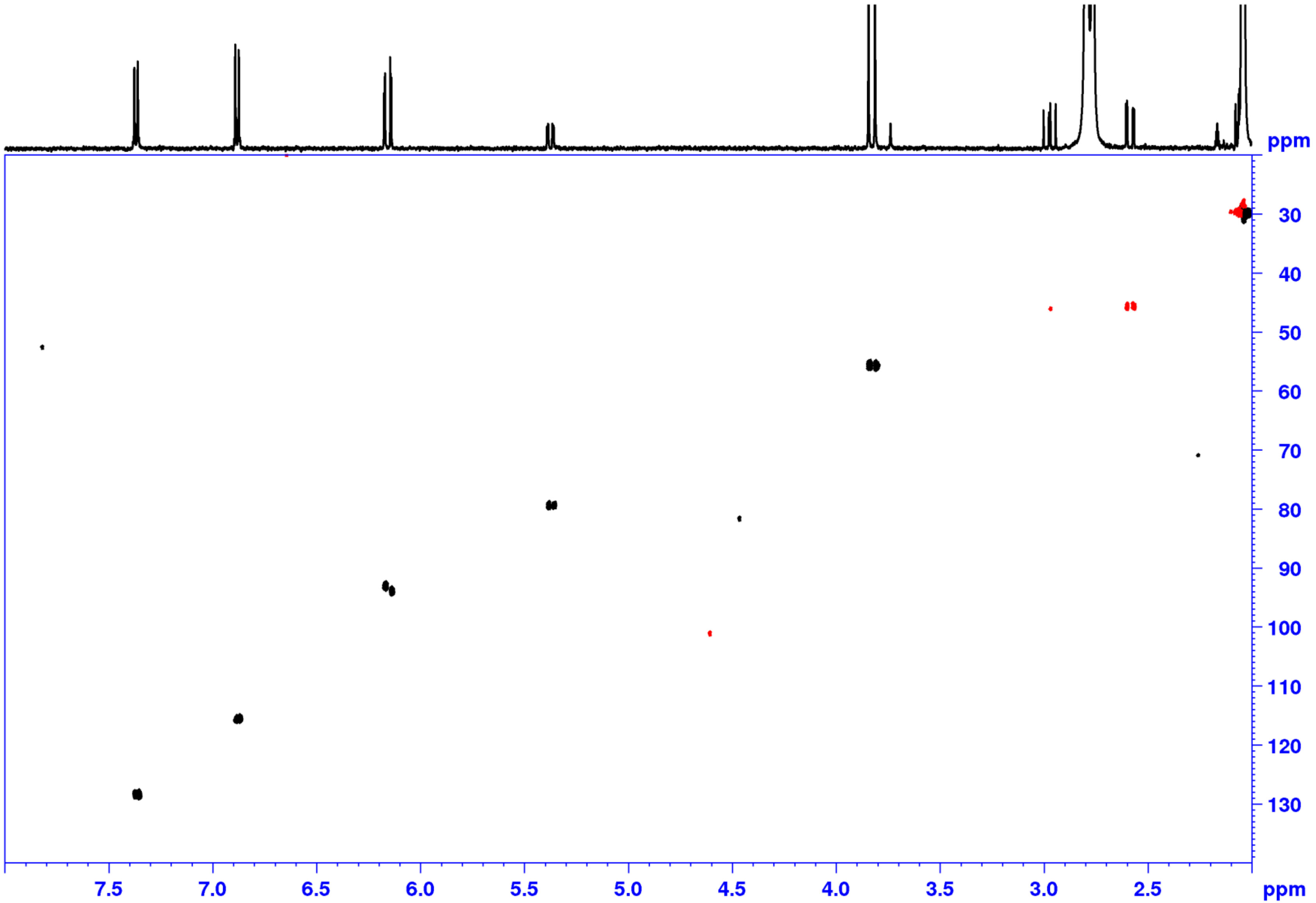


$^{1}H$-$^{13}C$ HSQC spectrum of 5,7-*O*-dimethyl naringenin in acetone-$d_6$

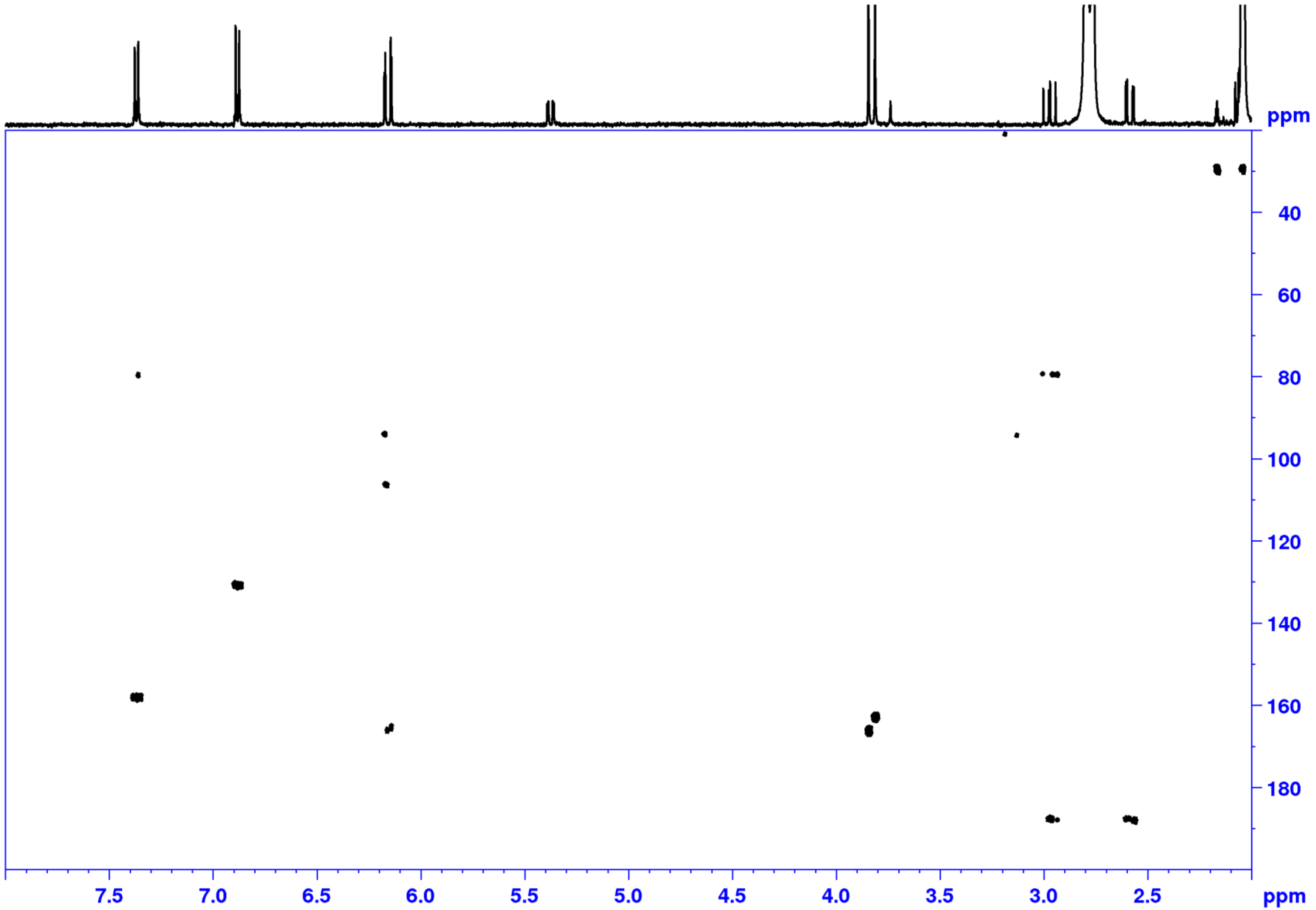


$^{1}H$-$^{13}C$ HMBC spectrum of 5,7-*O*-dimethyl naringenin in acetone-$d_6$

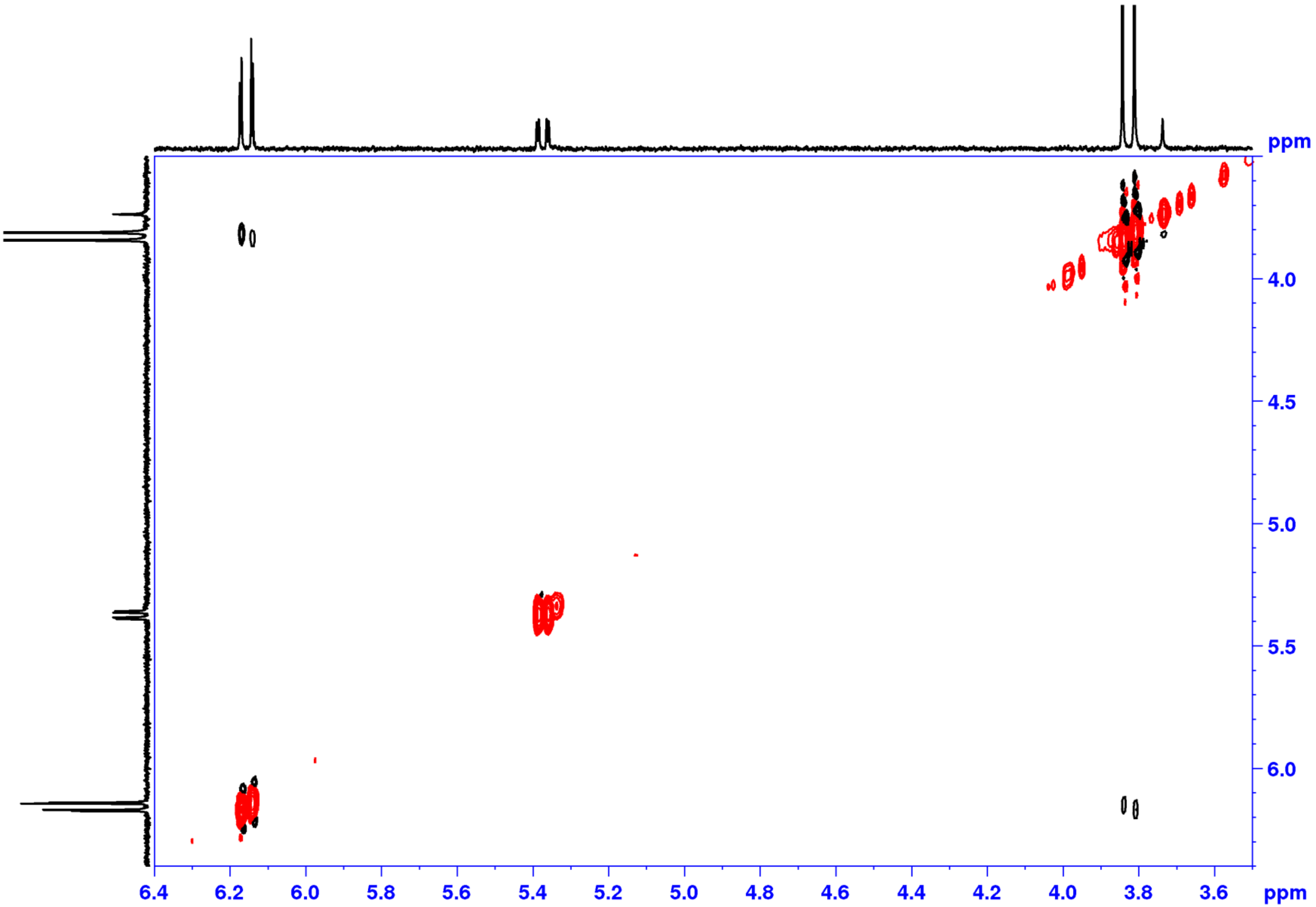


Detail of the $^{1}H$-$^{1}H$ ROESY spectrum of 5,7-*O*-dimethyl naringenin in acetone-$d_6$

# 5-*O*-methyl apigenin

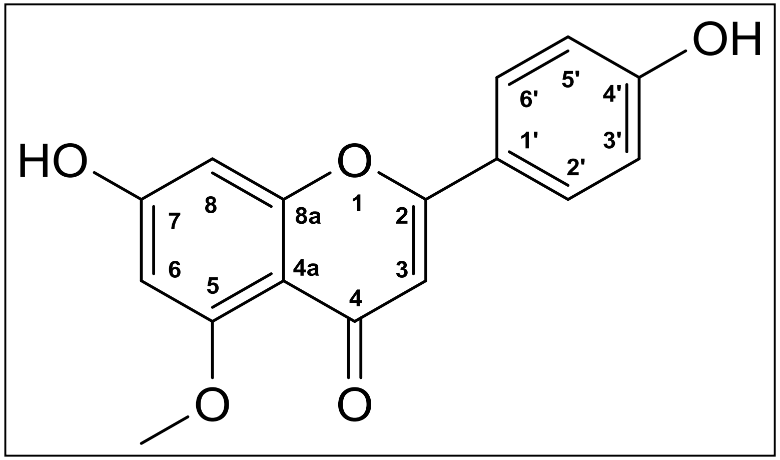


*Bulletin of the Korean Chemical Society* **2007**, *28*(12), 2527-2530; http://dx.doi.org/10.5012/bkcs.2007.28.12.2527

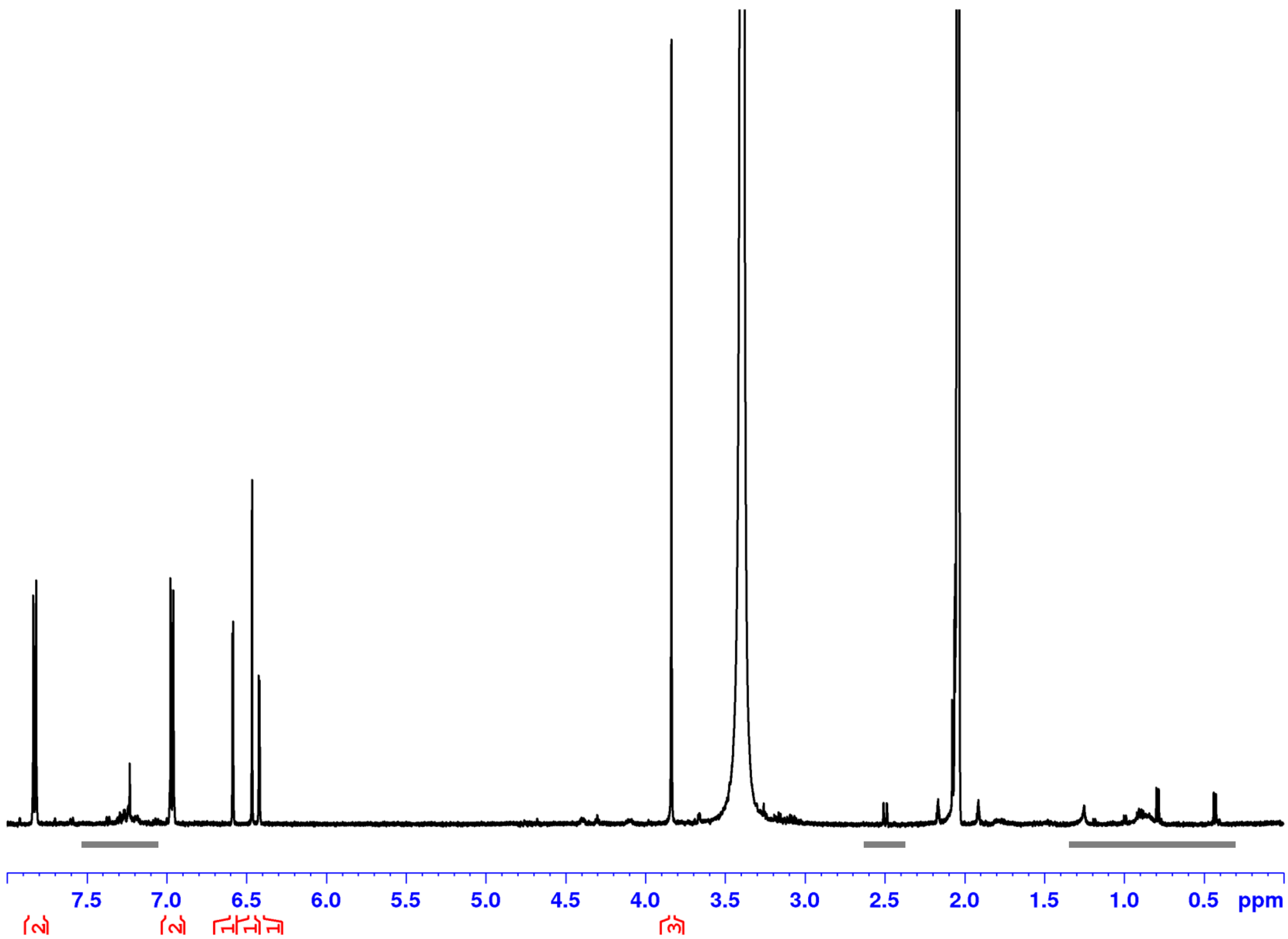


$^{1}$H NMR spectrum of 5-*O*-methyl apigenin in acetone-$d_6$ (grey bars indicate impurities)

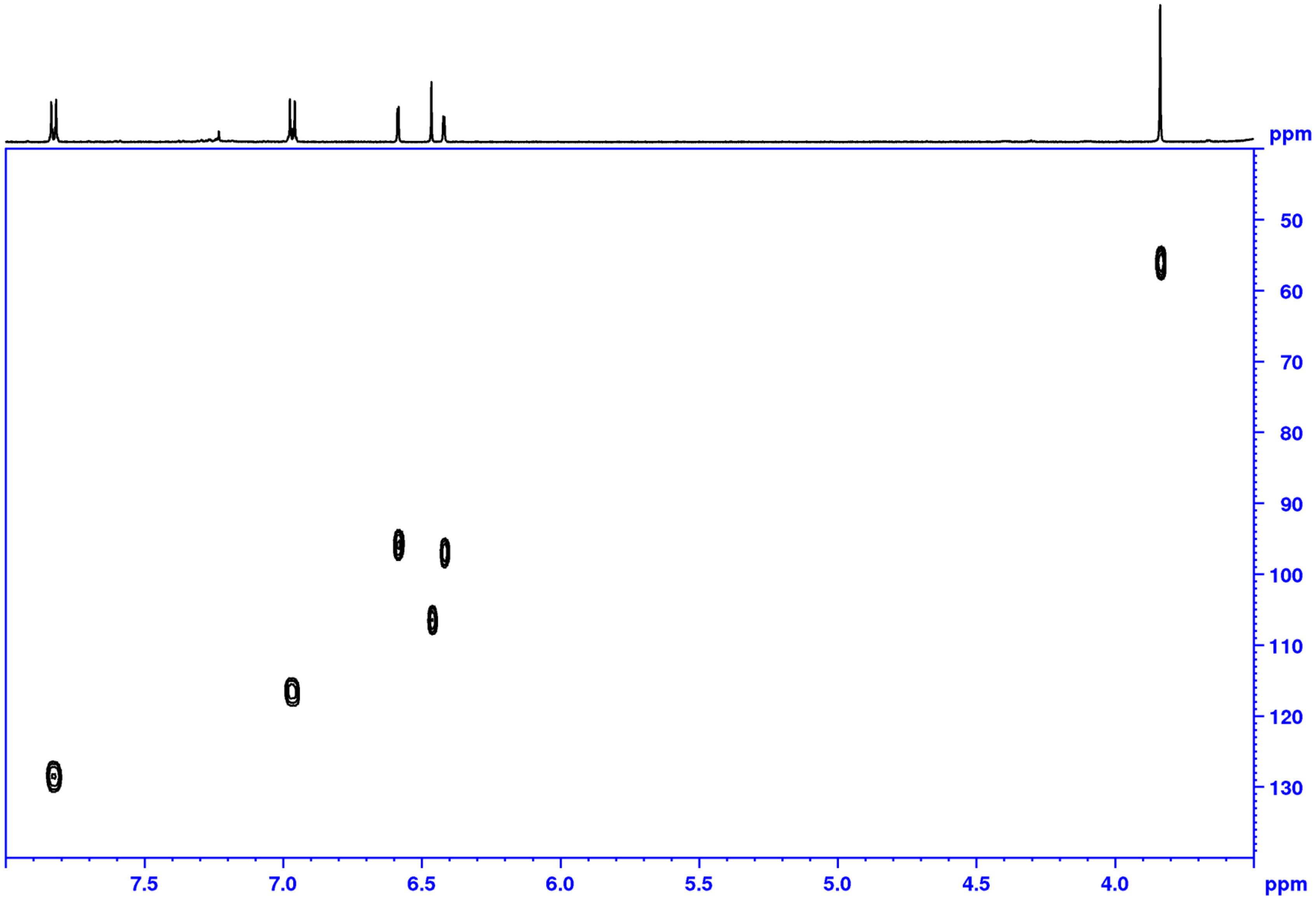


$^{1}H$-$^{13}C$ HSQC spectrum of 5-*O*-methyl apigenin in acetone-$d_6$

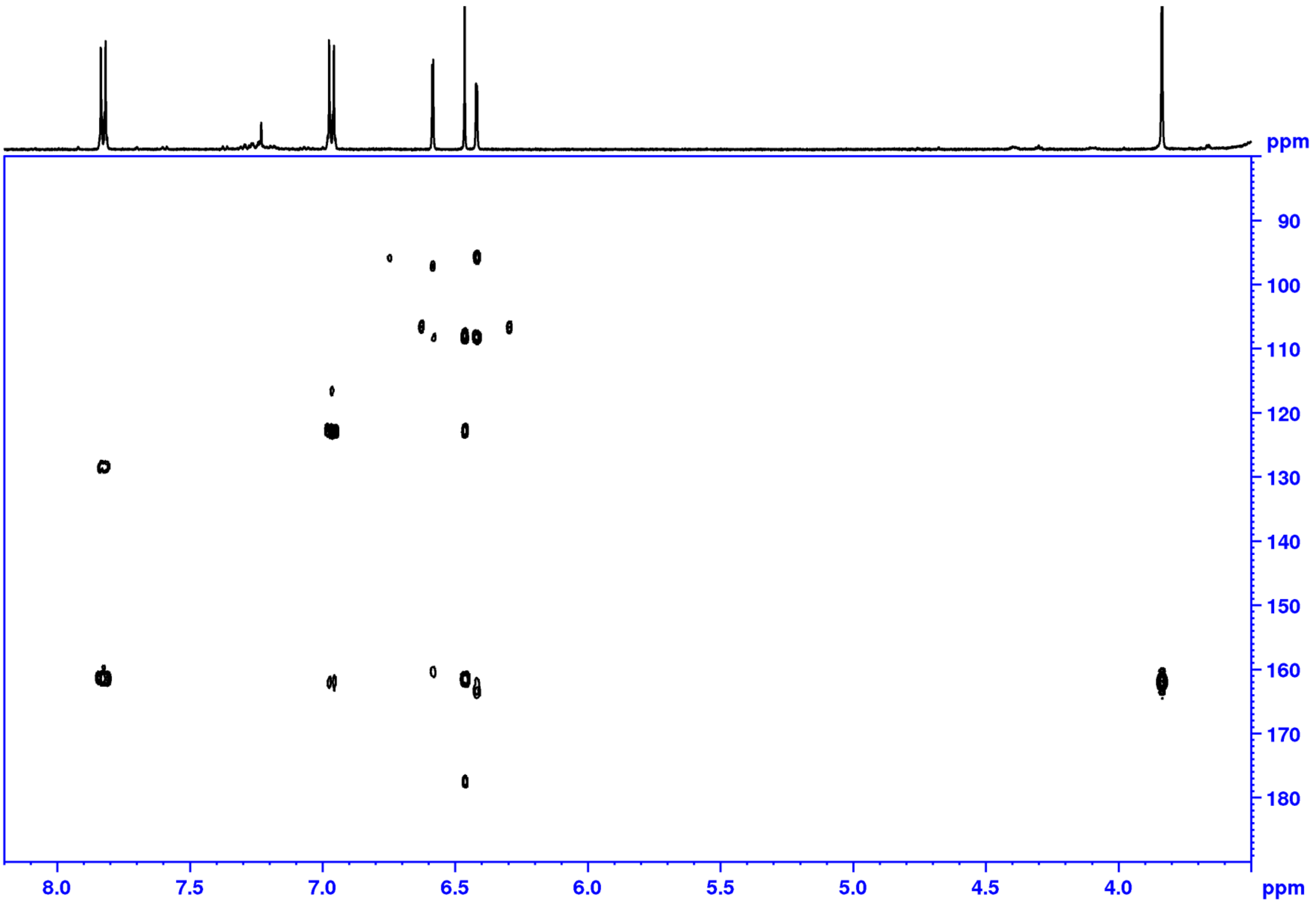


$^1H$-$^{13}C$ HMBC spectrum of 5-*O*-methyl apigenin in acetone-$d_6$

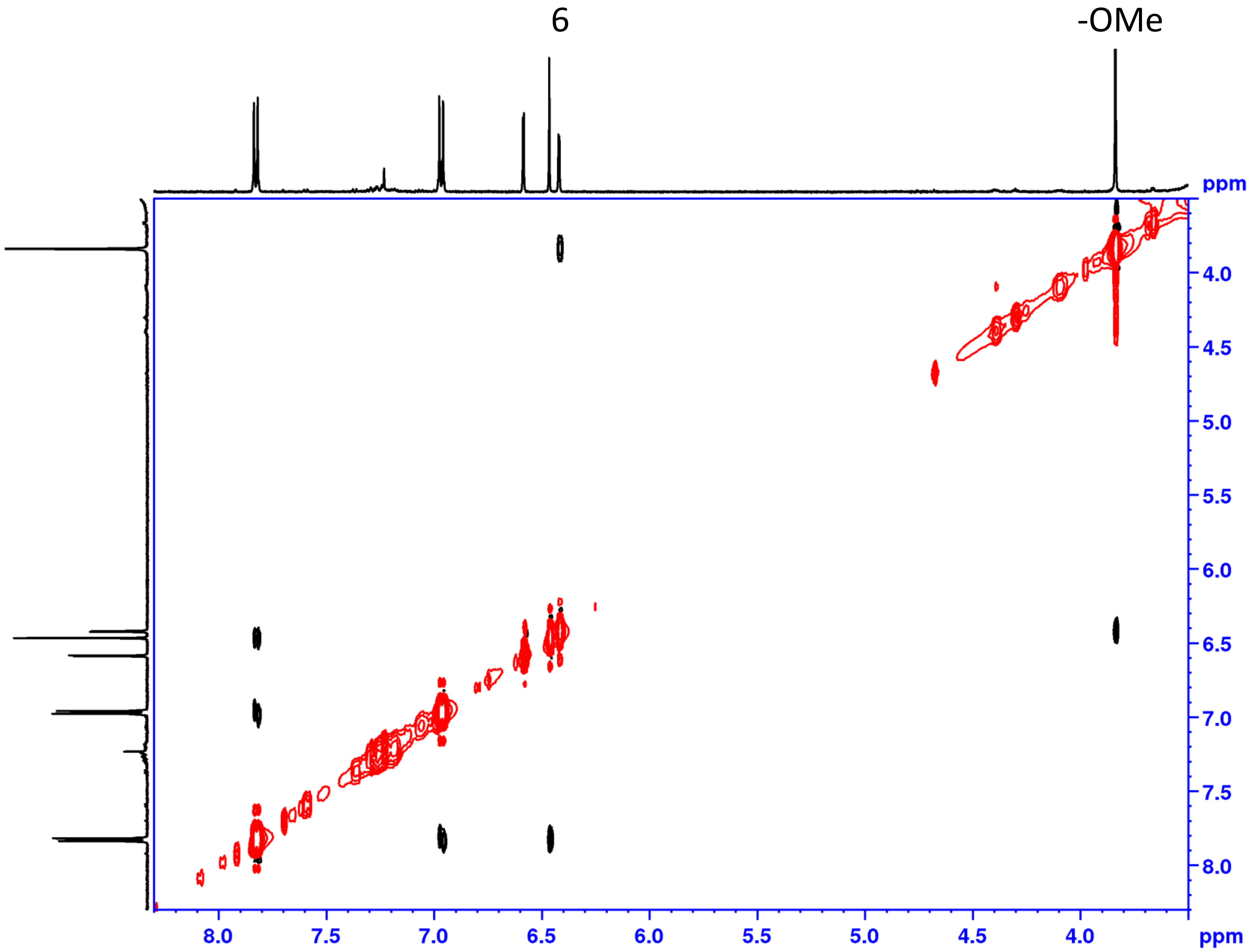


Detail of the $^{1}H$-$^{1}H$ ROESY spectrum of 5-*O*-methyl apigenin in acetone-$d_6$

# 7-*O*-methyl apigenin (genkwanin)

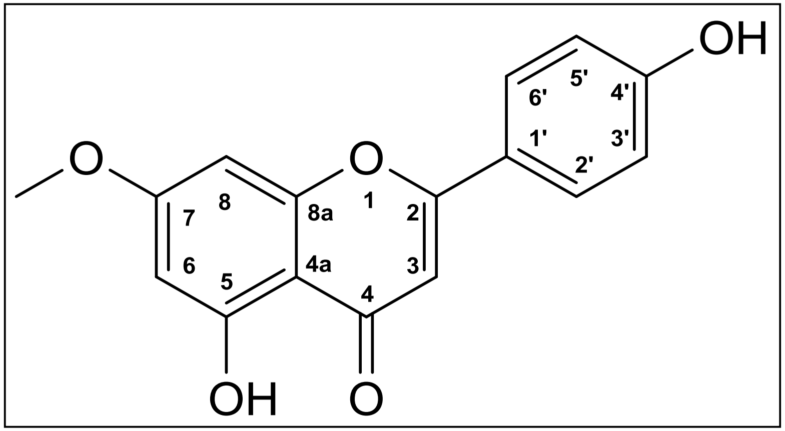


*Heterocycles* **2008**, 76(2), 1607-1615;
*Chinese Pharmaceutical Journal* (Taipei, Taiwan) **2006**, 58(1), 35-40;
*Alexandria Journal of Pharmaceutical Sciences* **2004**, 18(2), 165-170;
*Bulletin of the Korean Chemical Society* **2007**, 28(12), 2527-2530;
*Chemistry of Natural Compounds* **2005**, 41(2), 178-181.

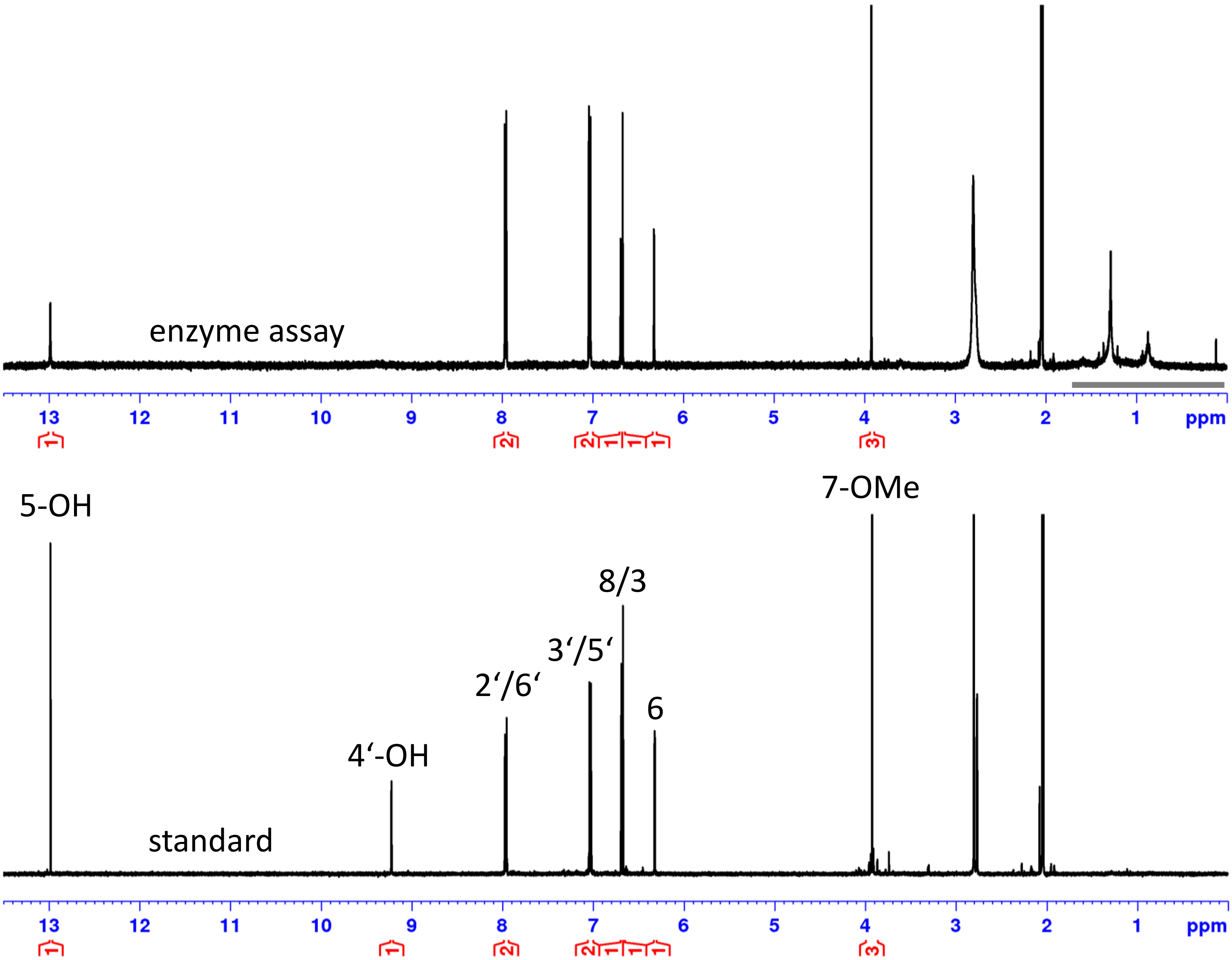


$^{1}$H NMR spectrum of 7-*O*-methyl apigenin in acetone-$d_6$ (grey bar indicates impurities)

# 5,7-*O*-dimethyl apigenin

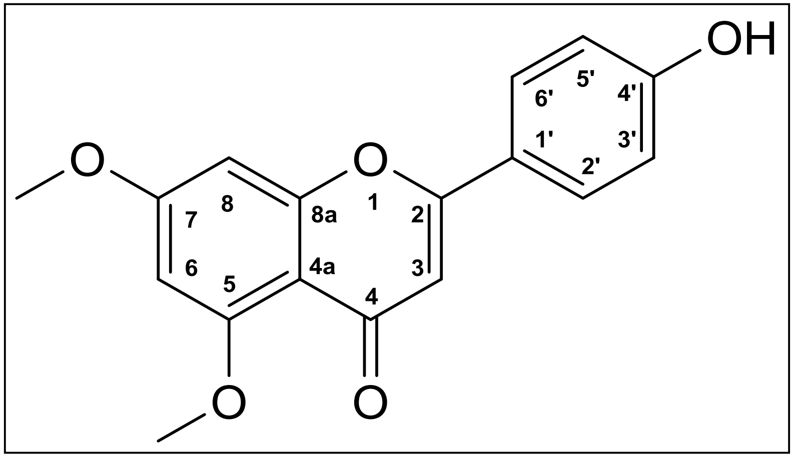


*Journal of the Chinese Chemical Society* (Taipei, Taiwan) **2004**, 51(1), 199-204;
*Bulletin of the Korean Chemical Society* **2007**, 28(12), 2527-2530;
*J. Med. Chem.* **2007**, 50(16), 3921-3927;
*J. Agr. Food Chem.* **2004**, 52(19), 5863-5868.

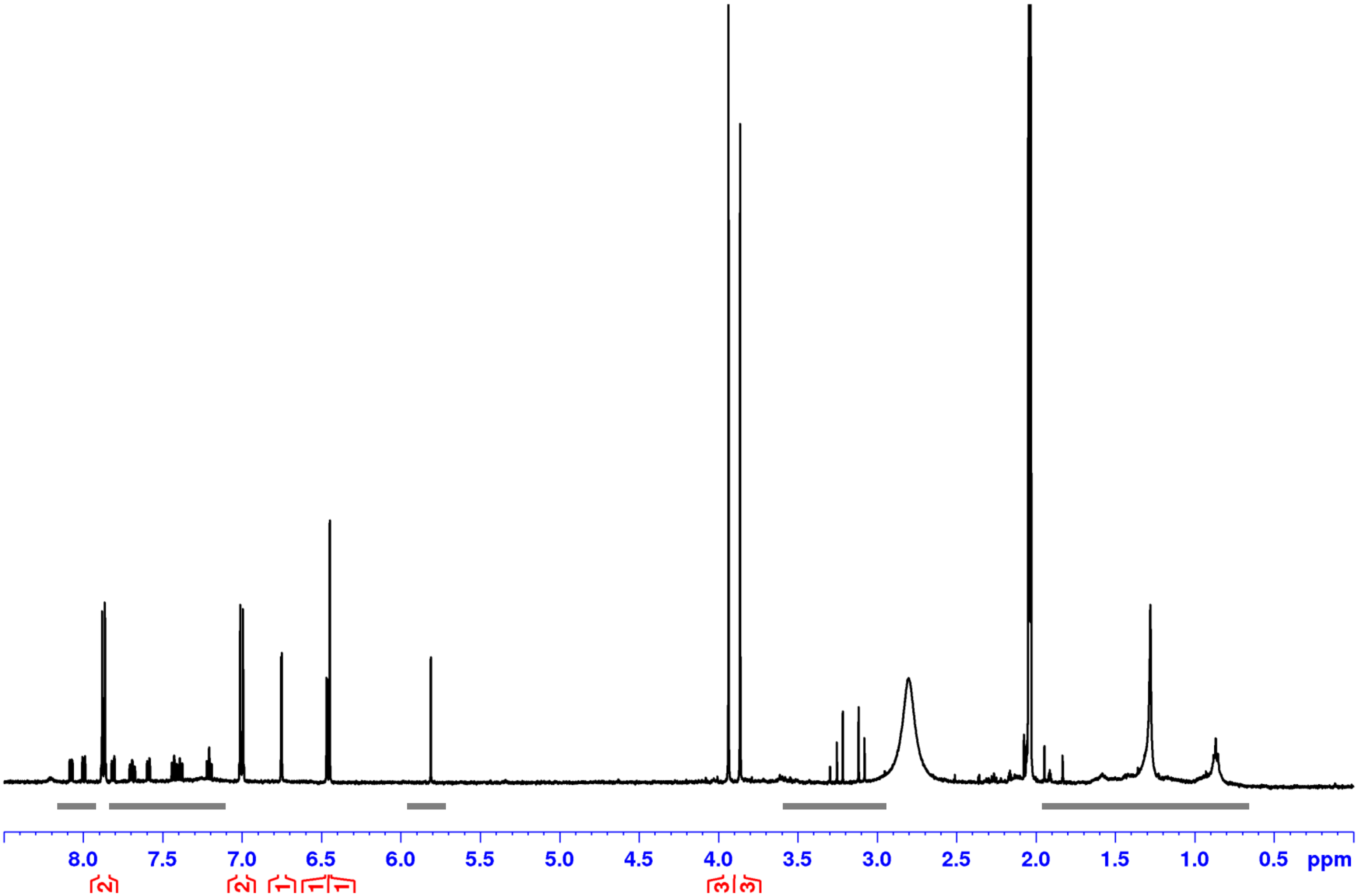


$^1$H NMR spectrum of 5,7-*O*-dimethyl apigenin in acetone-$d_6$ (grey bars indicate impurities)

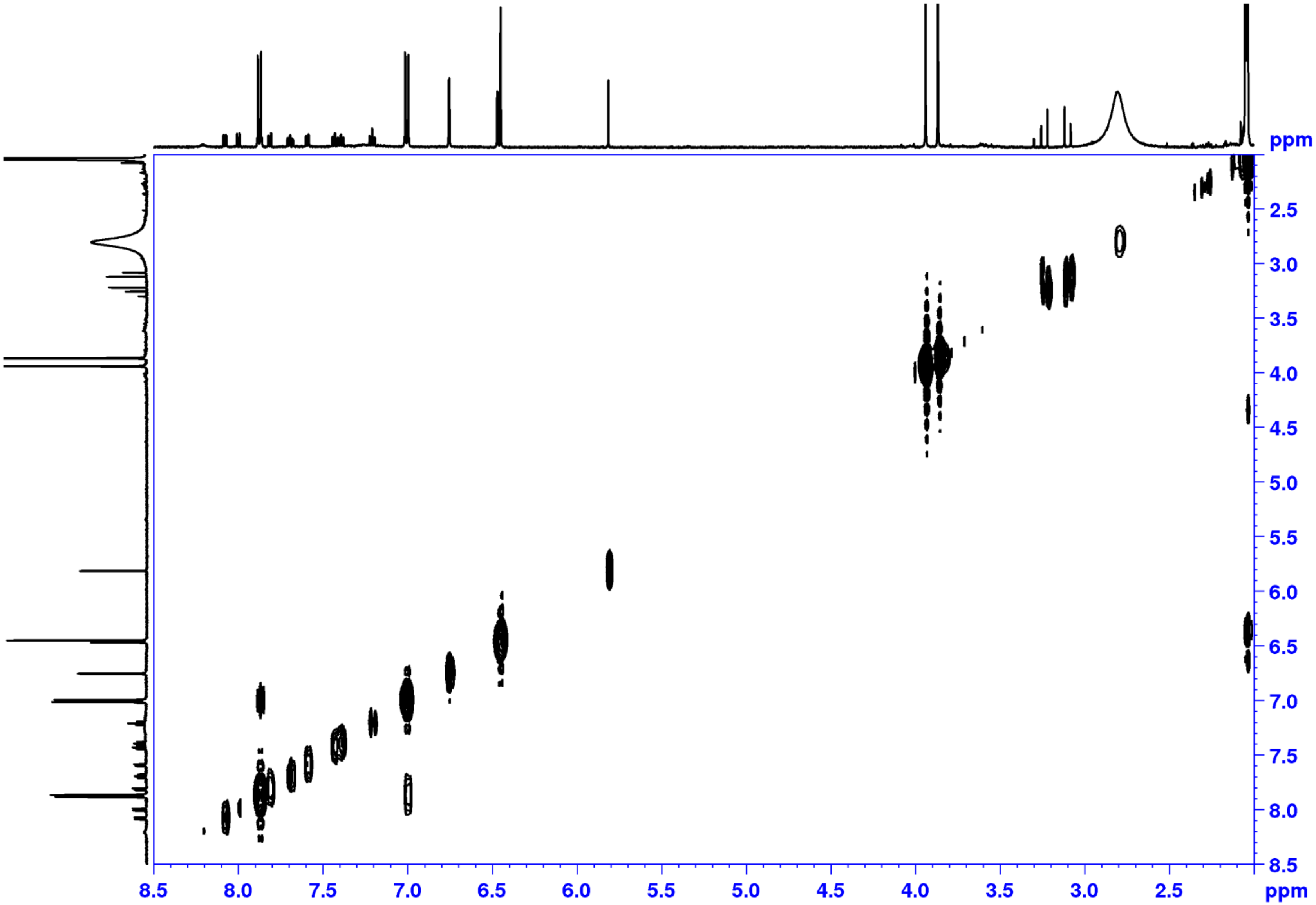


$^{1}H$-$^{1}H$ COSY spectrum of 5,7-*O*-dimethyl apigenin in acetone-$d_6$

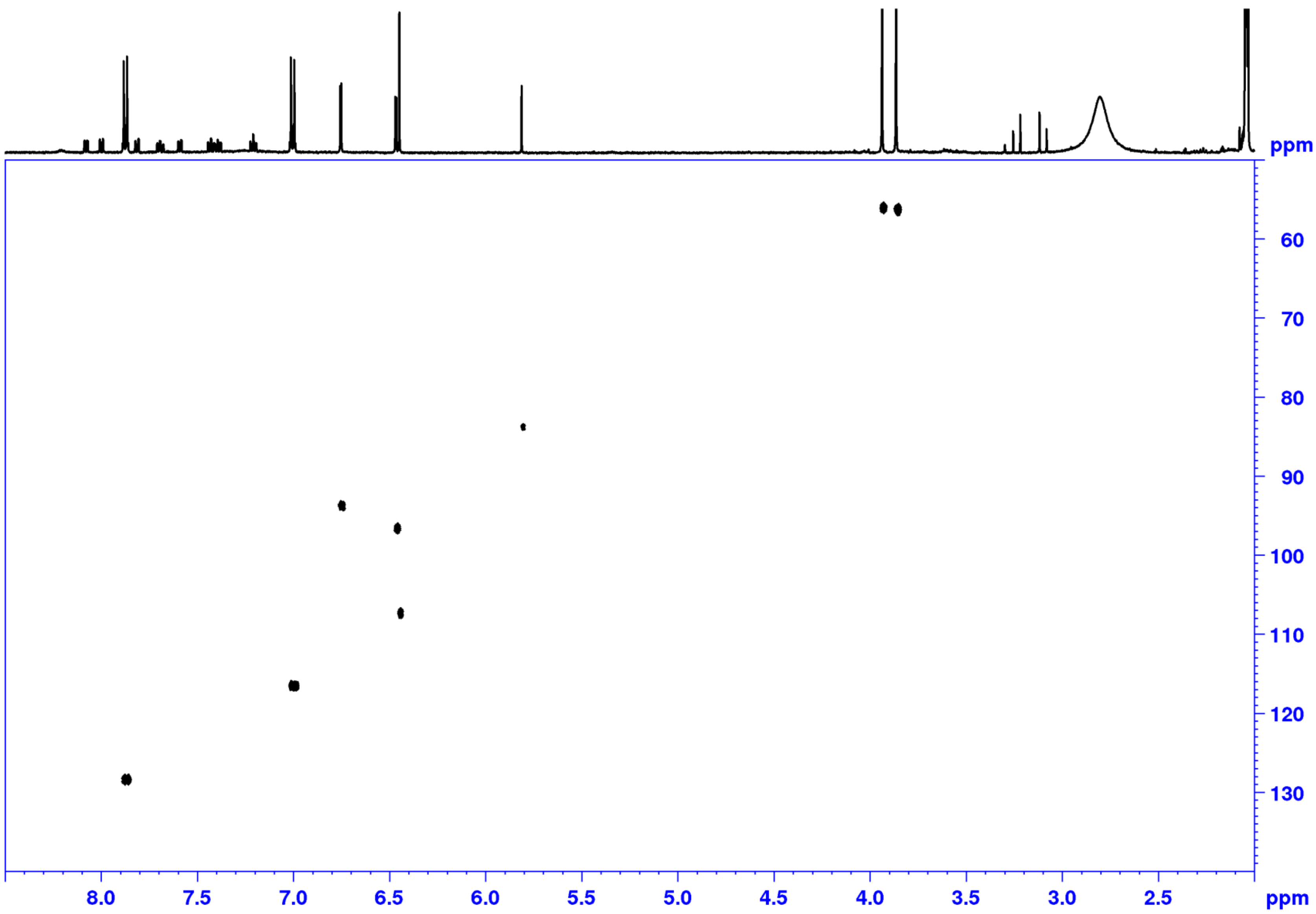


$^1H$-$^{13}C$ HSQC spectrum of 5,7-*O*-dimethyl apigenin in acetone-$d_6$

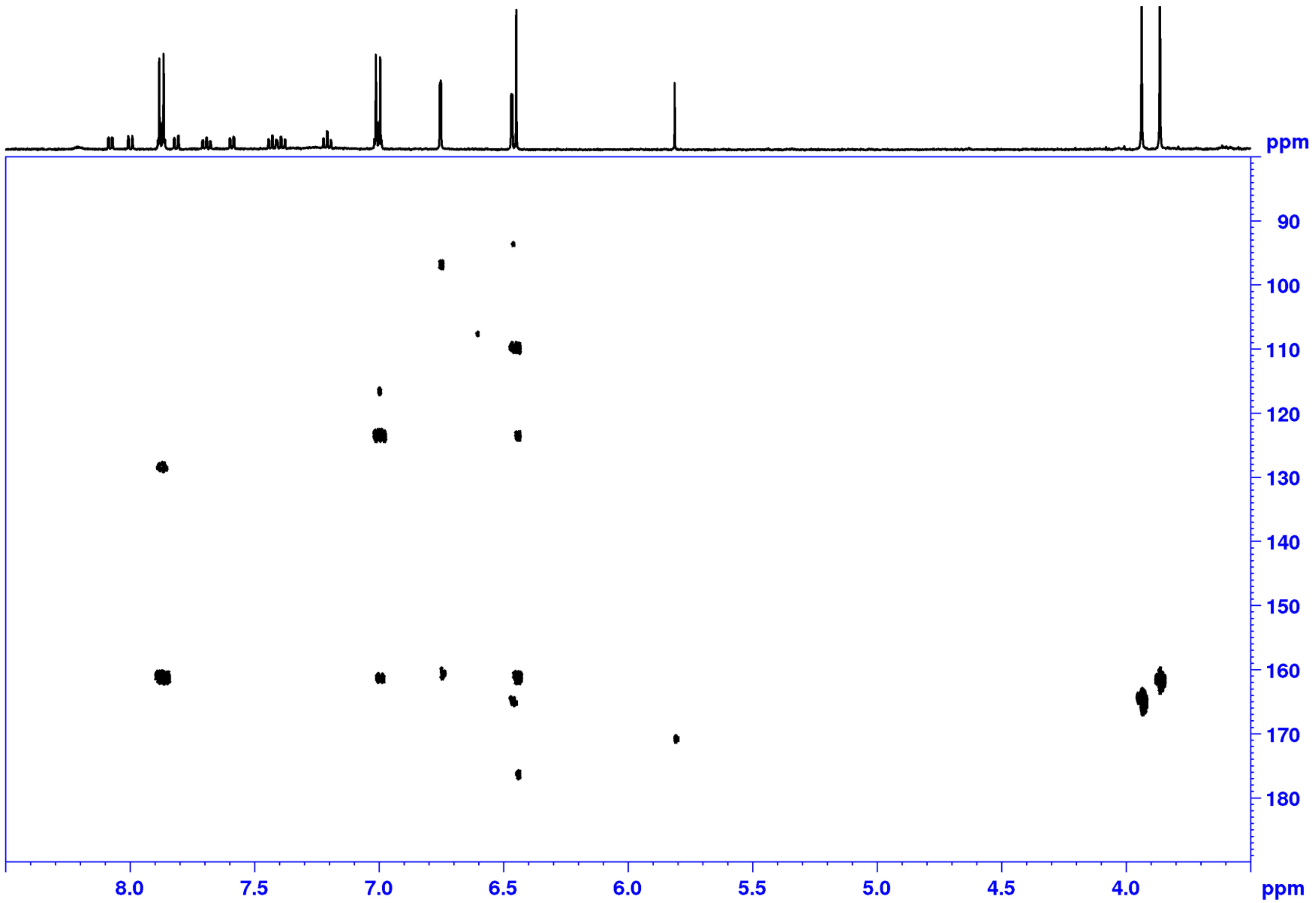


$^{1}H$-$^{13}C$ HMBC spectrum of 5,7-*O*-dimethyl apigenin in acetone-$d_6$

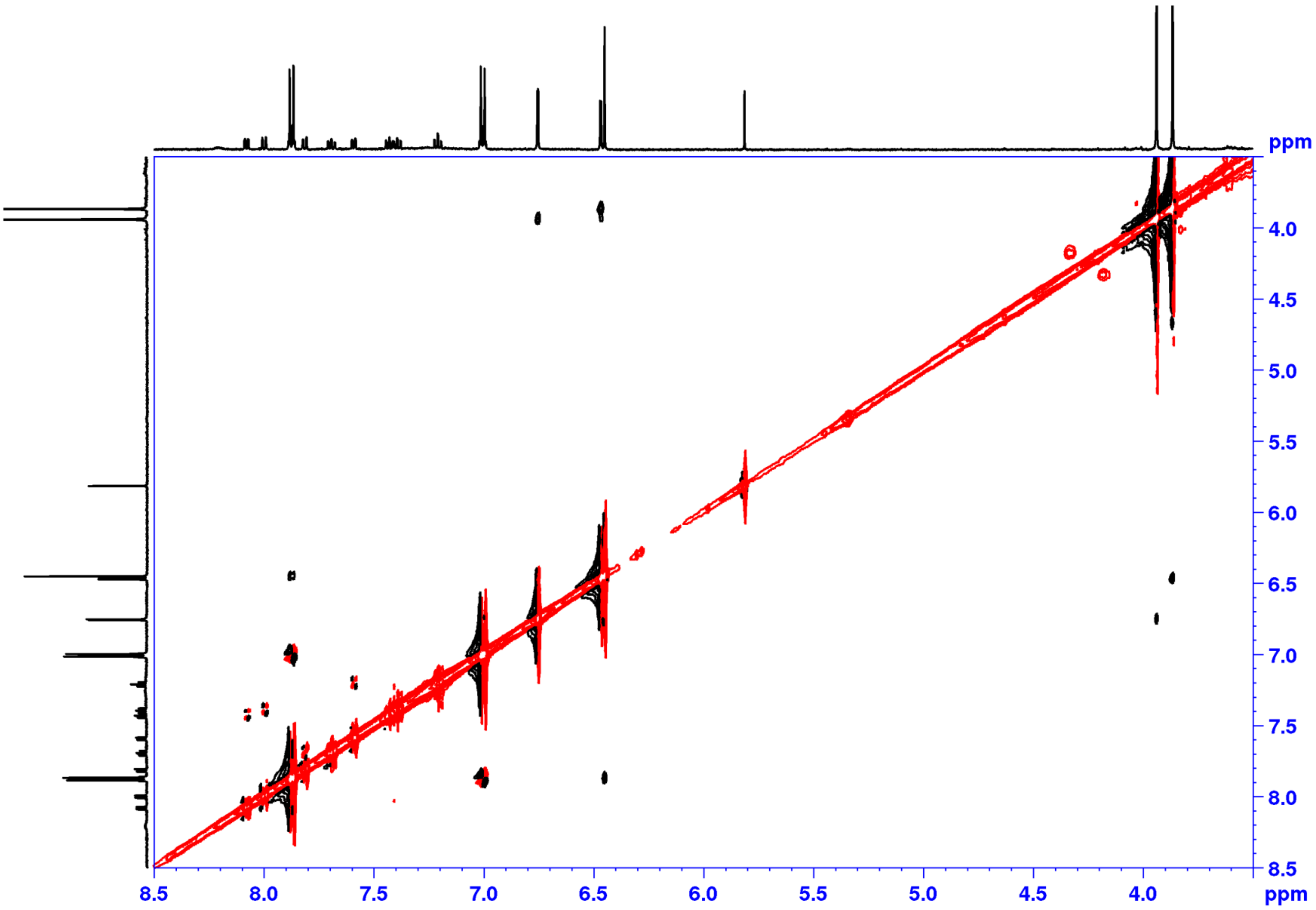


$^1H$-$^1H$ ROESY spectrum of 5,7-*O*-dimethyl apigenin in acetone-$d_6$

# 5-*O*-methyl scutellarein

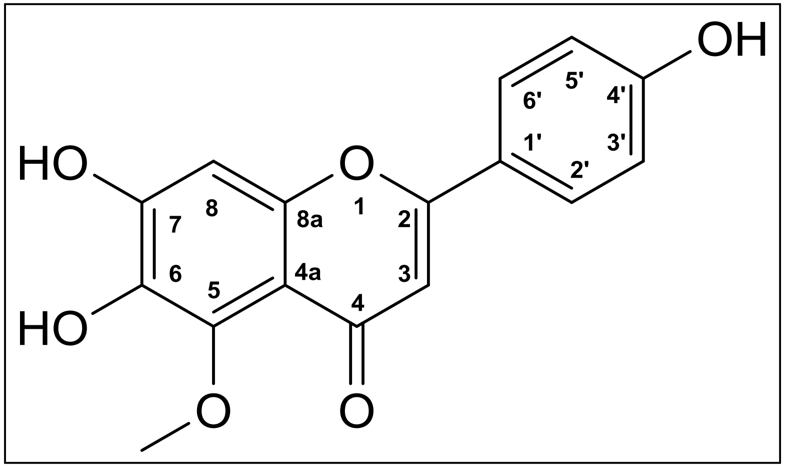

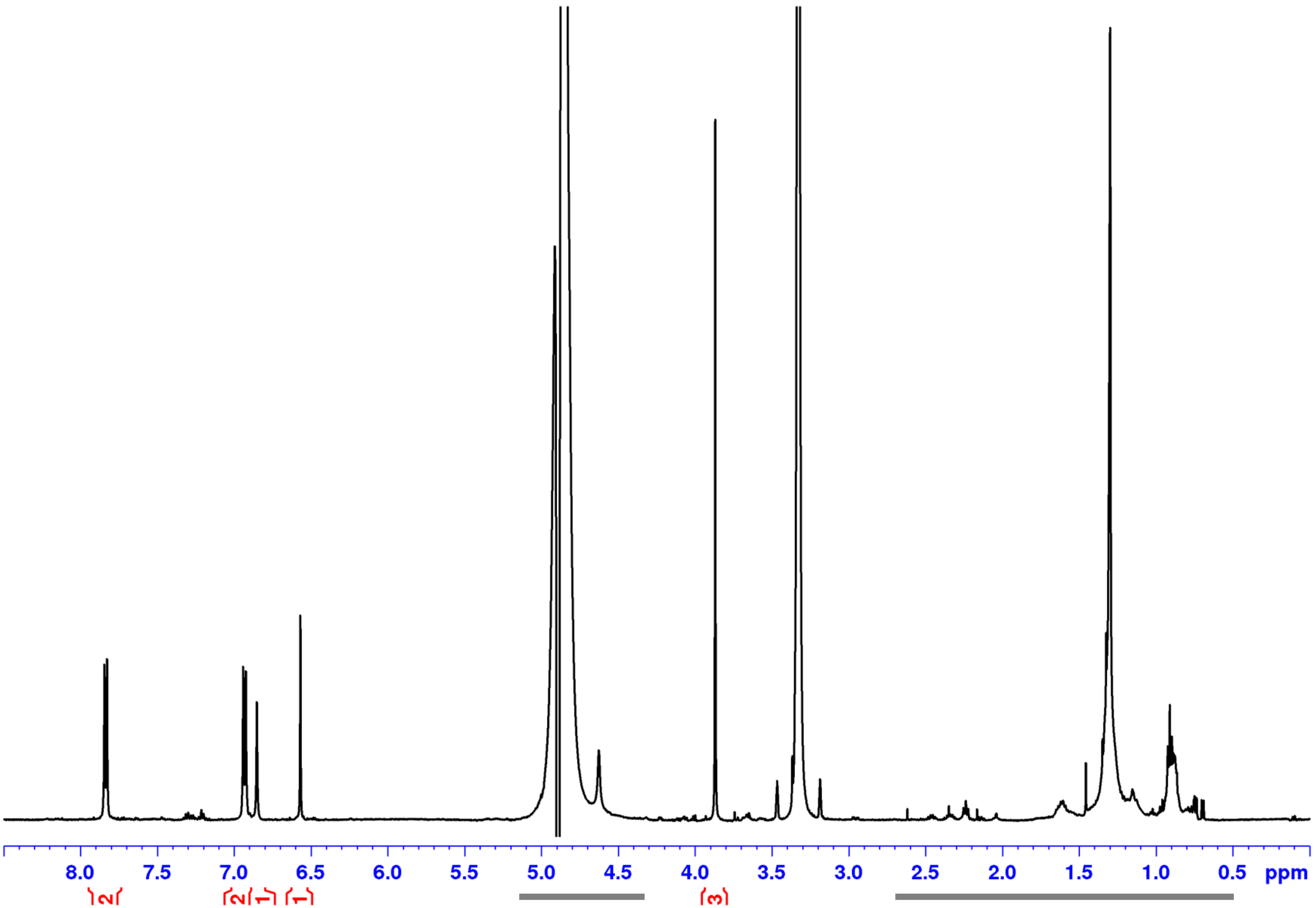


$^{1}H$ NMR spectrum of 5-*O*-methyl scutellarein in methanol-$d_3$ (grey bars indicate impurities)

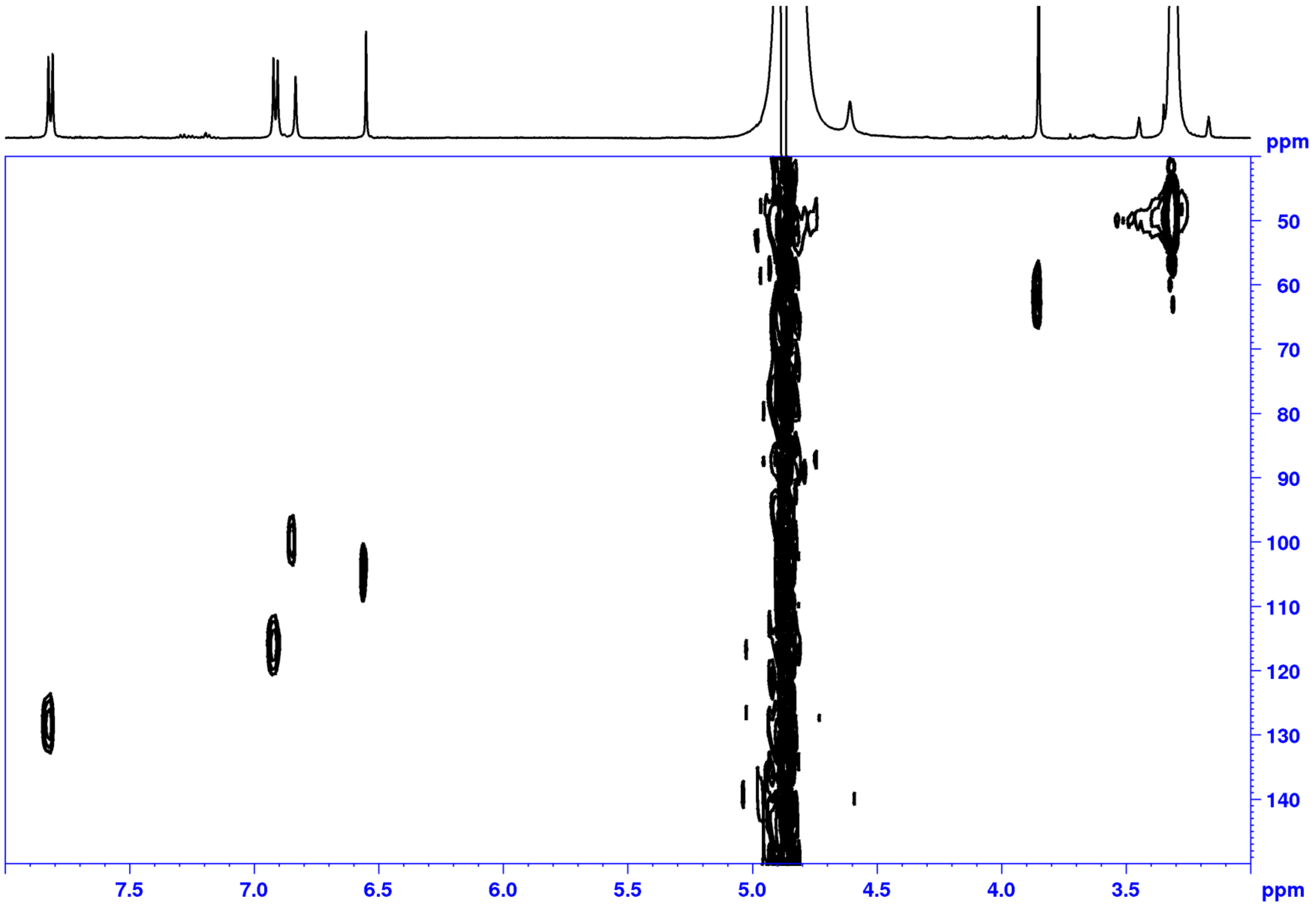


$^{1}H$-$^{13}C$ HSQC spectrum of 5-*O*-methyl scutellarein in methanol-$d_3$

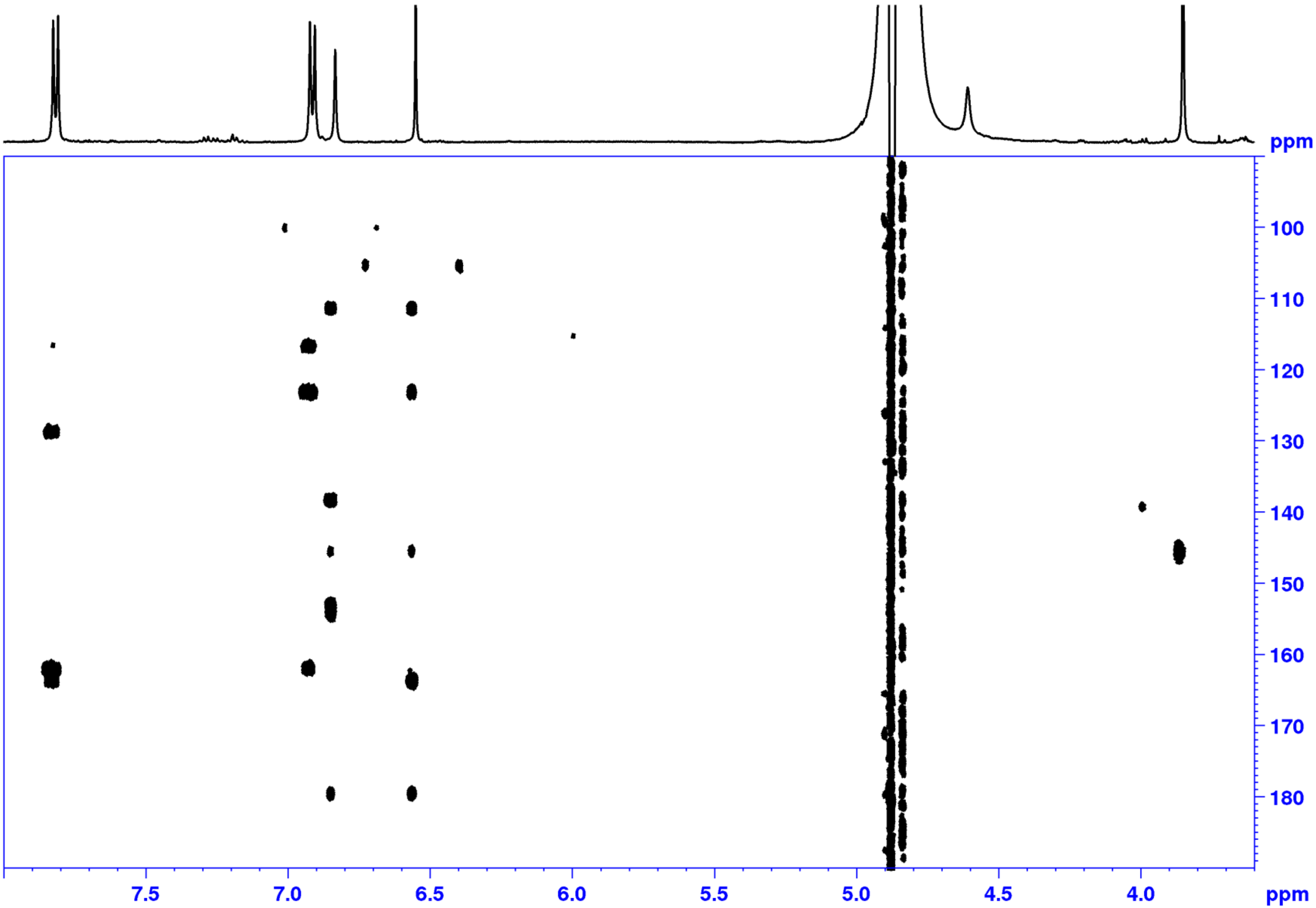


$^{1}H$-$^{13}C$ HMBC spectrum of 5-*O*-methyl scutellarein in methanol-$d_3$

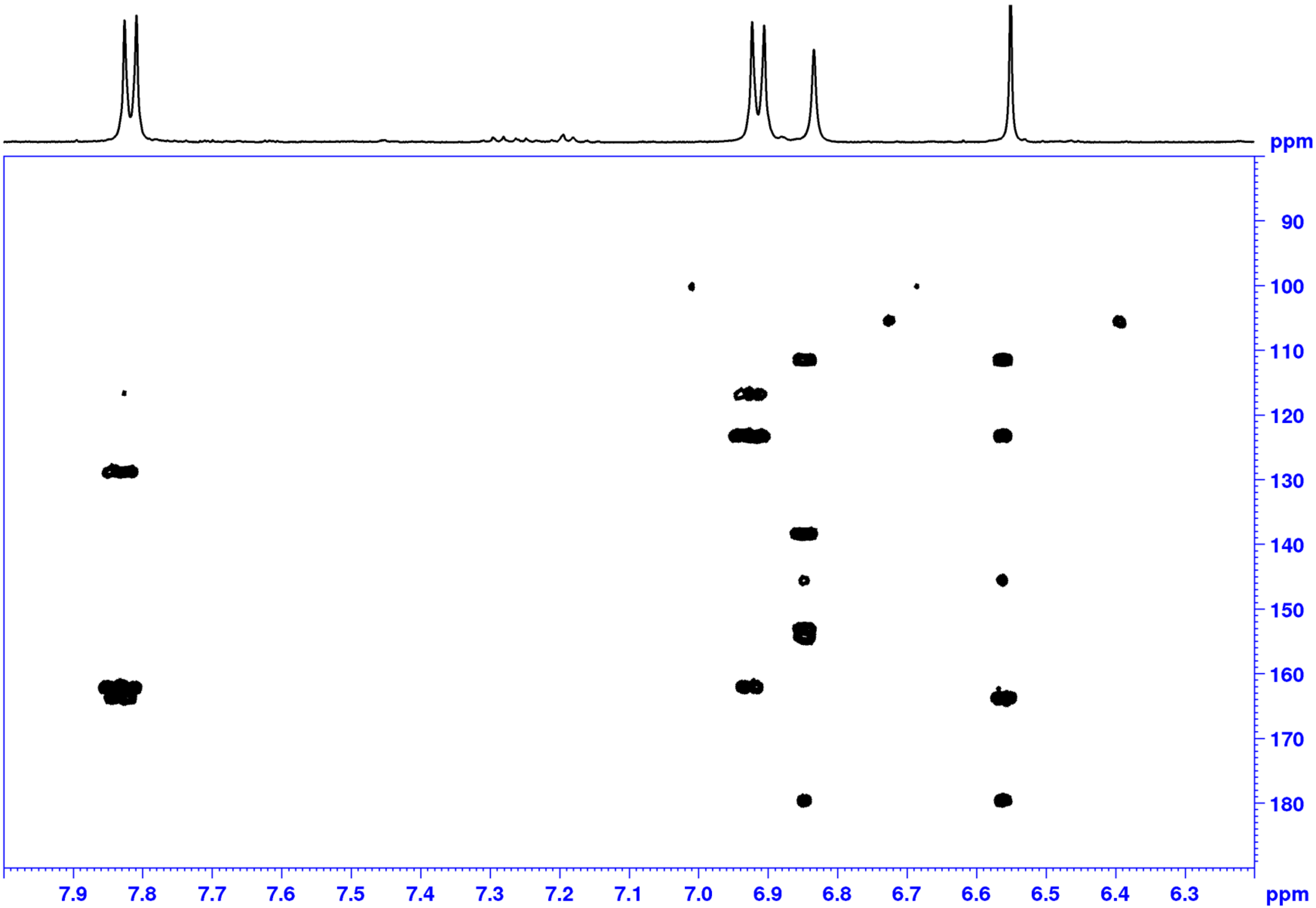


Detail of the $^{1}H$-$^{13}C$ HMBC spectrum of 5-*O*-methyl scutellarein in methanol-$d_3$

# 7-*O*-methyl scutellarein

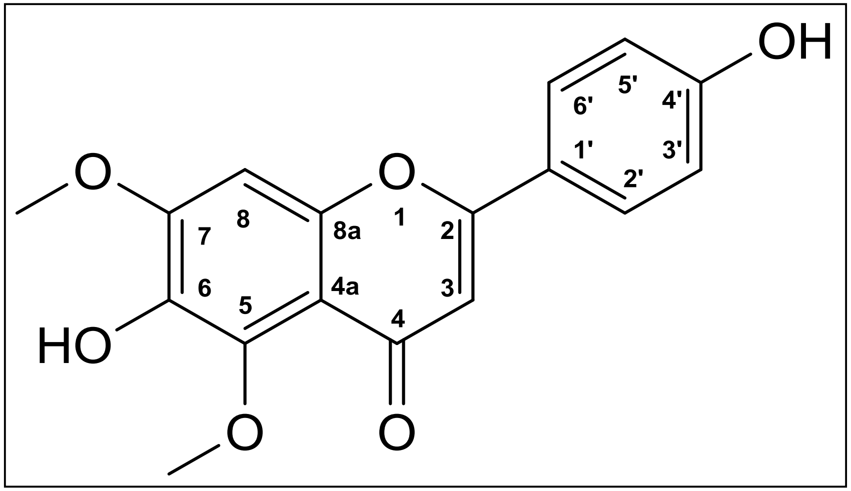

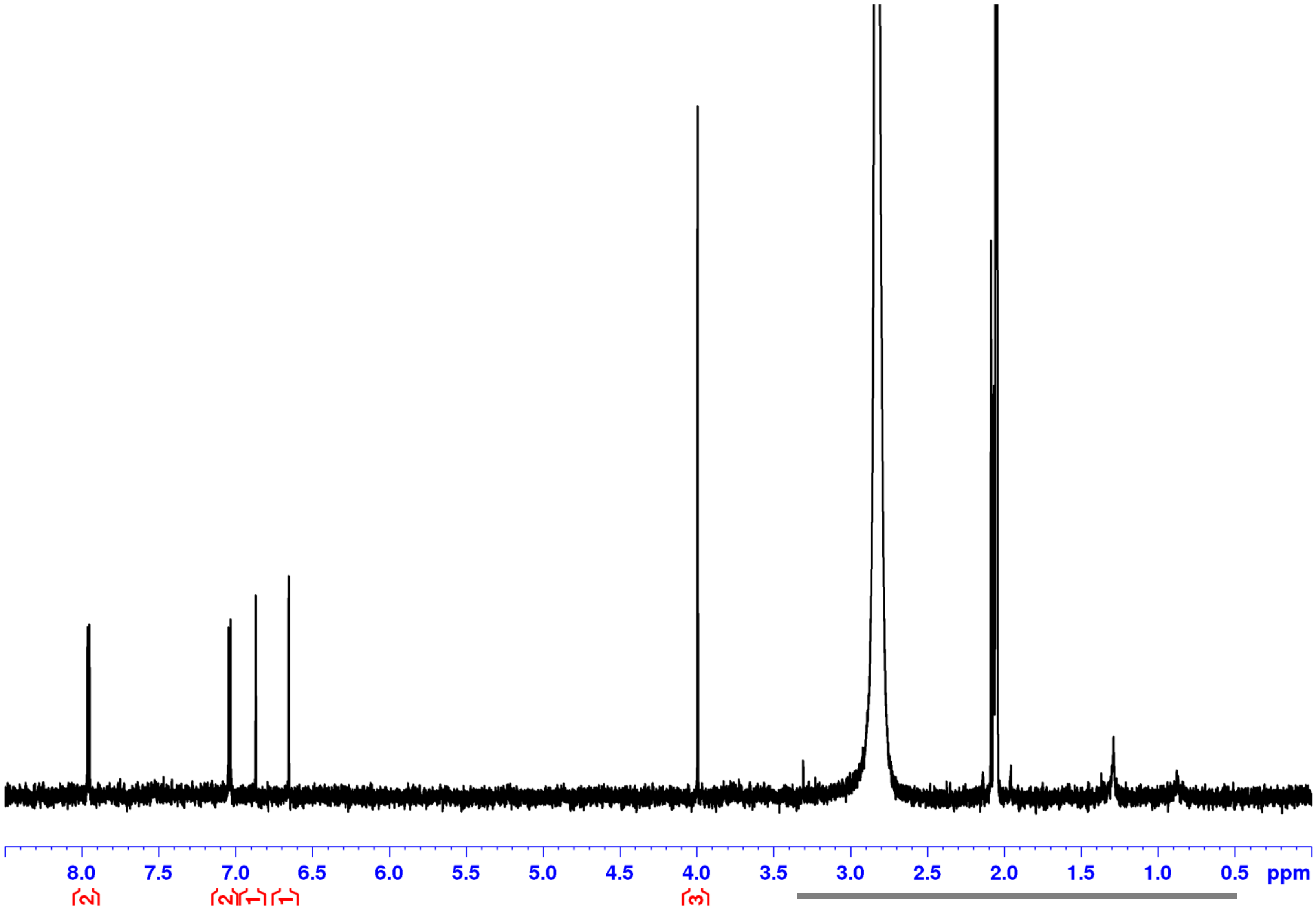


$^1$H NMR spectrum of 7-*O*-methyl scutellarein in acetone-$d_6$ (grey bar indicates impurities)

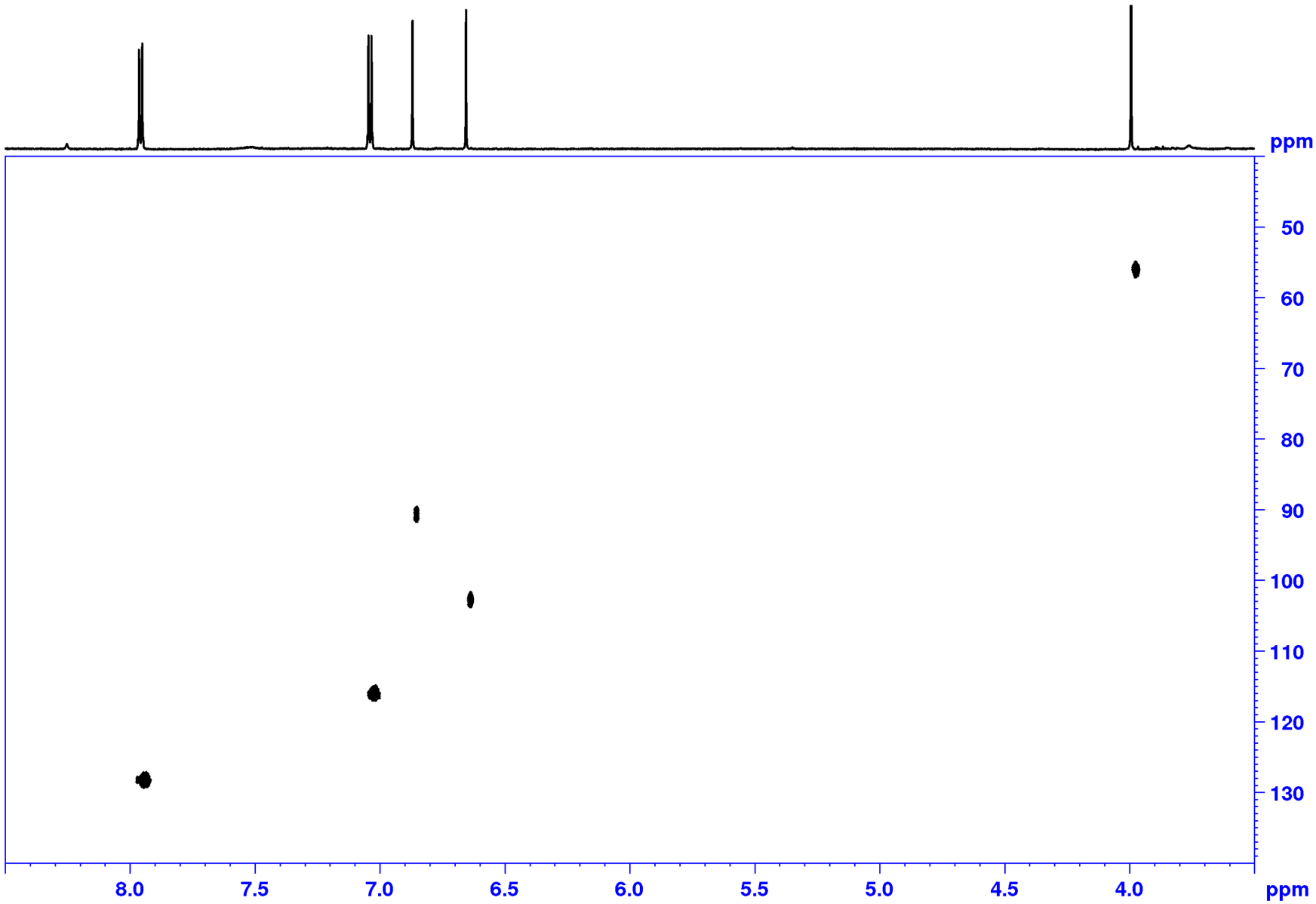


$^{1}H$-$^{13}C$ HSQC spectrum of 7-*O*-methyl scutellarein in acetone-$d_6$

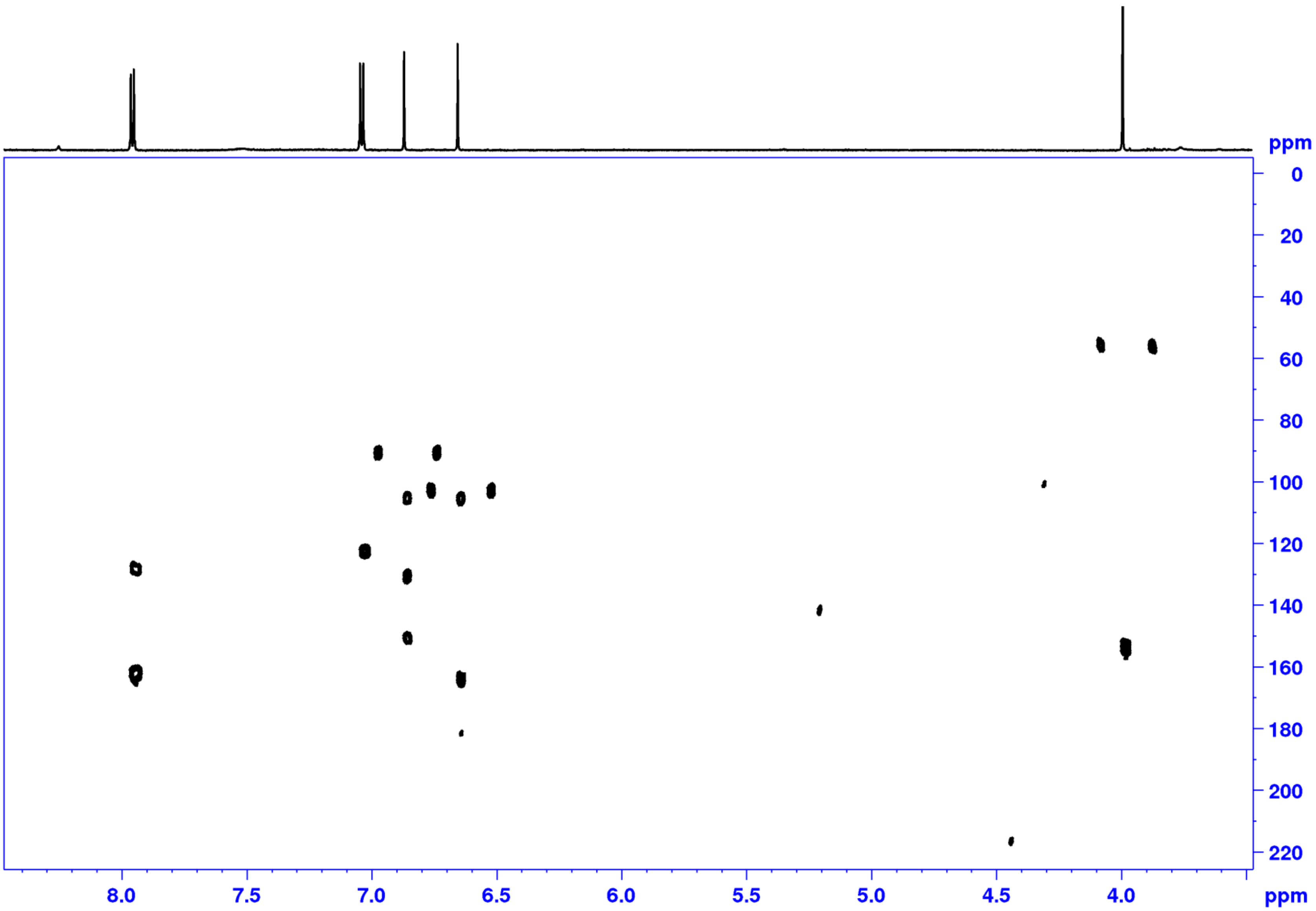


$^{1}H$-$^{13}C$ HMBC spectrum of 7-*O*-methyl scutellarein in acetone-$d_6$

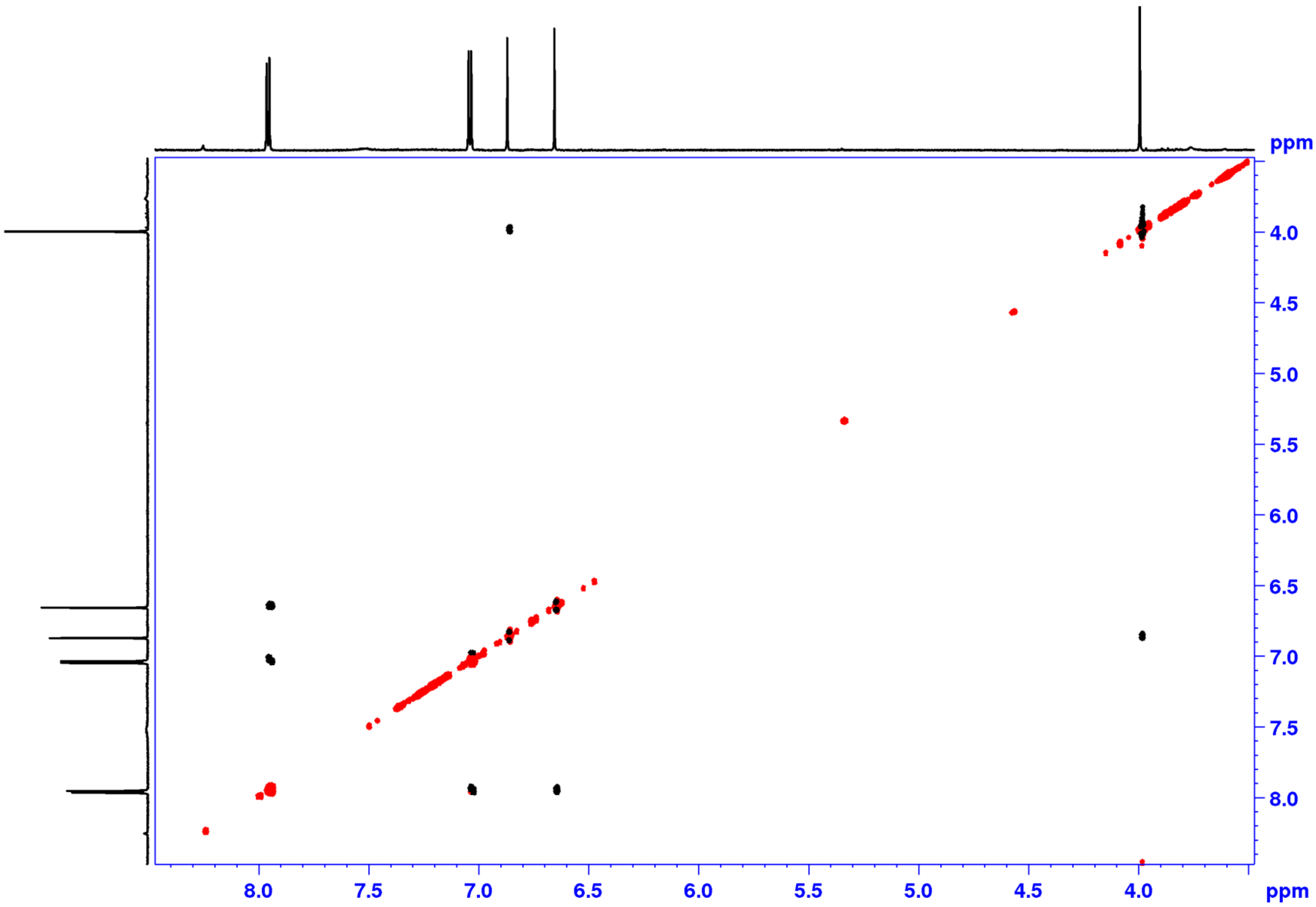


$^1H$-$^1H$ ROESY spectrum of 7-*O*-methyl scutellarein in acetone-$d_6$

# 5,7-*O*-dimethyl scutellarein

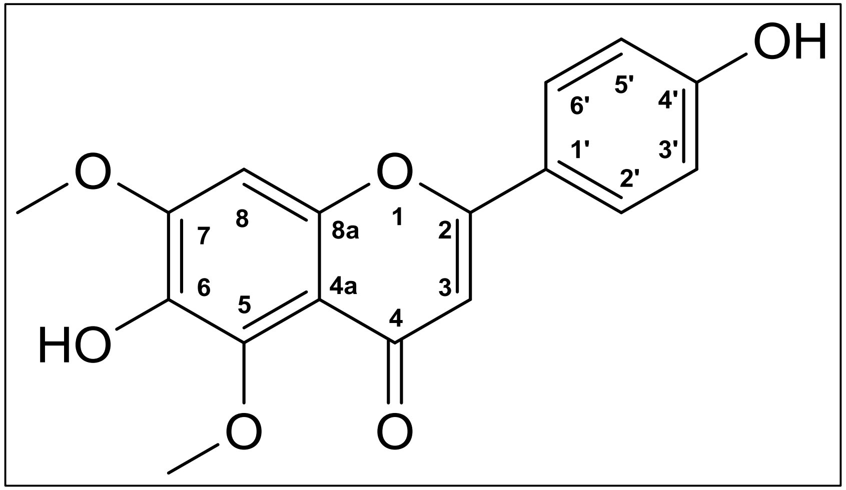

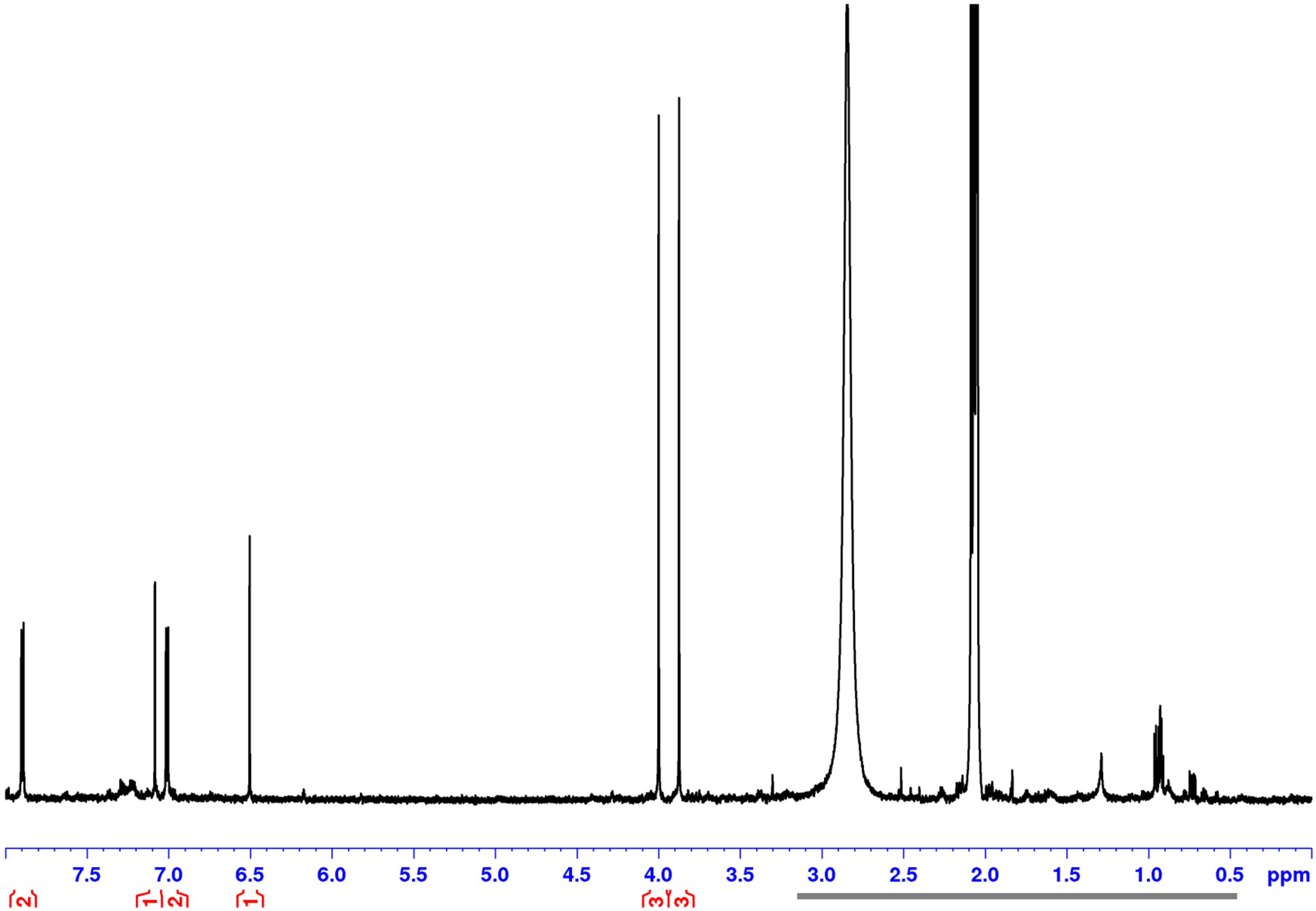


$^1H$ NMR spectrum of 5,7-*O*-dimethyl scutellarein in acetone-$d_6$ (grey bar indicates impurities)

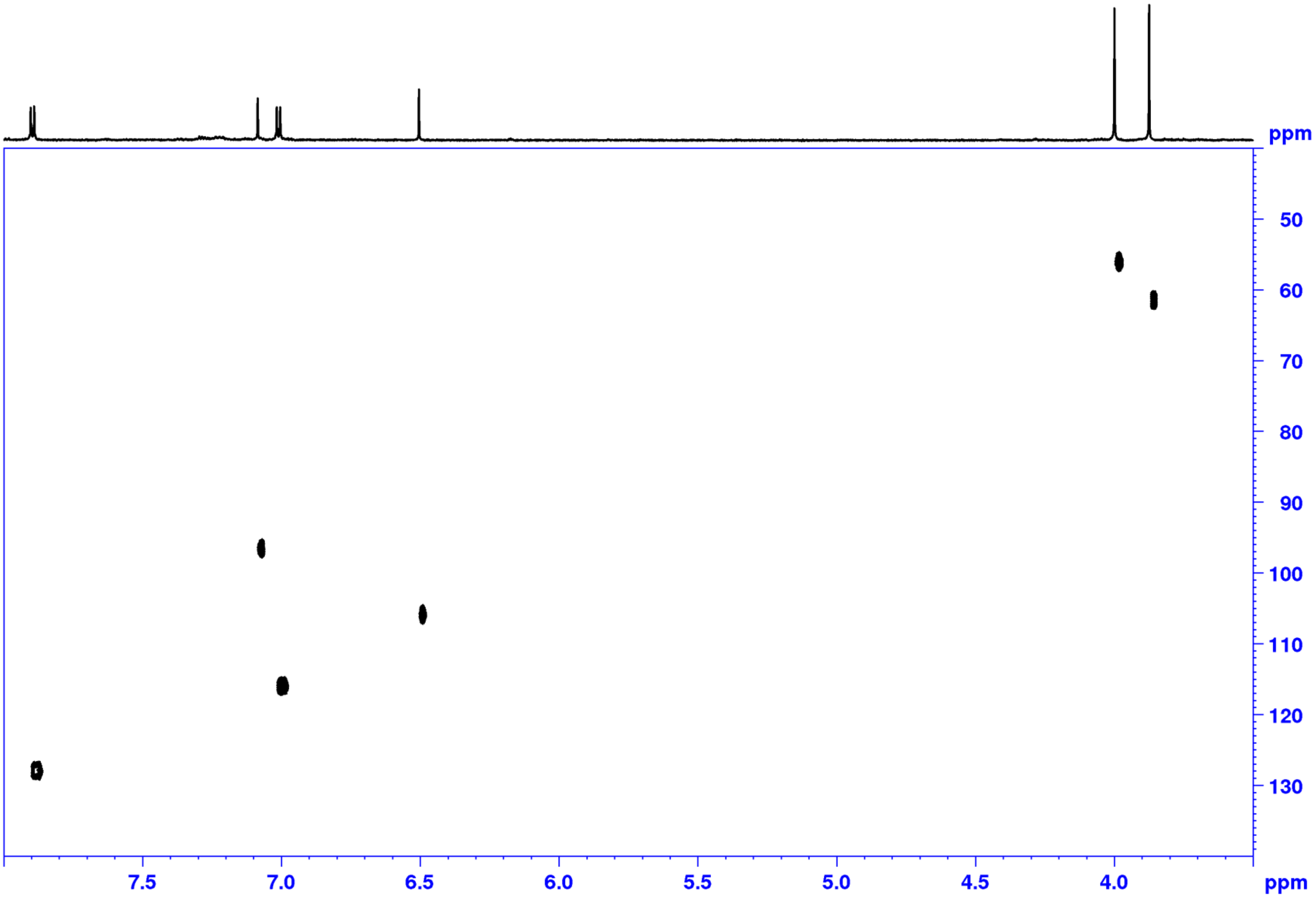


$^{1}H$-$^{13}C$ HSQC spectrum of 5,7-*O*-dimethyl scutellarein in acetone-$d_6$

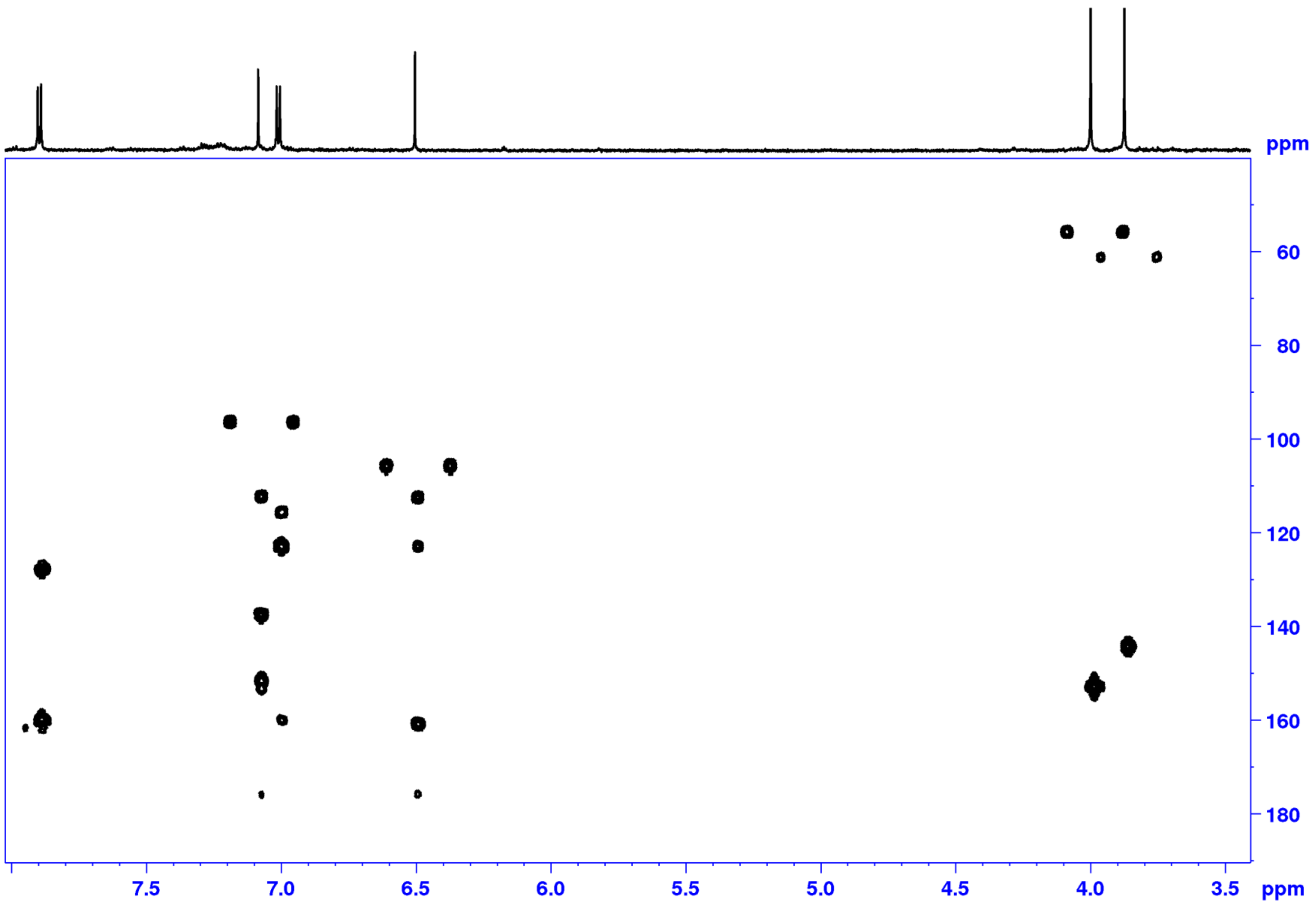


$^{1}H$-$^{13}C$ HMBC spectrum of 5,7-*O*-dimethyl scutellarein in acetone-$d_6$

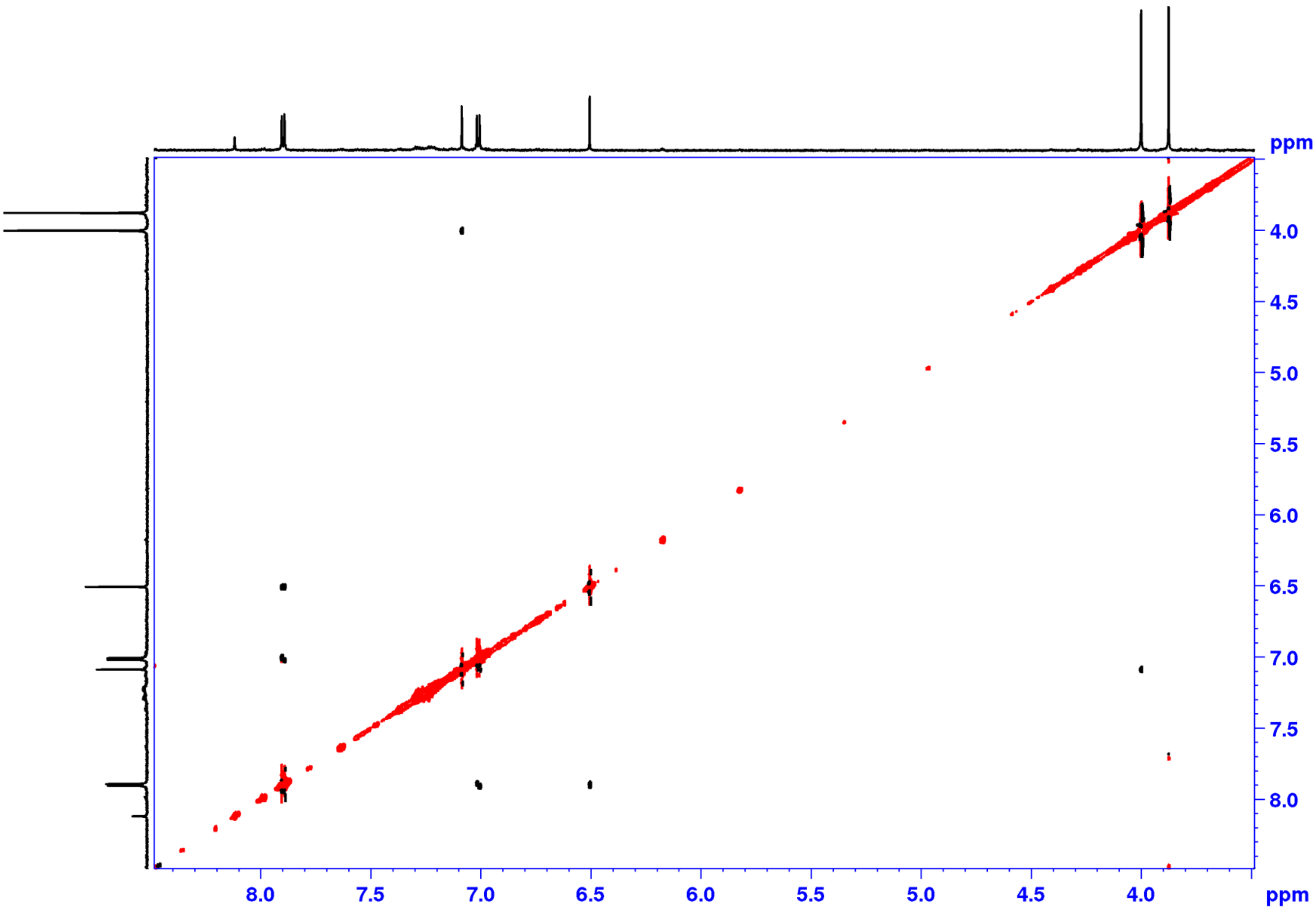


$^1H$-$^1H$ ROESY spectrum of 5,7-*O*-dimethyl scutellarein in acetone-$d_6$

# 3-*O*-methyl kaempferol (isokaempferide)

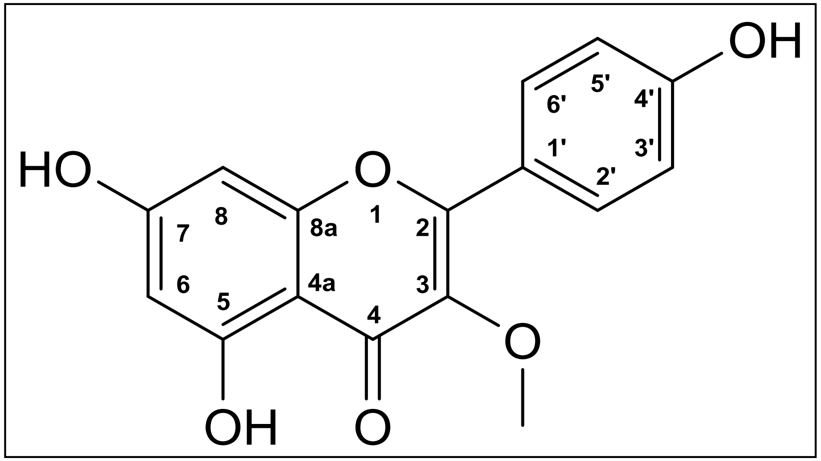


*Archives of Pharmacal Research* **2003**, 26(12), 1018-1023;
*Natural Medicines* (Tokyo, Japan) **2003**, 57(6), 250-252;
*Archives of Pharmacal Research* **2011**, 34(8), 1289-1296.

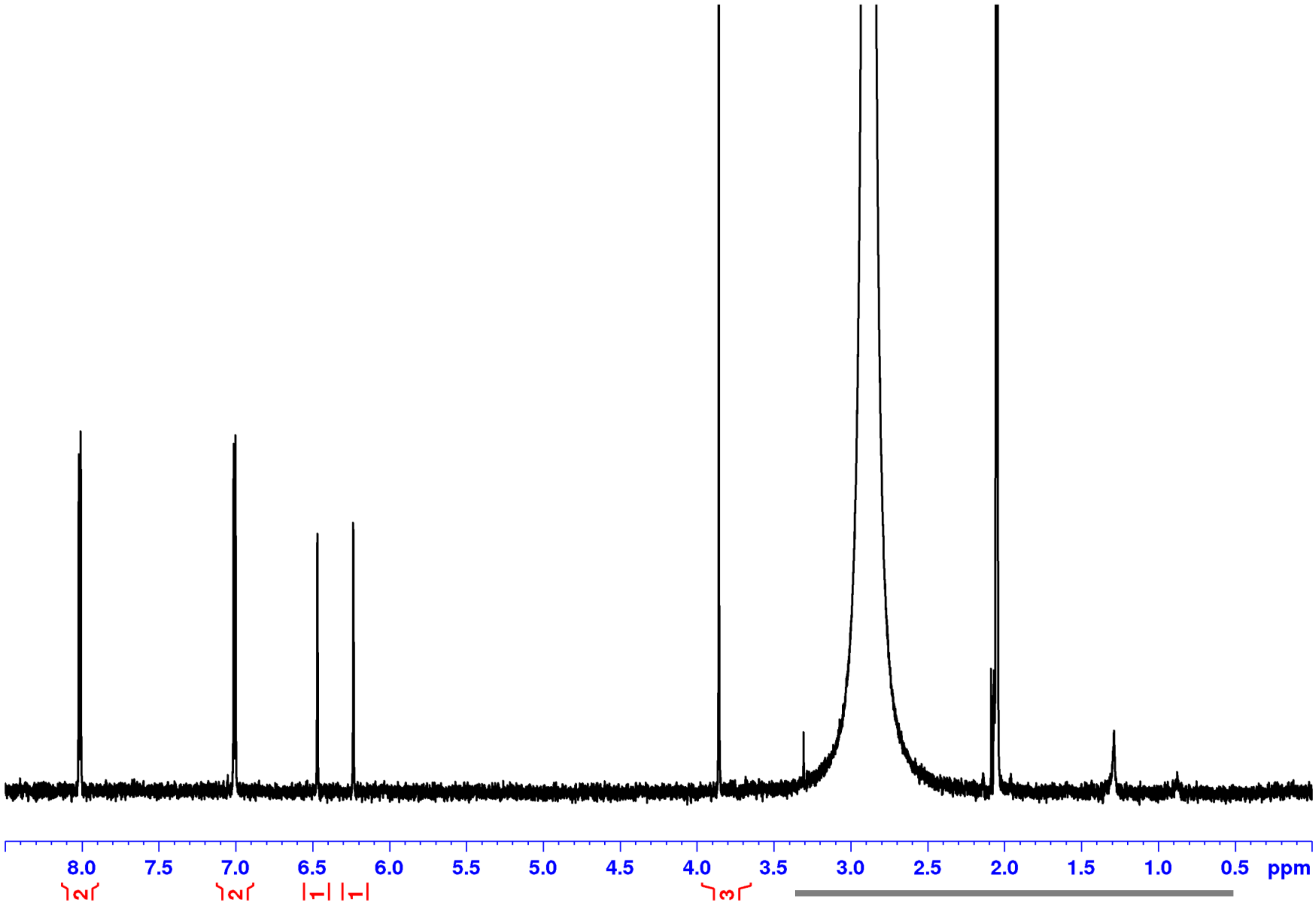


$^1H$ NMR spectrum of kaempferol-3-methyl ether (isokampferide) in acetone-$d_6$
(grey bar indicates impurities)

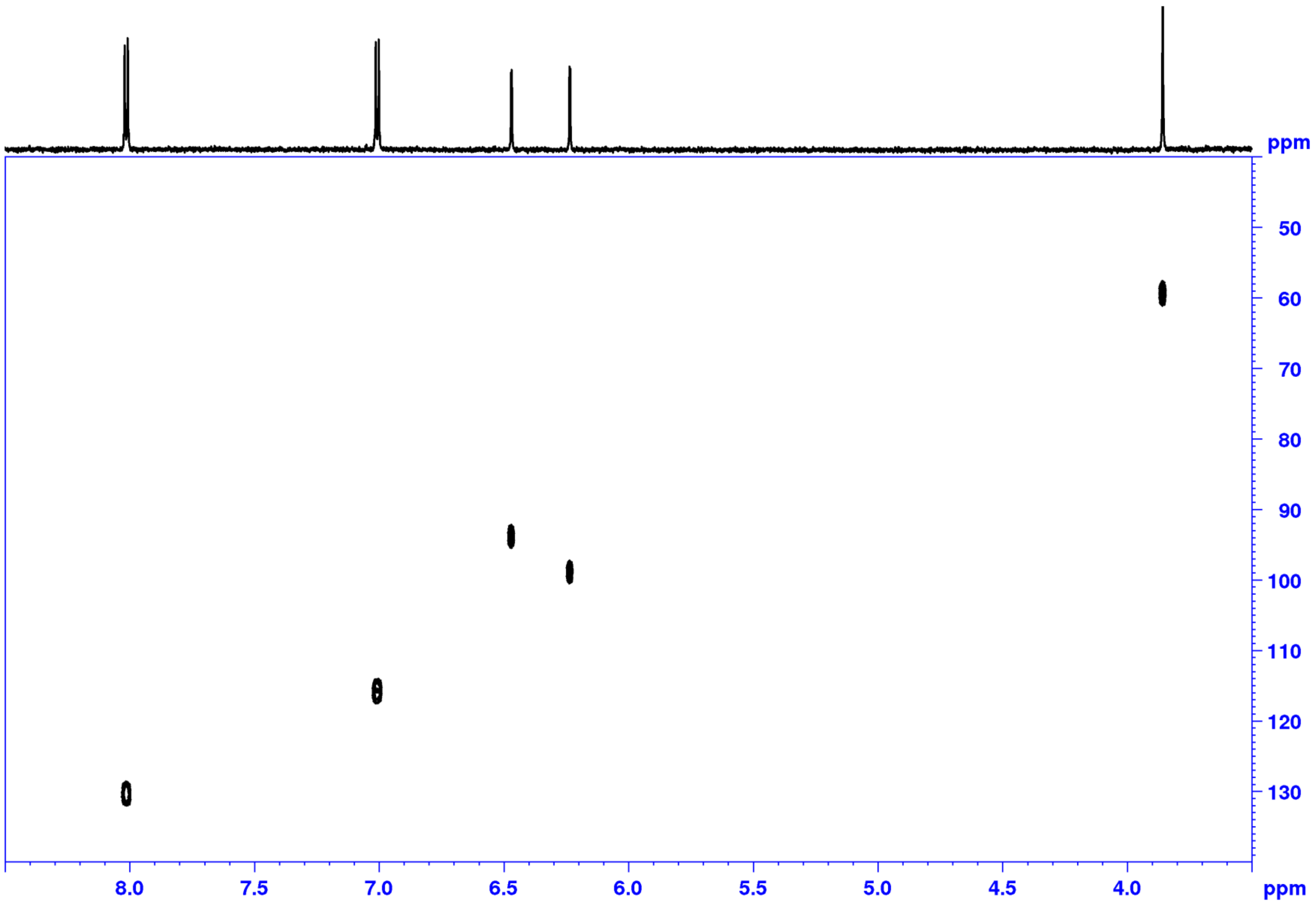


$^{1}H$-$^{13}C$ HSQC spectrum of kaempferol-3-methyl ether (isokampferide) in acetone-$d_6$

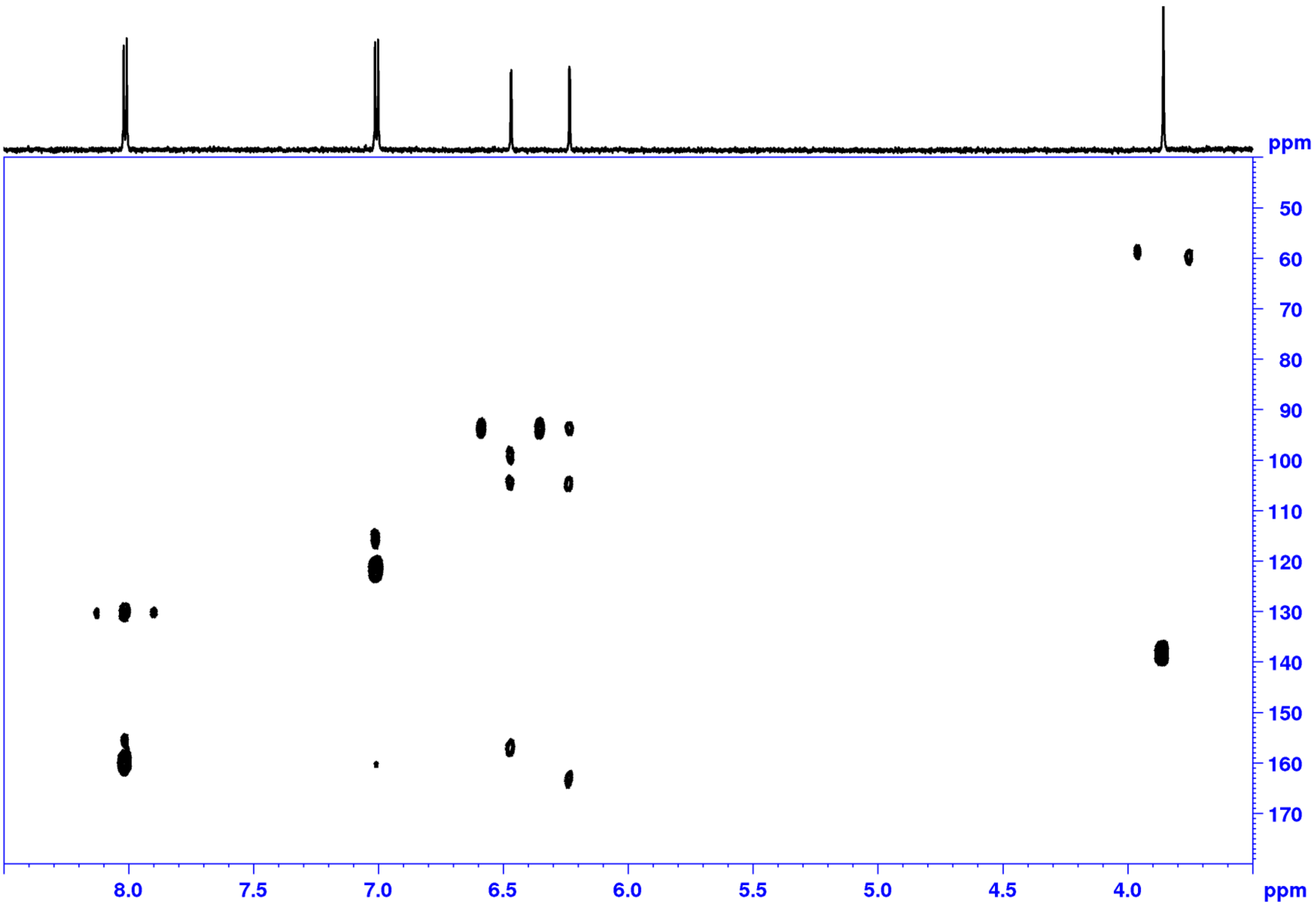


$^{1}H$-$^{13}C$ HMBC spectrum of kaempferol-3-methyl ether (isokampferide) in acetone-$d_6$

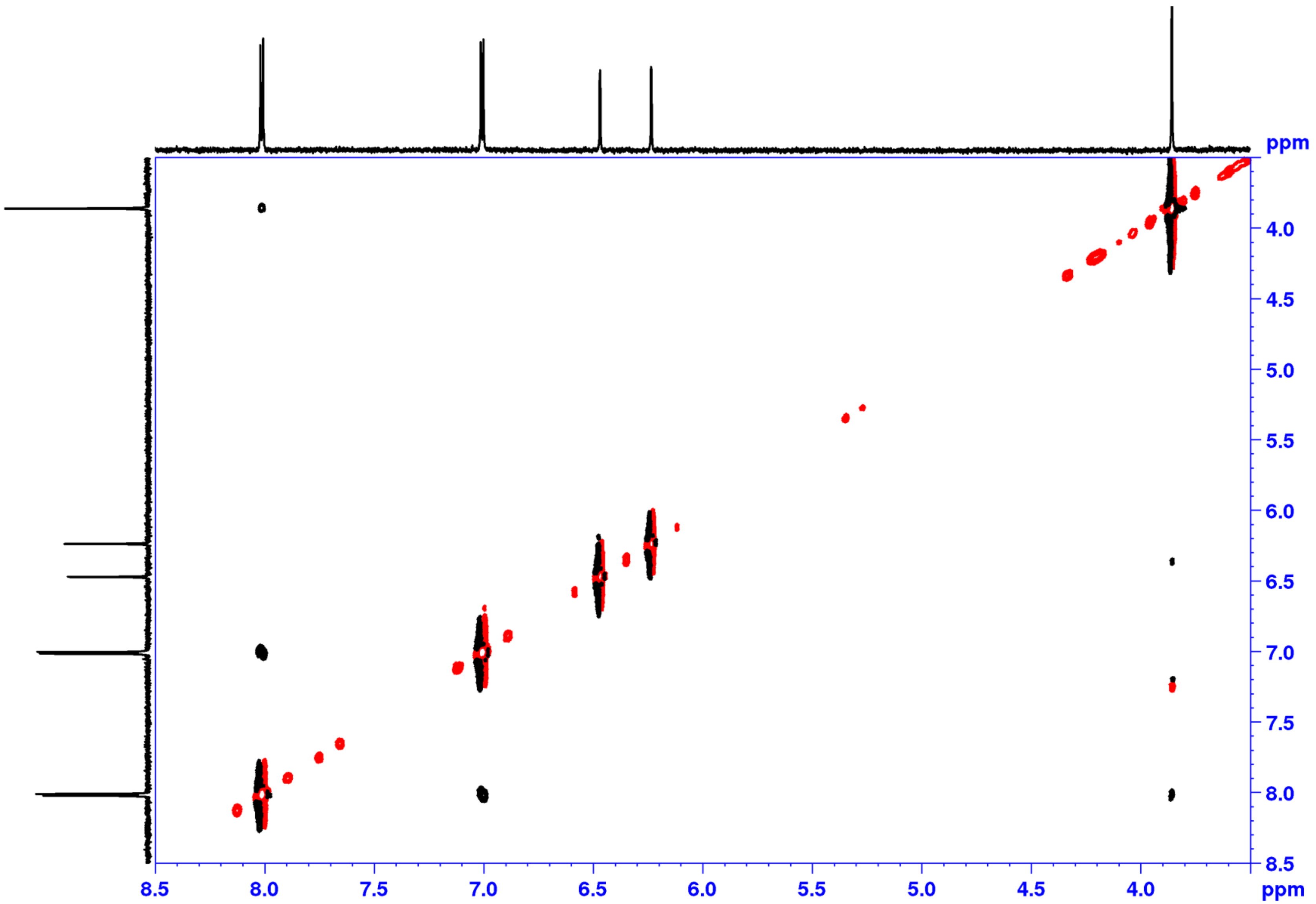


$^1H$-$^1H$ ROESY spectrum of kaempferol-3-methyl ether (isokampferide) in acetone-$d_6$

# 2-hydroxynaringenin
# (500 MHz, 269 K)

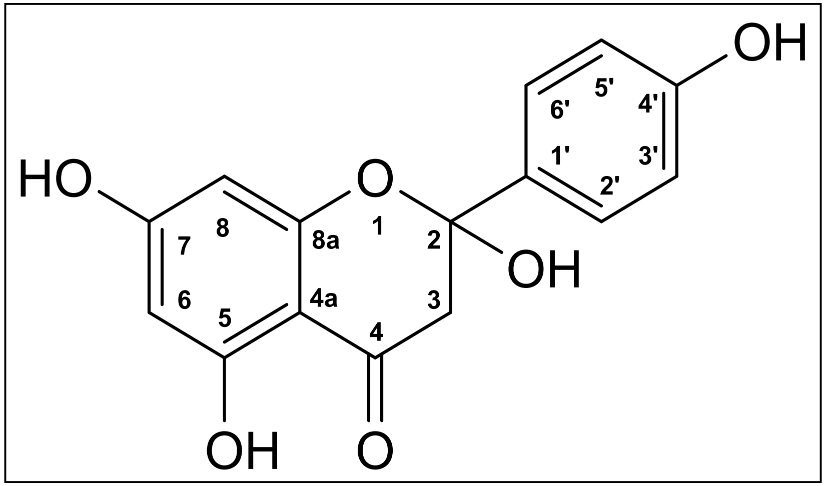


*Phytochemistry* **2016**, 125, 73-87.
https://doi.org/10.1016/j.phytochem.2016.02.013

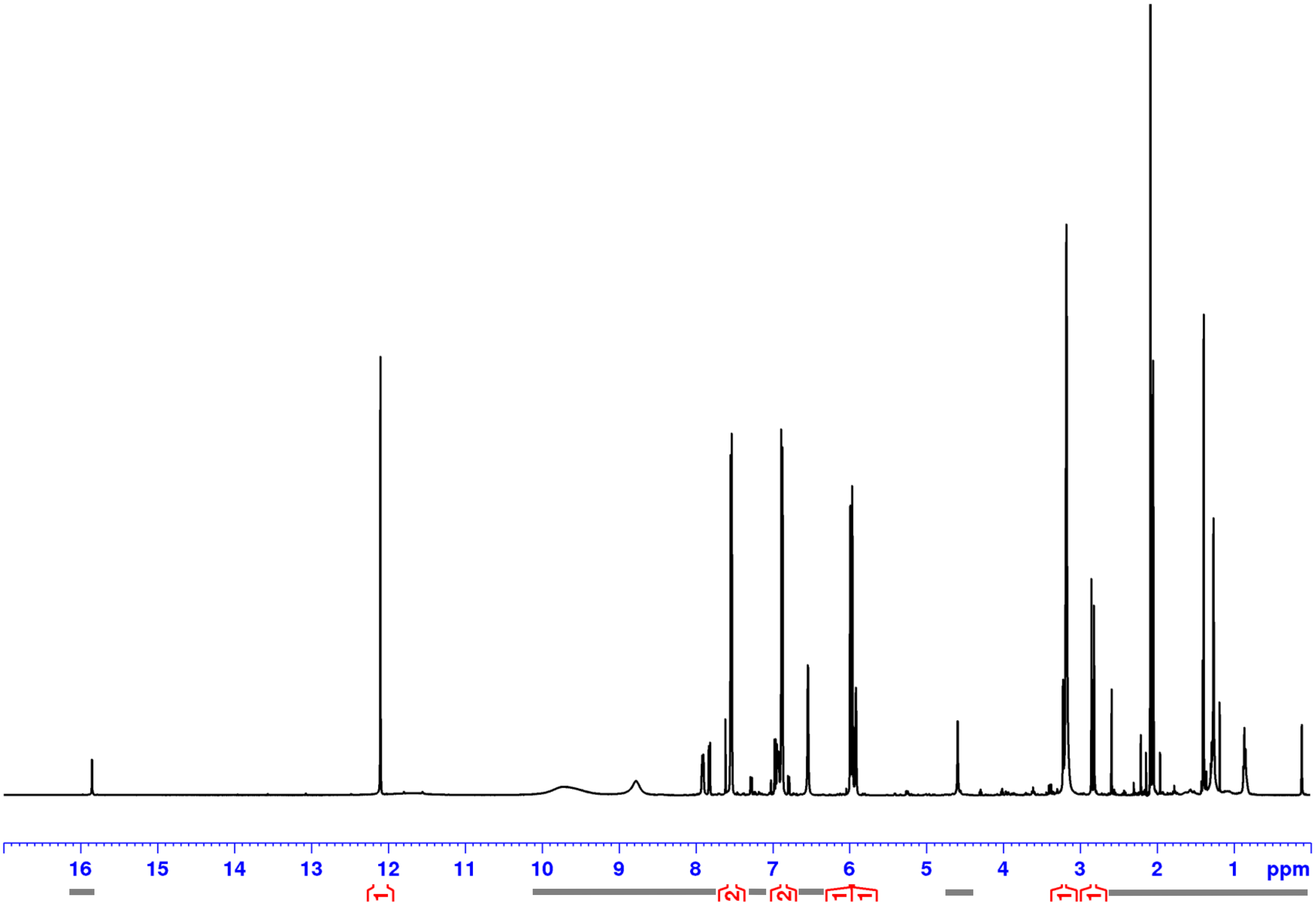


$^{1}$H NMR spectrum of 2-hydroxy naringenin in acetone-$d_6$ at 269 K (grey bars indicate impurities)

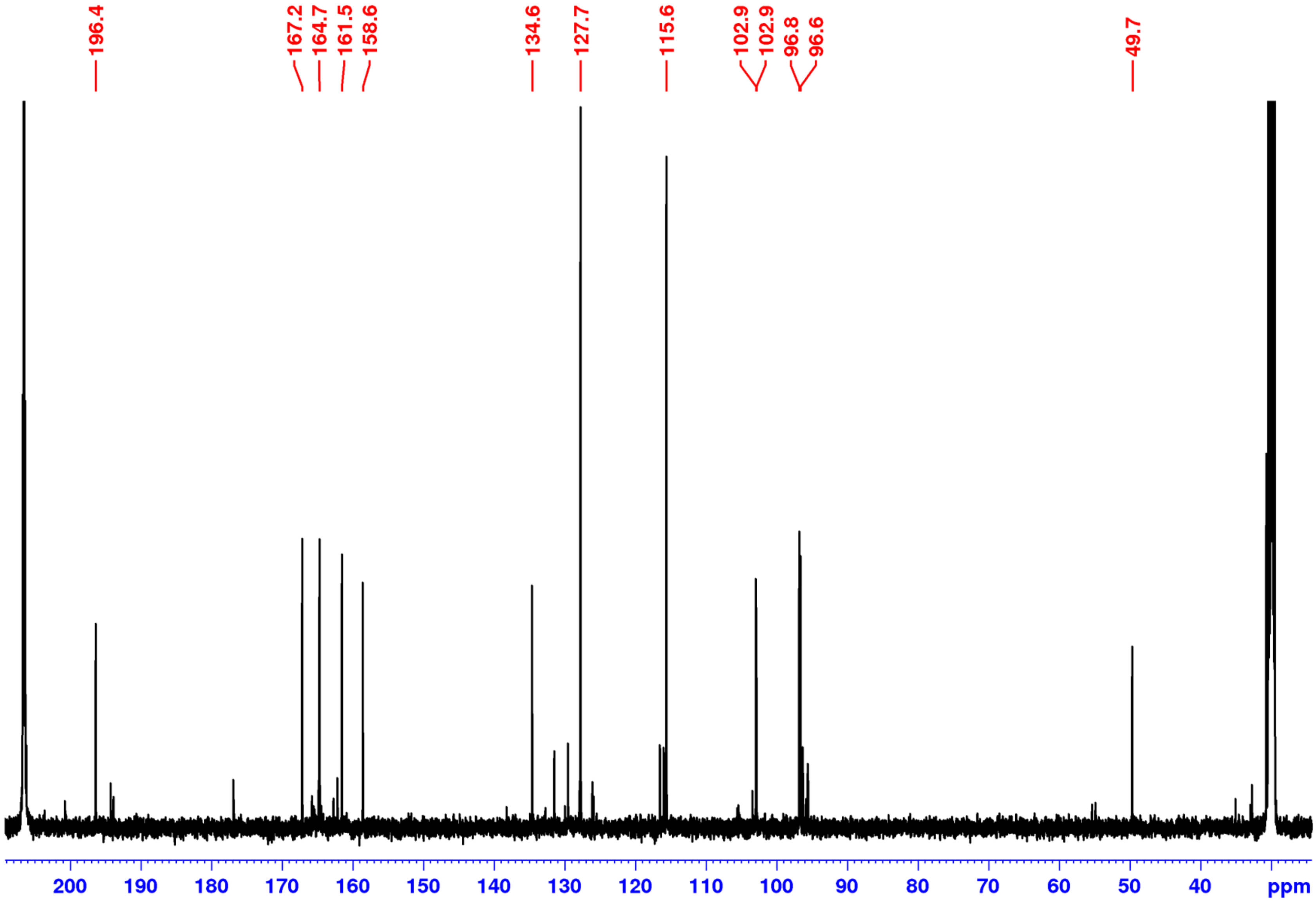


$^{13}C$ NMR spectrum of 2-hydroxy naringenin in acetone-$d_6$ at 269 K

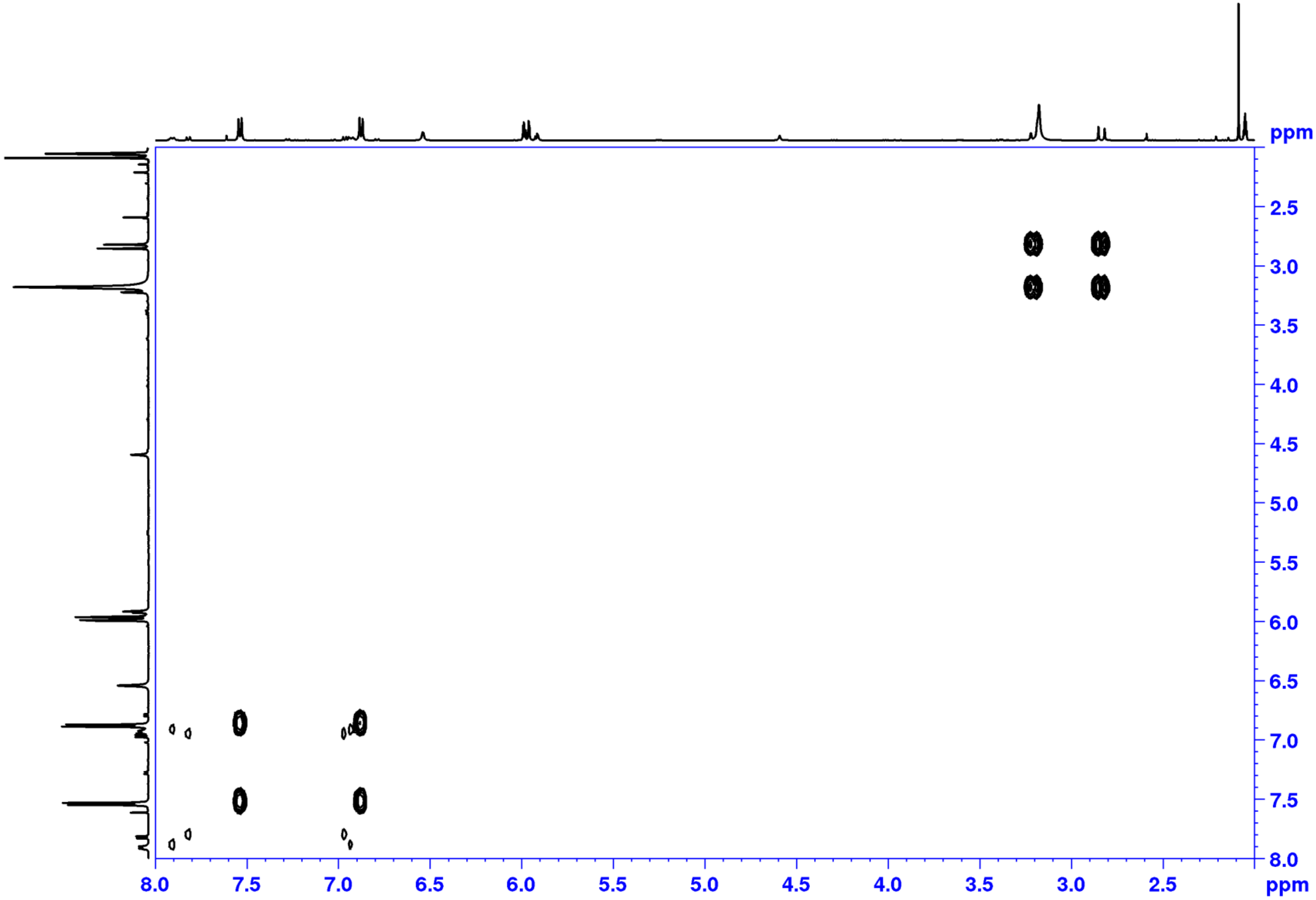


$^{1}$H-$^{1}$H COSY spectrum of 2-hydroxy naringenin in acetone-$d_6$ at 269 K

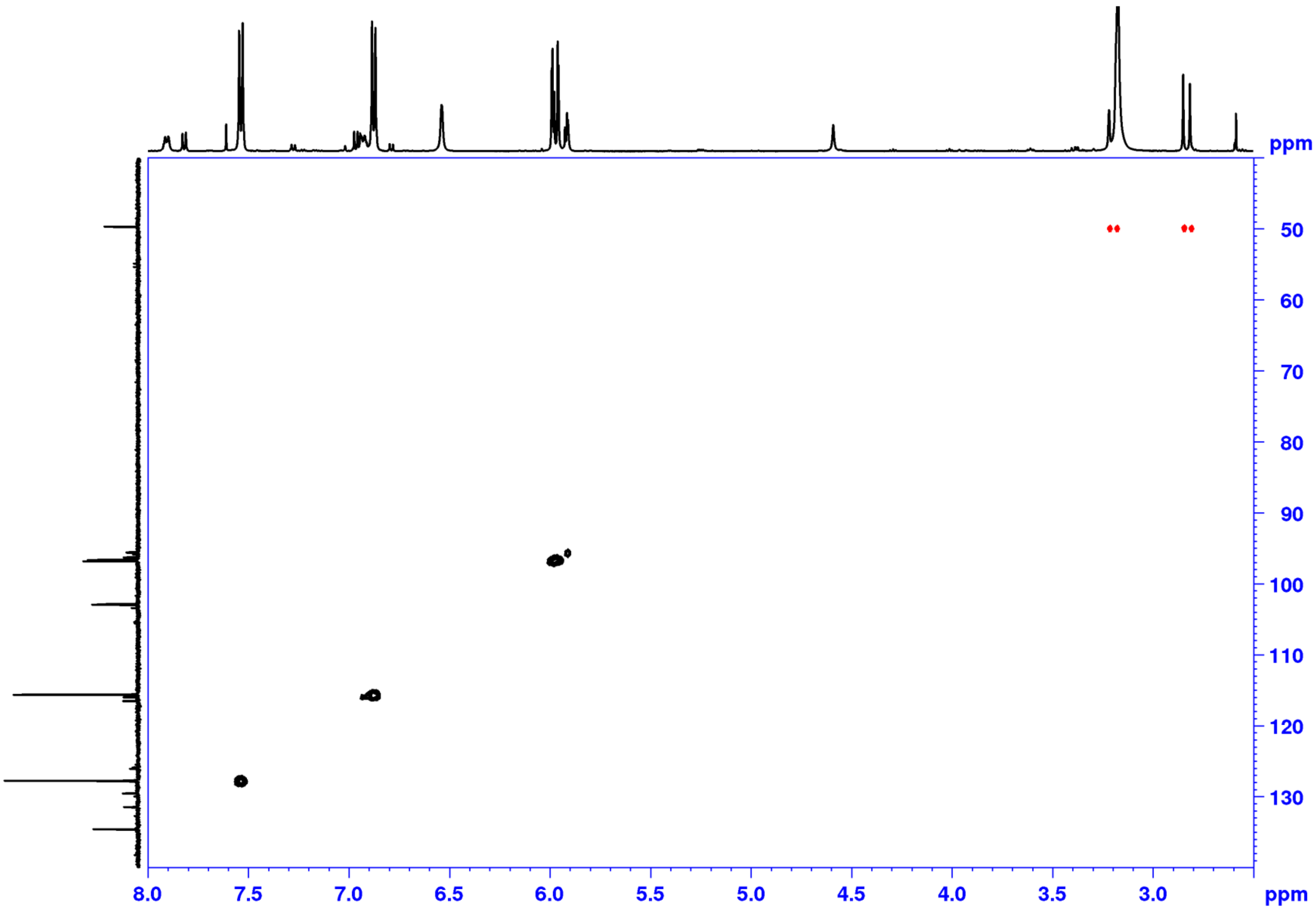


$^{1}H$- $^{13}C$ HSQC spectrum of 2-hydroxy naringenin in acetone-$d_6$ at 269 K

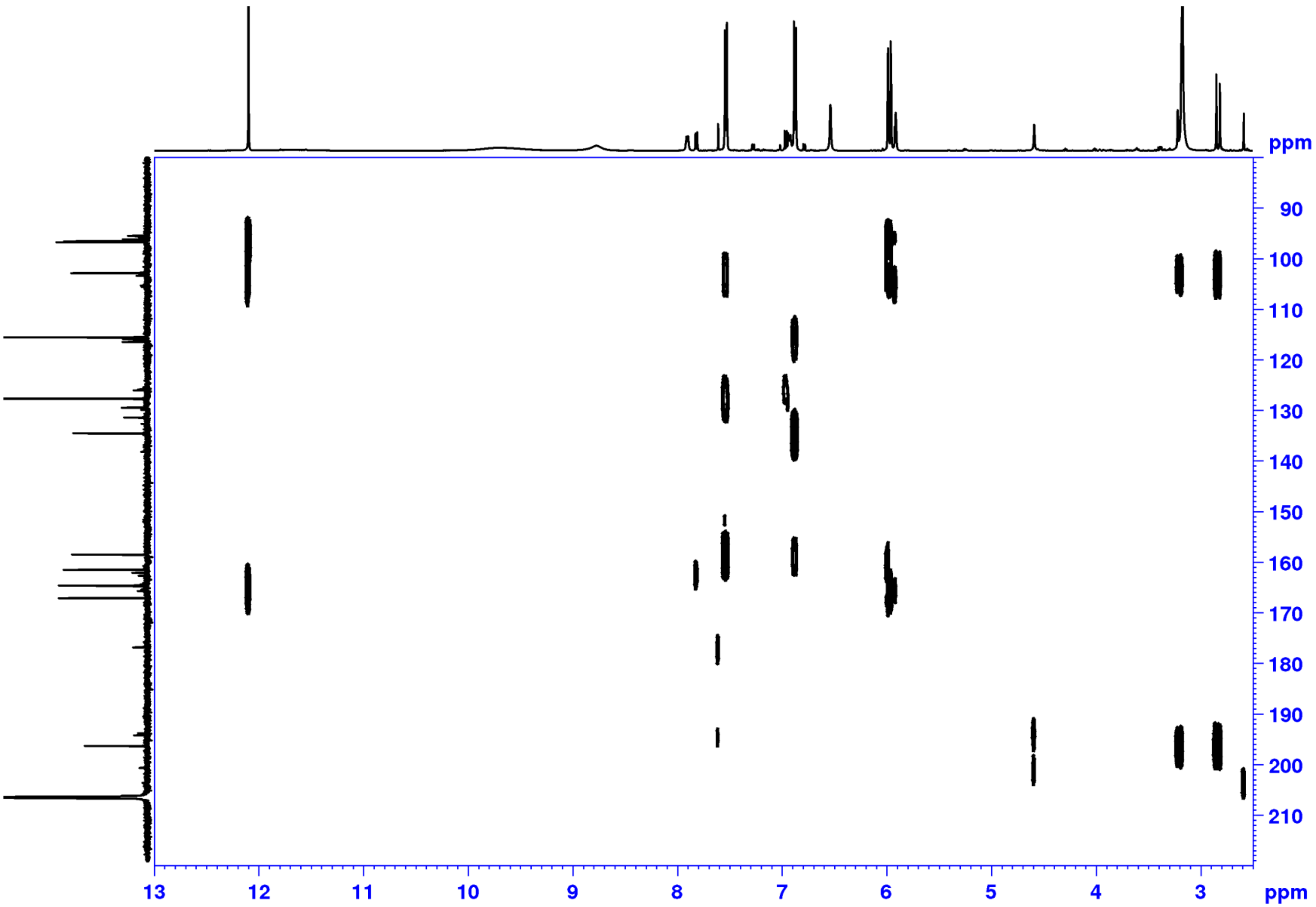


$^{1}H$- $^{13}C$ HMBC spectrum of 2-hydroxy naringenin in acetone-$d_6$ at 269 K

# Xilonenin
# (700 MHz)

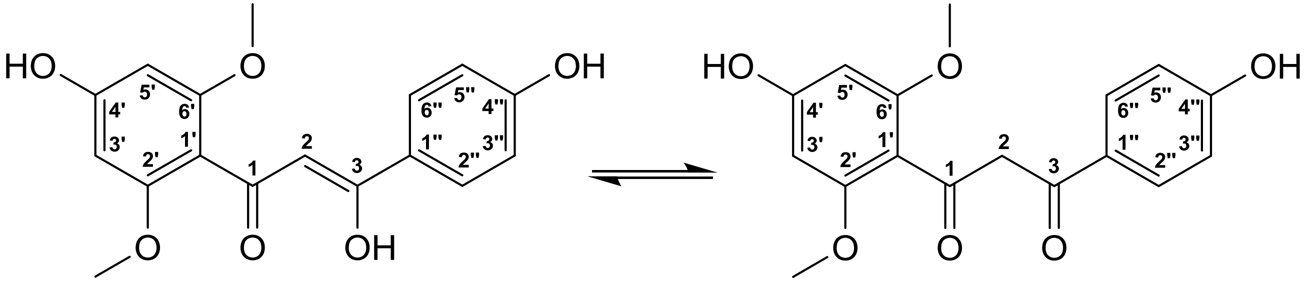

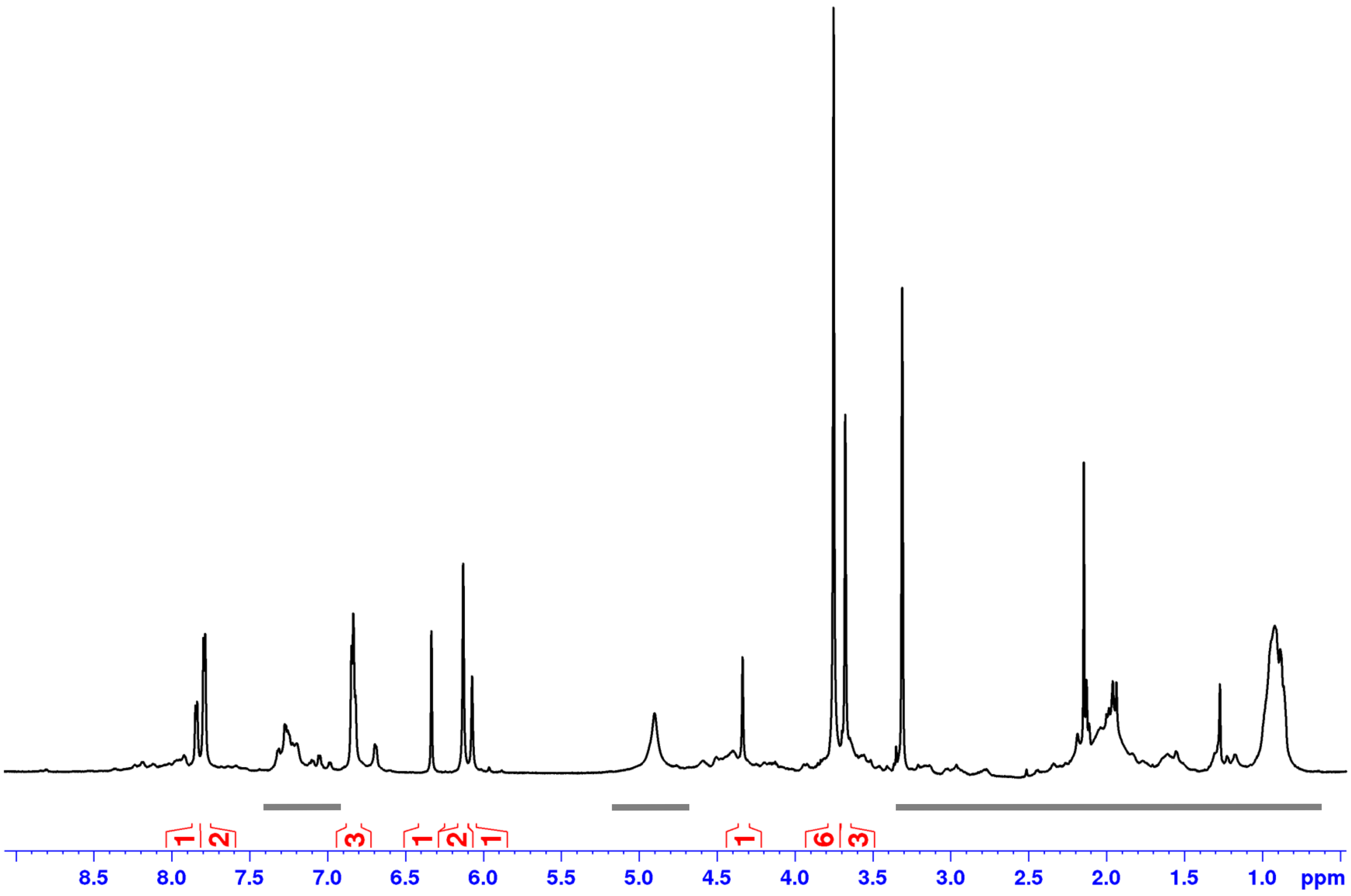


$^{1}$H NMR spectrum of xilonenin in MeOH-$d_3$ (grey bars indicate impurities)

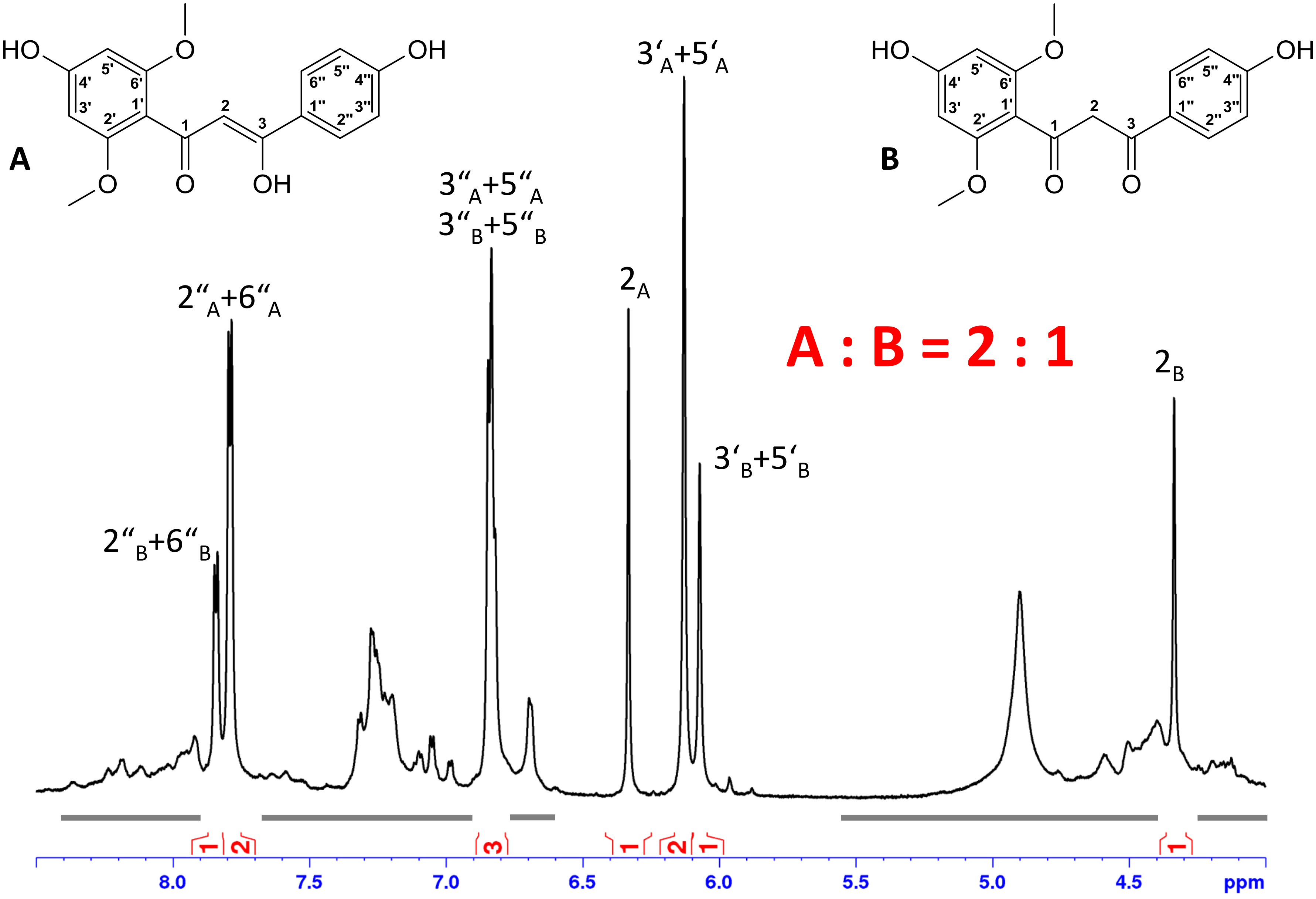


Detail of the $^{1}$H NMR spectrum of xilonenin in MeOH-$d_3$ (grey bars indicate impurities)

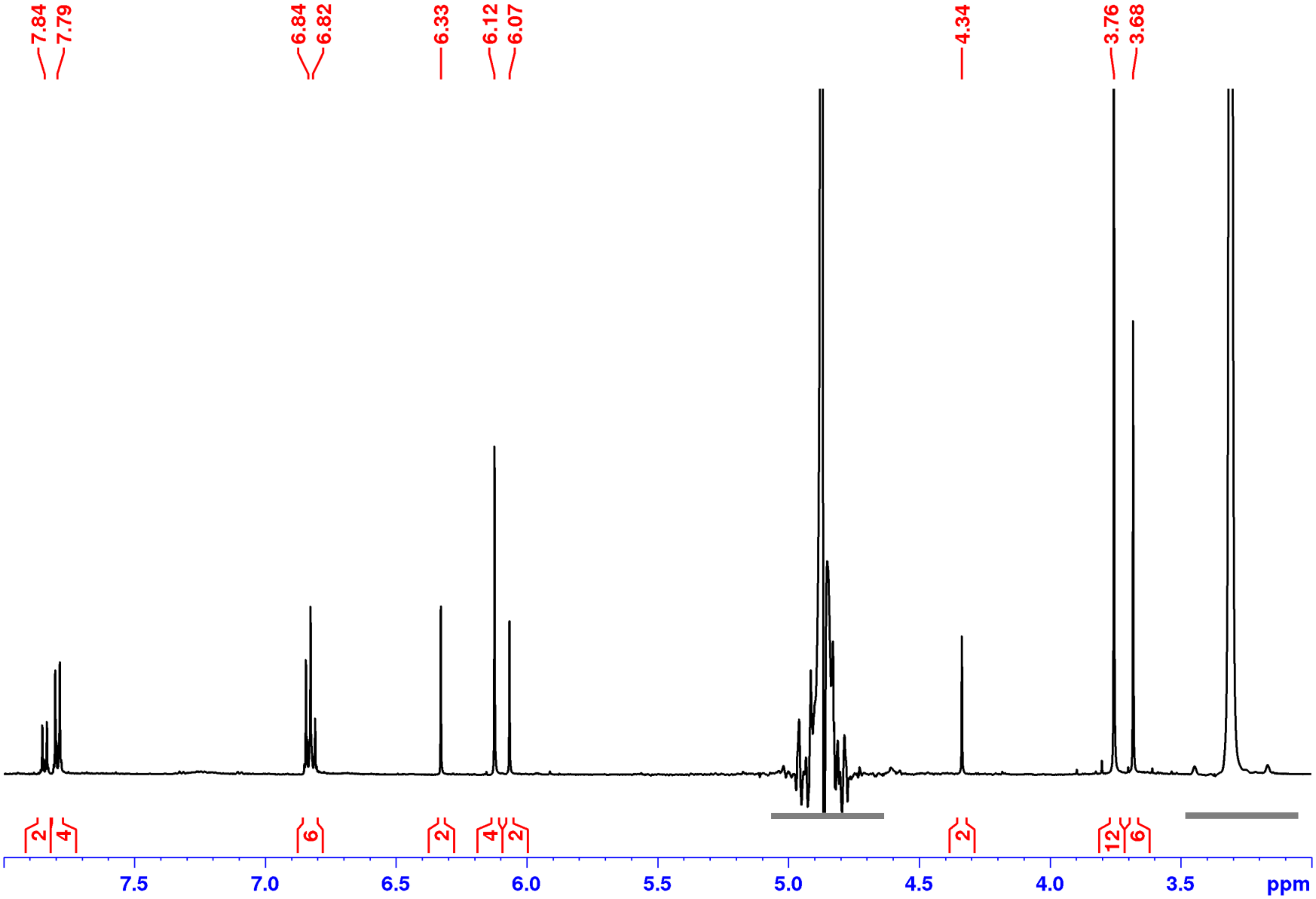


$^1$H NMR spectrum of purified xilonenin in MeOH-$d_3$ (grey bars indicate impurities)

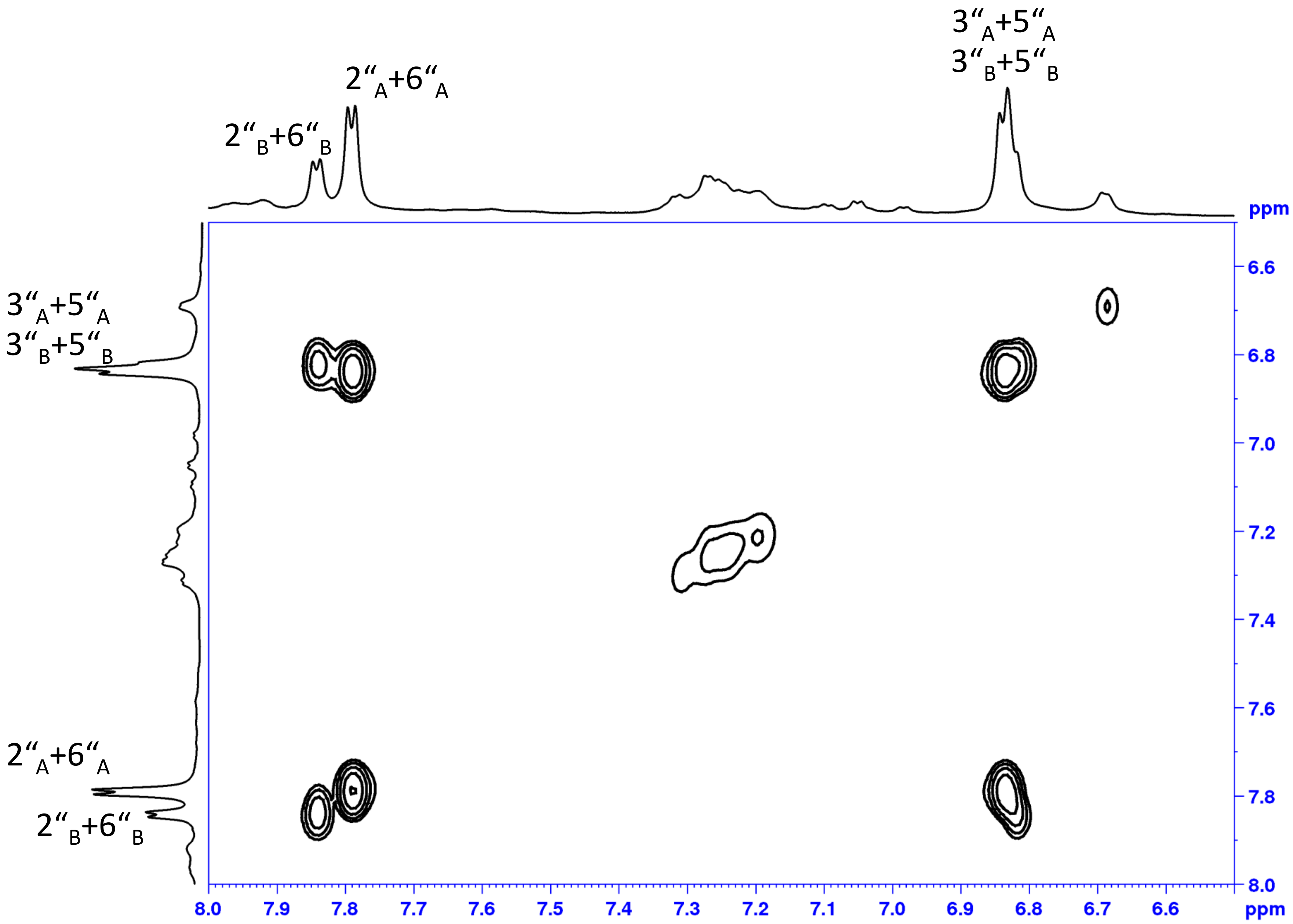


Detail of the $^{1}$H-$^{1}$H COSY spectrum of xilonenin in MeOH-$d_3$

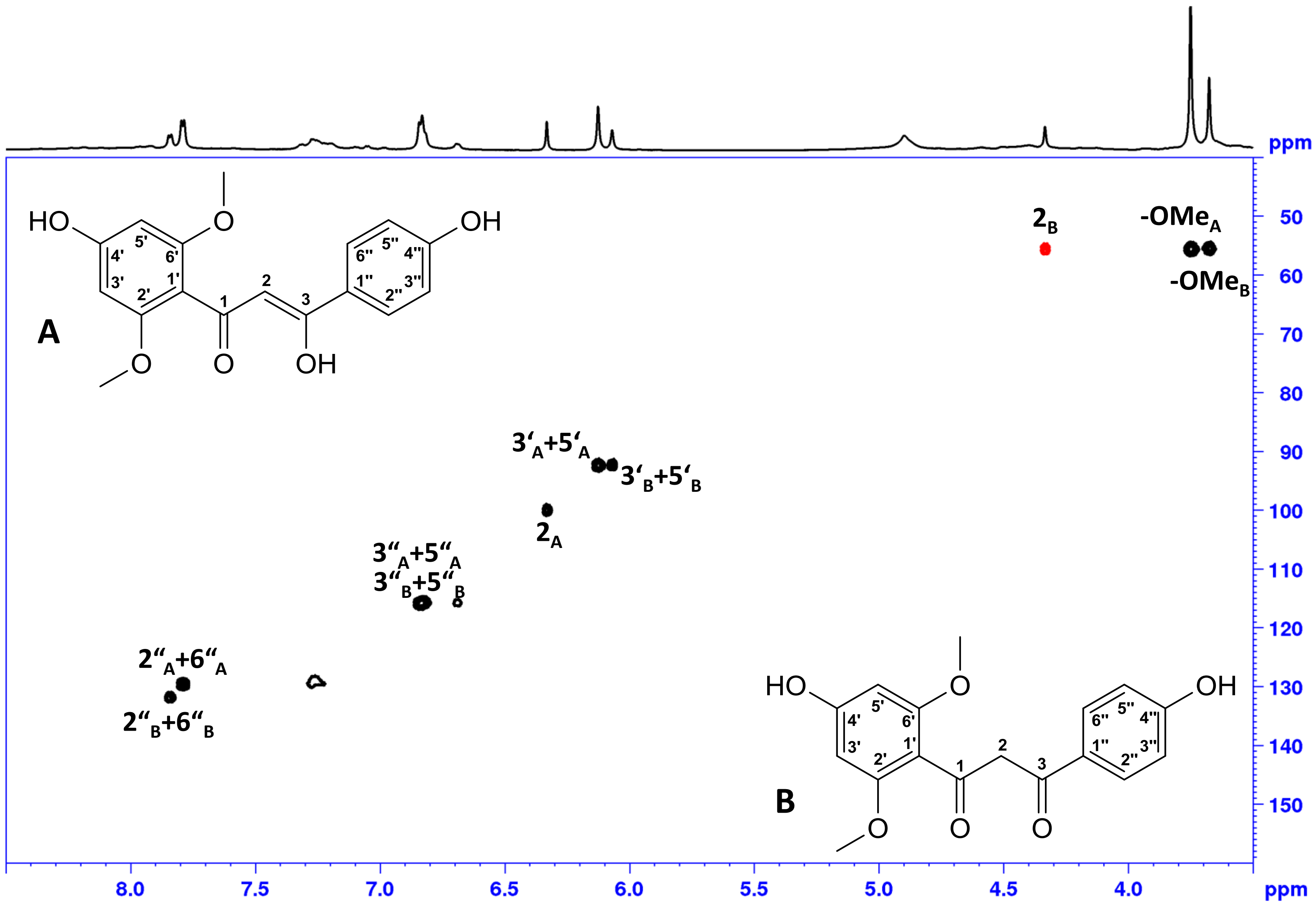


$^{1}H$-$^{13}C$ HSQC spectrum of xilonenin in MeOH-$d_3$

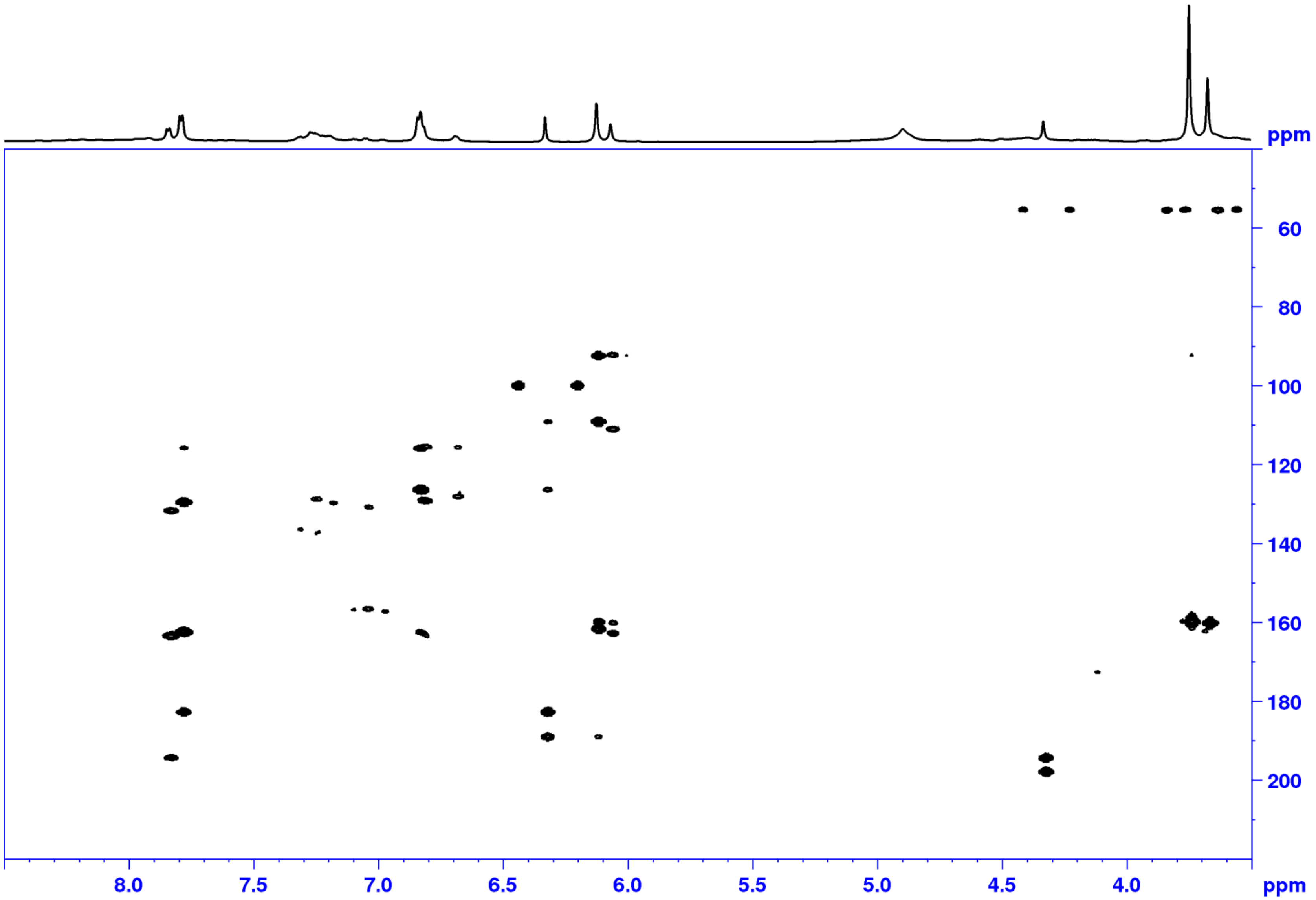


$^{1}H$-$^{13}C$ HMBC spectrum of xilonenin in MeOH-$d_3$

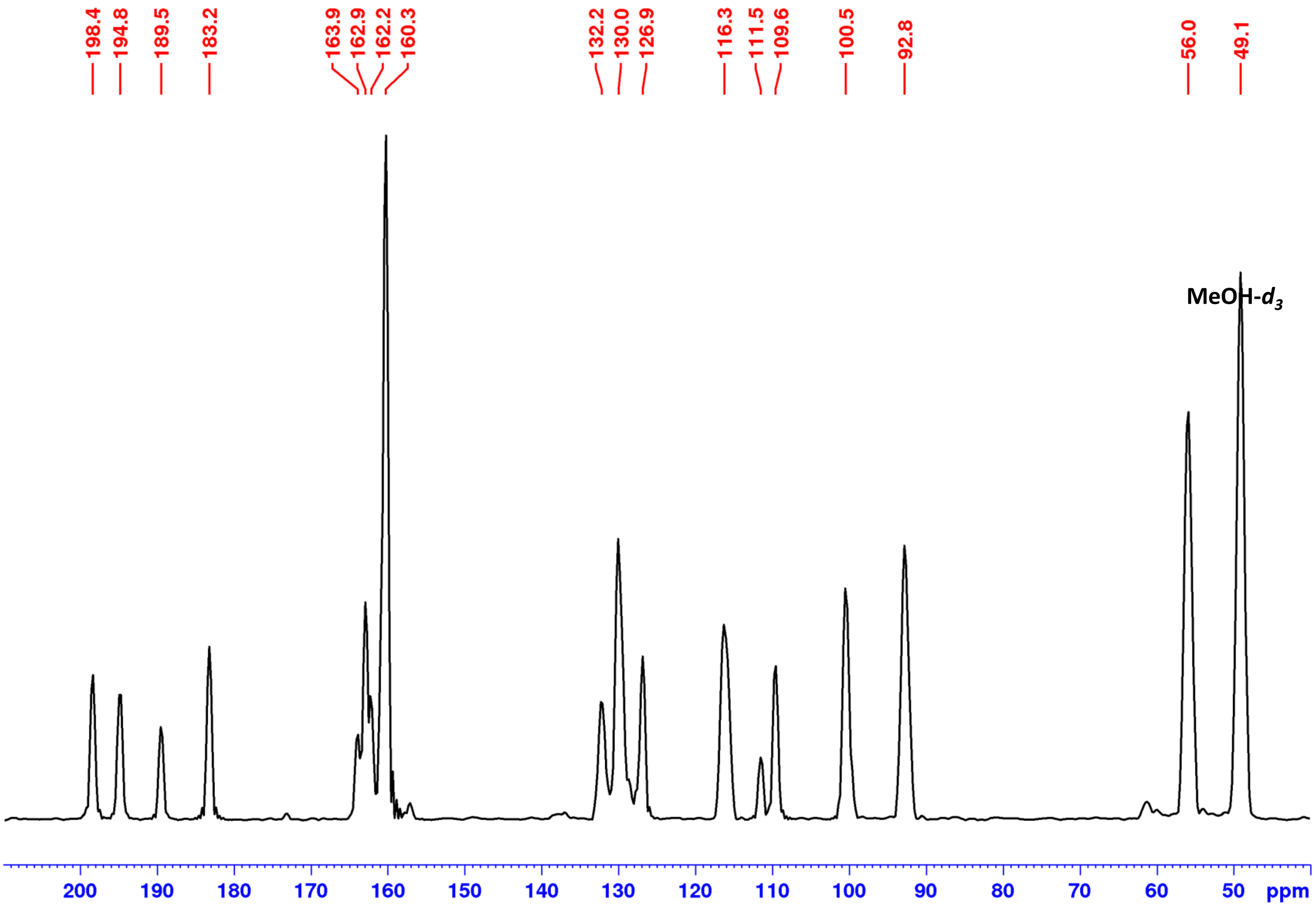


$^{13}$C-chemical shifts of xilonenin calculated from F1-projections of the $^{1}$H-$^{13}$C HSQC and HMBC spectra

**NMR chemical shift data of xilonenin tautomers (in DMSO-$d_6$).** The enol form **(A)** and keto form **(B)**.

**A** **B**

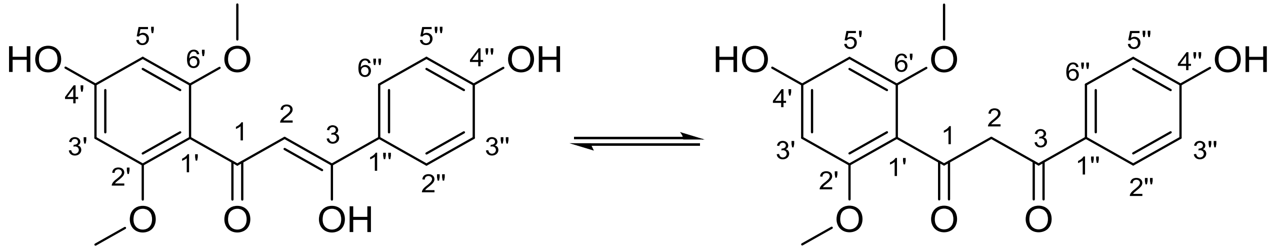


| pos. | $\delta_H$ | *mult.*, $J_{HH}$ [Hz] | $\delta_C$ |
|---|---|---|---|
| 1 | - | - | - |
| 2 | - | - | - |
| 3 | - | - | - |
| 1’ | - | - | - |
| 2’ | - | - | 158.2 |
| 3’ | 6.04 | *s* | 91.9 |
| 4’ | - | - | 160.6 |
| 5’ | 6.04 | *s* | 91.9 |
| 6’ | - | - | 158.2 |
| 1’’ | - | - | 124.2 |
| 2’’ | 7.61 | *d, 8.6* | 128.8 |
| 3’’ | 6.55 | *d, 8.4* | 116.2 |
| 4’’ | - | - | - |
| 5’’ | 6.55 | *d, 8.4* | 116.2 |
| 6’’ | 7.61 | *d, 8.6* | 128.8 |
| $-OCH_3$ | 3.63 | *s* | 55.2 |

| pos. | $\delta_H$ | *mult.*, $J_{HH}$ [Hz] | $\delta_C$ |
|---|---|---|---|
| 1 | - | - | 196.0 |
| 2 | 4.11 | *s* | 54.7 |
| 3 | - | - | 191.0 |
| 1’ | - | - | 109.5 |
| 2’ | - | - | - |
| 3’ | 6.04 | *s* | 91.9 |
| 4’ | - | - | - |
| 5’ | 5.98 | *s* | 91.9 |
| 6’ | - | - | - |
| 1’’ | - | - | - |
| 2’’ | 7.62 | - | 130.9 |
| 3’’ | 6.55 | *d, 8.4* | 116.2 |
| 4’’ | - | - | 169.0 |
| 5’’ | 6.53 | - | 116.2 |
| 6’’ | 7.62 | - | 130.9 |
| $-OCH_3$ | 3.60 | *s* | 55.2 |

**Annotated $^1$H NMR spectrum of xilonenin tautomers in DMSO-$d_6$.**The enol form **(A)** and keto form **(B)**.

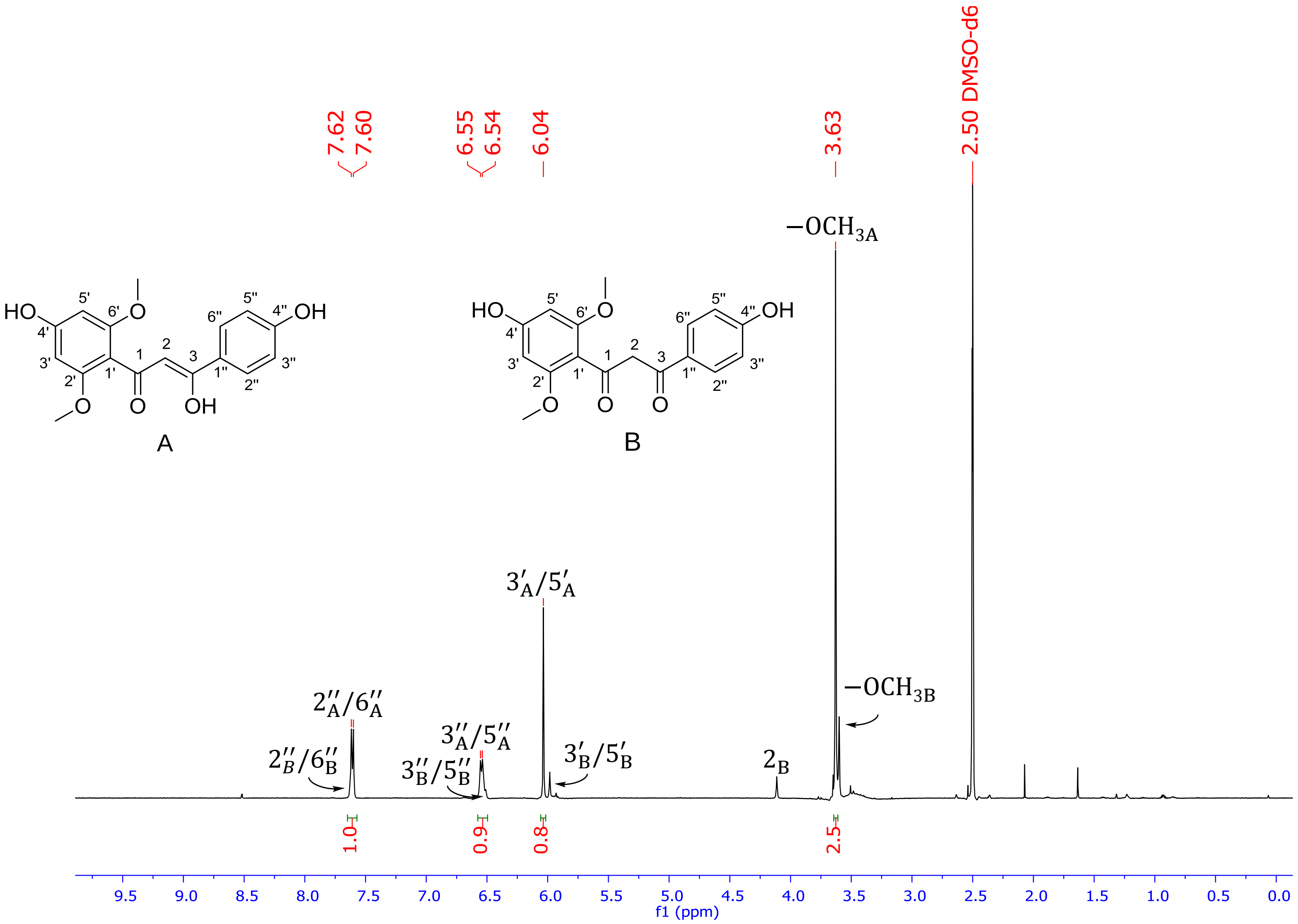

**Annotated ¹H NMR spectrum (3.5 – 8.0 ppm) of xilonenin tautomers in DMSO-$d_6$.** The enol form **(A)** and keto form **(B)**.

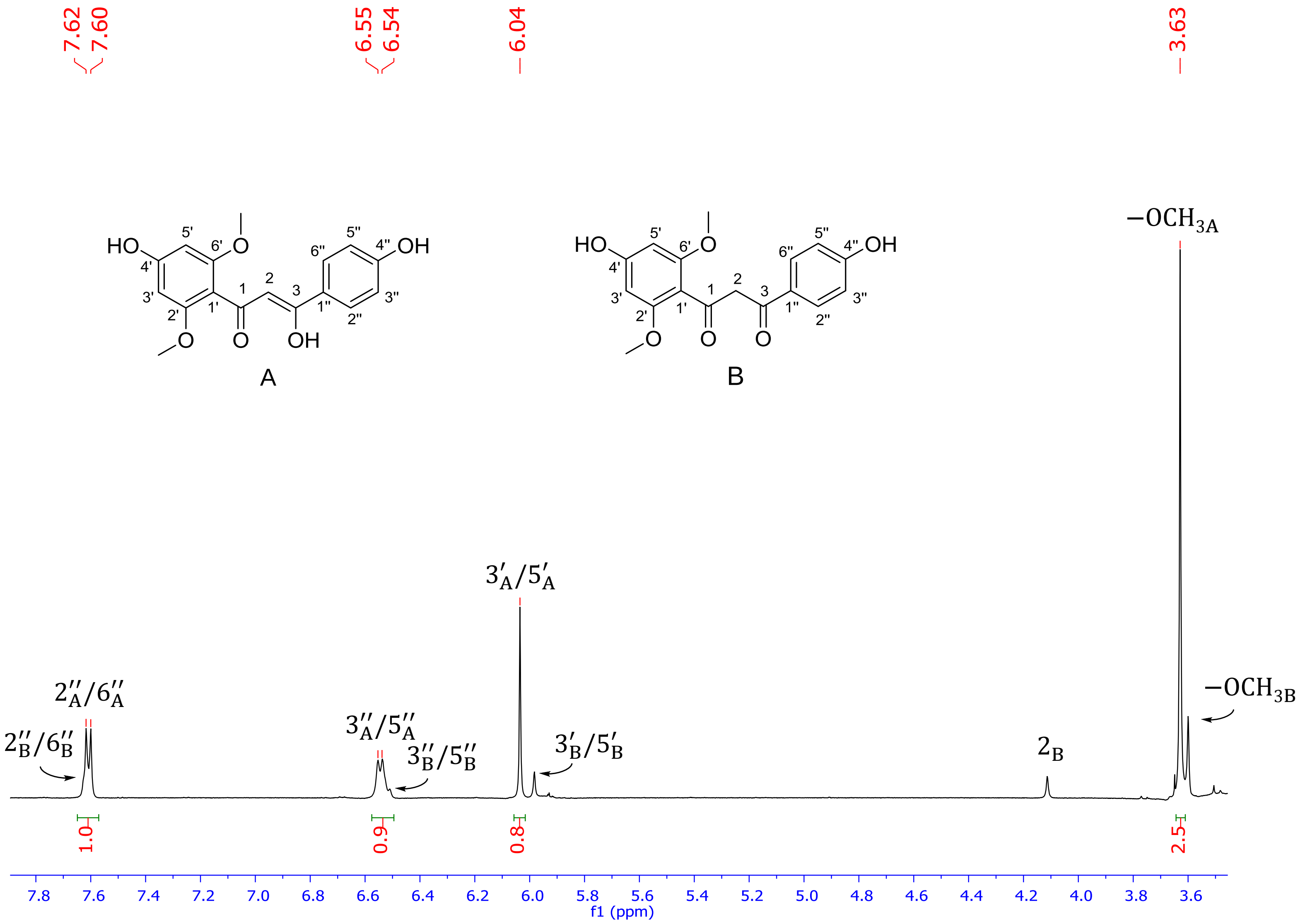

**Annotated COSY spectrum of xilonenin tautomers in DMSO-$d_6$.**The enol form **(A)**.

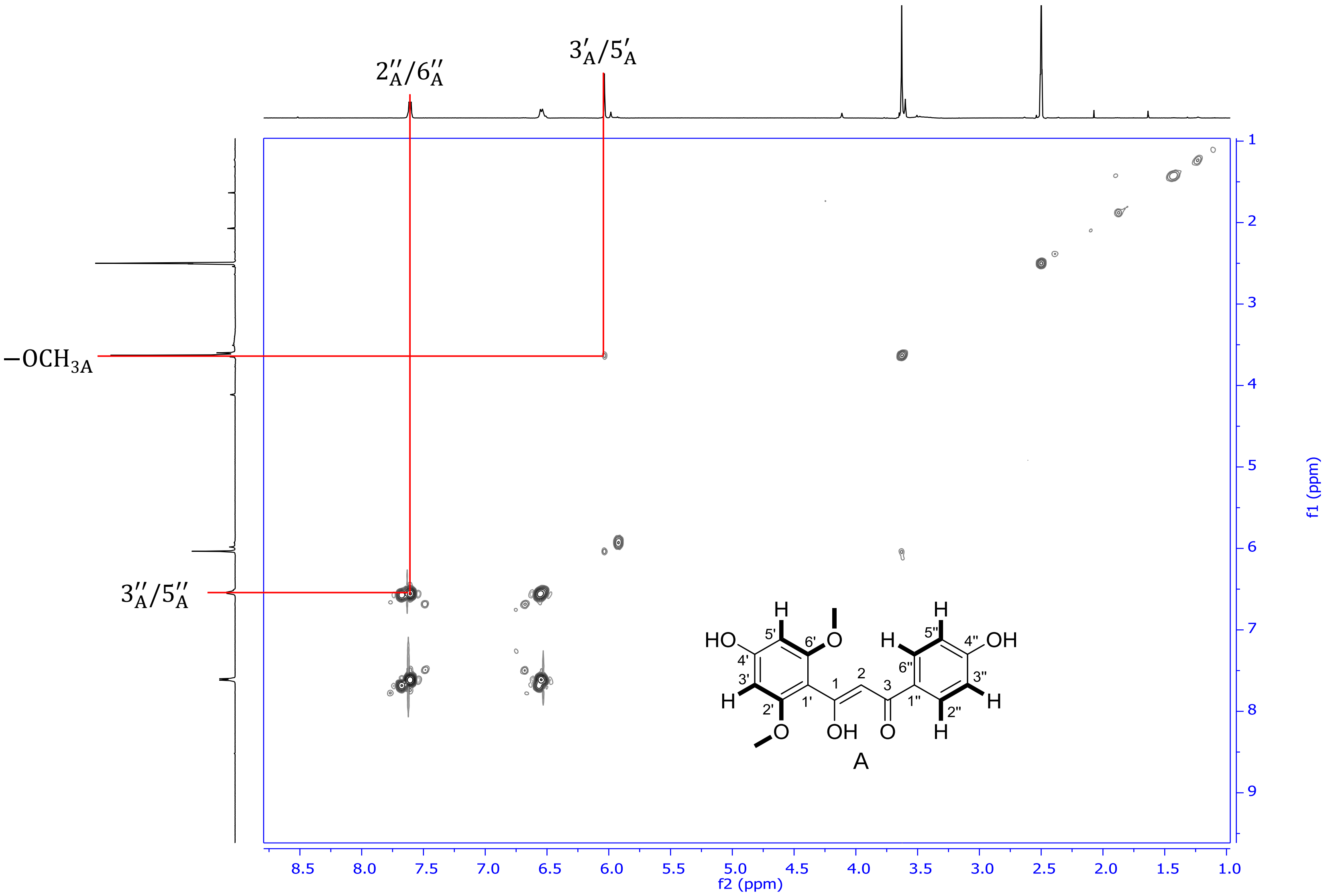

**Annotated HSQC spectrum of xilonenin tautomers in DMSO-$d_6$.**The enol form **(A)** and keto form **(B)**.

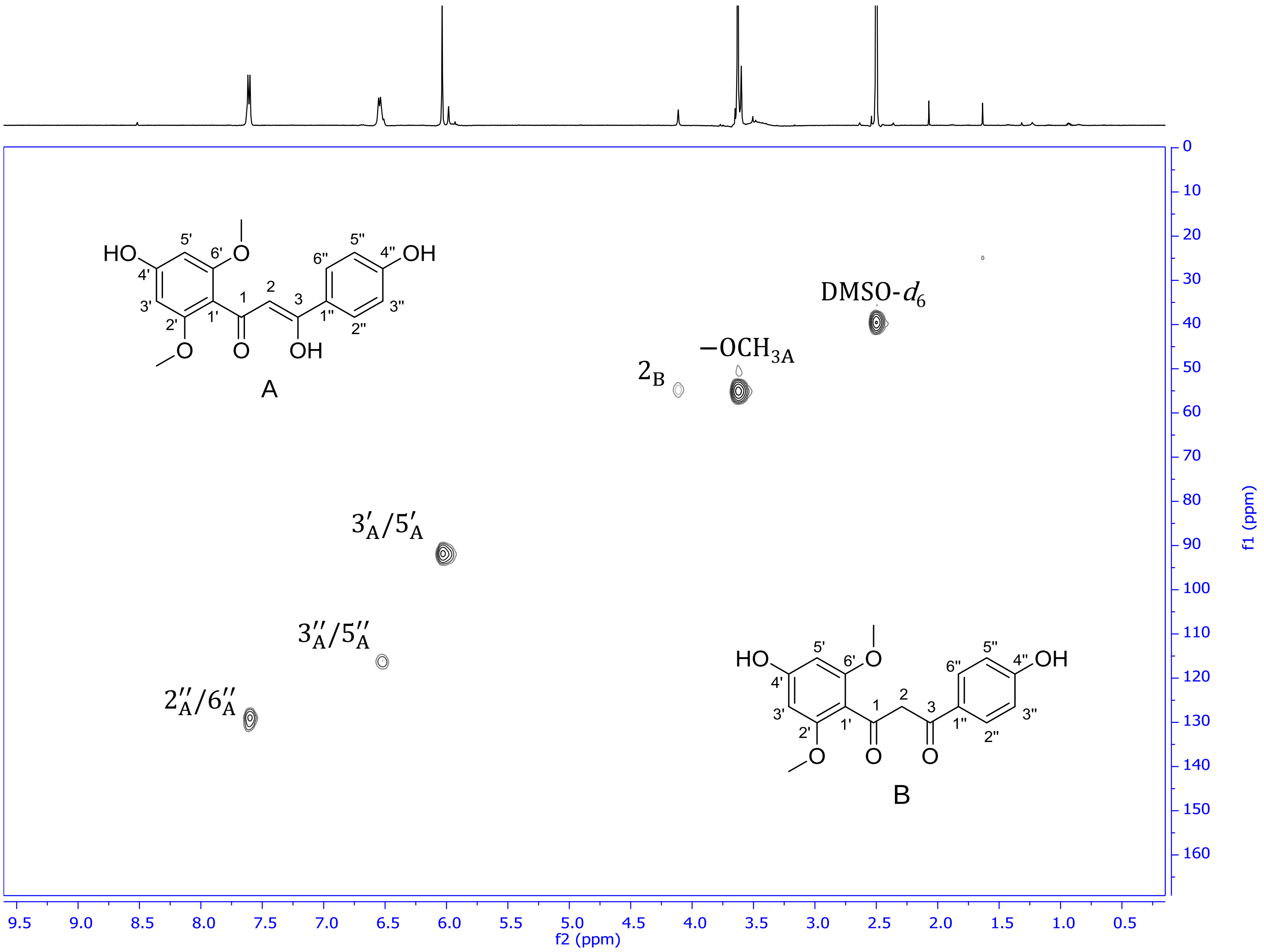

**Annotated HMBC spectrum of xilonenin tautomers in DMSO-$d_6$.**The enol form **(A)** and keto form **(B)**.

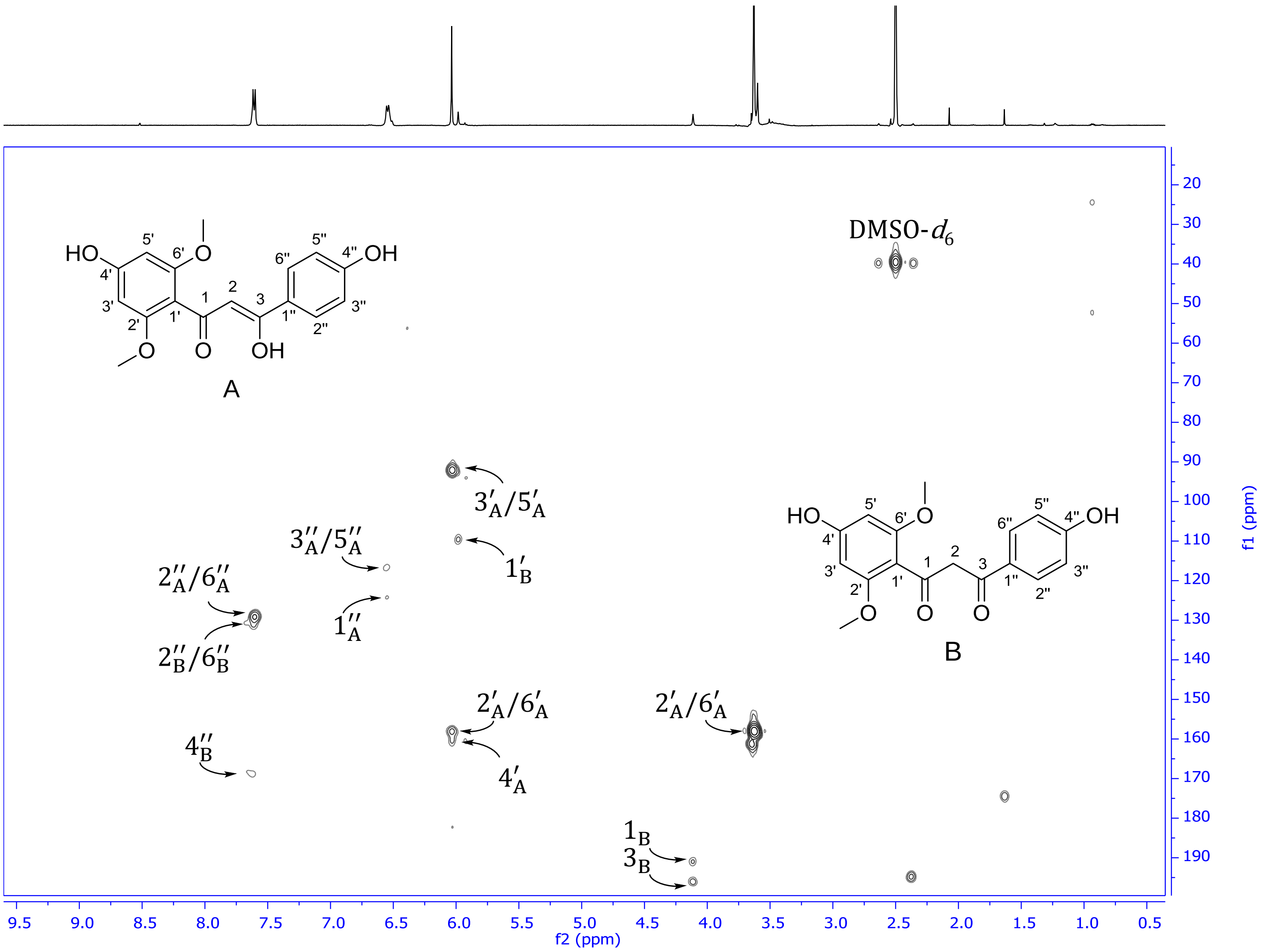

Supplement: kiab496_Supplementary_Data [file kiab496_supplementary_data.zip › kiab496-suppl_data/Supplemental Dataset S2.pdf]
